# Proceedings of the International Scientific Conference AIFI 2017. Therapeutic Exercise: Foundations, Evidences and Clinical Reasoning in Physiotherapy Practice: Rome, Italy. 12-13 October 2017

**DOI:** 10.1186/s40945-019-0069-0

**Published:** 2019-12-17

## S1 Translating motor control principles to practical applications in rehabilitation

### Mindy F. Levin (mindy.levin@mcgill.ca)

#### Professor, School of Physical and Occupational Therapy, 3654 Promenade Sir William Osler, Montreal, H3G 1Y5, Canada

Physiotherapists need to incorporate models of motor control and motor learning into their conceptual framework for clinical practice. Here, we consider how the nervous system organizes the action of a large number of body segments and joints in order to maintain reaching accuracy in motor tasks such as reaching from sitting or standing. Reaching can be accomplished by different combinations of joint movements permitting the system to adapt to unexpected situations, a process known as motor equivalence. Motor equivalence is defined as the set of combinations of different joint rotations (degrees of freedom) used to perform the same motor action. Following a stroke or damage to the central nervous system, deficits in motor planning and execution may ensue, leading to a reduced capacity to use the affected upper limb to meaningfully interact with objects in the environment. The capacity for adaptability depends on the residual ability of the nervous system to use different combinations of joint rotations to find solutions to motor problems. This capacity is limited in patients with hemiparesis due to decreases in the redundancy of the motor system, where redundancy is defined as a larger than needed number of movements available to the system. Reductions in redundancy may be related to deficits in threshold control and the specification of referent body postures.

Examples of how the stroke-damaged nervous system organizes reaching movements based on limited redundancy are presented while considering the extent to which compensatory motor patterns are adaptive. Key messages are that patients with chronic hemiparesis use excessive trunk movement even for reaches to close targets to assist hand transport during reaching [1,2] to assist in orienting the hand for grasping [3] and to assist arm swinging in standing and during walking [4]. In addition, for simple reaching tasks, when the trunk is involved, it is recruited (spatially and temporally) as an integral part of the reaching movement.

Compensatory trunk movement can also be adaptive. People with stroke use excessive trunk movement and arm-plane motion to compensate for limited shoulder flexion and elbow extension. Further investigation of adaptability is illustrated with results of studies of kinematic adaptability to sudden perturbation of the trunk when reaching from sitting [5] and when reaching from standing [6]. These studies show that people with even mild stroke have difficulty in rapidly changing elbow-shoulder interjoint coordination patterns to adapt reaching movements to sudden perturbation of trunk motion.

The ability to appropriately adapt interjoint coordination to changing task conditions is impaired in individuals with stroke, which may be explained by impairments in threshold control leading to deficits in the specification of referent body configurations for control of reaching.

Deficits in higher order motor control skills related to the use of motor compensations to adapt to unexpected situations, may restrict motor recovery. This capacity is not routinely identified in commonly used clinical scales. Recommendations for treatment approaches to increase redundancy and motor equivalence include the restriction of compensations during practice and encouraging the patient to explore the environment and find new solutions to motor problems.

**References**

1. Levin MF, Michaelsen S, Cirstea C, Roby-Brami A. Use of the trunk for reaching targets placed within and beyond the reach in adult hemiparesis. Exp Brain Res. 2002;143:171-80.

2. Michaelsen SM, Levin MF. Short-term effects of practice with trunk restraint on reaching movements in patients with chronic stroke: a controlled trial. Stroke. 2004;35:1914-19.

3. Roby-Brami A, Jacobs S, Bennis N, Levin MF. Hand orientation for grasping and arm joint rotation patterns in healthy subjects and hemiparetic stroke patients. Brain Res. 2003;969:217-29.

4. Ustinova KI, Goussev VM, Balasubramaniam R, Levin MF. Disruption of co-ordination between arm, trunk and center of pressure displacement in patients with hemiparesis. Motor Control. 2004;8:139-59.

5. Shaikh T, Goussev V, Feldman AG, Levin MF. Arm-trunk coordination for beyond the reach movements in adults with hemiparesis. Neurorehabil Neural Rep. 2014;28(4):355-66.

6. Tomita Y, Mullick AA, Levin MF. Reduced kinematic redundancy and motor equivalence during whole-body reaching in individuals with chronic stroke. Neurorehabil Neural Rep. 2018;32(2):175-86.

## S2 The technologies as tools for controlling the patient-environment relationship

### Maurizio Petrarca (mauriziopetrarca@gmail.com)

#### “Bambino Gesù” Children’s Hospital, Department of Neurosciences, Rome, Italy

In rehabilitation the therapeutic relation between the patient and the therapist is largely out of control due to the huge amount of variables that run simultaneously during the training. In the last 30 years many technologies were introduced in the fields of the rehabilitation mainly represented by systems for motion analysis and by robotic devices. Motion analysis systems allowed the gathering of a large extent of synchronized variables permitting the multifactorial analysis of the movement [1]. The analysis of the movement offered the opportunity to observe elements that usually are not visible like the forces exchanged between the subject and the environment, i.e., the terrain in the case of gait. Furthermore, it allowed to observe the muscle activities, that is, the forces utilized by the subject to balance the body inertia and the external reaction forces. When these elements are combined with the body movements, hypothesis on the motor strategies adopted by the single subject emerge. In neurological fields these methodologies are changing the interpretation of the movement organization in pathologic conditions, conditioning the clinical decision making process on surgery intervention, drugs administration and motor training. The current challenges in this field are moving towards the searching of variables synthesis for the decision making process and towards the analysis of the subject cognition and perception of the movement. The movement synthesis faced mainly with two different strategies: the personalization and accurate modeling of the movement; and the use of artificial intelligence for clustering and interpreting the data streaming emerging from movement analysis. The main limits of these approaches are represented by the lack of an internal representation of the state of the single subject, that is, a model of the patient internal process of decision.

Robotics allowed to dose the therapy. Usually robotic devices are developed for the training of a specific joint or limb in a specific task and context. They are introduced originally for executing repetitive tasks like isokinetic training or for executing tasks otherwise not manageable, i.e., the gait training. More recently, they are proposed as useful tools for substituting ‘traditional’ therapy [2]. Indeed, the real added value of robotics in rehabilitation is represented by the possibility to control the therapeutic relation. The restriction of the task and of the context is a limit from a therapeutics perspective, but allowed to observe the effect of the specific treatment on the function in conditions controlled and repeatable. What is mandatory is the correct analysis of the task, the context and the function object of the training. In that perspective the robotic device should be customized for dispensing the desired physical activity and for gathering the information on the relationship between the patient and the device during the training.

Combining the techniques of movement analysis, with the artificial intelligence and with robotics is opening new perspectives for studying ad for training the human function. The future evolution of the matter, but also of the concept of rehabilitation that is under construction [3], is not completely predictable considering the exponential evolution of the technologies and the early stage of the rehabilitation. What any rehabilitator need is a strong theory on motor control and learning, it is mandatory in order to not lose the route.

**References**

1. Benedetti MG, Beghi E, De Tanti A, Cappozzo A, Basaglia N, Cutti AG, Cereatti A, Stagni R, Verdini F, Manca M, Fantozzi S, Mazzà C, Camomilla V, Campanini I, Castagna A, Cavazzuti L, Del Maestro M, Croce UD, Gasperi M, Leo T, Marchi P, Petrarca M, Piccinini L, Rabuffetti M, Ravaschio A, Sawacha Z, Spolaor F, Tesio L, Vannozzi G, Visintin I, Ferrarin M. SIAMOC position paper on gait analysis in clinical practice: General requirements, methods and appropriateness. Results of an Italian consensus conference. Gait Posture. 2017 Oct;58:252-260.

2. Reinkensmeyer DJ, Burdet E, Casadio M, Krakauer JW, Kwakkel G, Lang CE, Swinnen SP, Ward NS, Schweighofer N. Computational neurorehabilitation: modeling plasticity and learning to predict recovery. J Neuroeng Rehabil. 2016 Apr 30;13(1):42.

3. Damiano DL. Activity, activity, activity: rethinking our physical therapy approach to cerebral palsy. Phys Ther. 2006 Nov;86(11):1534-40.

## S3 Effects of action observation on neonatal neuroplasticity

### Andrea Guzzetta (a.guzzetta@fsm.unipi.it)

#### IRCCS Stella Maris and University of Pisa

In the last years, growing evidence contributed to support the hypothesis that the motor system is part of a wider simulation network activated by a variety of conditions related to action, including motor imagery and action observation [1]. In the adult human brain, the existence of a system matching the observation and the execution of actions, defined by most as the mirror neuron system, is well established [2]. Surprisingly, very little is known about its emergence and early development.

Indeed, indirect evidence from ethologic and behavioral studies suggests that learning throughout observation of others is a key mechanism for developing social-emotional functions for communication and bonding, and cognitive functions for motor learning and goal prediction [3]. The development of new non-invasive tools to assess brain representation of complex functions, such as NIRS (Near-infrared Spectroscopy) or EEG (Electroencephalography), has recently allowed for more direct demonstrations of the presence of a sensory-motor matching system in infancy [4].

Action observation therapy has been found to be effective in improving hand motor function in both adults with stroke and children with unilateral cerebral palsy. In fact, while in adult stroke the main mechanism to restore the re-connection of the motor cortex with the spinal cord is the reorganisation of function within the ipsilesional cortex, within the primary motor cortex or in non-primary motor areas, in congenital lesions the specific phase of brain maturation allows for unique neuroplastic processes of sensorimotor reorganization. These are based on the existence, during the first weeks of life, of bilateral motor projections originating in the primary motor areas, which connect each hemisphere with both sides of the body. These tracts generally withdraw during development, but they can persist in case of cerebral damage, giving rise to a contralesional reorganization of motor function, exclusive of early brain damage [5].

We propose a provocative hypothesis arguing that the Action Observation therapy might be effective in very early intervention in infants with unilateral or asymmetric brain damage, but through a different underlying mechanism. If the activation of motor networks induced in infancy by action observation enhances the excitability of the damaged sensorimotor cortex, it could also accelerate the maturation of the corticospinal tract and the adaptive shaping of the spinal motor circuits. This hypothesis should be explored carefully in prospective studies and, if confirmed, might support the use of action observation therapy at a much earlier time than experimented so far.

**References**

1. Jeannerod M. Neural simulation of action: a unifying mechanism for motor cognition. Neuroimage. 2001 Jul;14(1 Pt 2):S103-9.

2. Molenberghs P, Cunnington R, Mattingley JB. Brain regions with mirror properties: a meta-analysis of 125 human fMRI studies. Neurosci Biobehav Rev. 2012 Jan;36(1):341-9.

3. Meltzoff AN, Kuhl PK, Movellan J, Sejnowski TJ. Foundations for a new science of learning. Science. 2009 Jul 17;325(5938):284-8.

4. Southgate V, Johnson MH, El Karoui I, Csibra G. Motor system activation reveals infants' on-line prediction of others' goals. Psychol Sci. 2010 Mar;21(3):355-9.

5. Eyre JA. Corticospinal tract development and its plasticity after perinatal injury. Neurosci Biobehav Rev. 2007;31(8):1136-49.

## S4 Resistance training and muscle hypertrophy: new research insights

### Richard S. Metcalfe (r.s.metcalfe@swansea.ac.uk)

#### Applied Sports Technology, Exercise and Medicine (A-STEM) Research Centre, Swansea University, Swansea, Wales, UK, SA1 8EN

Our understanding of the modifying effects of different resistance training parameters on gains in skeletal muscle hypertrophy and strength has increased substantially over the last 5-10 years. In particular, numerous research studies have now demonstrated that gains in muscle hypertrophy and strength following resistance training are independent of the load lifted, provided that the load is lifted until the point of momentary muscular failure [1-3]. The first study to provide evidence for this idea came from Burd et al. [1], who demonstrated that acute post-exercise increases in mixed muscle protein synthesis were no different when lifting loads of 30% of 1 repetition max (1RM) compared to 90% of 1RM, so long as both loads were taken to failure. Of course, acute changes in muscle protein synthesis do not necessarily equate to subsequent changes in muscle mass and strength with training, but this proof of concept study was followed up with a large and comprehensive 12-week training study in resistance trained participants [2]. This study was able to demonstrate no differences in the increases in fat free mass, as well as type 1 and type 2 muscle fibre cross sectional area, in groups performing 30% or 90% of 1RM to failure as their training stimulus [2]. They also observed no differences in the change in muscle strength. Both of these findings were neatly replicated by Schoenfeld et al. [3] who conducted a meta-analysis of all resistance training studies looking at low (<60% 1RM) and high (>60% 1RM) loads to failure in both trained and untrained participants. Taken together, these studies suggest that lifting heavy loads is sufficient, but not necessary, to achieve gains in muscle mass and strength with resistance training. In summary, for changes in muscle mass and muscle strength, practitioners can select a load that best suits their patient/client and be confident that, if lifted to failure, the benefits will be largely similar. Lifting weights to failure, regardless of load, may not be possible or desirable for many individuals (e.g. in rehabilitation settings). Interestingly, research over the last few years has also shown that, by applying some partial occlusion of blood flow to working muscles during aerobic or resistance exercise (‘blood flow restriction’ training), it may be possible to achieve gains in muscle mass and strength with light loads even when not lifted to failure [4,5]. In fact, blood flow restriction may have applications across the rehabilitation spectrum [5]. For example, Takarada et al. [6] demonstrated that intermittent blood flow restriction attenuated the loss of muscle cross sectional area during 14 days of unloading, whilst Abe et al. [7] found that blood flow restriction applied during walking promoted increases in muscle CSA compared with no changes with walking alone. Finally, a recent meta-analysis demonstrated that blood flow restriction applied during low load resistance training increases muscle mass and strength to a greater extent than low load training alone [4]. Taken together, this research suggests that lighter loads with blood flow restriction could be effective stimulus to apply in rehabilitation settings but more research is required in this area [5].

**References**

1. Burd NA, West DW, Staples AW, Atherton PJ, Baker JM, Moore DR, Holwerda AM, Parise G, Rennie MJ, Baker SK, Phillips SM. Low-load high volume resistance exercise stimulates muscle protein synthesis more than high-load low volume resistance exercise in young men. PLoS One. 2010 Aug 9;5(8):e12033.

2. Morton RW, Oikawa SY, Wavell CG, Mazara N, McGlory C, Quadrilatero J, Baechler BL, Baker SK, Phillips SM. Neither load nor systemic hormones determine resistance training-mediated hypertrophy or strength gains in resistance-trained young men. J Appl Physiol (1985). 2016 Jul 1;121(1):129-38.

3. Schoenfeld BJ, Grgic J, Ogborn D, Krieger JW. Strength and Hypertrophy Adaptations Between Low- vs. High-Load Resistance Training: A Systematic Review and Meta-analysis. J Strength Cond Res. 2017 Dec;31(12):3508-3523.

4. Hughes L, Paton B, Rosenblatt B, Gissane C, Patterson SD. Blood flow restriction training in clinical musculoskeletal rehabilitation: a systematic review and meta-analysis. Br J Sports Med. 2017 Jul;51(13):1003-1011.

5. Patterson SD, Hughes L, Head P, Warmington S, Brandner C. Blood flow restriction training: a novel approach to augment clinical rehabilitation: how to do it. Br J Sports Med. 2017 Dec;51(23):1648-1649.

6. Takarada Y, Takazawa H, Ishii N. Applications of vascular occlusion diminish disuse atrophy of knee extensor muscles. Med Sci Sports Exerc. 2000 Dec;32(12):2035-9.

7. Abe T, Kearns CF, Sato Y. Muscle size and strength are increased following walk training with restricted venous blood flow from the leg muscle, Kaatsu-walk training. J Appl Physiol (1985). 2006 May;100(5):1460-6.

## S5 The effects of exercise on muscle strength, body composition, physical functioning and the inflammatory profile of older adults

### Keliane Liberman^1,2^, Rose Njemini^1,2^, Ivan Bautmans^1,2,3^

#### ^1^Gerontology department, Vrije Universiteit Brussel, Laarbeeklaan 103, B-1090 Brussels, Belgium; ^2^Frailty in Ageing research department, Vrije Universiteit Brussel, Laarbeeklaan 103, B-1090 Brussels, Belgium; ^3^Geriatrics department, Universitair Ziekenhuis Brussel, Laarbeeklaan 101, B-1090 Brussels, Belgium

##### **Correspondence:** Ivan Bautmans (Ivan.Bautmans@vub.be)

Sarcopenia, defined by the loss of muscle mass and muscle strength, is a typical characteristic of ageing. From the age of 65 years, a loss of muscle strength of approximately 2% is seen per year [1]. Exercise is one the most efficient ways to counter sarcopenia in older adults through several mechanisms. Training induces neuromuscular adaptations in older adults, increasing voluntary activation and leading to gains in muscle strength [2]. Second, at least 10 weeks of resistance training leads to muscle hypertrophy in older adults [3]. A third mechanism is through the adaptations in the inflammatory profile. On short term, the contracting muscle activates a myokine response through the secretion of IL-6 by the muscle, triggering the anti-inflammatory response in circulating immune cells and returning to baseline values after 24 hours. On longer term, these effects will accumulate and reverse the inflammatory profile towards an anti-inflammatory profile [4].

Although there is evidence that resistance training has positive effects on muscle strength, physical functioning and the inflammatory profile of older adults, it has not been investigated thoroughly in frail older adults [5]. In frail older adults, NSAIDs are often prescribed to counter the inflammation. Unfortunately, given the numerous side effects, NSAID treatment is rarely possible and no effective interventions exist today to counter inflammation-induced weakness in those patients.

Currently, there is no consensus yet on the training modality with most favorable effects for older adults and exercise is not often prescribed to counter sarcopenia. The Frailty in Ageing (FRIA) research department of the Vrije Universiteit Brussel has been researching the dose-response relationship of resistance training in older adults. Forti et al. [6] showed that compared to a control group with no resistance exercise, high resistance exercise lead to decreases of IL-6 after 12 weeks. Similar results were obtained in an ongoing study of the department, where intensive resistance training lead to decreases in IL-6 compared to a control or to endurance strength training (lower resistance but higher number of repetitions). Mangine et al. [7] showed that training intensity rather than training volume leads to higher muscle strength gains. Another third study resulted in increases in anti-inflammatory cytokines after 12 weeks of high resistance training compared to lower exercise intensities [8].

From past and current studies, we can conclude that resistance exercise is an effective manner to counter sarcopenia when performed with sufficient high exercise volume and intensity. However, the priority should be set at implementing training interventions in frail older adults to counter sarcopenia.

**References**

1. Frontera WR, Hughes VA, Fielding RA, Fiatarone MA, Evans WJ, Roubenoff R. Aging of skeletal muscle: a 12-yr longitudinal study. J Appl Physiol (1985). 2000 Apr;88(4):1321-6.

2. Arnold P, Bautmans I. The influence of strength training on muscle activation in elderly persons: a systematic review and meta-analysis. Exp Gerontol. 2014 Oct;58:58-68.

3. Narici MV, Reeves ND, Morse CI, Maganaris CN. Muscular adaptations to resistance exercise in the elderly. J Musculoskelet Neuronal Interact. 2004 Jun;4(2):161-4.

4. Forti LN, Van Roie E, Njemini R, Coudyzer W, Beyer I, Delecluse C, Bautmans I. Effects of resistance training at different loads on inflammatory markers in young adults. Eur J Appl Physiol. 2017 Mar;117(3):511-519.

5. Liberman K, Forti LN, Beyer I, Bautmans I. The effects of exercise on muscle strength, body composition, physical functioning and the inflammatory profile of older adults: a systematic review. Curr Opin Clin Nutr Metab Care. 2017 Jan;20(1):30-53.

6. Forti LN, Njemini R, Beyer I, Eelbode E, Meeusen R, Mets T, Bautmans I. Strength training reduces circulating interleukin-6 but not brain-derived neurotrophic factor in community-dwelling elderly individuals. Age (Dordr). 2014;36(5):9704. doi: 10.1007/s11357-014-9704-6.

7. Mangine GT, Hoffman JR, Gonzalez AM, Townsend JR, Wells AJ, Jajtner AR, Beyer KS, Boone CH, Miramonti AA, Wang R, LaMonica MB, Fukuda DH, Ratamess NA, Stout JR. The effect of training volume and intensity on improvements in muscular strength and size in resistance-trained men. Physiol Rep. 2015 Aug;3(8). pii: e12472.

8. Forti LN, Van Roie E, Njemini R, Coudyzer W, Beyer I, Delecluse C, Bautmans I. Load-Specific Inflammation Mediating Effects of Resistance Training in Older Persons. J Am Med Dir Assoc. 2016 Jun 1;17(6):547-52.

## S6 New training strategies in cardiac and pulmonary rehabilitation

### Mara Paneroni (mara.paneroni@gmail.com)

#### Respiratory Department, ICS Maugeri, Lumezzane (BS), Italy

A large literature over the last 20 years have described the exercise intolerance problem of Chronic Obstructive Pulmonary Disease (COPD) and Heart Failure (HF) that leads to disability and reduction of quality of life in these patients population. One of the principal causes of exercise intolerance described are skeletal muscle dysfunctions due to anabolic and metabolic abnormalities with a reduction of strength and an early development of fatigue [1]. A rehabilitation approach including training has been largely documented to be able to improve performance and impact on disability and quality of life, so that it has been included in a principal guidelines wrote by the world's leading scientific societies [2,3]. Current training directions have to include protocol of endurance-training (grade A of evidence) which consist of cycle-ergometer or treadmill training performed in continuous or interval training way with a predefined intensity and duration ranges. Additional interventions are resistive training protocol in supported and unsupported way [2,3].

In the last years, studies are studying new strategies to improve effort tolerance and ability to sustained training workload in cardio-respiratory patients. Research area can be divided in studies aiming to 1) find the best endurance training program, 2) find new training techniques, 3) find external aids during training, and 4) find new effective complementary treatments.

In the endurance training area, studies are evaluating the possibility to apply periodization protocol of training in this population, similarly of athletes’ protocols. Periodization is planned long-term variation of the volume and intensity of training to prevent overtraining and promote optimal performance at the desired time. The different neuromuscular adaptations of the system can be reached within the same training phase, but not within the same session. Some kind of periodization has been tested with good results in COPD patients [4].

About the new training techniques proposed there are many studies analyzing the possibility to use funny and effective alternatives of endurance training performed by cycle-ergometer and treadmill with the aim to improve compliance. Examples are Thai-chi [5], Yoga [6], Nordic-walking, dancing [7] or water-based training. When performed at right training intensity, they are able to produce similar improvement compared to classical cycle-ergometer or treadmill endurance training.

Other studies are evaluating external aids during training with the aim to reduce dyspnea and respiratory workload and therefore improve the ability to sustain training session. In this field, oxygen supplementation and mechanical ventilation [8] can improve effort-reducing load applied on the respiratory system, whereas Functional Electrical Stimulation (FES) can improve muscle contraction and reduce fatigue during training session.

Lastly, a new main research area is the definition of the best complementary treatment for example the diet supplementation [9] defining the better dose to reach high response.

In conclusion, exercise training is an essential (1 A level of evidence) treatment in COPD and HF patients. New research perspectives in this field are defining the best dose of training, new good techniques and external and additional training aids. Researches have also to define the Responders/ non-Responders phenotypes.

**References**

1. Gosker HR, Lencer NH, Franssen FM, van der Vusse GJ, Wouters EF, Schols AM. Striking similarities in systemic factors contributing to decreased exercise capacity in patients with severe chronic heart failure or COPD. Chest. 2003 May;123(5):1416-24.

2. Rochester CL, Vogiatzis I, Holland AE, Lareau SC, Marciniuk DD, Puhan MA, Spruit MA, Masefield S, Casaburi R, Clini EM, Crouch R, Garcia-Aymerich J, Garvey C, Goldstein RS, Hill K, Morgan M, Nici L, Pitta F, Ries AL, Singh SJ, Troosters T, Wijkstra PJ, Yawn BP, ZuWallack RL; ATS/ERS Task Force on Policy in Pulmonary Rehabilitation. An Official American Thoracic Society/European Respiratory Society Policy Statement: Enhancing Implementation, Use, and Delivery of Pulmonary Rehabilitation. Am J Respir Crit Care Med. 2015 Dec 1;192(11):1373-86.

3. Dickstein K, Cohen-Solal A, Filippatos G, McMurray JJ, Ponikowski P, Poole-Wilson PA, Strömberg A, van Veldhuisen DJ, Atar D, Hoes AW, Keren A, Mebazaa A, Nieminen M, Priori SG, Swedberg K; ESC Committee for Practice Guidelines (CPG). ESC guidelines for the diagnosis and treatment of acute and chronic heart failure 2008: the Task Force for the diagnosis and treatment of acute and chronic heart failure 2008 of the European Society of Cardiology. Developed in collaboration with the Heart Failure Association of the ESC (HFA) and endorsed by the European Society of Intensive Care Medicine (ESICM). Eur J Heart Fail. 2008 Oct;10(10):933-89.

4. Klijn P, van Keimpema A, Legemaat M, Gosselink R, van Stel H. Nonlinear exercise training in advanced chronic obstructive pulmonary disease is superior to traditional exercise training. A randomized trial. Am J Respir Crit Care Med. 2013 Jul 15;188(2):193-200.

5. Ngai SP, Jones AY, Tam WW. Tai Chi for chronic obstructive pulmonary disease (COPD). Cochrane Database Syst Rev. 2016 Jun 7;(6):CD009953.

6. Gomes-Neto M, Rodrigues ES Jr, Silva WM Jr, Carvalho VO. Effects of Yoga in Patients with Chronic Heart Failure: A Meta-Analysis. Arq Bras Cardiol. 2014 Nov;103(5):433-439.

7. Kaltsatou AC, Kouidi EI, Anifanti MA, Douka SI, Deligiannis AP. Functional and psychosocial effects of either a traditional dancing or a formal exercising training program in patients with chronic heart failure: a comparative randomized controlled study. Clin Rehabil. 2014 Feb;28(2):128-38.

8. Menadue C, Piper AJ, van 't Hul AJ, Wong KK. Non-invasive ventilation during exercise training for people with chronic obstructive pulmonary disease. Cochrane Database Syst Rev. 2014 May 14;(5):CD007714.

9. van de Bool C, Steiner MC, Schols AM. Nutritional targets to enhance exercise performance in chronic obstructive pulmonary disease. Curr Opin Clin Nutr Metab Care. 2012 Nov;15(6):553-60.

## S7 Exercise therapy for chronic pain: retraining mind and brain

### Jo Nijs^1,2,3^ (Jo.Nijs@vub.be)

#### ^1^Pain in Motion International Research Group, www.paininmotion.be; ^2^Department of Physiotherapy, Human Physiology and Anatomy, Faculty of Physical Education & Physiotherapy, Vrije Universiteit Brussel, Belgium; ^3^Department of Physical Medicine and Physiotherapy, University Hospital Brussels, Belgium

Chronic pain is the post prevalent and most costly medical problem in the Western society. It is now well-established that sensitization of the central nervous system is an important feature in many patients with chronic pain [1-4], but the etiological mechanisms of this central nervous system dysfunction are poorly understood. Central sensitization encompasses various related dysfunctions of the central nervous system, all contributing to an increased responsiveness to a variety of stimuli [5]. This lecture will cover two important etiological mechanisms together with their therapeutic implications: aberrant glial activity and development of pain memories.

Recently, an increasing number of animal and human studies suggest that aberrant glial activation takes part in the establishment and/or maintenance of central sensitization [6-8]. Such glial overactivation results in a low-grade neuroinflammatory state, characterized by high levels of BDNF9, IL-1β, TNF-α, which in turn increases the excitability of the central nervous system neurons through mechanisms like long-term potentiation and increased synaptic efficiency [9,10]. Aberrant glial activity in chronic pain might have been triggered by severe stress exposure, and/or sleeping disturbances [10], each of which are established initiating factors for chronic pain development. Potential treatment avenues include several pharmacological options for diminishing glial activity, as well as conservative interventions like sleep management, stress management and exercise therapy.

The second potential etiological mechanism entails the development of pain memories. Even though nociceptive pathology has often long subsided, the brain of patients with chronic pain has typically acquired a protective (movement-related) pain memory [11,12]. Exercise therapy for patients with chronic pain is often hampered by such pain memories. Therapists can alter pain memories [13] in patients with chronic pain by integrating pain neuroscience education with exercise interventions [14]. The latter includes applying graded exposure in vivo principles during exercise therapy, for targeting the brain circuitries orchestrated by the amygdala (the memory of fear centre in the brain) [15,16].

**References**

1. Nijs J, Ickmans K. Chronic whiplash-associated disorders: to exercise or not? Lancet. 2014 Jul 12;384(9938):109-11.

2. Nijs J, Torres-Cueco R, van Wilgen CP, Girbes EL, Struyf F, Roussel N, van Oosterwijck J, Daenen L, Kuppens K, Vanwerweeen L, Hermans L, Beckwee D, Voogt L, Clark J, Moloney N, Meeus M. Applying modern pain neuroscience in clinical practice: criteria for the classification of central sensitization pain. Pain Physician. 2014 Sep-Oct;17(5):447-57.

3. Nijs J, Apeldoorn A, Hallegraeff H, Clark J, Smeets R, Malfliet A, Girbes EL, De Kooning M, Ickmans K. Low back pain: guidelines for the clinical classification of predominant neuropathic, nociceptive, or central sensitization pain. Pain Physician. 2015 May-Jun;18(3):E333-46.

4. Roussel NA, Nijs J, Meeus M, Mylius V, Fayt C, Oostendorp R. Central sensitization and altered central pain processing in chronic low back pain: fact or myth? Clin J Pain. 2013 Jul;29(7):625-38.

5. Nijs J, Van Houdenhove B, Oostendorp RA. Recognition of central sensitization in patients with musculoskeletal pain: Application of pain neurophysiology in manual therapy practice. Man Ther. 2010 Apr;15(2):135-41.

6. Albrecht DS, Granziera C, Hooker JM, Loggia ML. In Vivo Imaging of Human Neuroinflammation. ACS Chem Neurosci. 2016 Apr 20;7(4):470-83.

7. Loggia ML, Chonde DB, Akeju O, Arabasz G, Catana C, Edwards RR, Hill E, Hsu S, Izquierdo-Garcia D, Ji RR, Riley M, Wasan AD, Zürcher NR, Albrecht DS, Vangel MG, Rosen BR, Napadow V, Hooker JM. Evidence for brain glial activation in chronic pain patients. Brain. 2015 Mar;138(Pt 3):604-15.

8. Ji RR, Berta T, Nedergaard M. Glia and pain: is chronic pain a gliopathy? Pain. 2013 Dec;154 Suppl 1:S10-28.

9. Nijs J, Meeus M, Versijpt J, Moens M, Bos I, Knaepen K, Meeusen R. Brain-derived neurotrophic factor as a driving force behind neuroplasticity in neuropathic and central sensitization pain: a new therapeutic target? Expert Opin Ther Targets. 2015 Apr;19(4):565-76.

10. Nijs J, Loggia ML, Polli A, Moens M, Huysmans E, Goudman L, Meeus M, Vanderweeën L, Ickmans K, Clauw D. Sleep disturbances and severe stress as glial activators: key targets for treating central sensitization in chronic pain patients? Expert Opin Ther Targets. 2017 Aug;21(8):817-826.

11. Zusman M. Forebrain-mediated sensitization of central pain pathways: 'non-specific' pain and a new image for MT. Man Ther. 2002 May;7(2):80-8.

12. Zusman M. Mechanisms of musculoskeletal physiotherapy. Physical Therapy Reviews 2004; 9: 39-49.

13. Zusman M. Associative memory for movement-evoked chronic back pain and its extinction with musculoskeletal physiotherapy. Physical Therapy Reviews 2008; 13(1): 57-68.

14. Nijs J, Lluch Girbés E, Lundberg M, Malfliet A, Sterling M. Exercise therapy for chronic musculoskeletal pain: Innovation by altering pain memories. Man Ther. 2015 Feb;20(1):216-20.

15. Malfliet A, Kregel J, Coppieters I, De Pauw R, Meeus M, Roussel N, Cagnie B, Danneels L, Nijs J. Effect of Pain Neuroscience Education Combined With Cognition-Targeted Motor Control Training on Chronic Spinal Pain: A Randomized Clinical Trial. JAMA Neurol. 2018 Apr 16.

16. Malfliet A, Kregel J, Meeus M, Cagnie B, Roussel N, Dolphens M, Danneels L, Nijs J. Applying contemporary neuroscience in exercise interventions for chronic spinal pain: treatment protocol. Braz J Phys Ther. 2017 Sep - Oct;21(5):378-387.

## S8 Mechanism-based differential diagnosis of neuropathic, nociceptive and central sensitization pain in clinical practice

### Jo Nijs^1,2,3^ (Jo.Nijs@vub.be)

#### ^1^Pain in Motion International Research Group, www.paininmotion.be; ^2^Department of Physiotherapy, Human Physiology and Anatomy, Faculty of Physical Education & Physiotherapy, Vrije Universiteit Brussel, Belgium; ^3^Department of Physical Medicine and Physiotherapy, University Hospital Brussels, Belgium

Broadly, four pain classifications are widely considered: nociceptive (inflammatory) pain, neuropathic pain, central sensitization (CS) pain and mixed pain. To aid clinicians, a clinical method for classifying any pain as either predominant CS, neuropathic or nociceptive pain was developed, based on a large body of research evidence and international expert opinion [1].

The first step comprises screening for neuropathic pain. Guidelines for the classification of neuropathic pain are available [2]. In cases without neuropathic pain or with a mixed type of pain, screening for nociceptive and CS pain is the next step. To differentiate predominant nociceptive and CS pain, clinicians are advised to use the algorithm guiding them through the screening of three major classification criteria:

Criterion 1: Pain experience disproportionate to the nature and extent of injury or pathology [1]. Per definition, CS pain is disproportionate to the nature and extent of injury or pathology, making it a go- or no-go criterion for CS pain. Criterion 2: Neuro-anatomically illogical pain pattern [1]. A neuro-anatomically illogical pain pattern is present when the patients presents with a pain distribution that is not neuroanatomically plausible for the presumed source(s) of nociception [1]. Criterion 3: Hypersensitivity of senses unrelated to the musculoskeletal system [1]. For assessing sensory hypersensitivity the Central Sensitization Inventory [3] can be used. Several studies support the clinimetric properties of the Central Sensitization Inventory in different countries [3-6]. The cut-off of 40/100 allows correct identification of over 82% of CS pain patients [7], but the chances of false positives are relatively high, which supports our approach of combining this measure with a more comprehensive examination for identification of predominant CS pain.

Since the initial publication of the classification criteria for musculoskeletal pain in general, they have been adopted to better fit the specific needs for the clinical classification of pain types in people with low back pain [8], osteoarthritis [9] and pain following cancer treatment [10].

**References**

1. Nijs J, Torres-Cueco R, van Wilgen CP, Girbes EL, Struyf F, Roussel N, van Oosterwijck J, Daenen L, Kuppens K, Vanwerweeen L, Hermans L, Beckwee D, Voogt L, Clark J, Moloney N, Meeus M. Applying modern pain neuroscience in clinical practice: criteria for the classification of central sensitization pain. Pain Physician. 2014 Sep-Oct;17(5):447-57.

2. Treede RD, Jensen TS, Campbell JN, Cruccu G, Dostrovsky JO, Griffin JW, Hansson P, Hughes R, Nurmikko T, Serra J. Neuropathic pain: redefinition and a grading system for clinical and research purposes. Neurology. 2008 Apr 29;70(18):1630-5.

3. Mayer TG, Neblett R, Cohen H, Howard KJ, Choi YH, Williams MJ, Perez Y, Gatchel RJ. The development and psychometric validation of the central sensitization inventory. Pain Pract. 2012 Apr;12(4):276-85.

4. Neblett R, Cohen H, Choi Y, Hartzell MM, Williams M, Mayer TG, Gatchel RJ. The Central Sensitization Inventory (CSI): establishing clinically significant values for identifying central sensitivity syndromes in an outpatient chronic pain sample. J Pain. 2013 May;14(5):438-45.

5. Kregel J, Vuijk PJ, Descheemaeker F, Keizer D, van der Noord R, Nijs J, Cagnie B, Meeus M, van Wilgen P. The Dutch Central Sensitization Inventory (CSI): Factor Analysis, Discriminative Power, and Test-Retest Reliability. Clin J Pain. 2016 Jul;32(7):624-30.

6. Scerbo T, Colasurdo J, Dunn S, Unger J, Nijs J, Cook C. Measurement Properties of the Central Sensitization Inventory: A Systematic Review. Pain Pract. 2018 Apr;18(4):544-554.

7. Cuesta-Vargas AI, Neblett R, Chiarotto A, Kregel J, Nijs J, van Wilgen CP, Pitance L, Knezevic A, Gatchel RJ, Mayer TG, Viti C, Roldan-Jiménez C, Testa M, Caumo W, Jeremic-Knezevic M, Luciano JV. Dimensionality and Reliability of the Central Sensitization Inventory in a Pooled Multicountry Sample. J Pain. 2018 Mar;19(3):317-329.

8. Nijs J, Apeldoorn A, Hallegraeff H, Clark J, Smeets R, Malfliet A, Girbes EL, De Kooning M, Ickmans K. Low back pain: guidelines for the clinical classification of predominant neuropathic, nociceptive, or central sensitization pain. Pain Physician. 2015 May-Jun;18(3):E333-46.

9. Lluch E, Nijs J, Courtney CA, Rebbeck T, Wylde V, Baert I, Wideman TH, Howells N, Skou ST. Clinical descriptors for the recognition of central sensitization pain in patients with knee osteoarthritis. Disabil Rehabil. 2017 Aug 2:1-10.

10. Nijs J, Leysen L, Adriaenssens N, Aguilar Ferrándiz ME, Devoogdt N, Tassenoy A, Ickmans K, Goubert D, van Wilgen CP, Wijma AJ, Kuppens K, Hoelen W, Hoelen A, Moloney N, Meeus M. Pain following cancer treatment: Guidelines for the clinical classification of predominant neuropathic, nociceptive and central sensitization pain. Acta Oncol. 2016 Jun;55(6):659-63.

## S9 Adaptations to high-intensity interval training compared with moderate intensity continuous training

### Richard S. Metcalfe (r.s.metcalfe@swansea.ac.uk)

#### Applied Sports Technology, Exercise and Medicine (A-STEM) Research Centre, Swansea University, Swansea, Wales, UK, SA1 8EN

Over the last 15 years there has been increased research interest in the effects of high intensity interval training (HIIT) on human health. HIIT can be defined as periods of relatively intense or maximal exercise interspersed with periods of low intensity or resting recovery. As such, HIIT as an exercise stimulus is almost infinitely variable [1], but two clear themes have emerged from recent HIIT research. Firstly, in comparison to traditional moderate intensity continuous training (MICT), if a matched dose of HIIT (e.g. energy expenditure matched) is employed, then research suggests that HIIT elicits superior physiological adaptations [2-4]. In other words, the intensity of exercise is a key determinant of training stimulus. For example, MacInnis et al [4] employed a within subjects single-legged training study design, where participants simultaneously trained one leg with MICT (30-min at 50% Wmax) and one leg with energy expenditure and time matched HIIT (4 x 5 at 65% Wmax). They demonstrated that skeletal muscle mitochondrial enzyme activity and O_2_ flux were consistently improved to a greater extent in the HIIT trained leg [4]. Other meta-analyses have concluded that HIIT is also associated with superior improvements in maximal aerobic capacity (VO2max) [3] and insulin sensitivity [2].

The second theme to emerge concerns a specific form of HIIT which involves ‘all-out’ or supramaximal intensity efforts, also known as Sprint Interval Training (SIT). Studies have demonstrated that cycling based SIT produces similar physiological adaptations to MICT but with a substantially lower exercise volume and time commitment [5]. For example, one study compared 12 weeks of SIT (10-minute time commitment; 3 x 20-second sprints) with 12 weeks of MICT in a group of sedentary young men. They showed that SIT elicited similar improvements in VO_2_max, insulin sensitivity and mitochondrial density compared with MICT, despite SIT involving a five-fold lower exercise volume and time commitment [6]. More recent studies from our research group have examined in detail the effect of different training parameters on the changes in VO2max with SIT [7-9]. For example, our meta-analysis demonstrated that reducing the number of sprints in a SIT session does not attenuate (and may even enhance) the improvement in VO_2_max observed with several weeks of SIT [7]. In fact, the lowest number of sprints that remains effective for improving VO_2_max is just 2 [7]. We have also recently demonstrated that, when the number of sprint repetitions completed per session is low (2 per session), decreasing the duration of the sprints from 20-s to 10-s reduces the improvement in VO_2_max observed with training by around 50% [8]. The practical implications of these findings is that an exercise session which is effective for improving important health markers can be completed in as little as 10-minutes and is generally well tolerated, with previously sedentary participants rating sessions as ‘somewhat hard’ [8,10].

In summary, the nature of the adaptations observed with HIIT is similar to those observed with moderate intensity continuous exercise. HIIT protocols can be designed in a way that elicits superior physiological (and health related) adaptations compared with MICT, but these protocols still require high exercise volume and time-commitment. On the other hand, HIIT can also be designed in a way that elicits similar physiological (and health related) adaptations compared with MICT, but in a very time and dose-efficient manner.

**References**

1. Buchheit M, Laursen PB. High-intensity interval training, solutions to the programming puzzle: Part I: cardiopulmonary emphasis. Sports Med. 2013 May;43(5):313-38.

2. Jelleyman C, Yates T, O'Donovan G, Gray LJ, King JA, Khunti K, Davies MJ. The effects of high-intensity interval training on glucose regulation and insulin resistance: a meta-analysis. Obes Rev. 2015 Nov;16(11):942-61.

3. Milanović Z, Sporiš G, Weston M. Effectiveness of High-Intensity Interval Training (HIT) and Continuous Endurance Training for VO2max Improvements: A Systematic Review and Meta-Analysis of Controlled Trials. Sports Med. 2015 Oct;45(10):1469-81.

4. MacInnis MJ, Zacharewicz E, Martin BJ, Haikalis ME, Skelly LE, Tarnopolsky MA, Murphy RM, Gibala MJ. Superior mitochondrial adaptations in human skeletal muscle after interval compared to continuous single-leg cycling matched for total work. J Physiol. 2017 May 1;595(9):2955-2968.

5. Vollaard NBJ, Metcalfe RS. Research into the Health Benefits of Sprint Interval Training Should Focus on Protocols with Fewer and Shorter Sprints. Sports Med. 2017 Dec;47(12):2443-2451.

6. Gillen JB, Martin BJ, MacInnis MJ, Skelly LE, Tarnopolsky MA, Gibala MJ. Twelve Weeks of Sprint Interval Training Improves Indices of Cardiometabolic Health Similar to Traditional Endurance Training despite a Five-Fold Lower Exercise Volume and Time Commitment. PLoS One. 2016 Apr 26;11(4):e0154075.

7. Vollaard NBJ, Metcalfe RS, Williams S. Effect of Number of Sprints in an SIT Session on Change in V˙O2max: A Meta-analysis. Med Sci Sports Exerc. 2017 Jun;49(6):1147-1156.

8. Nalçakan GR, Songsorn P, Fitzpatrick BL, Yüzbasioglu Y, Brick NE, Metcalfe RS, Vollaard NBJ. Decreasing sprint duration from 20 to 10 s during reduced-exertion high-intensity interval training (REHIT) attenuates the increase in maximal aerobic capacity but has no effect on affective and perceptual responses. Appl Physiol Nutr Metab. 2018 Apr;43(4):338-344.

9. Metcalfe RS, Tardif N, Thompson D, Vollaard NB. Changes in aerobic capacity and glycaemic control in response to reduced-exertion high-intensity interval training (REHIT) are not different between sedentary men and women. Appl Physiol Nutr Metab. 2016 Nov;41(11):1117-1123.

10. Metcalfe RS, Babraj JA, Fawkner SG, Vollaard NB. Towards the minimal amount of exercise for improving metabolic health: beneficial effects of reduced-exertion high-intensity interval training. Eur J Appl Physiol. 2012 Jul;112(7):2767-75.

## S10 Shoulder revolution: beyond a structural perspective

### Diego Ristori (ristori.d@gmail.com)

#### Department of Neuroscience, Rehabilitation, Ophtalmology, Genetics, Maternal and Child Health – University of Genova – Campus of Savona, Italy

Shoulder pain (SP) represent a very common musculoskeletal condition that require physical therapy care [1]. Over the years, the usual evaluation strategy based on clinicical tests and diagnostic imaging has been challenged [2]. Clinical tests seem to be unable to identify the scructures which creates patient’s pain and the interpretation of diagnostic imaging is still controversial [3]. The resulting patho-anatomical diagnostic categories have demonstrate poor releability and seems to be inadequate to guide the treatment [4].

We will present the different alternative proposals existing in the literature [5-7] and integrate it in a single model, in order to provide clinicians with a helpful tool to deal with SP patients.

Our proposal would represent a pragmatic approach for SP patients. Our goal is to orientate the evaluation and treatment of this kind of patients toward a bio-psico social model. We hope that in the future the category of non-specific shoulder pain should be taken into account in diagnostic and prognostic studies.

**References**

1. Kuijpers T, van Tulder MW, van der Heijden GJ, Bouter LM, van der Windt DA. Costs of shoulder pain in primary care consulters: a prospective cohort study in The Netherlands. BMC Musculoskelet Disord. 2006 Nov 1;7:83.

2. Hegedus EJ, Goode AP, Cook CE, Michener L, Myer CA, Myer DM, Wright AA. Which physical examination tests provide clinicians with the most value when examining the shoulder? Update of a systematic review with meta-analysis of individual tests. Br J Sports Med. 2012 Nov;46(14):964-78.

3. Lewis JS. Rotator cuff tendinopathy/subacromial impingement syndrome: is it time for a new method of assessment? Br J Sports Med. 2009 Apr;43(4):259-64.

4. Hanchard NC, Lenza M, Handoll HH, Takwoingi Y. Physical tests for shoulder impingements and local lesions of bursa, tendon or labrum that may accompany impingement. Cochrane Database Syst Rev. 2013 Apr 30;(4):CD007427.

5. Schellingerhout JM, Verhagen AP, Thomas S, Koes BW. Lack of uniformity in diagnostic labeling of shoulder pain: time for a different approach. Man Ther. 2008 Dec;13(6):478-83.

6. Klintberg IH, Cools AM, Holmgren TM, Holzhausen AC, Johansson K, Maenhout AG, Moser JS, Spunton V, Ginn K. Consensus for physiotherapy for shoulder pain. Int Orthop. 2015 Apr;39(4):715-20.

7. McClure PW, Michener LA. Staged Approach for Rehabilitation Classification: Shoulder Disorders (STAR-Shoulder). Phys Ther. 2015 May;95(5):791-800.

## S11 Physical therapies in physioterapy

### Matteo Benedini (matteo@fisiocentermultimedica.com)

#### Presidente Nazionale Gruppo Interesse Specialistico Terapie Fisiche e Tecnologie Riabilitative (GIS TFTR AIFI), Presidente International Association Laser Therapy (IALT), Consigliere Associazione Italiana Medici del Volley - Lega Volley Serie A, Ricercatore Società Italiana Biofisica Elettrodinamica (SIBE), Associate of International Society of Proprioception and Posture (ISPP), Certificazione Internazionale Onde d’Urto (ISMST), Master in Osteopatia, Consulente Fisioterapista Atleti Nazionale Canoa Kayak, Consulente Fisioterapista Atleti Squadra Nazionale Sci Alpino, Fisiocenter Multimedica, Trauma Center and Sport Medicine Institute, Biophysic, Metabolic, Proprioception and Posture Lab, Sport Specific Rehabilitation and Performance Enhancement, Bagnolo San Vito – Mantova

Physical therapies are an important tool for the treatment of the most frequent acute and chronic pathologies of physiotherapeutic interest, involving both the muscle-tendon and osteoarticular areas.The most important therapies supported by scientific evidence are undoubtedly laser therapy [1,2] shock waves (Eswt) [3,4], radiofrequency, and extremely low frequency and intensity electromagnetic fields (ELF-EMF) [5-7]. The latter have undergone significant development in recent years, thanks to their ease of application and almost total safety. One of the most accredited phenomena used to explain the biological effects of ELF-EMF is the Ion Cyclotron Resonance-like (ICR-like) effect [8-10].

In this study an Italian made SEQEX device was used. SEQEX exploits the ICR-like phenomenon by delivering packets of complex frequencies while maintaining the intensity level of the field low. There is also the possibility of testing individual patients in order to establish which packets they respond best to, and customize their treatment.

The most widely studied effects of ELF-EMF on biological systems concern 1) the reduction of oxidative stress; 2) the modulation of inflammation; and 3) the improvement of microcirculation.

Based on these known effects, a conservative approach with ELF-EMF was applied in a case of non-traumatic avascular necrosis of the femoral head (AVN), 1^st^ degree according to Ficat classification. The various methods recommended for treatment of AVN in order to preserve the femoral head, including vascularized/non-vascularized bone grafting and the decompression of the nucleus, have produced inconsistent clinical outcomes. In this case study the patient was treated only with ICR-like SEQEX therapy in the following way: 1) non-focused total body treatment using an ELF-EMF radiant mat; 2) focused treatment with an accessory called a “Pro Pad” on the area involved, using the same electromagnetic fields as the non-focused treatment. The frequency of treatment was 5 times per week for a total of 4 months and the effect of the therapy on the AVN was measured by MRI. After 2 months of treatment, MRI investigation revealed a marked improvement. The 4-month MRI indicated total resolution of the problem, with complete relief from pain and recovery of motion. These results are consistent with previous findings in scientific literature regarding the use of electromagnetic fields.

**References**

1. Bjordal JM, Couppé C, Chow RT, Tunér J, Ljunggren EA. A systematic review of low level laser therapy with location-specific doses for pain from chronic joint disorders. Aust J Physiother. 2003;49(2):107-16.

2. Kadhim-Saleh A, Maganti H, Ghert M, Singh S, Farrokhyar F. Is low-level laser therapy in relieving neck pain effective? Systematic review and meta-analysis. Rheumatol Int. 2013 Oct;33(10):2493-501.

3. Wang CJ. Extracorporeal shockwave therapy in musculoskeletal disorders. J Orthop Surg Res. 2012 Mar 20;7:11. doi: 10.1186/1749-799X-7-11.

4. Chung B, Wiley JP. Extracorporeal shockwave therapy: a review. Sports Med. 2002;32(13):851-65.

5. Leon-Salas WD, Rizk H, Mo C, Weisleder N, Brotto L, Abreu E, Brotto M. A dual mode pulsed electro-magnetic cell stimulator produces acceleration of myogenic differentiation. Recent Pat Biotechnol. 2013 Apr;7(1):71-81.

6. Bao X, Shi Y, Huo X, Song T. A possible involvement of beta-endorphin, substance P, and serotonin in rat analgesia induced by extremely low frequency magnetic field. Bioelectromagnetics. 2006 Sep;27(6):467-72.

7. Riva Sanseverino E, Vannini A, Castellacci P. Therapeutic effects of pulsed magnetic fields on joint diseases. Panminerva Med. 1992 Oct-Dec;34(4):187-96.

8. Liboff AR. Geomagnetic cyclotron resonance in living things. J Biol Phys.. 1985;13(4):99-102

9. Liboff AR. A role for the geomagnetic field in cell regulation. Electromagn Biol Med. 2010 Aug;29(3):105-12.

10. Vincze G, Szasz A, Liboff AR. New theoretical treatment of ion resonance phenomena. Bioelectromagnetics. 2008 Jul;29(5):380-6.

## S12 Dry Needling: evidence and practice

### Firas Mourad (firas.mourad@me.com)

#### “Tor Vergata” Roma University, Roma, Italy; Alumno de Doctorado, Escuela Internacional de Doctorado, Universidad Rey Juan Carlos, Alcorcon, Madrid, Spain; Poliambulatorio Physio Power, Brescia, Italy; American Academy of Manipulative Therapy, Montgomery, AL, USA

Dry Needling (DN) is a manual therapy technique consisting in the insertion of thin monofilament needles, as used in the practice of acupuncture, without the use of injectate. Dry needling is typically used to treat muscles, ligaments, tendons, subcutaneous fascia, scar tissue, peripheral nerves, and neurovascular bundles for the management of a variety of neuromusculoskeletal pain syndromes [1]. DN is an expressly recognized competence of physiotherapy practice in many countries [2]. However, there are still some regulatory gaps in Italy. Moreover, it is essential to underline that DN is not acupuncture. Acupuncture is a regulated discipline in Italy that only Medical Doctor can practice [sentence n.482 of 27 March 2003 of the Supreme Court of Cassation, Sec. VI criminal]. However, a vast difference exists between. Acupuncture and dry needling relates to their underlying philosophy, thought processes, and decision making; the only thing they really have in common is the tool (i.e. the needle) [3]. The literature on the safety of DN is controversial. That is, as they often involve physiotherapists in many countries deliver traditional Chinese acupuncture (TCA) also [4]. However, a recent study was conducted on physiotherapist practicing DN. After 7629 treatment sessions of DN provided by physiotherapist it was not detected any serious adverse events and the authors concluded that DN provided by trained physiotherapist is safe [5]. Furthermore, a recent Systematic Review, where it was included only studies with physiotherapists practicing DN, concluded that when dry needling is utilized in appropriate patients, it may aid in decreasing musculoskeletal pain, allowing for additional, more active physical therapy interventions to maximize functional outcomes [6]. Within practitioners or disciplines, a particular group does not own, or have the rights to, a particular technique. Such restrictions, especially in medicine, would ultimately be disadvantageous to patients. Therefore, to prevent confusion and protect the patient, more clarity is needed. 

**References**

1. Dunning J, Butts R, Mourad F, Young I, Flannagan S, Perreault T. Dry needling: a literature review with implications for clinical practice guidelines. Phys Ther Rev. 2014 Aug;19(4):252-265.

2. Dommerholt J, Mayoral O, Gröbli C. Trigger point dry needling. J Man Manip Ther. 2006;14:E70-E87.

3. Unverzagt C, Berglund K, Thomas JJ. Dry needling for myofascial trigger point pain: a clinical commentary. Int J Sports Phys Ther. 2015 Jun;10(3):402-18.

4. Xu S, Wang L, Cooper E, Zhang M, Manheimer E, Berman B, Shen X, Lao L. Adverse events of acupuncture: a systematic review of case reports. Evid Based Complement Alternat Med. 2013;2013:581203.

5. Brady S, McEvoy J, Dommerholt J, Doody C. Adverse events following trigger point dry needling: a prospective survey of chartered physiotherapists. J Man Manip Ther. 2014 Aug;22(3):134-40.

6. Gattie E, Cleland JA, Snodgrass S. The Effectiveness of Trigger Point Dry Needling for Musculoskeletal Conditions by Physical Therapists: A Systematic Review and Meta-analysis. J Orthop Sports Phys Ther. 2017 Mar;47(3):133-149.

## S13 The “Case” of rhizarthrosis: the necessary cooperation between surgeon and physiotherapist in the degenerative disease

### Davide Zanin, Alessandro Pozzi

#### Unità di Chirurgia della mano e microchirurgia, Humanitas Centro della Mano – Torino

##### **Correspondence:** Davide Zanin (zanindvd@gmail.com)

**Introduction:** Thumb arthritis is a common term that is used to define the osteoarthritic changes of the carpo-metacarpal joint (CMCJ) at the base of the thumb. It is a very frequent condition that constitutes the 10% of all the forms of osteoarthritis. It is characterized by a pain at the thumb base that is intensified during pinch grips. Typical symptoms could also be considered retraction of the first web space and hyper-extension of the metacarpo-phalangeal joint (MPJ). The conservative treatment is comprehensive of all those action taken into account to reduce the pain and improve the thumb motility without considering any invasive surgical procedure. Aim of the present communication is to provide the reader with an overview of the most common conservative treatment options for thumb arthritis.

**Materials and Methods:** A literature reviews was made by the authors during the past year attempting to define the most popular conservative treatment options. An analysis of the authors current practice has been also provided to suggest which is in the authors intents the best treatment algorithm for thumb arthritis.

**Conclusion:** Both night and functional splinting together with a proper occupational reeducation are the most popular forms of conservative treatment for thumb arthritis according tot he literature review. One of the main goals of these treatments is to keep a wide first web space and to prevent deformities of the MPJ. Recent researches suggest that the strengthening of the opponent muscle and the first interosseus muscle could partially restore the CMCJ stability that got lost due to the arthritic degenerative changes.

**Results:** The optimal conservative treatment should be based on a proper night splinting and functional strategies to avoid CMCJ overload during daytime. Thermotherapy and joint distraction could provide short term benefits but both require an high compliance.

**References**

1. Cooney WP 3rd, Chao EY. Biomechanical analysis of static forces in the thumb during hand function. J Bone Joint Surg Am. 1977 Jan;59(1):27-36.

2. O'Brien VH, Giveans MR. Effects of a dynamic stability approach in conservative intervention of the carpometacarpal joint of the thumb: a retrospective study. J Hand Ther. 2013 Jan-Mar;26(1):44-51.

3. Bouton M. Role du couple oppoosant-1er interosseux dorsal dans la stabilité de l’articulation trapezo-metacarpienne. Ann Kinesither. 2000;27(7):316-324.

4. Hochberg MC, Altman RD, April KT, Benkhalti M, Guyatt G, McGowan J, Towheed T, Welch V, Wells G, Tugwell P; American College of Rheumatology. American College of Rheumatology 2012 recommendations for the use of nonpharmacologic and pharmacologic therapies in osteoarthritis of the hand, hip, and knee. Arthritis Care Res (Hoboken). 2012 Apr;64(4):465-74.

5. Caragianis S. The prevalence of occupational injuries among hand therapists in Australia and New Zealand. J Hand Ther. 2002 Jul-Sep;15(3):234-41.

## S14 Central sensitization and persistent pain in rheumatic diseases

### Andrea Polli (andrea.polli@vub.be)

#### Pain in Motion (PiM) Group http://www.paininmotion.be/; Faculty of Rehabilitation Sciences and Physiotherapy – KINE Department; Vrije Universiteit Brussel; Brussels, Belgium

Pain is a major symptom in most rheumatoid diseases, and the most disabling symptom in patients with RA [1]. For decades, pain has been considered as resulting from ongoing inflammation, thus controlling inflammatory mechanisms would have reduced pain symptoms. However, recent evidence challenges this assumption [2]. Here, we use the example of rheumatoid arthritis (RA) to propose a different approach to treat pain in chronic inflammatory diseases. RA is an auto-immune condition in which the immune system attacks one’s synovial membrane and induces bone erosion [1]. Thanks to the introduction of disease-specific drugs (DMARDs), low-dose glucocorticoids, and biological drugs that specifically target relevant inflammatory mediators, the treatment of RA has undergone great advances in the past years [1]. Disease progression has substantially slowed down and patient’s quality of life has improved. However, despite a good control of inflammation, pain remains a major problem and persists even when RA is in its remission phase [2]. Whilst pain seems to respond to anti-inflammatory drugs in the early phase of the disease, it often remains constant as the disease progresses and do not respond to DMARDs or anti-inflammatory treatment [3]. This evidence highlights that pain is not related to inflammation in the chronic phase of the disease; other mechanisms must be involved in maintaining it [2]. Accumulating evidence suggests that successful analgesia can only be achieved if the exact underlying mechanisms are addressed. A better understanding of pain in rheumatic diseases is warranted [4]. Several lines of evidence suggest that mechanisms within the central nervous system (CNS) are implicated and facilitate pain persistence [3]. The CNS undergoes plastic changes that in turn alter nociceptive processing and increase sensitivity of neurons. The hyper-excitability of the CNS (also referred to as central sensitization) is reflected in the clinic by the responses to external stimuli [4]. People with RA show widespread reduction in thermal and mechanical pain thresholds. Such hypersensitivity to noxious stimuli is not only reported in inflamed joints, but also in healthy joints [2]. In line with it, people with RA often refers widespread pain, that is not limited to the inflamed joints. Although only a few studies have focussed on treating central sensitization in RA, much more data are available from other conditions where central sensitization is a predominant feature, such as fibromyalgia [3]. People with fibromyalgia indeed show widespread pain, pain thresholds reduction, and symptoms of central sensitization [5]. Besides, they refer sleep disturbances, fatigue, and psychological distress. This same clinical picture is arising in RA too. A recent study demonstrated how a mechanism-based reasoning might detect clinically relevant subgroups of patients with RA [6]. Among a sample of 169 patients, 50% of the sample showed low level of inflammation and low pain, and about 15% of patients were in the active state of the disease, with elevated inflammation, swollen joints, pain and fatigue. Importantly, around 35% of the sample showed minimal inflammation but intense widespread pain, fatigue, psychological distress, and sleep disturbances.

Clinicians treating people with inflammatory disease need to take into account this recent evidence and consider treatments that target central mechanisms. Centrally acting drugs (pregabalin, gabapentin, selective serotonin- and noradrenaline- reuptake inhibitor, etc.) should accompany disease specific medications when pain is not associated to inflammation [3]. Similarly, behavioural strategies able to decrease central sensitization, such as pain neuroscience education, sleep management, and regular moderate physical activity [7,8], should be foster to successfully reduce these patients’ pain and improve their quality of life.

**References**

1. Smolen JS, Aletaha D, McInnes IB. Rheumatoid arthritis. Lancet. 2016 Oct 22;388(10055):2023-2038.

2. Walsh DA, McWilliams DF. Mechanisms, impact and management of pain in rheumatoid arthritis. Nat Rev Rheumatol. 2014 Oct;10(10):581-92.

3. Lee YC, Nassikas NJ, Clauw DJ. The role of the central nervous system in the generation and maintenance of chronic pain in rheumatoid arthritis, osteoarthritis and fibromyalgia. Arthritis Res Ther. 2011 Apr 28;13(2):211.

4. Woolf CJ. Central sensitization: implications for the diagnosis and treatment of pain. Pain. 2011 Mar;152(3 Suppl):S2-15.

5. Staud R, Domingo M. Evidence for abnormal pain processing in fibromyalgia syndrome. Pain Med. 2001 Sep;2(3):208-15.

6. Lee YC, Frits ML, Iannaccone CK, Weinblatt ME, Shadick NA, Williams DA, Cui J. Subgrouping of patients with rheumatoid arthritis based on pain, fatigue, inflammation, and psychosocial factors. Arthritis Rheumatol. 2014 Aug;66(8):2006-14.

7. Nijs J, Malfliet A, Ickmans K, Baert I, Meeus M. Treatment of central sensitization in patients with 'unexplained' chronic pain: an update. Expert Opin Pharmacother. 2014 Aug;15(12):1671-83.

8. Nijs J, Loggia ML, Polli A, Moens M, Huysmans E, Goudman L, Meeus M, Vanderweeën L, Ickmans K, Clauw D. Sleep disturbances and severe stress as glial activators: key targets for treating central sensitization in chronic pain patients? Expert Opin Ther Targets. 2017 Aug;21(8):817-826

## S15 Adolescent idiopathic scoliosis: Options for an effective and reasonable conservative treatment

### Michele Romano (michele.romano@isico.it)

#### ISICO (istituto Scientifico Italiano Colonna vertebrale), SOSORT (Society On Scoliosis Orthopedic and Rehabilitation Treatment)

Typically, in all families, the idea of the misalignment of the child’s spine is a latent concern due to stress. The reason for this stress is not immediately understood because scoliosis is not a mortal disease, it does not cause disability and it has a quite low prevalence. Starting a rehabilitation treatment, the gold objective we intend is the healing of the patient. We can obtain this when we know the aetiology and we can treat the cause, or when our body is able to self-nurse itself (maybe with our help). In case of scoliosis we have no one of these options. We don’t know the aetiology. The self-healing of scoliosis never had been observed. If the spinal misalignment is evolutive, the only type of evolution is the worsening. How can we proceed? Until now we should be content and work on “secondary” targets because it is impossible to work on the “causal” target. These secondary targets are the opposition to the misalignment and the increasing of the capacity of the spine to hold this correction. The first objective is reached using an active and conscious therapeutic movement named “self-correction”. Why is this action so important? This depends on our ignorance about the aetiology. Until now we don’t know the origin of the disease and therefore we can only try to influence the trend but not really affect it. The second objective is reached by improving the function of the stabilizing muscles of the spine to counter the postural collapse. The tools to reach this are the specific exercises. These exercises will be used not to improve the strength of the stabilization muscles of the trunk but to challenge the holding of the correction, putting the patient in a difficult postural situation to obtain the automatic involvement of these muscles.

**References**

1. Negrini S, Minozzi S, Bettany-Saltikov J, Zaina F, Chockalingam N, Grivas TB, Kotwicki T, Maruyama T, Romano M, Vasiliadis ES. Braces for idiopathic scoliosis in adolescents. Spine (Phila Pa 1976). 2010 Jun 1;35(13):1285-93.

2. Romano M, Minozzi S, Bettany-Saltikov J, Zaina F, Chockalingam N, Kotwicki T, Maier-Hennes A, Negrini S. Exercises for adolescent idiopathic scoliosis. Cochrane Database Syst Rev. 2012 Aug 15;(8):CD007837.

3. Berdishevsky H, Lebel VA, Bettany-Saltikov J, Rigo M, Lebel A, Hennes A, Romano M, Białek M, M'hango A, Betts T, de Mauroy JC, Durmala J. Physiotherapy scoliosis-specific exercises - a comprehensive review of seven major schools. Scoliosis Spinal Disord. 2016 Aug 4;11:20.

4. Romano M, Negrini A, Parzini S, Tavernaro M, Zaina F, Donzelli S, Negrini S. SEAS (Scientific Exercises Approach to Scoliosis): a modern and effective evidence based approach to physiotherapic specific scoliosis exercises. Scoliosis. 2015 Feb 5;10:3.

5. Negrini S, Donzelli S, Aulisa AG, Czaprowski D, Schreiber S, de Mauroy JC, Diers H, Grivas TB, Knott P, Kotwicki T, Lebel A, Marti C, Maruyama T, O'Brien J, Price N, Parent E, Rigo M, Romano M, Stikeleather L, Wynne J, Zaina F. 2016 SOSORT guidelines: orthopaedic and rehabilitation treatment of idiopathic scoliosis during growth. Scoliosis Spinal Disord. 2018 Jan 10;13:3.

## S16 A rehabilitative-school integrated program to improve the Quality of Life of children affected by rheumatic diseases: Pilot Study

### Carla De Conti^1^, Francesca Rodà^2^, Eleonora Salomon^2^, Annalisa Arlotta^3^, Patrizia Bertolini^3^, Luciano Selleri^4^, Rodolfo Brianti^2^, Annamaria Salghetti^2^

#### ^1^SinergyMED 2.0, Conegliano; ^2^Struttura Complessa di Medicina Riabilitativa Azienda Ospedaliero-Universitaria di Parma; ^3^Dipartimento di Medicina e Chirurgia, Unità di Neuroscienze, Università degli Studi di Parma; ^4^Ufficio Scolastico Regionale, Ambito Territoriale Scolastico di Parma e Piacenza

##### **Correspondence:** Carla De Conti (carla.deconti@sinergymed.it)

**Introduction:** In Italy about 10,000 children are affected by rheumatic diseases (RD) every year. The most widespread is Juvenile Idiopathic Arthritis (JIA), followed by different forms of inflammation of the connective tissue such as Systemic Lupus Erythematosus (SLE), Dermatomyositis and ultimately, the less frequent but equally disabling Fibromyalgia. The clinical and operational framework of paediatric RD varies widely in its manifestations but always presents a limited participation in motor activities of the everyday life, such as recreational and sporting activities, which are typical of school-aged children. To date, there are few scientific studies concerning the importance and effectiveness of the rehabilitative-educational and self-management aspects of the disease. Moreover, there are no clinical studies concerning the adaptation to physical activity in groups. This pilot study aims to investigate these aspects by including guidance figures (by relatives, care-givers and teachers) and by evaluating possible changes to make to the patients’ participation to physical activity in and outside of school.

**Materials and Methods:** Subjects: 18 paediatric patients with RD aged 7 to 16. Centers involved: Struttura Complessa di Medicina Riabilitativa dell’AOU di Parma, Unità Operativa Complessa Onco-Ematologia Pediatrica dell’AOU di Parma, Provveditorato degli Studi di Parma. Intervention: The patients met both the Physiatrist and the Physiotherapist for a first functional and motor assessment (T1): mobility test , manual muscle test (MRC), aerobic capacity (6 MWT), pain and disease perception (VAS). During the same meeting, JAMAR, a questionnaire on the quality of life, was given out together with a prospective diary to record any activity during the study period. A second assessment was conducted at 9 months from the end of the treatment. In addition to what was covered in the first evaluation, this one also included the data recorded in the “diary” of patients and caregivers regarding the participation to inside- and outside-school physical activities. Based on the outcome of the T1, a plan of the activities that small groups of evenly aged children sharing the same functional impairment had to carry out in the "rehabilitation garden" and the school's gyms was designed. The patients performed functional, aerobic and muscular strengthening exercises and received guidance on ergonomics and physical education and recreational activities calibrated to the characteristics of the group. Each child completed 6 sessions of rehabilitation, lasting two hours each, 1 day/week for 6 weeks. Prior to intervening with the patients, sessions with the school were organized for the coordination and definition of the planned initiatives and for teacher training. In addition, meetings were held to discuss any confrontations/reports/updates regarding the project, with paediatricians.

**Results:** Sample size calculated: 46 subjects. We proposed the project to 27 eligible patients, 18 enrolled and 11 completed the whole treatment. The main diagnosis was JIA, with mild to moderate functional involvement. The project and treatment were well accepted and tolerated by patients and their families; both of them asked for additional meetings. Teachers contributed actively in designing and developing each session. There was no statistically significant difference in each test at the end of the project, probably due to the insufficient size and heterogeneity of our sample. This new rehabilitative approach highlighted the efficacy of a multidisciplinary collaboration between a hospital, a school and a patient (and his/her family).

**Conclusion:** Despite the lack of significant differences in objective measures, this study demonstrated the feasibility of an integrated rehabilitative-school program “quality of life-based” for children affected by rheumatic diseases, their family and the school staff. Furthermore, we consider this model easily exportable and reproducible in other healthcare institutions.

**References**

1. Russo E, Trevisi E, Zulian F, Battaglia MA, Viel D, Facchin D, Chiusso A, Martinuzzi A. Psychological profile in children and adolescents with severe course Juvenile Idiopathic Arthritis. ScientificWorldJournal. 2012;2012:841375.

2. Klepper SE. Exercise in pediatric rheumatic diseases. Curr Opin Rheumatol. 2008 Sep;20(5):619-24.

3. Murphy NA, Carbone PS; American Academy of Pediatrics Council on Children With Disabilities. Promoting the participation of children with disabilities in sports, recreation, and physical activities. Pediatrics. 2008 May;121(5):1057-61.

4. Long AR, Rouster-Stevens KA. The role of exercise therapy in the management of juvenile idiopathic arthritis. Curr Opin Rheumatol. 2010 Mar;22(2):213-7.

5. Takken T, van Brussel M, Engelbert RH, Van der Net J, Kuis W, Helders PJ. Exercise therapy in juvenile idiopathic arthritis. Cochrane Database Syst Rev. 2008 Apr 16;(2):CD005954.

6. ATS Committee on Proficiency Standards for Clinical Pulmonary Function Laboratories. ATS statement: guidelines for the six-minute walk test. Am J Respir Crit Care Med. 2002 Jul 1;166(1):111-7. Erratum in: Am J Respir Crit Care Med. 2016 May 15;193(10):1185.

7. Filocamo G, Consolaro A, Schiappapietra B, Dalprà S, Lattanzi B, Magni-Manzoni S, Ruperto N, Pistorio A, Pederzoli S, Civino A, Guseinova D, Masala E, Viola S, Martini A, Ravelli A. A new approach to clinical care of juvenile idiopathic arthritis: the Juvenile Arthritis Multidimensional Assessment Report. J Rheumatol. 2011 May;38(5):938-53.

8. Tarakci E, Yeldan I, Baydogan SN, Olgar S, Kasapcopur O. Efficacy of a land-based home exercise programme for patients with juvenile idiopathic arthritis: a randomized, controlled, single-blind study. J Rehabil Med. 2012 Nov;44(11):962-7.

## S17 “The back goes to school”: presentation about a pilot project in Lazio (Italy)

### Ambra Galante^1,2^, Vincenzo Cabala^1,3^, Hilenia Catania^1,2^

#### ^1^Master in Pediatric Physiotherapy, University of Florence, Rehabilitation Department Meyer Children's Hospital, Florence, Italy; ^2^ Freelance, Rome; ^3^Rehabilitation Department, Vaclav Vojta Center, Rome

##### **Correspondence:** Ambra Galante (ambragalante@fastwebnet.it)

**Background:** In Italy the prevention program called "Okkio alla Salute" has been active for many years. This program aims to study the distribution of excess weight and risk behaviors in primary school children. In 2014 it emerges that 31% of children are overweight in the Lazio region, with prevalence in the province of Frosinone. Recent literature, related to the correlation between body mass index (BMI) and trunk asymmetry, shows that most overweight people also have trunk asymmetry. In autumn 2015 the school district of Frosinone officially requires the collaboration of Italian Association of Physiotherapists (AIFI) in the district of Lazio. The aim is to integrate OKKIO ALLA SALUTE data with the incidence of Paramorphisms and column dysmorphisms in the Province of Frosinone. AIFI Lazio welcomes the request and involves the Pediatric Specialist Interest Group of AIFI (GIS), identifying 3 Physiotherapists Specialists in the Pediatric Area as evaluators. Another aim of the project is to implement prevention and health promotion programs in the school context.

**Materials and Methods:** Screening was done between April and May 2016 in five days. The children of 12 classes (fourth and fifth grade) in the province of Frosinone were evaluated. The evaluation team consisted of a school doctor, a nurse and three physiotherapists. The materials used for the postural assessment were: an evaluation form created specifically for screening, an anthropometer, measuring tape. The card was composed of: a medical part filled in by the doctor (personal data, weight, height, any relevant news, treatments in progress, sports) and a physiotherapeutic part (pain, dysmetria, ligamentous laxity, the characteristics of walking). It was noted: the visual impression of impact (that is, if the child appeared frankly asymmetric or symmetrical) the posture in anterior, posterior and lateral view and the result of the bending test. At the end of the card the indications of the team were noted regarding the need for an orthopaedic or nutritional visit, subsequently handed over to the families. In conclusion, a meeting was held with parents to discuss the screening result and to provide information on scoliosis and its treatment.

**Results:** Of 120 children evaluated only 18% were overweight and 70% appeared symmetrical at a first observation. The bending test was positive in 60% of the cases but only in 26% of the cases was it sent to the specialist doctor. No correlation between overweight and alteration to bending test or between overweight and evident postural asymmetries was found.

**Discussion:** Although a relationship between overweight and asymmetry of the trunk has not been found, an interesting data regards the positive subjects to the bending test: 65% of these appeared symmetrical to the operators at a first observation. This means that a specialized assessment is required for all school-age subjects, to identify the cases at risk that could also be among those apparently symmetrical. In this vision, it is essential to implement screening programs in schools.

**Conclusion:** The strong point of this project were the multidisciplinary team, the respect of the correct institutional path to school-aifi-pediatric specialist interest group, the low cost for families both economic and time, the serenity of children in visiting, in a protected environment like school. The aspects to be improved relate to the evaluation form by inserting more sensitive measuring instruments (scolio meter). A path of this kind promoted by local health authorities and local public administrations is desirable.

**References**

1. Urquhart DM, Berry P, Wluka AE, Strauss BJ, Wang Y, Proietto J, Jones G, Dixon JB, Cicuttini FM. 2011 Young Investigator Award winner: Increased fat mass is associated with high levels of low back pain intensity and disability. Spine (Phila Pa 1976). 2011 Jul 15;36(16):1320-5.

2. Durmala, J, Sosnowska M, Sosnowski M. Nutritional status in idiopathic scoliosis. Scoliosis. 2012;7(1):O22.

3. Grivas TB, Burwell RG, Mihas C, Vasiliadis ES, Triantafyllopoulos G, Kaspiris A. Study of body mass (BMI) index and truncal asymmetry (TA) in healthy adolescents. Scoliosis. 4(2);O9.

4. Grivas TB, Burwell RG, Mihas C, Vasiliadis ES, Triantafyllopoulos G, Kaspiris A. Relatively lower body mass index is associated with an excess of severe truncal asymmetry in healthy adolescents: Do white adipose tissue, leptin, hypothalamus and sympathetic nervous system influence truncal growth asymmetry? Scoliosis. 2009 Jun 30;4:13.

5. Grivas TB, Burwell GR, Dangerfield PH. Body mass index in relation to truncal asymmetry of healthy adolescents, a physiopathogenetic concept in common with idiopathic scoliosis: summary of an electronic focus group debate of the IBSE. Scoliosis. 2013 Jun 25;8(1):10.

## S18 Project: “the back goes to school”: territorial realities and literature review

### Antonella D’Aversa (anto.daversa@gmail.com)

#### Regional Referent “Pediatric Physiotherapy” of AIFI – Puglia

The project “The back goes to school” was presented and promoted in Puglia for the first time in 2012, when two experienced physical therapists for district and a provincial referent went to the schools of the five apulian provinces to provide children information on spinal deformities and submit them to the Adams test to perform a first assessment screening. Now, we have a large press review of that project but, unfortunately, no scientific data to start a wider research project.

In 2016, the project was reviewed, then presented to physiotherapists during the training event “Evidenze scientifiche nel trattamento riabilitativo per le deformità del rachide in età evolutiva” organized by AIFI Puglia with the Pediatric Physiotherapy and Manual Therapy Groups (Lecce, April 2017). On that occasion the colleagues were identified and trained to goes at schools of Apulian provinces to present the project “The back goes to school”. This opportunity was also propitious to review the most up-to-date scientific literature on the spinal deformity and to make a comparison with what happens, especially for the prevention of such deformities, in the world.

Part of this literature was presented and discussed during “La parabola del rachide: dismorfismi e malattie reumatiche dall’età evolutiva all’adulto” at the International Scientific Congress of AIFI (Rome, October 2017).

Specifically, with a very interesting systematic review [1] it’s focusing attention on the importance of school screening programs as well as on the difficulties related to the bureaucratic, logistic and economic aspects of screening in the world. Except for some Eastern countries (China, Japan) [2], Australia and Sweden, in other countries, including Europe, the school screening programmes are not compulsory, on an exclusively voluntary basis for physiotherapists and without fees for educational institutions. These projects are very interesting for schools but not very reproducible over time. The problem of data collection, the roles to be established and the times to be met must be added to the problem of costs.

The discussion therefore declared the need to review planning, also from a political and logistic point of view, to establish agreement protocols and, perhaps, to restore that now disappeared but extremely useful school medicine for the prevention of children’s musculoskeletal problems.

**References**

1. Altaf F, Drinkwater J, Phan K, Cree AK. Systematic Review of School Scoliosis Screening. Spine Deform. 2017 Sep;5(5):303-309.

2. Deepak AS, Ong JY, Choon D, Lee CK, Chiu CK, Chan C, Kwan MK. The Clinical Effectiveness of School Screening Programme for Idiopathic Scoliosis in Malaysia. Malays Orthop J. 2017 Mar;11(1):41-46.

## S19 History of a AIFI Project: experience into Piedmont and Aosta Valley territory

### Gabriella Carpanese, Denis Janin, Giuseppe Tedesco

#### Gis Fisioterapia Pediatrica, Aifi Piemonte e Valle D'Aosta

##### **Correspondence:** Gabriella Carpanese (formica1962@gmail.com)

The following report outlines the history of the Project “spine goes to school” in the Piedmont and Aosta Valley territory and its evolution over time. In 2011, after a pilot experience in the primary schools in the town of Cuneo, AIFI PDF went on developing the Project “spine goes to school”.

This Project will be included into health care educational and promotional regional projects, as low back pain prevention and intervention during school age: it is addressed to children in their sixth grade.

AIFI PVD is signing a “Memorandum of Understanding” with: the Ministry of Education, University and Research, General Directorate, Regional School Department; the Piedmont Region: Education Directorate for Education, Professional Vocational Education and Jobs, and Directorate for the Health Protection; the City of Turin, Sport Department the Project includes different phases, such as: a physiotherapy training phase, where AIFI PVD carries on the initiative into the schools; a meeting with the headmaster of the selected school

and a contact person for the teachers: here the physiotherapist will introduce the project and deliver a questionnaire worked out for the children’s parents and will agree upon the working time schedule in the school; two meetings in the classroom are to take place two months’ time between: in the classroom, since this is the place where children spend most time and will acquire the main notions covering spine ergonomy.

The first meeting (lasting two hours) will provide: collection of questionnaires proposed to the parents; an interactive practical and theoretical path, aimed to the spine knowledge: sitting posture in the classroom, how to pack, prepare and carry a backpack; the delivery of a warrant surrender of a poster synthesizing the concepts: its scope is to take part to a contest providing a great prize.

During a second meeting, lasting one hour, the students will fill up a learning form - during the meeting the learnt concepts will be reminded and the poster is delivered.

The final party will take place in Turin: it is the conclusive event where the results emerging from the forms are presented, the posters are displayed and the winners of the contest are declared.

The winners will be awarded by sport champions, both disabled and able.

From 2011 through 2015 four editions took place: again the project was started during the school year 2016-2017 keeping the same structure - it was carried forward by some physiotherapists on personal initiative backed by AIFI PVD

The results: along these years the project has involved 185 schools, 5137 students, 240 physiotherapists.

Upshots: after our experience we can conclude: it’s important for AIFI PVD to go on backing this training and prevention activity in the schools.

Besides the direct work with the students in their school, the project might evolve: a useful route for both teachers and parents can be provided, in order to considerably improve the lifestyle of the boys.

Moreover, a more accurate mode of data collection should be identified in order to better calibrate any future interventions.

The Memorandum of Understanding with the Institutions is useful to yield visibility to the project: on the other side, it requires much struggle and is conditioned by several variables. To carry on the project on an individual basis, directed by AIFI PVD, currently seems to be the easy way to ensure continuity in our region.

In future we hope AIFI National to start up a look out on the trend of project “spine goes to school”: it should be understood - in a variable environment and in regional actualitis which are different between - how the physiotherapist can carry forward his training and prevention activity concerning this problem at school age.

## S20 To write a scientific article: the meaning of the checklists

### Roberto Gatti (roberto.gatti@hunimed.eu)

#### Humanitas Research Centre and University

Scientific research articles provide a method for scientists to communicate with other scientists about the results of their research but without a complete description of the intervention other researchers cannot replicate or build on research findings or it is not clear to decision makers how to reliable implement the intervention (1).

In order to deal meaning and development of the checklists regarding the well writing of a scientific article, the reporting of the experimental studies has been considered in this presentation.

In 1980s, the IMRAD style was defined as modality of scientific reporting. Articles were divided in Introduction, Methods, Results and Discussion (2). Introduction is dedicated to provide the context of the study and to define its objective, Methods are addressed to provide details to allow researchers to do the same experiment, Results have to synthetically show the results arising from methods, and Discussion have to discuss the results inside the context introduced at the beginning of the article. Reporting a scientific article should retrace the questions which have led the researcher to promote the study: why (Introduction), how (Methods), what (Results) and so what (Discussion).

In 1996 the IMRAD structure was implemented with the first edition of the CONSORT checklist. This edition had subheadings and descriptors able to detail how trial was performed. The need to implement the IMRAD structure is understandable from the words of prof Altman: “ the CONSORT statement means that authors will no longer be able to hide inadequacies in their study by omitting important information…” (3).

In 2010 Hopewell (4) published an interesting comparative study about the differences in reporting methodological items in journals indexed on PubMed in the years 2000 and 2006. The trend showed a significant increase in following the CONSORT checklist, although: the situation remained sub-optimal; did not involve all items as, for example, the blinding; did not involve all scientific journals. Similar results were reported in 2012 in a systematic review with meta-analysis published on the Cochrane Database of Systematic Review (5).

In those years some authors began to warn about the inappropriateness of the CONSORT checklist for the non-pharmacological trials. In 2007 Boutron (6) highlighted that the CONSORT checklist was not entirely applicable to non-pharmacologic trials as it forecast interventions involving several components; items as blinding are more difficult to achieve; experimental designs relies on more complex methods. Few time later, always Boutron published the extension of the CONSORT checklist for trials assessing non-pharmacologic treatment (7). This checklist stressed some aspects linked to the role and the intervention modalities of people involved in the studies.

Nevertheless, the CONSORT checklist for non-pharmacologic trials is not sufficient for the reporting of physiotherapy studies. Physiotherapy intervention are multimodal; involve the use of manual techniques, consumable materials, equipment, education, training and feedback. Moreover, the dose or intensity of treatment may be progressed over time (8).

From these considerations, in 2014 has been proposed, as further development of the CONSORT checklist, the Template for Intervention Description and Replication checklist and guide (TIDieR) (9). Its main characteristic is that all the details inherent every possible sources of variability in determining the results of the study have to be described. For example, it is no longer acceptable to report the intervention administered to control group as “usual care”.

It is even more clear that to correctly report an experimental study is not only a favor to other researchers but also a modality to improve own methodological skills.

**References**

1. CONSORT, transparent reporting of trial. http://www.consort-statement.org/resources/tidier-2

2. Sollaci LB, Pereira MG. The introduction, methods, results, and discussion (IMRAD) structure: a fifty-year survey. J Med Libr Assoc. 2004 Jul;92(3):364-7.

3. Altman DG. Better reporting of randomised controlled trials: the CONSORT statement. BMJ. 1996 Sep 7;313(7057):570-1.

4. Hopewell S, Dutton S, Yu LM, Chan AW, Altman DG. The quality of reports of randomised trials in 2000 and 2006: comparative study of articles indexed in PubMed. BMJ. 2010 Mar 23;340:c723.

5. Turner L, Shamseer L, Altman DG, Weeks L, Peters J, Kober T, Dias S, Schulz KF, Plint AC, Moher D. Consolidated standards of reporting trials (CONSORT) and the completeness of reporting of randomised controlled trials (RCTs) published in medical journals. Cochrane Database Syst Rev. 2012 Nov 14;11:MR000030.

6. Boutron I, Guittet L, Estellat C, Moher D, Hróbjartsson A, Ravaud P. Reporting methods of blinding in randomized trials assessing nonpharmacological treatments. PLoS Med. 2007 Feb;4(2):e61.

7. Boutron I, Moher D, Altman DG, Schulz KF, Ravaud P; CONSORT Group. Extending the CONSORT statement to randomized trials of nonpharmacologic treatment: explanation and elaboration. Ann Intern Med. 2008 Feb 19;148(4):295-309.

8. Yamato TP, Maher CG, Saragiotto BT, Hoffmann TC, Moseley AM. How completely are physiotherapy interventions described in reports of randomised trials? Physiotherapy. 2016 Jun;102(2):121-6.

9. Hoffmann TC, Glasziou PP, Boutron I, Milne R, Perera R, Moher D, Altman DG, Barbour V, Macdonald H, Johnston M, Lamb SE, Dixon-Woods M, McCulloch P, Wyatt JC, Chan AW, Michie S. Better reporting of interventions: template for intervention description and replication (TIDieR) checklist and guide. BMJ. 2014 Mar 7;348:g1687.

## S21 Single-Subject Design: Experimental Designs for Research and for Clinical Practice

### Stefania Costi^1,2,3^, Davide Corbetta^4,5^

#### ^1^Physical Medicine and Rehabilitation Unit - Arcispedale Santa Maria Nuova-IRCCS, Viale Risorgimento 80, 42123, Reggio Emilia, Italy; ^2^Department of Surgery, Medicine, Dentistry and Morphological Sciences, University of Modena and Reggio Emilia, Via del Pozzo 71, 41124, Modena, Italy; ^3^Department of Neuroscience, Rehabilitation, Ophthalmology, Genetics and Maternal Child Health, University of Genoa, L.go P. Daneo n°3, 16132, Genoa, Italy; ^4^Department of Rehabilitation and Functional Recovery, San Raffaele Scientific Institute, Via Olgettina 60, 20132 Milan, Italy; ^5^Physiotherapy Degree Course, Vita-Salute San Raffaele University, Via Olgettina 58, 20132 Milan, Italy

##### **Correspondence:** Davide Corbetta (corbetta.davide@hsr.it)

**Background:** The individual variability among people presenting motor impairments often leads to the difficulty to obtain an adequate sample size in the conduction of trials in physiotherapy. Furthermore, in clinical practice, it is often difficult to recognize the relationship between the administration of a treatment and its expected results. Psychological and educational sciences often use single-subject design (SSD) studies to explore behaviours under experimental conditions. This study design allows to test the relationship between an independent variable, the treatment, and a dependent variable, the main outcome of interest. The purpose of this work is to present researchers and clinicians the methodology of the SSD studies and their application in physiotherapy both in research context and everyday practice [1].

**Results:** In SSD studies, repeated measurements of the outcome of interest occur across time starting from a condition without treatment, the so called “A-phase”, and continuing during the administration of the treatment, the so called “B-phase”. A-phase measurements serve as a standard of performance that can be compared to B-phase measurements in terms of change in the mean level, change in trend or change in variability of measure, depending on the nature of the assessed outcome. Different types of SSD studies exist, those alternating introduction and removal of the treatment called “treatment removal”, following the AB, ABA or ABAB schemes, those with the introduction of one or more alternative treatments, named C, D and so on, called “alternating treatments”, following the ABACAD scheme, those with a progression of different treatments according to achieved levels of the outcome of interest called “changing criterion”, following the ABCD scheme, and those where more subjects follow the scheme of alternating phases starting at different time points, called “multiple baseline” [2].

**Conclusions:** SSD studies offer an option for the identification of an individual response to a specific intervention when traditional between-group designs would not be appropriate both in clinical and research contexts. SSD studies result in acceptable internal validity but in very low external validity.

**References**

1. Romeiser-Logan L, Slaughter R, Hickman R. Single-subject research designs in pediatric rehabilitation: a valuable step towards knowledge translation. Dev Med Child Neurol. 2017 Jun;59(6):574-580

2. Graham JE, Karmarkar AM, Ottenbacher KJ. Small sample research designs for evidence-based rehabilitation: issues and methods. Arch Phys Med Rehabil. 2012 Aug;93(8 Suppl):S111-6.

## S22 ACL injuries: clinical management and return to sport

### Alberto Vascellari (mascvoz@gmail.com)

#### Orthopaedic and Traumatology Department, Oderzo Hospital, Oderzo, Treviso, Italy

The rehabilitation protocols of anterior cruciate ligament reconstruction (ACLr), should follow the criteria of evidence based practice. They should therefore follow the guidelines proposed by the literature, the clinical expertise that emerges from surveys, so that surgeon’s and rehabilitation staff’s personal experiences, trying to satisfy the patient's values, since rehabilitation should be as customized as possible. The aim of this paper is to report rehabilitation guidelines proposed by the literature and rehabilitation approaches of the “ACL Study Group” (ACLsg) and of the Italian surgeons of the SIGASCOT (Società Italiana del Ginocchio Artroscopia Sport Cartilagine Tecnologie Ortopediche) [1].

There is no evidence in the literature to support the use of post-operative brace. Several biomechanical studies have shown the effectiveness in reducing the loads on the ACL, while some clinical studies reported that bracing does not protect against post-operative injury, does not decrease pain, produce changes in rom , or improve knee stability [2]. However, brace is used with a rate of 35 % in ACLsg, while this rate rises to 49 % in SIGASCOT members [1].

After ACLr, full knee extension ROM should be achieved as soon as possible. Extension loss results in abnormal joint arthrokinematics at both the tibiofemoral and patellofemoral joints. This in turn leads to abnormal articular cartilage contact pressures and quadriceps inhibition

77% of the ACLsg allow immediately full ROM after ACLr, while 41 % of SIGASCOT members limited the flexion at different degrees within the first 2 weeks. In a randomized controlled trial, Ito et al. [3] reported no laxity associated with ROM exercises immediately after ACLr with hamstring autograft.

A randomized trial compared the efficacy of immediate weight-bearing versus a delay of 2 weeks following autograft patellar tendon ACLr [4] and reported no deleterious effects and decreased incidence of anterior knee. While a third of SIGASCOT surgeons allow patients to load the operated knee as much as tolerated within the first 2 weeks, other surgeons limited the loading at different timing. The author’s protocol permits full weight bearing only when patients have a complete extension ROM and no extension lag.

The quadriceps are an important dynamic knee joint stabiliser during closed kinetic chain (CKC) activities [5]. Early CKC quadriceps exercise are associated with significantly more high clinical scores while average knee laxity was not significantly affected. The ACL strain responses produced during CKC exercises are equal and similar to those produced during other rehabilitation exercises (i.e., squatting, active extension of the knee). During open kinetic chain (OKC) activities, an anterior shear force from approximately 38° of flexion to full extension has been reported [6]. In one study [7] early start of OKC quadriceps exercises after hamstring ACLr resulted in significantly increased anterior knee laxity in comparison with late start and with early and with late start after bone –patellar tendon– ACLr. There was no general trend of increased anterior knee laxity over time between 3 and 7 months. In agreement with literature, the majority of SIGASCOT surgeons (88 %) preferred to start quadriceps strengthening okc exercises between 90 and 40° after 6 weeks [1]. The author’s protocol introduce OKC after 2 or 3 months, according to the kind of graft utilized.

About return to sports (RTS), 73 % of SIGASCOT members allowed RTS between the 6^th^ and the 8^th^ month [1]. Gokeler et al. [8] assessed patients 6 months after ACLr with a RTS test battery, and found that only two out of 28 patients passed all criteria of the test protocol. This findings suggest that the majority of patients 6 months after ACLr require additional rehabilitation to pass RTS criteria.

**References**

1. Vascellari A, Grassi A, Combi A, Tomaello L, Canata GL, Zaffagnini S; SIGASCOT Sports Committee. Web-based survey results: surgeon practice patterns in Italy regarding anterior cruciate ligament reconstruction and rehabilitation. Knee Surg Sports Traumatol Arthrosc. 2017 Aug;25(8):2520-2527.

2. Wright RW, Haas AK, Anderson J, Calabrese G, Cavanaugh J, Hewett TE, Lorring D, McKenzie C, Preston E, Williams G; MOON Group. Anterior Cruciate Ligament Reconstruction Rehabilitation: MOON Guidelines. Sports Health. 2015 May;7(3):239-43.

3. Ito Y, Deie M, Adachi N, Kobayashi K, Kanaya A, Miyamoto A, Nakasa T, Ochi M. A prospective study of 3-day versus 2-week immobilization period after anterior cruciate ligament reconstruction. Knee. 2007 Jan;14(1):34-8.

4. Tyler TF, McHugh MP, Gleim GW, Nicholas SJ. The effect of immediate weightbearing after anterior cruciate ligament reconstruction. Clin Orthop Relat Res. 1998 Dec;(357):141-8.

5. Bodor M. Quadriceps protects the anterior cruciate ligament. J Orthop Res. 2001 Jul;19(4):629-33. PubMed PMID: 11518272.

6. Wilk KE, Escamilla RF, Fleisig GS, Barrentine SW, Andrews JR, Boyd ML. A comparison of tibiofemoral joint forces and electromyographic activity during open and closed kinetic chain exercises. Am J Sports Med. 1996 Jul-Aug;24(4):518-27.

7. Heijne A, Werner S. Early versus late start of open kinetic chain quadriceps exercises after ACL reconstruction with patellar tendon or hamstring grafts: a prospective randomized outcome study. Knee Surg Sports Traumatol Arthrosc. 2007 Apr;15(4):402-14. Epub 2007 Jan 12. Erratum in: Knee Surg Sports Traumatol Arthrosc. 2007 Apr;15(4):472-3. Knee Surg Sports Traumatol Arthrosc. 2007 Apr;15(4):472-3.

8. Gokeler A, Welling W, Zaffagnini S, Seil R, Padua D. Development of a test battery to enhance safe return to sports after anterior cruciate ligament reconstruction. Knee Surg Sports Traumatol Arthrosc. 2017 Jan;25(1):192-199. doi: 10.1007/s00167-016-4246-3.

## S23 ACL injury: Clinical Management and Return to Sport

### Davide B. Albertoni (albertoni.d@gmail.com)

#### Private Practitioner, Codogno (LO)

Return to sport (RTS) is often considered in different meanings, but the choice of a "limited" definition can largely influence therapeutic success. In order to have a more adequate picture of the current outcomes of surgical treatment of ACL reconstruction and of conservative approach in this lesion, it is appropriate to use the definition of Morris (2016), which defines the RTS as “the period of time following the reconstruction of ACL within which the athlete competes at the pre-injury level with other athletes in official events”.

With this definition the rate of RTS after surgical reconstruction of the ACL remains rather low, which is around 65% (Ardern, 2014), with an even lower percentage if the sport is practiced at a competitive level. Moreover, the percentages of recurrence are significant (around 8-9%), both for the operated limb but also for the contralateral limb (Wiggins, 2016). These percentages are also much higher in subjects younger than 25 years old. Furthermore, the likelihood of developing osteoarthritis appears to be higher in the ACL reconstruction than in subjects with ACL injury who instead opted for a conservative approach.

In light of these data, it is necessary to re-evaluate the role of ACL surgical reconstruction and pay close attention to the RTS criteria proposed in the various studies, to identify the appropriateness of these and to address the rehabilitative treatment with greater specificity. The most common criterion for RTS is time, that is usually around 6 months, but recent systematic reviews and more recent publications now consider it too short to really reach appropriate treatment goals. In addition, a review on the recovery times of the professional athletes of the American Premier Leagues, indicates an average RTS of about 1 year, with some athletes who recover even beyond that period (Mai, 2017)

The likelihood of recurrence of injury is higher in the first two years after ACL reconstruction, and it is not even possible to discriminate against the most risky athletes based on performance, since athletes with recurrence of injury are frequently those with better performance and shorter RTS. It is therefore necessary to modify the RTS criteria, increasing the minimum time for the resumption of sporting activity, which must be a choice shared by more professionals, which foresees a process of gradual exposure, which follows objective criteria, with high quantitative values and takes into account also qualitative and psychological factors (Dingenen, 2017)

**References**

1. Morris RC, Hulstyn MJ, Fleming BC, Owens BD, Fadale PD. Return to Play Following Anterior Cruciate Ligament Reconstruction. Clin Sports Med. 2016 Oct;35(4):655-68.

2. Ardern CL, Taylor NF, Feller JA, Webster KE. Fifty-five per cent return to competitive sport following anterior cruciate ligament reconstruction surgery: an updated systematic review and meta-analysis including aspects of physical functioning and contextual factors. Br J Sports Med. 2014 Nov;48(21):1543-52.

3. Wiggins AJ, Grandhi RK, Schneider DK, Stanfield D, Webster KE, Myer GD. Risk of Secondary Injury in Younger Athletes After Anterior Cruciate Ligament Reconstruction: A Systematic Review and Meta-analysis. Am J Sports Med. 2016 Jul;44(7):1861-76.

4. Mai HT, Chun DS, Schneider AD, Erickson BJ, Freshman RD, Kester B, Verma NN, Hsu WK. Performance-Based Outcomes After Anterior Cruciate Ligament Reconstruction in Professional Athletes Differ Between Sports. Am J Sports Med. 2017 Aug;45(10):2226-2232.

5. Dingenen B, Gokeler A. Optimization of the Return-to-Sport Paradigm After Anterior Cruciate Ligament Reconstruction: A Critical Step Back to Move Forward. Sports Med. 2017 Aug;47(8):1487-1500.

## S24 Groin pain and miotendinopathy in elite athletes

### Luigi Di Filippo^1,2,3^ (fisioanalysis@gmail.com)

#### ^1^General Director FisioAnalysis - Medical and Sport Center, Alessandria, Italy. ^2^Lecturer at University of TorVergata – Rome, Italy. ^3^Vice-President GISPT (Group of Italian Sports Physical Therapist)

Groin pain in athletes is one of the most difficult to treat clinical problems in sports medicine [1].

The reasons are the amount of differential diagnoses, complexity of pathophysiologic causes and the long time of limited sport participation. In order to maximize efficient treatment, thorough diagnostics and a clear therapeutic regimen are crucial [2].

Only 6% of the studies on treatment of athletes with groin pain are of high quality. There was a significant correlation between lower study quality and higher treatment success [3].

Having an adductor-related groin injury doubles the recovery time compared to injuries with no adductor and no abdominal pain. If it is combined with an abdominal-related injury, the recovery time is more than quadrupled.

A high proportion of the injuries were located on the dominant side (68%) [4].

For athletes with long-standing adductor related groin pain there is moderate evidence that active exercises improve treatment success compared with passive treatments, that multimodal treatment with a manual therapy technique shortens the time to return to sports (RTS) compared with active exercises, and that adductor tenotomy improves treatment success over time [5].

Conservative treatment has demonstrated a superior RTS time when compared to surgery, while little difference between the two treatments in the abdominal and adductor groupings in RTS rate and RTS time [6].

**References**

1. Serner A, van Eijck CH, Beumer BR, Hölmich P, Weir A, de Vos RJ. Study quality on groin injury management remains low: a systematic review on treatment of groin pain in athletes. Br J Sports Med. 2015 Jun;49(12):813.

2. Davies AG, Clarke AW, Gilmore J, Wotherspoon M, Connell DA. Review: imaging of groin pain in the athlete. Skeletal Radiol. 2010 Jul;39(7):629-44.

3. Weber MA, Rehnitz C, Ott H, Streich N. Groin pain in athletes. Rofo. 2013 Dec;185(12):1139-48.

4. Hölmich P, Thorborg K, Dehlendorff C, Krogsgaard K, Gluud C. Incidence and clinical presentation of groin injuries in sub-elite male soccer. Br J Sports Med. 2014 Aug;48(16):1245-50.

5. Weir A, Jansen JA, van de Port IG, Van de Sande HB, Tol JL, Backx FJ. Manual or exercise therapy for long-standing adductor-related groin pain: a randomised controlled clinical trial. Man Ther. 2011 Apr;16(2):148-54.

6. King E, Ward J, Small L, Falvey E, Franklyn-Miller A. Athletic groin pain: a systematic review and meta-analysis of surgical versus physical therapy rehabilitation outcomes. Br J Sports Med. 2015 Nov;49(22):1447-51.

## S25 Anterior ankle impingement in sport athlete

### Miriam Rosa (miriam.rosa86@gmail.com)

#### GIS Sport AIFI

Anterior ankle impingement is a very common injury in athletes. This injury is also called “athlete’s ankle” or “footballer’s ankle”. The pain is anterior, anteromedial o anterolateral and it is common after acute ankle sprain, recurrent ankle sprain o microtrauma. Patients decrease their dorsiflexion and change their gait, they are unable to run, squat, walk on inclined superficie or play sports, ADL impairment decrease from moderate to severe. There is tenderness in anterior and anterolater of ankle [10]. Dorsiflexion PROM and a AROM can be evaluated by goniometer, inclinometer, tape in weight bearing or no weight bearing with the same results [2]. If five or more of these conditions are present, there is anterior ankle impingement (sen= .94 + LR=3.76 - LR=.08): pain during activity, anterolater tenderness, swelling, anterolater paid during dorsiflexion, pain during single leg squat, no lateral instability. Additional tests are the anterior draw, Silfverskiöld, dorsiflexion [7, 8]. Functional tests are knee to wall, Y balance test, squat test, single leg squat test and low limb symmetry index [1, 3, 9]. The athlete is the centre of rehabilitation and different factors like injuries characteristics, sociodemographic factors linked physical factors, psychological factors, social/contextual factors and functional performance must be evaluated before returning to sport. Rehabilitation starts immediately after injury to decrease pain, improve local and distal load. The specific knowledge of the athlete's sport is very important.

The road to recovery is not simple. The first phase consists in conservative treatment, even if there are few evidences. In the second phase FT can use corticosteroid injection. After 3-6 months, if the conservative treatment doesn't leed to results, surgery is required. The aims of rehabilitation are improve ROM, strength, endurance and maintaining vascular capacity by manual therapy, mobilization with or without movement, talar stability tape, flexibility static or dynamic, use of theraband, proprioceptive exercise, strength and endurance exercise, sport specific exercise [4, 6]

**References**

1. Arend M, Kalev M, Mäestu J. Weekly Ankle Lunge Test Screening Might Help Prevent Ankle Injuries. Br J Sports Med. 2017, 51(4): 287.

2. Bennell KL, Talbot RC, Wajswelner H, Techovanich W, Kelly DH, Hall AJ. Intra-rater and inter-rater reliability of a weight-bearing lunge measure of ankle dorsiflexion. Aust J Physiother. 1998;44(3):175-180.

3. Choi HS, Shin WS. Validity of the lower extremity functional movement screen in patients with chronic ankle instability. J Phys Ther Sci. 2015 Jun;27(6):1923-7.

4. Collins N, Teys P, Vicenzino B. The initial effects of a Mulligan's mobilization with movement technique on dorsiflexion and pain in subacute ankle sprains. Man Ther. 2004 May;9(2):77-82.

5. Moustafa El-Sayed AM. Arthroscopic treatment of anterolateral impingement of the ankle. J Foot Ankle Surg. 2010 May-Jun;49(3):219-23.

6. Lavery KP, McHale KJ, Rossy WH, Theodore G. Ankle impingement. J Orthop Surg Res. 2016 Sep 9;11(1):97.

7. Liu SH, Nuccion SL, Finerman G. Diagnosis of anterolateral ankle impingement. Comparison between magnetic resonance imaging and clinical examination. Am J Sports Med. 1997 May-Jun;25(3):389-93.

8. Molloy S, Solan MC, Bendall SP. Synovial impingement in the ankle. A new physical sign. J Bone Joint Surg Br. 2003 Apr;85(3):330-3.

9. Shaffer SW, Teyhen DS, Lorenson CL, Warren RL, Koreerat CM, Straseske CA, Childs JD. Y-balance test: a reliability study involving multiple raters. Mil Med. 2013 Nov;178(11):1264-70.

10. Talusan PG, Toy J, Perez JL, Milewski MD, Reach JS Jr. Anterior ankle impingement: diagnosis and treatment. J Am Acad Orthop Surg. 2014 May;22(5):333-9.

## S26 Anterior Ankle Impingement in Sports

### Francesco Lijoi (dr.francesco.lijoi@gmail.com)

#### “Malatesta Novello” Private Hospital, Cesena, Italy

Ankle impingement may be due to a conflict (collision) between soft or bony structures in the peripheral borders of the joint. In the anterior aspect of the ankle impingements can be identified in three different locations: lateral to the third peroneus tendon (antero-lateral impingement), between the third peroneus and the tibialis anterior tendon (central impingement) or medial to the tibialis anterior tendon (anteromedial impingement).

The anterolateral impingement is a soft-tissue impingement. An inversion sprain can cause lesions of the capsule and the synovia with hematoma that can determine hypertrophic fibrous tissue. Repetitive movements or inadequate rehabilitation can induce the formation of a hypertrophic synovial tissue that can create impingement.

The central impingement is typically a bony impingement. Osteophytes on the anterior edge of tibia and talus can impinge in dorsiflexion producing pain. It has been demonstrated that these osteophytes are due to direct traumas on the anterior aspect of the ankle with ossification of perichondral and periosteal membranes [1]. This condition is commonlys known as the “footballer ankle”.

The anteromedial impingement is also a bony impingement. It is due to an osteophytes formation on the anterior edge of medial malleolus and the medial aspect of the talus at the junction between cartilage and bone. Repetitive collisions of these areas during inversion sprains have the effect to produce reparative tissue formation and subsequent ossification of it causing these types of osteophyte.

The diagnosis of the soft anterior ankle impingement is clinical, while it is both clinical and radiographic for the bony impingements. Direct digital palpation of the site of impingement (lateral, central or medial) causes pain, lateral xray of the ankle is diagnostic for the central impingement, a medial oblique view of the ankle can show the presence of medial osteophytes in case of anteromedial impingement [2]. For the diagnosis of a soft anterolateral impingement only the clinical history and the clinical examination are helpful: MRI is not diagnostic but can only rule out other pathologies [3]. Sometimes an intraarticular carbo-test can be helpful in the diagnosis of this kind of soft tissue impingement.

Surgical treatment is performed after a minimum six- month period of physical, manual, infiltrative therapy and eventually orthotic procedures. Currently the arthroscopic procedures have been demonstrated to have less complications, better results and shorter times of recovery than open surgery [4]. The arthroscopic treatment of the anterolateral impingement allows good and excellent results in about 90% of the patients, but the presence of chondral lesions and instability that causes new recurrent inversion sprains have negative influence on the final result [5]. The worse predictive factors for the final result of the bony impingement are not the location or the dimension of the osteophytes [6] but the degree of the degenerative arthritic changes in the joint: at two years follow-up good and excellent results are 90% in the patients without joint space narrowing, only 50% in the others [7].

**References**

1. Tol JL, Verheyen CP, van Dijk CN. Arthroscopic treatment of anterior impingement in the ankle. J Bone Joint Surg Br. 2001 Jan;83(1):9-13.

2. van Dijk CN, Wessel RN, Tol JL, Maas M. Oblique radiograph for the detection of bone spurs in anterior ankle impingement. Skeletal Radiol. 2002 Apr;31(4):214-21.

3. Donovan A, Rosenberg ZS. MRI of ankle and lateral hindfoot impingement syndromes. AJR Am J Roentgenol. 2010 Sep;195(3):595-604.

4. Niek van Dijk C. Anterior and posterior ankle impingement. Foot Ankle Clin. 2006 Sep;11(3):663-83.

5. Urgüden M, Söyüncü Y, Ozdemir H, Sekban H, Akyildiz FF, Aydin AT. Arthroscopic treatment of anterolateral soft tissue impingement of the ankle: evaluation of factors affecting outcome. Arthroscopy. 2005 Mar;21(3):317-22.

6. Moon JS, Lee K, Lee HS, Lee WC. Cartilage lesions in anterior bony impingement of the ankle. Arthroscopy. 2010 Jul;26(7):984-9.

7. van Dijk CN, Tol JL, Verheyen CC. A prospective study of prognostic factors concerning the outcome of arthroscopic surgery for anterior ankle impingement. Am J Sports Med. 1997 Nov-Dec;25(6):737-45.

## S27 Effects of exercise on neural plasticity in people with cognitive impairments

### Matteo Paci (matteo.paci@applicazione.it)

#### Unit of Functional Rehabilitation, Azienda USL Toscana Centro, Prato, Italy

Several studies demonstrated that physical activity has positive effects from a biological, functional, psychological, emotional, and social point of view [1, 2].

In order to study the influence of physical activity and environmental stimuli on the neural plasticity and behavior, the classic model is the environmental enrichment (EE), defined as “a combination of inanimate and social complex stimuli” [3]. The EE facilitates exploration, cognitive activity, social interaction, and active physical exercise in animal models [3]. The EE also influences the expression of several factors considered essential to brain plasticity, including the Brain-derived neurotrophic factor (BDNF), a neurotrophin particularly relevant to neuroplasticity [4].

In a recent review, Zoladz and Pilc [5] concluded that physical exercise could be able to facilitate the activation of BDNF in some regions of the brain, and that such facilitation, induced by the exercise, could play a role in increasing the cognitive functions.

In elderly rats, Bherer et al. [2] showed that physical activity induces angiogenesis, synaptogenesis and neurogenesis in their hippocampus. In humans, brain-imaging studies and brain electrophysiological measurements, in addition to angiogenesis, synaptogenesis and neurogenesis, reported that physical exercise has structural and functional effects, providing transient and permanent changes in brain aging [2].

Sofi et al. [6] showed that elderly subjects, who performed both high and moderate baseline levels of physical activity, were significantly protected against cognitive decline at the follow-up. In this meta-analysis [6], however, only the Mini Mental State Examination was used as outcome measure, The review of Angevaren et al. [7] reported effects of exercise in healthy subjects on motor function and auditory attention. Moderate effects were also observed for speed and visual attention. On the other hand, other cognitive functions do not seem to be influenced by the exercise.

Finally, Sumic et al. [8] showed that personalized and age-adjusted physical activity can reduce the risk of cognitive decline of 88% even in very elderly people (aged over 85 years).

Effects on cognitive functions have also been identified for patients with Mild Cognitive Impairment (MCI) [9] and for patients with dementia [10].

Many pyramidal and extrapyramidal motor impairments affect a substantial portion of patients with dementia, even at an early stage of the disease, and progressively worsen along with cognitive impairment [11].

MCI may be associated with motor impairment, maily in balance and deambulation [12], sometimes in a subclinical phase [13]; some authors suggested that the presence of motor impairment may be an early indicator of a cognitive disorder, since motor impairment may precede the onset of cognitive impairment for dementia by a decade and longer [11, 14].

**References**

1. Lemura LM, von Duvillard SP, Mookerjee S. The effects of physical training of functional capacity in adults. Ages 46 to 90: a meta-analysis. J Sports Med Phys Fitness. 2000 Mar;40(1):1-10.

2. Bherer L, Erickson KI, Liu-Ambrose T. A review of the effects of physical activity and exercise on cognitive and brain functions in older adults. J Aging Res. 2013;2013:657508.

3. Sale A, Berardi N, Maffei L. Enrich the environment to empower the brain. Trends Neurosci. 2009 Apr;32(4):233-9.

4. Baroncelli L, Braschi C, Spolidoro M, Begenisic T, Sale A, Maffei L. Nurturing brain plasticity: impact of environmental enrichment. Cell Death Differ. 2010 Jul;17(7):1092-103.

5. Zoladz JA, Pilc A. The effect of physical activity on the brain derived neurotrophic factor: from animal to human studies. J Physiol Pharmacol. 2010 Oct;61(5):533-41.

6. Sofi F, Valecchi D, Bacci D, Abbate R, Gensini GF, Casini A, Macchi C. Physical activity and risk of cognitive decline: a meta-analysis of prospective studies. J Intern Med. 2011 Jan;269(1):107-17.

7. Angevaren M, Aufdemkampe G, Verhaar HJ, Aleman A, Vanhees L. Physical activity and enhanced fitness to improve cognitive function in older people without known cognitive impairment. Cochrane Database Syst Rev. 2008 Jul 16;(3):CD005381.

8. Sumic A, Michael YL, Carlson NE, Howieson DB, Kaye JA. Physical activity and the risk of dementia in oldest old. J Aging Health. 2007 Apr;19(2):242-59.

9. Zheng G, Xia R, Zhou W, Tao J, Chen L. Aerobic exercise ameliorates cognitive function in older adults with mild cognitive impairment: a systematic review and meta-analysis of randomised controlled trials. Br J Sports Med. 2016 Apr 19.

10. Forbes D, Thiessen EJ, Blake CM, Forbes SC, Forbes S. Exercise programs for people with dementia. Cochrane Database Syst Rev. 2013 Dec 4;(12):CD006489.

11. Albers MW, Gilmore GC, Kaye J, Murphy C, Wingfield A, Bennett DA, Boxer AL, Buchman AS, Cruickshanks KJ, Devanand DP, Duffy CJ, Gall CM, Gates GA, Granholm AC, Hensch T, Holtzer R, Hyman BT, Lin FR, McKee AC, Morris JC, Petersen RC, Silbert LC, Struble RG, Trojanowski JQ, Verghese J, Wilson DA, Xu S, Zhang LI. At the interface of sensory and motor dysfunctions and Alzheimer's disease. Alzheimers Dement. 2015 Jan;11(1):70-98.

12. Bahureksa L, Najafi B, Saleh A, Sabbagh M, Coon D, Mohler MJ, Schwenk M. The Impact of Mild Cognitive Impairment on Gait and Balance: A Systematic Review and Meta-Analysis of Studies Using Instrumented Assessment. Gerontology. 2017;63(1):67-83.

13. Kueper JK, Speechley M, Lingum NR, Montero-Odasso M. Motor function and incident dementia: a systematic review and meta-analysis. Age Ageing. 2017 Sep 1;46(5):729-738.

14. Montero-Odasso M, Oteng-Amoako A, Speechley M, Gopaul K, Beauchet O, Annweiler C, Muir-Hunter SW. The motor signature of mild cognitive impairment: results from the gait and brain study. J Gerontol A Biol Sci Med Sci. 2014 Nov;69(11):1415-21.

## S28 Outcome measures in patients with cognitive impairment

### Leonardo Pellicciari (leonardo.pellicciari@gmail.com)

#### Unit of Functional Rehabilitation, Azienda USL Toscana Centro, Empoli (FI), Italy

In patients with cognitive impairment (CI), to have suitable measurement tools plays a fundamental role because their prevalence will drastically increase in the next few years [1], and because these patients are generally excluded in randomized clinical trials (RCTs), due to the lack of appropriate measuring instruments. Therefore, the RCTs external validity is missing. Recently, a call for inclusion [2] was proposed to encourage researchers to include these patients in RCTs. Then, researchers need to have tools with robust psychometric properties even in patients with CI.

Considering the Performance-based Measures (specifically, Timed Up & Go [TUG], Chair Rise Test [CRT], Figure of Eight Walk Test [F8W], Frailty and Injuries: Cooperative Studies of Intervention Techniques [FICSIT-4], dynamometer, and 6 Minute Walk test [6MWT]), reliability was assessed in 58 subjects with CI [3]. Regarding the intra-observer reliability, authors obtained an Intraclass Correlation Coefficient (ICC) for the TUG, dynamometer, and F8W that recommend their use in single subject measurements (ICC> 0.90); intra-rater reliability values (0.70<ICC<0.90) for FICSIT-4, CRT, and 6MWT suggest their use in groups measuring. Considering the measurement error, the Minimal Detectable Change (MDC) values of TUG corresponded to about 66% of the total average score; changes below MDC have no clinical relevance. A systematic review [4] reported that only one of 16 study showed a greater post-intervention improvement than MDC value for TUG. This finding suggests that improvements over MDC are hardly achievable in clinical practice; therefore, these tests are unsuitable to quantify the effects of treatment within this population.

Therefore, Bossers et al. [5] proposed a performance-based measure that is specifically designed for patients with CI. The Groeningen Meander Walking Test is an evolution of the F8W; the itinerary was replaced by a path with curves alternated to right and left. This modification is intended to make the task more intuitive, to require simpler instructions, and to avoid the intersection of the path that may be a critical moment for patient with CI. Intra-rater reliability has been studied in a sample of 42 subjects with CI. The ICC (=0.942) allows measurements on single subjects. Moreover, the MDC (corresponding to a change of 30% of the total time) is less than that of the F8W (equal to 40% of the total time). MDC values are still high, but better than the original test.

The measurement of latent variables requires the subjective judgement of a person, such as the patient (using a Patient Reported Outcome Measures) or such a relative or caregiver (utilising an Observer Reported Outcome Measures [ObsOMs]). Regarding patients with CI, it is not appropriate to consider their judgment, as the psychometric properties could be affected by their pathology. Therefore, it is more appropriate to use the ObsROMs.

In the Italian context, few ObsOMs were validated, such as the Direct Assessment for Dementia Scale [6] and the Alzheimer's Functional Assessment Tool [7], which demonstrated good reliability and construct validity. However, any other psychometric properties considered crucial in selecting an appropriate outcome measure (i.e., content and structural validity) have not been examined. Therefore, not all information are available to select the appropriate tool for patients with CI.

In conclusion, in selection of an appropriate outcome measure, clinicians and researchers have to consider all the psychometric properties; not only the reliability but also the measurement error to understand if the change presented by the patient is real or due to the intrinsic error of the measurement instrument. Finally, in the choice of the assessment scale, clinicians and researchers do not only consider cross-cultural and construct validity, but analyse other psychometric properties, as the content and structural validity.

**References**

1. Mura T, Dartigues JF, Berr C. How many dementia cases in France and Europe? Alternative projections and scenarios 2010-2050. Eur J Neurol. 2010 Feb;17(2):252-9. doi: 10.1111/j.1468-1331.2009.02783.x. Epub 2009 Oct 1.

2. Mundi S, Chaudhry H, Bhandari M. Systematic review on the inclusion of patients with cognitive impairment in hip fracture trials: a missed opportunity? Can J Surg. 2014 Aug;57(4):E141-5.

3. Blankevoort CG, van Heuvelen MJ, Scherder EJ. Reliability of six physical performance tests in older people with dementia. Phys Ther. 2013 Jan;93(1):69-78.

4. Blankevoort CG, van Heuvelen MJ, Boersma F, Luning H, de Jong J, Scherder EJ. Review of effects of physical activity on strength, balance, mobility and ADL performance in elderly subjects with dementia. Dement Geriatr Cogn Disord. 2010;30(5):392-402.

5. Bossers WJ, van der Woude LH, Boersma F, Scherder EJ, van Heuvelen MJ. The Groningen Meander Walking Test: a dynamic walking test for older adults with dementia. Phys Ther. 2014 Feb;94(2):262-72.

6. De Vreese LP, Caffarra P, Savarè R, Cerutti R, Franceschi M, Grossi E; Multicentre Study Group. Functional disability in early Alzheimer's disease – a validation study of the Italian version of the disability assessment for dementia scale. Dement Geriatr Cogn Disord. 2008;25(2):186-94.

7. De Vreese LP, Gomiero T, Uberti M, De Bastiani E, Weger E, Mantesso U, Marangoni A. Functional abilities and cognitive decline in adult and aging intellectual disabilities. Psychometric validation of an Italian version of the Alzheimer's Functional Assessment Tool (AFAST): analysis of its clinical significance with linear statistics and artificial neural networks. J Intellect Disabil Res. 2015 Apr;59(4):370-84.

## S29 Effect of physical exercise on markers of cellular immunosenescence: a systematic review

### Hung Cao Dinh^1,2^, Rose Njemini^1,2^, Ivan Bautmans^1,2,3^

#### ^1^Gerontology department, Vrije Universiteit Brussel, Laarbeeklaan 103, B-1090 Brussels, Belgium; ^2^Frailty in Ageing research department, Vrije Universiteit Brussel, Laarbeeklaan 103, B-1090 Brussels, Belgium; ^3^Geriatrics department, Universitair Ziekenhuis Brussel, Laarbeeklaan 101, B-1090 Brussels, Belgium

##### **Correspondence:** Ivan Bautmans (Ivan.Bautmans@vub.be)

Ageing is characterized by a progressive decline in immune function referred to as immunosenescence (IS), which increases the susceptibility of elderly persons to infection, autoimmune disease, and cancer[1, 2]. With advancing age, there is a manifested decrease of naïve T-cell repertoire with a concomitant accumulation of highly differentiated memory and senescent T-cell phenotypes[3]. There are strong indications that physical exercise in elderly persons may prevent the age-related decline in immune response without significant side effects[4]. Consequently, exercise is being considered as a safe mode of intervention to reduce IS[3, 5, 6]. The aim of this review was to appraise the existing evidence regarding the impact of exercise on surface markers of cellular IS in either young and old humans or animals. PubMed and Web of Science were systematically screened and 29 relevant articles in humans or animals were retrieved[7, 8]. We found 2 categories of study: studies reporting the acute effects of exercise and studies showing exercise-induced effects on basal levels. Most of the intervention studies demonstrated that an acute bout of exercise induced increases in senescent, naïve, memory CD4+ and CD8+ T-lymphocytes and significantly elevated apoptotic lymphocytes in peripheral blood. As regards long-term effects, exercise induced higher levels of T-lymphocytes expressing CD28+ in both young and elderly subjects. The findings concerning the influence of exercise on NK cells were sometimes contradictory, some studies showed the increase in NK cell activity while the others recorded the opposite or no effect. Few studies have been conducted so far to investigate the effects of exercise on markers of IS in elderly persons. Exploring data from our ongoing randomized controlled trial Senior Project Intensive Training (SPRINT), we sought to address the effect of strength training at different intensities on the changes of cellular IS in elderly. 100 older women (aged 65 years and over) were randomized to 3 times/weekly training for 6 weeks at either intensive strength training (IST, n=31), strength endurance training (SET, n=33), or control (CON, n=36). The exercise protocols for the IST and SET intervention groups were designed to be approximately equal in volume (% one-repetition maximum; the maximum weight that can be moved once over the whole range of movement (1RM) x number of repetitions). The large muscle groups of the participants were trained at 3x10 repetitions at 75% 1RM, 2x30 repetitions at 40% 1RM for IST, SET respectively. The CON performed flexibility training consisting of 3 sets of sustained (30 sec) passive, static stretching exercises of the large muscle groups. The surface markers of senescence were determined before and after 6 weeks (24h-48h after the last training) using flow cytometry. Absolute blood counts were measured by a dual platform methodology (flow cytometry and the Cell-Dyn Sapphire hematology analyzer). We report for the first time that 6 weeks of SET decreased significantly the resting percentage and absolute blood count of senescence-prone T-cells in older women. Conceivably, training protocols with many repetitions - at a sufficiently high external resistance - seem to be necessary for the reduction of senescence-prone cells in older persons. We can conclude that exercise has considerable effects on markers of cellular aspects of the immune system. Recent results from our study provide evidence to current cellular concepts indicating that exercise training may have an anti-IS effect. Further research is highly needed to fully elucidate the mechanism of lymphocyte IS following exercise.

**References**

1. Pawelec G. Immunosenescence: impact in the young as well as the old? Mech Ageing Dev. 1999 Apr 1;108(1):1-7.

2. Castle SC. Clinical relevance of age-related immune dysfunction. Clin Infect Dis. 2000 Aug;31(2):578-85.

3. Simpson RJ. Aging, persistent viral infections, and immunosenescence: can exercise "make space"? Exerc Sport Sci Rev. 2011 Jan;39(1):23-33.

4. Chin A Paw MJ, de Jong N, Pallast EG, Kloek GC, Schouten EG, Kok FJ. Immunity in frail elderly: a randomized controlled trial of exercise and enriched foods. Med Sci Sports Exerc. 2000 Dec;32(12):2005-11.

5. Turner JE. Is immunosenescence influenced by our lifetime “dose” of exercise? Biogerontology. 2016 Jun;17(3):581-602.

6. Simpson RJ, Lowder TW, Spielmann G, Bigley AB, LaVoy EC, Kunz H. Exercise and the aging immune system. Ageing Res Rev. 2012 Jul;11(3):404-20.

7. Cao Dinh H, Beyer I, Mets T, Onyema OO, Njemini R, Renmans W, De Waele M, Jochmans K, Vander Meeren S, Bautmans I. Effects of Physical Exercise on Markers of Cellular Immunosenescence: A Systematic Review. Calcif Tissue Int. 2017 Feb;100(2):193-215.

8. Zimmer P, Baumann FT, Bloch W, Zopf EM, Schulz S, Latsch J, Schollmayer F, Shimabukuro-Vornhagen A, von Bergwelt-Baildon M, Schenk A. Impact of a half marathon on cellular immune system, pro-inflammatory cytokine levels, and recovery behavior of breast cancer patients in the aftercare compared to healthy controls. Eur J Haematol. 2016 Feb;96(2):152-9.

## S30 Exercises for motor and functional deficits in people with cognitive impairments

### Matteo Paci^1^, Leonardo Pellicciari^2^

#### ^1^Unit of Functional Rehabilitation, Azienda USL Toscana Centro, Prato, Italy; ^2^Unit of Functional Rehabilitation, Azienda USL Toscana Centro, Empoli (FI), Italy

##### **Correspondence:** Matteo Paci (matteo.paci@applicazione.it)

It has been shown that physical activity reduces the risk of dementia and enhances the cognitive function in people with dementia and cognitively impaired older adults [1]. However, some authors believe that a wide type of exercise programs are not appropriate for people with cognitive impairments [2].

Aerobic training is the most frequently type of exercise used in clinical studies [1], while progressive resistance training and balance training are less extensively studied [3].

In general, combinations of these type of exercise seems to be able to improve balance in people with cognitive impairment [4], activities of daily living both in people with mild cognitive impairment [4] and with dementia [5], and general functional performance in cognitively impaired people [2]. Positive effects of exercises were also found in terms of strength, physical fitness, endurance, and positive behaviour [6, 7].

In people with neurological conditions, (e.g. stroke) results may have a poor external validity, since a number of studies exclude people with cognitive impairment [8].

To perform a program of exercises in old participants with cognitive impairments, it should be taken into account the presence of comorbidity (especially cardiac and respiratory comorbidities), drug therapy, musculoskeletal lesions, hydration, nutrition, sleep, and risk of falling [9]. Additional caution and safety adaptations should also be provided for this type of population [9].

However, some questions remain unanswered [1]. For example, it is not clear which are the optimal frequency, duration, type of cognitive commitment that provide a positive outcome with the exercise. The use of aerobic, strength and balance exercises or a mix of these approaches makes informations very heterogeneous. Finally, it is unclear how the changes induced by the exercise vary taking into account the age, disease and presence of brain injury.

Further studies are needed to assess the characteristics of exercises in order to adapt them to people with cognitive deficits and dementia.

**References**

1. Voss MW, Vivar C, Kramer AF, van Praag H. Bridging animal and human models of exercise-induced brain plasticity. Trends Cogn Sci. 2013 Oct;17(10):525-44.

2. Hauer K, Ullrich P, Dutzi I, Beurskens R, Kern S, Bauer J, Schwenk M. Effects of Standardized Home Training in Patients with Cognitive Impairment following Geriatric Rehabilitation: A Randomized Controlled Pilot Study. Gerontology. 2017;63(6):495-506.

3. Fiatarone Singh MA, Gates N, Saigal N, Wilson GC, Meiklejohn J, Brodaty H, Wen W, Singh N, Baune BT, Suo C, Baker MK, Foroughi N, Wang Y, Sachdev PS, Valenzuela M. The Study of Mental and Resistance Training (SMART) study—resistance training and/or cognitive training in mild cognitive impairment: a randomized, double-blind, double-sham controlled trial. J Am Med Dir Assoc. 2014 Dec;15(12):873-80.

4. Lewis M, Peiris CL, Shields N. Long-term home and community-based exercise programs improve function in community-dwelling older people with cognitive impairment: a systematic review. J Physiother. 2017 Jan;63(1):23-29.

5. Forbes D, Thiessen EJ, Blake CM, Forbes SC, Forbes S. Exercise programs for people with dementia. Cochrane Database Syst Rev. 2013 Dec 4;(12):CD006489.

6. Heyn P, Abreu BC, Ottenbacher KJ. The effects of exercise training on elderly persons with cognitive impairment and dementia: a meta-analysis. Arch Phys Med Rehabil. 2004 Oct;85(10):1694-704.

7. Heyn PC, Johnson KE, Kramer AF. Endurance and strength training outcomes on cognitively impaired and cognitively intact older adults: a meta-analysis. J Nutr Health Aging. 2008 Jun-Jul;12(6):401-9.

8. Kafri M, Dickstein R. External validity of post-stroke interventional gait rehabilitation studies. Top Stroke Rehabil. 2017 Jan;24(1):61-67.

9. Montero-Fernández N, Serra-Rexach JA. Role of exercise on sarcopenia in the elderly. Eur J Phys Rehabil Med. 2013 Feb;49(1):131-43.

## S31 Neurocognitive rehabilitation and a new paradigm: the “Comparison Between Actions”: a means to learn, know and for the qualitative recovery of action

### Franca Pantè, Carlo Perfetti

#### **Correspondence:** Franca Pantè (franca.pante@gmail.com)

The Neurocognitive Rehabilitation Theory (NCR) according to C. Perfetti could be included in the "Science of Narrative Medicine", one of the three "circles" of Evidence-Based Medicine. According to the NCR, the quality of recovery is determined by the activation of cognitive processes and by the modality of their activation. Furthermore, it states that the action of creating knowledge activates those plastic processes, that represent an instrument to reorganize the injured system. The commitment to know, with all its components (pedagogical and biological), is essential to modify the central nervous system and the organization of the whole body.

The study of knowledge and its repercussion on the therapeutic exercise has gone through different stages.

After the first phase, generically connected to the study of cognitive processes, which around 1970 were called “Superior Cortical Functions”, the studies continued with reference to Maturana, Varela and Morin works, which significantly modified both the theory and the rehabilitation exercise. One of their precepts was: “know the knowledge”. Future studies underlined that it was no longer sufficient to study only how the patient knows (profile and reasoning), but it was also necessary to investigate how the subject “lives the knowledge”. Therefore, in 2001 two research projects were proposed: "Living the knowledge" and "Talking With the Patient", aimed to investigate the point of view of the “subject who knows”, to understand which processes and modifications are involved in knowing and to penetrate on what the subject thinks and feels while he/she is knowing.

The projects investigate the patient's conscious experience, underlines the importance of the "*first person descriptions*", in all phases of the rehabilitative intervention. The comprehension of the language of the patient about what and how he feels his body, together with the therapist’s third person observation become crucial to formulate new hypotheses about a more complex interpretation of pathology (motor, sensitive, cognitive and emotional aspects). In order to verify/falsify such hypotheses the physical therapist should invent new exercises. These projects led to significant improvements of results in recovery of patients’ skills.

In 2009 another problem was addressed: the organizational autonomy of the patient who was in some cases excessively dependent on the rehabilitation set. A critical rereading of the instruments was carried out: the therapist's verbal instructions (used by the therapist and the patient as a substitution of his own mental operations), and the role of "motor image", introduced in neurocognitive rehabilitation since 1996. The motor image turned out to be too specific and partial, too far for the patient from the real action. The patient cannot make an aware "immediate comparison" between the representation of the exercise experience (a "map") and its meaning within the real action ("the territory").

**References**

1. Bateson G. Mente e Natura. Adelfi Milano. 1984

2. Gentner D, Schmidt LA. Analogical Learning and Reasoning in: Oxford Handbook of Cognitive Psycology,Oxford University Press, New York. 2013

3. Lurija AR. Le funzioni corticali superiori. Giunti Firenze Perfetti C. La rieducazione motoria dell’emiplegico, Ghedini, Milano 1979

4. Pantè F, Rizzello C, Zernitz M. Il confronto: un nuovo strumento: Prima parte: L’osservazione Riabilitazione Neurocognitiva. 2012;8(2):120-131

5. Pantè F. Il dolore come problema riabilitativo: dall’osservazione all’esercizio, Riabilitazione Neurocognitiva. 2007(3);2:93 -101

6. Perfetti C. Immagine motoria, rappresentazione mentale ed esercizio terapeutico, Riabilitazione cognitiva 2000.

7. Perfetti C. Il linguaggio della riabilitazione. “Parlare col malato”. Un percorso di studio. Riabilitazione Neurocognitiva. 2008;4(3):203-234

8. Perfetti C. La didattica del reale. Riabilitazione Neurocognitiva. 2011;7(1):10-36

9. Perfetti C, Pantè F, Rizzello C, Zernitz M. Dall’Esercizio Terapeutico Conoscitivo al Confronto tra zioni. Quali implicazioni riabilitative? Riabilitazione Neurocognitiva. 2014;9(2):117-133.

10. Varela FJ. Un know how per l’etica, Laterza Roma-Bari. 1996

11. Iacono MA. Storie di mondi intermedi, Mefisto edizioni ET. 2016

## S32 Narrative-Based research

### Paola Caruso (paolacaruso@gmail.com)

#### S.I.Fi.R. Società Italiana Fisioterapia e Riabilitazione

In the last years, next to the interest in good clinical decisions made on the basis of EBM, the interest of medicine has developed also for what is unknown and imponderable, for the self, and for the patient's life, for the story of the disease. This approach is also known as patient-centered work, conscious practice, care focused on the relationship, or narrative medicine.

For more than a decade, the Italian National Institute of Health (Istituto Superiore di Sanità- ISS) and the National Center for Rare Diseases (Centro Nazionale delle Malattie Rare-CNMR) have promoted the use of narrative medicine in a multidisciplinary approach. To promote the integration between Narrative Based Medicine (NBM) and EBM, ISS published the “Guidelines for the use of narrative medicine”.

Narrative medicine can be a useful tool as it offers the opportunity to think and deal with concepts like “Disease” as a biological fact (ie what are the clinical knowledge of the disease), “Sickness” as social perception of the disease (economic, political, institutional), "Illness" as the patient's subjective experience. So we have to consider Narrative Medicine as a methodological tool for the evaluation of patients, useful for choosing and programming therapeutic interventions and outcomes; it is an approach that influences the compliance, being the basis of communication, mutual understanding and establishing a relationship of trust.

Today scientific research is carried out in two forms: quantitative approach or qualitative approach. Quantitative approach is about reducing to numbers and statistics, find final scores from measurement scales and it allowes to calculate the changes with statistical analysis. It gives answers about effectiveness, causes, prediction, prognosis, diagnosis, cost / benefit and description. The qualitative approach focuses on the "process" of carrying out an action rather than the final "product": research focuses on «how», «why» and «when» things happen and not only the fact that they occur. It gives answers about exploration, description, explication, reasoning and it helps to develop of theories.

The construction of narrations in clinical practice leads to the union between Narrative Medicine and Evidence Medicine in order to create a clinical practice based on scientific evidence, but also on the values to which the patient refers and to his preferences.

The qualitative dimension allow to get important information about the patient's way to live the disease (illness), his feelings, objectives, critical issues and fears.

It is necessary that the physiotherapist learns to use the dialogue with the person as well as to “analyse” the pathology, so that he can collect data that will allow him to deepen clinical reasoning from the point of view of cognitive, sensory, and emotional components.

Important tools for the physiotherapists are the data collection ( for the construction of a personalized treatment plan), the analysis of perceived quality, and the patient's diary, in which he has to write everyday about what he felt and experienced during the rehabilitation session and what has changed in his ways of experiencing the body in his life outside the rehabilitation context. That's important to understand what he learned and therefore how he modified his behaviour and how he was able to integrate the experiences of the exercises in his life.

**References**

1. Charon R. Narrative and medicine. N Engl J Med. 2004 Feb 26;350(9):862-4.

2. Charon R, Wyer P; NEBM Working Group. Narrative evidence based medicine. Lancet. 2008 Jan 26;371(9609):296-7.

3. Giarelli G. Storie di cura: medicina narrativa e medicina delle evidenze: l'integrazione possibile. F. Angeli, 2005.

4. Bert G. Medicina narrativa: storie e parole nella relazione di cura. Il Pensiero Scientifico Editore, 2007.

5. Gibson BE, Martin DK. Qualitative research and evidence-based physiotherapy practice. Physiotherapy, 2003, 89.6: 350-358.

6. Istituto Superiore di Sanità. Linee di indirizzo per l’utilizzo della Medicina Narrativa in ambito clinico-assistenziale, per le malattie rare e cronico-degenerative. Rome: I Quaderni di Medicina” de Il Sole24Ore Sanità (Allegato al N. 7, 24 feb.-2mar. 2015), 2015.

## S33 Exercise on coding and use in the check list: ICF on simulated cases

### Orazio Meli, Maria Elena Tondinelli, Marina Ciriello, Franca Tirinelli

#### Società Italiana Fisioterapia e Riabilitazione – S.I.Fi.R

##### **Correspondence:** Orazio Meli (meli@airpg.it)

By its very nature, the ICF Classification is a tool that requires the involvement of different disciplines and skills, and must guarantee the multidimensional bio-psycho-social approach to disability, a work strategy that characterizes the Classification itself. Therefore, in the context of a care process, when the different professionals integrate their skills using the ICF as an analysis tool, they must treat in advance how to organize relations within the working group and how to standardise the support process, with a multidimensional evaluation. This kind of evaluation must guarantee the taking charge identifying a specific methodology, an assumption of responsibility predefining the organisation of the assistance process for each professional involved, a multi-disciplinary and multidimensional path essential to maintain the communication and effective relationship.

These conditions can be ensured by standardizing and structuring an organized and shared path regarding the "evaluation survey" of all the professionals involved.

All professionals have to follow the phases of the evaluation process, consisting at first in the collection of information through anamnesis, instrumental and laboratory survey data, opinions of other experts, any other type of health documentation, specific functional assessment for own professional competence also using tools such as tests, scales of measurement and others in order to objectify, where possible, his/her observations; than interpretation of data and identification of problems, assumptions for overcoming the problems, definition of objectives and intermediate and final verification.

Because of its "universal" language, the ICF Classification is adopted by each professional, allowing to enhance the effectiveness of communication and of the relationship between the members of the working group.

The adoption of a single and shared model also provides useful guidance for the use of a single reference method for the management of the care process, characterizing and expressing the assumption of responsibility of each through the formalization of their own specific contribution.

We propose the use of a new "vocabulary" in the beta phase, which is the International Classification of Health Interventions (ICHI) project in progress under the WHO, third reference classification after ICD and ICF. Sharing languages such as ICF and ICHI classification allows to increase the possibility to describe changes in functions in a standardised way, allows to share an interpretative language, to evaluate the effectiveness of the interventions, to scientifically validate the evaluation and assistance process, and to make a strong contribution to feeding the flow of information that can be exported to different areas.

**References**

1. Ed. Erickson - Classificazione Internazionale del Funzionamento, della Disabilità e della Salute – 2001.

2. International Journal of Environmental Research and Public Health - Use of a New International Classification of Health Interventions for Capturing Information on Health Interventions Relevant to People with Disabilities – 2018 - Nicola Fortune, Richard Madden and Ann-Helene Almborg

## S34 Use of the ICF in the stroke patients rehabilitation

### Marina Ciriello (marina.ciriello@ospedalideicolli.it)

#### Consigliera SIFiR - Società Italiana Fisioterapia e Riabilitazione

Many studies have shown that diagnosis alone is not absolutely sufficient to identify needs, the level of care and the clinical consequences in terms of functionality. To prepare all information necessary for a health and rehabilitation intervention on the whole person according to an integrated, holistic and biopsychosocial health model, the implementation of the ICF becomes extremely useful.

Concerning the Stroke, several studies have shown the usefulness of the ICF in the care of patients: it allows a more complete view of the circumstances that favour or hinder the rehabilitation process after a stroke (Silva SM , 2015), it facilitates the implementation of local services and the structuring of the multidisciplinary team (Tempest S , 2006), clarifying the roles within the team and facilitating clinical reasoning (Tempest S 2006, 2013 ).

An ideal measuring system that satisfies all the clinometric criteria does not exist, as pointed out by Harrison in 2013, so the use of the ICF becomes even more important. In the clinical practice the functional clinical evaluation of stroke patients is divided into cognitive function, communicative function, motor and sensory impairments, disability and quality of life, seeing them as separate aspects.

On the other hand, the purpose of the rehabilitative interventions is to promote the recovery of the skills compromised by the stroke, to promote social reintegration, to use the residual operational skills, to define the prognosis and the related needs in order to facilitate the early reorganization of the patient's activity and satisfy his request for assistance, as underlined by the Italian Guidelines (LG SPREAD). The ICF facilitates this overview of the patient's needs, as highlighted by the literature.

Some problems have emerged from the use of ICF in clinical practice, especially whether it is useful or not to find correlations between the qualifiers and universally shared evaluation scales, for at least some domains. However, ICF qualifiers could be also considered not evaluative, but descriptive. This means that they are not supposed to evaluate, but to help the description.

The use of ICF in clinical practice has shown that replacing the assessment tools with the ICF is improper, but its usefulness is unquestionable for the assessment and is completed by providing an overview of the Stroke patient's needs

**References**

1. Goljar N, Burger H, Vidmar G, Leonardi M, Marincek C. Measuring patterns of disability using the International Classification of Functioning, Disability and Health in the post-acute stroke rehabilitation setting. J Rehabil Med. 2011 Jun;43(7):590-601.

2. Harrison JK, McArthur KS, Quinn TJ. Assessment scales in stroke: clinimetric and clinical considerations. Clin Interv Aging. 2013;8:201-11.

3. Linee Guida SPREAD Stroke Prevention and Educational Awareness Diffusion. 2017

4. Riberto M, Lopes KA, Chiappetta LM, Lourenção MI, Battistella LR. The use of the comprehensive International Classification of Functioning, Disability and Health core set for stroke for chronic outpatients in three Brazilian rehabilitation facilities. Disabil Rehabil. 2013 Mar;35(5):367-74.

5. Silva SM, Corrêa FI, Faria CD, Buchalla CM, Silva PF, Corrêa JC. Evaluation of post-stroke functionality based on the International Classification of Functioning, Disability, and Health: a proposal for use of assessment tools. J Phys Ther Sci. 2015 Jun;27(6):1665-70.

6. Tempest S, McIntyre A. Using the ICF to clarify team roles and demonstrate clinical reasoning in stroke rehabilitation. Disabil Rehabil. 2006 May 30;28(10):663-7.

7. Tempest S, Harries P, Kilbride C, De Souza L. Enhanced clarity and holism: the outcome of implementing the ICF with an acute stroke multidisciplinary team in England. Disabil Rehabil. 2013;35(22):1921-5.

## S35 Scientific findings dissemination among truthfulness and traps

### Marco Baccini (marco.baccini@unifi.it)

#### Azienda Ospedaliero-Universitaria Careggi and School of Physiotherapy, Florence University

Some years ago, a renowned epidemiologist claimed that most published research findings are false, despite formal statistical significance (i.e. p< 0.05) [1]. Based on a statistical approach similar to the one used in diagnostic tests, he demonstrated that only adequately powered RCTs with little bias and confirmatory meta-analyses of good quality RCTs produce findings that are more likely true than false. In addition to the prior (pre-study) probability of the studied relationship being true, main determinants are the statistical errors (type I and II) and the presence of bias. Most of published trials in the rehabilitation field are underpowered and with high or uncertain risk of bias, so we may doubt about the truthfulness of many reported findings. Unfortunately, we cannot be more confident that a research finding is true when it was published in high Impact Factor (IF) journal, because the IF is a measure of the *importance* of a journal in a specific field of research, rather than of the *quality* of a specific journal or article. Moreover, a strong positive correlation was found between the article retraction rate and the journal IF [2], indicating that false findings are not rare also in high IF journals. In fact, an article is most often retracted when serious doubts emerge about the faithfulness or even the honesty of the study. Traps also come from the press coverage of health issues, since mass media strongly favour initial studies that are published in renowned journals, whose findings are often either refuted or strongly attenuated by subsequent research [3].

For some years now, further serious threats come also from the so-called “predatory” open access journals, i.e. by publications that are suspected of taking large fees by the authors without providing robust editorial services. Beall [4] started publishing a list of potentially predatory journals in 2008, and kept updating its list with the addition of new journals and publishers until he removed it in January 2017. The lack of an accurate peer review process seems to be the key feature of predatory journals, possibly resulting in publishing low quality articles. Some evidence of that has been produced. Bohannon sent a fake drug paper reporting a clearly flawed experiment with worthless results to open access journals in the Beall’s list: surprisingly, the vast majority of them accepted the article, often after superficial or no peer review [5]. Predatory journals are also more likely to recruit fake editors: about 1/3 of them accepted the application for editor of a fictitious Polish scientist named Anna O. Szust (“oszust” is the Polish word for “fraud”), who bragged about fake scientific degrees and extended research interests, but no articles in indexed journals [6].

The number of papers published in such journals grew from about 53,000 papers in 2010 up to over 400,000 in 2014 [7], increasing the risk of disseminating unsound scientific findings. The research in the rehabilitation field is not free from the phenomenon [8]: fifty-six rehabilitation journals are included in the Beall’s list, seven being also indexed in PubMed. These journals had published 5610 articles up to October 2016. A brief analysis of the duration of the peer review process (estimated by the time interval from submission to acceptance) reveals that some of these journals accepted for publication a large number of papers by 2 or even 1 week since submission.

Data presented show that several traps may lead to the dissemination of unsound research findings, which might be translated into the physiotherapy practice. How can we improve the situation? First, improving the quality of research conducting and reporting is strongly recommended. Spreading the practice of trial pre-registration and following the proper guidelines (e.g., CONSORT, TIDieR) are all actions that move in that direction. Likely, increasing the control for indexing in biomedical databases is also needed. However, it is high time that health prefessionals increase their scholarly publishing literacy and acquire further competences in critical appraisal of published research.

**References**

1. Ioannidis JP. Why most published research findings are false. Plos Med. 2005 Aug;2(8):e124

2. Fang FC, Casadevall A. Retracted science and the retraction index. Infec Immun. 2011 Oct;79(10)3855-9.

3. Gonon F, Konsman JP, Cohen D, Boraud T.Why most biomedical findings echoed by newspapers turn out to be false: the case of attention deficit hyperactivity disorder. PLoS One. 2012;7(9):e44275.

4. Beall J. Criteria for determining predatory open-access publishers. 2nd edition. Denver, CO: Scholarly Open Access; 2012.

5. Bohannon J. Who’s afraid of peer review? Science. 2013 Oct 4;342(6154):60-5.

6. Sorokowski P, Kulczycki E, Sorokowska A, Pisanski. Predatory journals recruit fake editor. Nature. 2017 Mar 22;543(7646):481-483.

7. Shen C1, Björk BC. 'Predatory' open access: a longitudinal study of article volumes and market characteristics. BMC Med. 2015 Oct 1;13:230.

8. Manca A, Martinez G, Cugusi L, Dragone D, Mercuro G, Deriu F. Predatory Open Access in Rehabilitation. Arch Phys Med Rehabil. 2017 May;98(5):1051-1056.

## S36 Use of molecular markers in the evaluation of the therapeutic exercise’s effectiveness

### Maria Consiglia Calabrese (mac.calabrese@virgilio.it)

**Background and Objective:** Exercise-based cardiac rehabilitation (CR) is effectively used as an adjuvant therapy in a number of cardiovascular diseases (CVDs), including chronic heart failure (CHF), and it is recommended by the American and European Society of Cardiology guidelines. Exercise training (ET) increases physical and functional capacity, ameliorates quality of life, decreases symptoms (fatigue and dyspnoea) and, more importantly, reduces the incidence of acute cardiac events, mortality and hospitalization rates. Recently, it has been shown that a moderate exercise is able to induce the recovery of antioxidant defences, whose expression changes with aging and during CVDs. Despite the number of evidences underling the CR-associated cardiovascular protection, CR itself is still an underused medical resource and the mechanisms accounting for such benefits are not completely elucidated yet. The present study investigates whether a well-structured rehabilitation program of 4 weeks can to modify systemic antioxidant potential in HF patients, and examins the mechanisms by which exercise improves cardiovascular function.

**Materials and Methods:** 50 subjects with diagnosis of CHF (NYHA class II and III) were recruited from the Cardiac Rehabilitation Unit of “San Giovanni di Dio e Ruggi d’Aragona” Hospital in Salerno. On admission, patients underwent case history recording, clinical examination, electrocardiogram, chest X-Ray, echocardiogram, cardiopulmonary stress test and a 6-minute walking test, blood sample collection for routinary and experimental analysis. The CR program consisted in ET of 30' on cycloergometer, respiratory gymnastic along with educational meetings, for a meantime of 4 weeks. Blood samples were collected at baseline and at the end of CR, and oxidants (TBARS and 8-hydroxy-2-deoxyguanosine), antioxidants (catalase, Cat, and superoxide dismutase, SOD), and bioavailability of nitric oxide (NO) were measured in patients’ sera, whereas Sirtuin 1 (Sirt1) activity was quantified in patients’ lymphocytes. Human endothelial cells (ECs), exposed or not to H2O2-oxidative stress, were conditioned with patients’ sera, and cellular redox state and senescence were evaluated. A similar approach in an animal model of post-ischemic HF was used to confirm and assess the effect of exercise on senescence. Finally, inhibitors of Sirt1(EX-527) and Cat (ATZ) activities were used to investigate the roles of these proteins in modulating endothelial cell senescence.

**Results:** The results demonstrated that CR stimulated an increase of oxidants with concomitant rise of Sirt1 activity, antioxidants and NO bioavailability. Moreover, CR prevented the ECs senescence via Sirt1 and Cat activation while the inhibition of these enzymes eliminated such effect, both in humans and in the animal model. Lastly, Sirt1 and Cat activities were, respectively, inversely and directly associated with cardiopulmonary stress test duration.

**Conclusion:** Findings suggest that CR triggers cellular adaptations leading to enhance systemic antioxidant effectiveness. Circulating levels of Sirt1 and Cat activity are suggested to be promising markers for assessing the efficacy of CR program.

**References**

1. Mozaffarian D, Benjamin EJ, Go AS, Arnett DK, Blaha MJ, Cushman M, de Ferranti S, Després JP, Fullerton HJ, Howard VJ, Huffman MD, Judd SE, Kissela BM, Lackland DT, Lichtman JH, Lisabeth LD, Liu S, Mackey RH, Matchar DB, McGuire DK, Mohler ER 3rd, Moy CS, Muntner P, Mussolino ME, Nasir K, Neumar RW, Nichol G, Palaniappan L, Pandey DK, Reeves MJ, Rodriguez CJ, Sorlie PD, Stein J, Towfighi A, Turan TN, Virani SS, Willey JZ, Woo D, Yeh RW, Turner MB; American Heart Association Statistics Committee and Stroke Statistics Subcommittee. Heart disease and stroke statistics--2015 update: a report from the American Heart Association. Circulation. 2015 Jan 27;131(4):e29-322.

2. Lawler PR, Filion KB, Eisenberg MJ. Efficacy of exercise-based cardiac rehabilitation post-myocardial infarction: a systematic review and meta-analysis of randomized controlled trials. Am Heart J. 2011 Oct;162(4):571-584.e2.

3. Schutzer KA, Graves BS. Barriers and motivations to exercise in older adults. Prev Med. 2004 Nov;39(5):1056-61.

4. Rengo G, Leosco D, Zincarelli C, Marchese M, Corbi G, Liccardo D, Filippelli A, Ferrara N, Lisanti MP, Koch WJ, Lymperopoulos A. Adrenal GRK2 lowering is an underlying mechanism for the beneficial sympathetic effects of exercise training in heart failure. Am J Physiol Heart Circ Physiol. 2010 Jun;298(6):H2032-8.

5. Leosco D, Rengo G, Iaccarino G, Golino L, Marchese M, Fortunato F, Zincarelli C, Sanzari E, Ciccarelli M, Galasso G, Altobelli GG, Conti V, Matrone G, Cimini V, Ferrara N, Filippelli A, Koch WJ, Rengo F. Exercise promotes angiogenesis and improves beta-adrenergic receptor signalling in the post-ischaemic failing rat heart. Cardiovasc Res. 2008 May 1;78(2):385-94.

6. Nolte K, Herrmann-Lingen C, Wachter R, Gelbrich G, Düngen HD, Duvinage A, Hoischen N, von Oehsen K, Schwarz S, Hasenfuss G, Halle M, Pieske B, Edelmann F. Effects of exercise training on different quality of life dimensions in heart failure with preserved ejection fraction: the Ex-DHF-P trial. Eur J Prev Cardiol. 2015 May;22(5):582-93.

7. Ismail H, McFarlane JR, Nojoumian AH, Dieberg G, Smart NA. Clinical outcomes and cardiovascular responses to different exercise training intensities in patients with heart failure: a systematic review and meta-analysis. JACC Heart Fail. 2013 Dec;1(6):514-22.

8. Corbi G, Conti V, Russomanno G, Rengo G, Vitulli P, Ciccarelli AL, Filippelli A, Ferrara N. Is physical activity able to modify oxidative damage in cardiovascular aging? Oxid Med Cell Longev. 2012;2012:728547.

9. Meyer P, Gayda M, Juneau M, Nigam A. High-intensity aerobic interval exercise in chronic heart failure. Curr Heart Fail Rep. 2013 Jun;10(2):130-8.

10. Tanno M, Kuno A, Horio Y, Miura T. Emerging beneficial roles of sirtuins in heart failure. Basic Res Cardiol. 2012 Jul;107(4):273.

## S37 Development, validation and first implementation of a biofeedback system for the assessment of the bite force control

### Marco Testa (marco.testa@unige.it)

#### Department of Neuroscience, Rehabilitation, Ophthalmology, Genetics, Maternal and Child Health – University of Genova, Campus of Savona, Savona Italy

**Background and Objective:** The function of mastication requires an accurate bite force and jaw movement control to manipulate and break food of different size, shape and hardness. The jaw and muscles motor control is guaranteed by a complex sensory inflow arising from periodontal receptors and muscle spindles, as well as from mucosal and tongue receptors which contribute to the generation of an effective masticatory pattern and to finely tune bite force and mandible movement, according to size, hardness and shape of the food pieces. Several biomechanical and neuromuscular factors could influence and characterize this individual ability of sensory-motor control. Many neurologic, rheumatologic and dental disorders affect the stomatognatic area and determine impairment of muscles force and/or alteration of the jaw motor control. Although the maximal bite force and the jaw kinematics are widely used and important aspects to describe the function of the masticatory system, there is still a lack of clinical instruments for the assessment of more sophisticated and peculiar motor abilities, like the accuracy of the force output and jaw motion, capable to better characterize the motor control of the jaw. The present research project aims to design and validate a biofeedback system to assess the jaw motor function by the measurement of the individual capacity of modulating the bite force and the jaw movements during specific reach and hold tasks.

**Materials and Methods:** The research project of designing and validating a system for the assessment of the capacity of control of muscles force modulation lasted three years and was organized in order to reach the following objectives: (i) design and characterization/validation of adequate sensors system for bite force recording (ii) design of “reach and hold” software to assess unilateral and bilateral individual capacity to finely modulate the masticatory muscles force output, (iii) development and validation of performance indexes to measure the individual capacity to finely modulate the masticatory force output and (iv) implementation and validation of the bite force control assessment system in pathologic populations.

**Result:** As final output, were successfully designed and validated a prototypal device based on visual feedback, a number of clinical procedures and a system of outcome measures to assess the individual ability to control the delivery of bite force.

**Conclusion:** The system presented acceptable reliability and the performance indices seem capable to describe the individual ability to deliver bite force, opening to the possibility to use this system as support for functional diagnosis. The improvement of the performance indices in following session of the different studies testimonies a motor learning process and encourages the implementation of the system in rehabilitation. Moreover, the bilateral coordination of the bite force was assessed for the first time and this methodology could have a role in the evaluation of pathologies like multiple sclerosis where the interhemispheric coordination is involved.

**References**

1. Testa M, Di Marco A, Pertusio R, Van Roy P, Cattrysse E, Roatta S. A validation study of a new instrument for low cost bite force measurement. J Electromyogr Kinesiol. 2016 Oct;30:243-8.

2. Testa M, Geri T, Gizzi L, Falla D. High-density EMG Reveals Novel Evidence of Altered Masseter Muscle Activity During Symmetrical and Asymmetrical Bilateral Jaw Clenching Tasks in People With Chronic Nonspecific Neck Pain. Clin J Pain. 2017 Feb;33(2):148-159.

3. Testa M, Geri T, Gizzi L, Petzke F, Falla D. Alterations in Masticatory Muscle Activation in People with Persistent Neck Pain Despite the Absence of Orofacial Pain or Temporomandibular Disorders. J Oral Facial Pain Headache. 2015 Fall;29(4):340-8.

4. Testa M, Geri T, Signori A, Roatta S. Visual Feedback of Bilateral Bite Force to Assess Motor Control of the Mandible in Isometric Condition. Motor Control. 2015 Oct;19(4):312-24.

5. Testa M, Rolando M, Roatta S. Control of jaw-clenching forces in dentate subjects. J Orofac Pain. 2011 Summer;25(3):250-60.

## S38 Anatomical and neurophysiological substrates of muscle synergies of the upper limb, after stroke

### Andrea Turolla^1,2^ (andrea.turolla@ospedalesancamillo.net)

#### ^1^Laboratory of Neurorehabilitation Technologies, IRCCS Fondazione Ospedale San Camillo, Venice, Italy; ^2^Department of Neuroscience, University of Sheffield, Sheffield, UK

**Background and Objective:** The treatment of upper limb motor function impairments and associated participation restrictions still represent a challenging therapy target in stroke neurorehabilitation. [1]. Recent evidence showed that virtual reality (VR) is better than conventional physiotherapy for the treatment of upper limb, after stroke. [2-5] Both genetics and neurophysiological factors drive functional recovery and carrying the Val66Met single nucleotide polymorphism (SNP) of the brain derived neurotrophic factor (BDNF) was argued to be a potential determinant of poor motor recovery. [6] Motor control theories postulate that the motor system pools groups of muscles in functional units called muscle synergies, to control voluntary movements. [7,8] A determined number of muscle synergies, which is stable across subjects, but affected by stroke, allows the description of natural motor behaviour. [9] Evidence from animals proposed a subcortical and spinal substrate for muscle synergies. [10] In this series of studies, a virtual reality environment commonly applied in real clinical settings for the treatment of upper limb after stroke, was used as a reference framework to test hypotheses on both the genetics and neurophysiological factors described above.

**Materials and Methods:** Literature review.

**Results:** Two studies explored whether carrying the Val66Met SNP BDNF determines a bad recovery of upper limb motor function and whether different brain morphologies are associated with each genotype, in stroke survivors. Two other studies explored whether muscle synergies are represented in the human brain and whether their representation is affected by stroke. A fifth study explored whether muscle synergies might represent a robust neurophysiological outcome to test differences in efficacy between VR-based treatments and conventional therapy. With regard to genetics, the key findings were that polymorphisms of the BDNF do not determine clinically detectable differences, but brain morphological differences exist, because of the genotypes, with bigger brain areas in carriers of the Val66Met SNP BDNF. Neurophysiological findings showed that muscle synergies are represented in the brain structures of the pyramidal motor system, but their representation extends to brain areas devoted to higher order cognitive functions, after stroke. Finally, it was found that VR-based therapy determines a better functional brain reorganisation around muscle synergies brain seeds, than conventional physiotherapy.

**Conclusion:** More research is needed to determine whether these findings represent reliable modules which can be incorporated within a computational model of neurorehabilitation.

**References**

1. Pomeroy V, Aglioti SM, Mark VW, McFarland D, Stinear C, Wolf SL, Corbetta M, Fitzpatrick SM. Neurological principles and rehabilitation of action disorders: rehabilitation interventions. Neurorehabil Neural Repair. 2011 Jun;25(5 Suppl):33S-43S.

2. Laver KE, George S, Thomas S, Deutsch JE, Crotty M. Virtual reality for stroke rehabilitation. Cochrane Database Syst Rev. 2011 Sep 7;(9):CD008349.

3. Kiper P, Agostini M, Luque-Moreno C, Tonin P, Turolla A. Reinforced feedback in virtual environment for rehabilitation of upper extremity dysfunction after stroke: preliminary data from a randomized controlled trial. Biomed Res Int. 2014;2014:752128.

4. Kiper P, Piron L, Turolla A, Stożek J, Tonin P. The effectiveness of reinforced feedback in virtual environment in the first 12 months after stroke. Neurol Neurochir Pol. 2011 Sep-Oct;45(5):436-44.

5. Luque-Moreno C, Oliva-Pascual-Vaca A, Kiper P, Rodríguez-Blanco C, Agostini M, Turolla A. Virtual Reality to Assess and Treat Lower Extremity Disorders in Post-stroke Patients. Methods Inf Med. 2016;55(1):89-92.

6. Qin L, Jing D, Parauda S, Carmel J, Ratan RR, Lee FS, Cho S. An adaptive role for BDNF Val66Met polymorphism in motor recovery in chronic stroke. J Neurosci. 2014 Feb 12;34(7):2493-502.

7. Cheung VC, Piron L, Agostini M, Silvoni S, Turolla A, Bizzi E. Stability of muscle synergies for voluntary actions after cortical stroke in humans. Proc Natl Acad Sci U S A. 2009 Nov 17;106(46):19563-8.

8. Kiper P, Szczudlik A, Venneri A, Stozek J, Luque-Moreno C, Opara J, Baba A, Agostini M, Turolla A. Computational models and motor learning paradigms: Could they provide insights for neuroplasticity after stroke? An overview. J Neurol Sci. 2016 Oct 15;369:141-148.

9. Cheung VC, Turolla A, Agostini M, Silvoni S, Bennis C, Kasi P, Paganoni S, Bonato P, Bizzi E. Muscle synergy patterns as physiological markers of motor cortical damage. Proc Natl Acad Sci U S A. 2012 Sep 4;109(36):14652-6.

10. Roh J, Cheung VC, Bizzi E. Modules in the brain stem and spinal cord underlying motor behaviors. J Neurophysiol. 2011 Sep;106(3):1363-78.

## S39 Therapeutic Exercise: impairment oriented or task oriented approach? A taxonomy analysis

### Thomas Bowman^1^, Elisa Gervasoni^1^, Michela Agostini^2^, Francesca Marazzini^3^, Susanna Mezzarobba^4^, Daniele Munari^5^, Riccardo Parelli^1^, Elisa Pelosin^6^, Maurizio Petrarca^7^, Paolo Pillastrini^8^, Rita Russo^9^, Cristina Simionato^1^, Andrea Turolla^2^, Davide Cattaneo^1^

#### ^1^IRCCS Fondazione Don Carlo Gnocchi, Centro "Santa Maria Nascente", via Capecelatro 66, MI; ^2^ IRCCS Fondazione Ospedale San Camillo. Via Alberoni, 70, Lido, VE; ^3^ AIAS Milano, via Paolo Mantegazza, 10, 20156 Milano, MI; ^4^ Università degli studi di Trieste, Piazzale Europa, 1, 34127 Trieste TS; ^5^ UOC Neuroriabilitazione, Azienda ospedaliera Universitaria integrata. Verona, VR; ^6^ Dipartimento di Neuroscienze, Riabilitazione, Oftalmologia, Genetica e Scienze Materno Infantili (DINOGMI). Università degli Studi di Genova, Largo P. Daneo 3,16132, Genova; ^7^ San Giovanni Battista –SMOM- Via Morselli 13, Roma; ^8^ Dipartimento di Scienze Biomediche e Neuromotorie (DIBINEM), Università di Bologna, BO; ^9^ UO di Riabilitazione Specialistica, Presidio ospedaliero San Carlo Borromeo, ASST Santi Paolo e Carlo, Milano (MI)

##### **Correspondence:** Davide Cattaneo (dcattaneo@dongnocchi.it)

**Background and Objective:** Rehabilitation is an active and dynamic process through which a disabled person is helped to acquire knowledge and skills in order to maximize their physical, psychological, and social functioning. The aims of this process can be addressed to reduce disability, acquire new skills and strategies to maximize activity and improve participation. Over the last 15-20 years rehabilitation has moved from “professional artistry” to an evidence-based scientific approach and the task-oriented approach (TOA) and impairment-oriented approach (IOA) have been used to provide a framework to identify neurological treatments. [1] TOA assumes that rehabilitation needs to be regarded as a problem-solving process with its own specific focus on activity limitation. [2] The principles of TOA are aimed to ensure challenging and meaningful practice, address important (interfering) changeable impairments, enhance motor capacity through overload and specificity and preserve natural goal-directedness in movement organization. [3] TOA is focused on the task to be learned in which the level of task difficulty follows the person' skills and current level of performance defined as 'Challenge point'. Moreover, the intensity of the treatment has to be appropriate to incur an increase in function. Thus, the variability of activities has to be progressively challenging and varied to maximize transfer to the individual daily living environment. [4] Previous studies have demonstrated that intensive physical rehabilitation paradigms focusing on participation in specific tasks may improve locomotor recovery to a greater extent compared to traditional therapeutic techniques. [5, 6, 7] Conversely, IOA is aimed to improve the condition of an impaired body structure (physiological, anatomical or psychological) focusing on the morphological (peripheral) and neural (central) origin of impairments. One of the main premises of IOA is that inappropriate reduction in both aerobic capacity and muscle strength is a consequence of inactivity secondary to the disease which further translate into impaired functional capacity [8]. Considering this, IOA for strength impairments consists in isolating muscle actions by focusing on individual muscles and maximizing strength and using spared motor units avoiding secondary complications and compensatory patterns. Nowadays substantial evidence shows that progressive resistance training is efficient in improving muscle strength per se, without any changes in balance, functional capacity, mood and quality of life. This less convincing evidence may be improved defining parameters related to specificity, amount, and intensity of training which are still critical factors to facilitate recovery following neurological injury. [5] Nowadays, the comparison of the effects of TOA and IOA has been difficult due to different methodological approaches, the lack of consistency in the assessments and lack of identification of the key components of TOA and IOA that are essential to produce clinical improvements. Comparison between these two approaches can provide fuller understanding of the differential effects of TOA and IOA facilitating the development of effective and tailored treatments.

**Materials and Methods:** In this perspective a taxonomy provides a framework of rehabilitation intervention for consistent identification and labeling of treatments to describe quantify and comparing them in terms of outcome, dose, or intensity of interventions. [9]

**Results:** We develop a taxonomy for interventions and define a core assessment tool to link treatments characteristics with treatment outcomes in neurological conditions.

**Conclusion:** Further studies are needed to apply taxonomy to uncover the “black box” approach that views all treatments as standard and interchangeable, understand the prevalence of therapeutic strategies, and study the effects of TOA and IOA in neurological conditions.

**References**

1. Rasova K, Feys P, Henze T, van Tongeren H, Cattaneo D, Jonsdottir J, Herbenova A. Emerging evidence-based physical rehabilitation for multiple sclerosis - towards an inventory of current content across Europe. Health Qual Life Outcomes. 2010 Jul 28;8:76. doi: 10.1186/1477-7525-8-76.

2. Huang H, Wolf SL, He J. Recent developments in biofeedback for neuromotor rehabilitation. J Neuroeng Rehabil. 2006 Jun 21;3:11.

3. Selzer M, Clarke S, Cohen L, Duncan P, Gage F . Textbook of Neural Repair and Rehabilitation: Volume 2, Medical Neurorehabilitation. Cambridge University Press, 2006.

4. Rasova K. Neurorehabilitation of People with Impaired Mobility Interventions and Assessment Tools. Third Medical Faculty, Charles University, Czech Republic. 2017

5. Hornby TG, Straube DS, Kinnaird CR, Holleran CL, Echauz AJ, Rodriguez KS, Wagner EJ, Narducci EA. Importance of specificity, amount, and intensity of locomotor training to improve ambulatory function in patients poststroke. Top Stroke Rehabil. 2011 Jul-Aug;18(4):293-307.

6. Pelosin E, Avanzino L, Barella R, Bet C, Magioncalda E, Trompetto C, Ruggeri P, Casaleggio M, Abbruzzese G. Treadmill training frequency influences walking improvement in subjects with Parkinson's disease: a randomized pilot study. Eur J Phys Rehabil Med. 2017 Apr;53(2):201-208.

7. Jonsdottir J, Cattaneo D, Recalcati M, Regola A, Rabuffetti M, Ferrarin M, Casiraghi A. Task-oriented biofeedback to improve gait in individuals with chronic stroke: motor learning approach. Neurorehabil Neural Repair. 2010 Jun;24(5):478-85.

8. Kjølhede T, Vissing K, Dalgas U. Multiple sclerosis and progressive resistance training: a systematic review. Mult Scler. 2012 Sep;18(9):1215-28.

9. Hart T, Tsaousides T, Zanca JM, Whyte J, Packel A, Ferraro M, Dijkers MP. Toward a theory-driven classification of rehabilitation treatments. Arch Phys Med Rehabil. 2014 Jan;95(1 Suppl):S33-44.e2.

## S40 How to build the therapeutic exercise starting from the analysis of the sign

### Laura Beccani, Giulia Borelli

#### AUSL Reggio Emilia, Santa Maria Nuova Hospital, Rehabilitation Unit for Serious Disabilities of Evolutive Age (UDGEE)

##### **Correspondence:** Laura Beccani (laurettha@hotmail.com)

The purpose of this report is to analyze the architecture of the function in the child with Cerebral Palsy (CP) through the analysis of the sign, to evaluate the nature of the defect and to design a suitable intervention for functional recovery and re-education.

It is important to understand how Cerebral Palsy is another pathway taken by the child in the construction of his adaptive functions, which we are still consider development. The different clinical forms of CP do not only represent a direct expression of the structural damage suffered by the CNS but constitute the recognizable manifestation of the pathway followed by the CNS to construct or "re" construct adaptive functions, despite the inevitable presence of the lesion.

The biological idea of paralysis as delay, slowing down, arrest, regression of development (semeiotics of the defects) must counteract the neuro-psycho-biological concept of the development of paralysis, as a new dynamic relationship that the individual trys “anyway” to build with the environment that surrounds him (semiotics of residual resources), to respond to the needs dictated by development, whose progression constitutes an unstoppable process (self-organization).

Knowing the pathology means recognizing the signs of paralysis as constraints imposed by the pathology but witnessing the logic followed by the SNC in constructing the performance and its adaptation to it. They reveal the margin of maneuver, or freedom of choice possessed by the SNC. This is the measure of the possible rehabilitation. It is therefore important to know how to identify the clinical sign and it is essential to know how to correctly interpret it as “defect” (consequence of a top down error or a bottom up alteration) or “compensation” (solution that the SNC puts in place to contain the consequences of an error that cannot be avoided or a defect that cannot be changed).

The function is an operational solution implemented by the child's SNC to satisfy a specific need that is biologically significant for him. The function is the final product of a process in which the organizational capabilities of the SNC interact, mutually influencing each other:

- the subject (top down components),
- the operative possibilities of his locomotor apparatus (bottom up components),
- the models offered by the community (imitation processes carried by mirror neurons),
- the physical characteristics of the environment (strategies for organizing the action, governed by canonical neurons)
- the clues contained in it (affordances).

The knowledge of the natural history of paralysis helps to outline the development strategy that will be followed by the child, the predictable path in the construction of the functions, towards which the therapy must be able to measure itself. If we understand the rules of self-reorganization process, by studying the behaviors of the past (natural history) and the present (functional diagnosis) we can reasonably predict future behavior (functional prognosis) and design therapeutic interventions.

The rehabilitative team tools available together or associated in the therapeutic intervention base on the identification and interpretation of the sign are:

1. Physiotherapy
2. Medications:
  Systemic
  District
  Focal
3. Orthoses and aids
4. Functional surgery
5. Adaptive modifications of the environment

The goal of functional rehabilitation is to realize the person with his / her differences and help them to become aware of their possibilities as well as their limitations.

Accepting the limit for the disabled child means renouncing an impossible future of normality in order to believe in a present of maximum autonomy, not only in motor terms but above all in the development of a personality and a proper thought (self-determination).

**References**

1. Bertozzi L, Montanari L, Mora I. Architettura delle funzioni: lo sviluppo neuromotorio del bambino fra normalità e patologia. Springer Science & Business Media, 2002.

2. Borelli G, Neviani R, Sghedoni A, Conti MR, Montanari L, Ovi A, Ferrari A. La fisioterapia nella paralisi cerebrale infantile: Principi ed esperienze-Postura seduta. Springer Science & Business Media, 2013.

3. Ferrari A, Cioni G. Le forme spastiche della paralisi cerebrale infantile. Guida all’esplorazione delle funzioni adattive. Ed. Sprinter, 2005.

## S41 How information from literature modify the practice of aquatic therapy: the multiple sclerosis

### Adriano Coladonato, Virginia Colibazzi, Fulvio Cavuoto, Marco Antonio Mangiarotti

#### A.N.I.K. (Associazione Nazionale IdroKinesiterapia)

##### **Correspondence:** Adriano Coladonato (adriano.coladonato@gmail.com)

**Background and Objective:** Multiple sclerosis (MS) is a chronic disease of the central nervous system, characterized by various inflammatory manifestations that lead to demyelination and subsequent axonal loss. Demyelination causes an alteration of the ionic mechanisms of conduction of the axonal membrane; this phenomenon, known as Uhthoff's phenomenon, explains the exacerbation of patients' symptoms in response to thermal stress induced by a passive exposure to heat, by exercise (which increases metabolism) or by both factors. The sensitization to heat is also worsened by a central alteration of the thermoregulatory mechanisms. [1] In the past, health professionals instructed patients with MS to minimize their exposure to high ambient temperatures, discouraging exercise or intense physical work. Today, numerous scientific evidence [2] indicates that exercise is recommended for people with MS in order to improve physical fitness, reduce fatigue and increase strength and safety while walking, but it should be performed avoiding excessive body heat and therefore establishment of the phenomenon of Uhthoff. This works aims to integrate the scientific evidence available to physical exercise in multiple sclerosis and to propose an updated practical work protocol.

**Materials and Methods:** Literature review.

**Results:** Studies demonstrate the correlation between aquatic exercise and neurotropic factors derived from the brain [3] as well as the induction of neurogenesis processes, neuroplasticity and the recovery of motor and cognitive functions [4]. Most of the intervention protocols proposed in the literature include the use of hydrobikes, aquatic treadmills or the re-adaptation of land-based exercises in water. The positive effects of exercise in aquatic settings include a better equilibrium condition linked to buoyancy (safe environment), better response to cardiovascular stress and lower metabolic expenditure, immediate cooling at the entrance to the water, the possibility of performing stretching and strenght exercises in better cardiovascular conditions also promoting patient autonomy.

**Conclusion:** To optimize the benefits deriving from the physical properties of water and to coherently associate them with the neuromotor principles of rehabilitation, it would be desirable to develop personalized therapeutic proposals to the patient's degree of disability through sequential exercises, progressive and functional to pre-established rehabilitative objectives.

Based on these premises, rehabilitative exercise in the aquatic context demonstrates both rational coherence with MS neurophysiology and clinical efficacy and should be proposed to patients within a shared multidisciplinary rehabilitation project.

**References**

1. Davis SL, Wilson TE, White AT, Frohman EM. Thermoregulation in multiple sclerosis. J Appl Physiol (1985). 2010 Nov;109(5):1531-7.

2. NICE. Multiple sclerosis in adults: management. Clinical guideline. Published: 8 October 2014, www.nice.org.uk/guidance/cg186.

3. Bansi J, Bloch W, Gamper U, Kesselring J. Training in MS: influence of two different endurance training protocols (aquatic versus overland) on cytokine and neurotrophin concentrations during three week randomized controlled trial. Multiple Sclerosis Journal. 2013;19(5):613-621.

4. Ellis T, Motl RW. Physical activity behavior change in persons with neurologic disorders: overview and examples from Parkinson disease and multiple sclerosis. J Neurol Phys Ther. 2013 Jun;37(2):85-90.

## S42 Translating motor control principles to practical applications in rehabilitation

### Mindy F. Levin (mindy.levin@mcgill.ca)

#### Professor, School of Physical and Occupational Therapy, 3654 Promenade Sir William Osler, Montreal, H3G 1Y5, Canada

The primary focus of neurological rehabilitation is the reacquisition of lost motor skills to improve independence in activities of daily living and quality of life. To achieve this, rehabilitation takes advantage of central nervous system neuroplasticity through motor learning mechanisms [1]. The purpose of this presentation is to describe how motor learning mechanisms can be addressed by creating enriched training environments using virtual reality (VR) based simulations. Motor control and motor learning principles related to the reacquisition of upper limb movement skills will be discussed in relation to how they can be exploited by VR training environments [2]. Virtual reality can address dynamical motor learning approaches that emphasize the dynamics of change in a movement sequence and its outcome over practice. This approach draws on the general idea of Bernstein (1967) that skill learning is reflected in redundant degrees of freedom. According to the dynamic approach, learning is a problem-solving system that uses available constraints and possibilities to discover solutions to a movement problem. In this scheme, acquiring coordination is not hampered by the many interacting variables (i.e., joint degrees of freedom), but simplified by them. This approach allows exploitation of the natural properties of the system. It is an emergent rather than reductive approach and gives rise to adaptability based on task demands and constraints [3]. Types of motor learning are reviewed and the advantages of using virtual reality to create enriched environments for task practice that incorporate different types and delivery schedules of feedback is discussed. Virtual reality environments for rehabilitation (‘virtual rehabilitation’) offer rich, controllable multi-modal stimulation, salient intrinsic (task-related) feedback that is programmable, the opportunity for learning by problem-solving that engages both motor and cognitive processes, motivation and arousal for the learner, and the opportunity to individualize activities and manipulate their level of difficulty. Different types of VR platforms include those that offer teacher-animation activities, problem-solving scenarios, game-like activity and those that have different levels of immersion. Key outcome measures are identified, and examples of how motor control and motor learning principles can be incorporated into different VR simulations (for improving upper limb motor function) are discussed. Finally, the limitations of current VR technologies with respect to their effectiveness are discussed, together with summaries of effectiveness evidence, client suitability for the use of different learning approaches, and transfer of learning to daily life tasks.

**References**

1. Nudo RJ. Adaptive plasticity in motor cortex: implications for rehabilitation after brain injury. J Rehabil Med. 2003 May;(41 Suppl):7-10.

2. Kleim JA, Jones TA. Principles of experience-dependent neural plasticity: implications for rehabilitation after brain damage. J Speech Lang Hear Res. 2008 Feb;51(1):S225-39.

3. Newell KM. Change in movement and skill: Learning, retention, and transfer. In: Latash ML, Turvey MT (Eds), Dexterity and its Development, Taylor and Francis: New York, 1996, pp. 393-430.

4. Bernstein NA. The Coordination and Regulation of Movements. Pergamon Press. 1967.

5. Levin MF, Weiss PL, Keshner EA. Emergence of virtual reality as a tool for upper limb rehabilitation: incorporation of motor control and motor learning principles. Phys Ther. 2015 Mar;95(3):415-25.

6. van Dijk L, van der Sluis C, Bongers RM. Reductive and Emergent Views on Motor Learning in Rehabilitation Practice. J Mot Behav. 2017 May-Jun;49(3):244-254.

7. Weiss PLT, Keshner EA, Levin MF. Virtual Reality for Physical and Motor Rehabilitation: New York. 2014.

## S43 Exergames as a strategy to maximize training in paediatric patients with respiratory diseases

### Beatrice Ferrari (beatrice.ferrari@meyer.it)

#### Rehabilitation Unit, Meyer Children Hospital, Florence, Italy

Exergames (exercise + gaming) are videogames that involve body movements and force reactions. They have been considered a potential tool to improve or maintain physical fitness also during rehabilitation of several conditions (e.g. Parkinson’s disease, balance impairement, multiple sclerosis, post stroke rehabilitation, acquired brain injury, cerebral palsy, low back pain).

Scientific literature about exergames is improving and new dedicated journals were founded (e.g. Games For Health Journal, JMIR Serious Games).

Research papers reported exergames as attractive and promising instruments for rehabilitation from childhood to elder population because they are low-cost, accessible and portable devices (console), and game tasks could be personalized in intensity and complexity.

Exergames could be useful also for pulmonary rehabilitation (PR). Amadeo et al. [5] compared cardiovascular and metabolic response during an exergame-based training session with those during an incremental field test (Modified Shuttle Walking Test) in Cystic Fibrosis children. They reported that an exergame-based session could be a moderate/high intensity activity and could easily reach the targeted heart rate throughout the entire workout.

Some papers showed a positive effect of exergames on adherence in healthy elderly and adults, but there is still a lack of data on long-term adherence of exergames especially in children. Nevertheless, as for every other treatment and prescription, exergames adherence is influenced by intrinsic motivation, impairment characteristic, setting properties, and last but not least operator communication and educational skills.

Training programs need to be personalized and optimized to the patient specific requirements, and also administered and supervised by specialized physiotherapists.

Exergames can be considered a real exercise with all its known benefits. We can get a workout from low intensity to high intensity depending on the type of game selected and the type of population.

Once you set on a single patient and perform a training, exergames may give an answer to the lack of human resources (specialized respiratory physiotherapists) or the difficult access of patients to PR programs.

Although further studies are required, we can assume that exergames are able to promote the maintenance of higher levels of physical activity in patients with chronic lung disease

Adherence to long-term training is a challenge that probably we will not solve with a console, but why do not try to propose something that is really effective, inexpensive and also fun?

**References**

1. Levac D, Espy D, Fox E, Pradhan S, Deutsch JE. "Kinect-ing" with clinicians: a knowledge translation resource to support decision making about video game use in rehabilitation. Phys Ther. 2015 Mar;95(3):426-40.

2. Knols RH, Vanderhenst T, Verra ML, de Bruin ED. Exergames for patients in acute care settings: systematic review of the reporting of methodological quality, fitt components, and program intervention details. Games for Health Journal. 2016;5(3):224-235.

3. Carbonera RP, Vendrusculo FM, Donadio MV. Physiological responses during exercise with video games in patients with cystic fibrosis: A systematic review. Respir Med. 2016 Oct;119:63-69.

4. Salonini E, Gambazza S, Meneghelli I, Tridello G, Sanguanini M, Cazzarolli C, Zanini A, Assael BM. Active Video Game Playing in Children and Adolescents With Cystic Fibrosis: Exercise or Just Fun? Respir Care. 2015 Aug;60(8):1172-9.

5. Amadeo B, Innocenti D, Gambazza S, Zuffo S. Effects of Microsoft X-Box Kinect™ on children with cystic fibrosis: exercise or just fun?. Physiotherapy. 2015;101:e70

6. Larsen LH, Schou L, Lund HH, Langberg H. The Physical Effect of Exergames in Healthy Elderly-A Systematic Review. Games Health J. 2013 Aug;2(4):205-12.

7. Bishay LC, Sawicki GS. Strategies to optimize treatment adherence in adolescent patients with cystic fibrosis. Adolesc Health Med Ther. 2016 Oct 21;7:117-124.

## S44 Rehabilitative ultrasound imaging (RUSI) in the physiotherapy practice

### Alessia Quercioli (querciolialessia@gmail.com)

#### Master Executive in Sport Physiotheraphy, University of Siena (on course)

**Background:** In the last thirty years the interest for the use of Ultrasound Imaging in Physiotherapy has developed. In 2006 has been coined the term “Rehabilitative Ultra-sound Imaging” (RUSI), that points out the use of Ultrasound Imaging as a tool for assessment and treatment in neuro-muscular dysfunctions.

**Objectives:** Evaluation of the use of Ultrasound Imaging through RUSI technique in Physiotherapy, for the assessment and treatment of abdominals, paraspinals and pelvic floor muscles dysfunction.

**Materials and Methods:** The research has been conducted on MEDLINE and PEDro between May and October 2017, inserting 14 laces of search, with date of publication 10 years; English language; human kind. The duplicated articles, those with no remarkable title or abstract, and those that didn’t respect the inclusion and exclusion criteria have been excluded. The level of evidence of the studies has been attributed with the Classification ICSI of 2006.

**Results:** The research has produced 7322 articles of which, applying the above-quoted parameters, 10 have been maintained: 4 Randomized Controlled Trials; 1 Case Reports and 5 Letterature Reviews.

**Conclusion:** RUSI is a safe and non-invasive method for the measurement of muscle architecture (thickness) of the above-quoted muscular districts. The studies show good results for RUSI assessment of muscle morphology and behaviour and for RUSI feedback in healthy subjects and in patients with Lumbo-Pelvic dysfunctions. Indeed, it allows the Physiotherapist to observe in real time the activation of deepest muscles, and to the Patient to understand the correct execution of the motor task.

**References**

1. Henry SM, Westervelt KC. The use of real-time ultrasound feedback in teaching abdominal hollowing exercises to healthy subjects. J Orthop Sports Phys Ther. 2005 Jun;35(6):338-45.

2. Henry SM, Teyhen DS. Ultrasound imaging as a feedback tool in the rehabilitation of trunk muscle dysfunction for people with low back pain. J Orthop Sports Phys Ther. 2007 Oct;37(10):627-34.

3. Callaghan MJ. A physiotherapy perspective of musculoskeletal imaging in sport. Br J Radiol. 2012 Aug;85(1016):1194-7.

4. Painter EE, Ogle MD, Teyhen DS. Lumbopelvic dysfunction and stress urinary incontinence: a case report applying rehabilitative ultrasound imaging. J Orthop Sports Phys Ther. 2007 Aug;37(8):499-504.

5. Teyhen DS, Miltenberger CE, Deiters HM, Del Toro YM, Pulliam JN, Childs JD, Boyles RE, Flynn TW. The use of ultrasound imaging of the abdominal drawing-in maneuver in subjects with low back pain. J Orthop Sports Phys Ther. 2005 Jun;35(6):346-55.

6. Teyhen D. Rehabilitative Ultrasound Imaging Symposium San Antonio, TX, May 8-10, 2006. J Orthop Sports Phys Ther. 2006 Aug;36(8):A1-3.

7. Teyhen DS, Gill NW, Whittaker JL, Henry SM, Hides JA, Hodges P. Rehabilitative ultrasound imaging of the abdominal muscles. J Orthop Sports Phys Ther. 2007 Aug;37(8):450-66.

8. Van K, Hides JA, Richardson CA. The use of real-time ultrasound imaging for biofeedback of lumbar multifidus muscle contraction in healthy subjects. J Orthop Sports Phys Ther. 2006 Dec;36(12):920-5.

9. Whittaker JL, Teyhen DS, Elliott JM, Cook K, Langevin HM, Dahl HH, Stokes M. Rehabilitative ultrasound imaging: understanding the technology and its applications. J Orthop Sports Phys Ther. 2007 Aug;37(8):434-49.

10. Worth SA, Henry SM, Bunn JY. Real-time ultrasound feedback and abdominal hollowing exercises for people with low back pain. New Zealand Journal of Physiotherapy. 2007;35(1):4.

## S45 Therapeutic exercise in respiratory failure critical ill patients and in intensive care unit: evidences and an Italian experience

### Marta Lazzeri, Chiara Lorenza Bioletto, Maria Elena Mazzanti, Carla Novo, Simona Pellegrina

#### Cardiothoracic and Vascular Department ASST Grande Ospedale Metropolitano Niguarda Milano

##### **Correspondence:** Marta Lazzeri (marta.lazzeri@ospedaleniguarda.it)

Medical and healthcare technological advances have improved Intensive Care Unit (ICU) survival, but a lot of patients, who survived the acute respiratory distress syndrome, developed functional limitations, muscle wasting, weakness and reduced quality of life.

This impairment may also persist at one year or more after discharge from the ICU [1]. ICU-acquired weakness (ICU-AW) is a frequent problem and its incidence is nearly 50% in patients with sepsis, multi-organ failure or protracted mechanical ventilation [2]. ICU-AW may be due to an axonal polyneuropathy or myopathy or a combination of both. Prolonged mechanical ventilation also results in diaphragm weakness or atrophy and this condition can make difficult to wean these patients.

The main risk factors for developing ICU-AW are prolonged immobilization, length of ICU stay, duration of mechanical ventilation, severity and duration of systemic inflammatory response, hyperglycemia, corticosteroid administration and neuromuscular-blocking agents [3].

Early progressive mobilization is defined as a wide range of activities, rolling from supine to side-lying, moving in bed, in-bed cycling, sitting on the edge-of-bed, transferring to/from a chair and walking, activities performed also with mechanically ventilated ICU patients.

Historically, patients in the ICU were considered “too sick” to tolerate a physical activities program, instead in the last ten years increasing scientific research showing that the early progressive mobilizations can be safe and feasible with patients in ICU.

The early mobilization programs have been shown to reduce the incidence of pulmonary complications and delirium, decrease the duration of mechanical ventilation, the length of ICU and hospital stay and improved functional outcomes [4,5].

We retrospectively analysed the outcome of 37 patients (28 male/9 females, age 61± 11 years ) consecutively admitted to cardiac surgery ICU, ASST Niguarda Hospital Milan, between 1 January and 30 June 2015 requiring prolonged mechanical ventilation, ICU stay for more than 7 days and showing severe and very severe impairments in functional autonomy in the immediate postoperative period. All patients are subjected to early mobilization and the ultimate goal of physical therapy was the patient’s return to his/her pre-morbid functional level.

Table 1 shows the characteristics of patients and postoperative care interventions.

Daily physical therapy was delivered everyday (7/7) to each patient as soon as their hemodynamic assessment allowed physical treatment, even while on mechanical ventilation.

We recorded the number of treatment sessions administered, the adverse events that occurred and the functional level at hospital discharge.

The mean ICU length of stay was 15 (±9) days and total days hospital stay was 35 (± 19) days. The 46% of patients were seated within the third postoperative day, of these 89 % still had infusion of vasoactive agents (amines and sodium nitroprusside) and all walk independently or with an aid, for example with a trolley, at hospital discharge.

Adverse events were infrequent, occurring in 4 of 975 (0.4%) treatment sessions performed (3 severe hypotension and 1 very severe bradycardia) and no patient was extubated or died during physical activity.

We conclude that early activity is feasible and safe in our clinical practice with patients undergone to cardiovascular surgery followed by prolonged recovery in ICU and results in progressive improvement of functional autonomy.

**References**

1. Herridge MS, Cheung AM, Tansey CM, Matte-Martyn A, Diaz-Granados N, Al-Saidi F, Cooper AB, Guest CB, Mazer CD, Mehta S, Stewart TE, Barr A, Cook D, Slutsky AS; Canadian Critical Care Trials Group. One-year outcomes in survivors of the acute respiratory distress syndrome. N Engl J Med. 2003 Feb 20;348(8):683-93.

2. Puthucheary ZA, Rawal J, McPhail M, Connolly B, Ratnayake G, Chan P, Hopkinson NS, Phadke R, Dew T, Sidhu PS, Velloso C, Seymour J, Agley CC, Selby A, Limb M, Edwards LM, Smith K, Rowlerson A, Rennie MJ, Moxham J, Harridge SD, Hart N, Montgomery HE. Acute skeletal muscle wasting in critical illness. JAMA. 2013 Oct 16;310(15):1591-600.

3. Stevens RD, Dowdy DW, Michaels RK, Mendez-Tellez PA, Pronovost PJ, Needham DM. Neuromuscular dysfunction acquired in critical illness: a systematic review. Intensive Care Med. 2007 Nov;33(11):1876-91.

4. Calvo-Ayala E, Khan BA, Farber MO, Ely EW, Boustani MA. Interventions to improve the physical function of ICU survivors: a systematic review. Chest. 2013 Nov;144(5):1469-1480.

5. Hodgson CL, Stiller K, Needham DM, Tipping CJ, Harrold M, Baldwin CE, Bradley S, Berney S, Caruana LR, Elliott D, Green M, Haines K, Higgins AM, Kaukonen KM, Leditschke IA, Nickels MR, Paratz J, Patman S, Skinner EH, Young PJ, Zanni JM, Denehy L, Webb SA. Expert consensus and recommendations on safety criteria for active mobilization of mechanically ventilated critically ill adults. Crit Care. 2014 Dec 4;18(6):658.

**Table 1 (abstract S45).** Characteristics of patients and postoperative care interventions

	Average ± SD	N° patients (%)
Surgical procedure		
Valve replacement		12 (33%)
Hearth transplant		5 (13,5%)
Ventricular assist device		5 (13,5%)
Coronary artery bypass graft (cabg)		5 (13,5%)
Thoracic aorta replacement		5 (13,5%)
Valve replacement +cabg		3 (8%)
Others		2 (5%)
Postoperative supports		
Mechanical ventilation (days)	10 ± 9 days	37(100%)
Non invasive mechanical ventilation	15 ± 12 days	32(87%)
Reintubation		14(37%)
Tracheostomy		14(37%)
Aortic Balloon Pump (IAPB)	6 ± 3 days	11(10%)
Veno-arterial extracorporeal membrane oxygenation (ECMO A-V)	5 days	1
Continuous veno-venous hemofiltration (CVVH)	12 ± 13 days	6 (16%)
Intravenous sedation	8 ± 4 days	29(78%)
Amines	10 ± 5 days	33(90%)
Sodium nitroprusside (SNP)	8 ± 4 days	29 (78%)

## P1 Phase IV of cardiac rehabilitation: a clinical vignette

### Andrea Aliberti (andrea.aliberti@virgilio.it)

#### Freelance physiotherapist

**Background and Objective:** Phase IV of Cardiac Rehabilitation (CR) aim to improve the quality of life and to reduce the long-term secondary risks. This is achieved through physical activity and improvement in patient's lifestyle. Clinical treatment and patient management should be guided by international guidelines and scientific literature, pertaining physiology of cardiovascular system, its interaction with other organs, and its response to physical activity. Aim of this paper was to summarises principles and indications to guide phase 4 of CR.

**Materials and Methods:** A clinical vignette was used to show how the principles should be applied in a clinical setting.

**Results:** A volitional workout test in a patient enrolled in phase IV of CR is reported in Figure 1, with a 3 years follow-up. First, an accurate medical history of the patient (health history and habits of life) should be performed, and the rehabilitation program should be drafted and shared with the multidisciplinary team of health personnel who has in charge the patient. The multidisciplinary team composition depends also to comorbidities that afflict the patient. Emphasis was placed on the fundamental problem for the success of phase 4: the patient's ability to change his lifestyle. The goal was to demonstrate that at this stage the patient's emotional involvement is strategic to achieve his treatment compliance over time. The importance of communicating their rehabilitation program well to patients was also emphasized. The choice of how to present the program to the patient must be well managed and modulated on variables such as age and sex of the patient, ethnicity, cultural level, systemic conditions of the patient (cardiopathies and possible comorbidities), habits of daily life, economic, psychological , social, or work conditions, desires and objectives, and the presence of a caregiver close to the patient.

**Conclusions:** The patient must feel at the center of the program: the more emotionally he is involved, the greater the effectiveness of the program in the course of his life.

**References**

1. Zipes DP, Peter L, Bonow RO, Braunwald E. Malattie del cuore di Braunwald. Elsevier srl. 2007.

2. BACPR. Standard and Core Components for Cardiovascular Disease Prevention and Rehabilitation 2017. https://www.bacpr.com/resources/BACPR_Standards_and_Core_Components_2017.pdf

**Fig. 1 (abstract P1).**
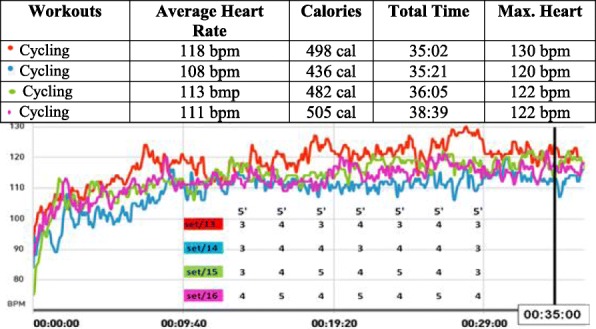
Feedback with heart rate data, time, degree of difficulty from the beginning (September 2013) to the third year (September 2016) of a patient enrolled in CR Phase IV. Legend: BPM, beat per minute

## P2 Physiotherapists’ perception of exercise-based applications for smartphones and tablets

### Maddalena Amadori (maddalena.fisio@ravenna33.it)

#### PgDip, Ravenna 33, Ravenna, Italy

**Background and Objective:** There is a growing interest in both healthcare and physiotherapy studies that investigated the use of applications (app) for mobile devices. To our knowledge there is a lack of study which investigated the physiotherapists’ perception of using exercise-based mobile applications in physiotherapy in the United Kingdom. Objectives of this study were to identify the knowledge that physiotherapists have about app, to explore the physiotherapists’ experience of using exercise-based app, to understand the use of exercise-based mobile app within physiotherapy setting, to explore the context in which the physiotherapists advise the patients on the app, and finally to explore limits and advantages of exercise-based mobile app from the physiotherapists’ perspective.

**Materials and Methods:** Six physiotherapists working in different areas in UK were recruited. The six physiotherapists were interviewed using semi-structured interviews. The transcripts were then analysed through a thematic-analysis.

**Results:** The main findings were presented as 5 themes which emerged from the interviews’ transcription: application’s design, economics, physiotherapist-patient interaction, patient-centered, physical activity. The 5 themes were discussed unpinning the codes from which the themes emerged.

**Conclusion:** The themes were strictly related, physiotherapists have knowledge of mobile app mainly within physical activity. Participants highlighted the need for exercise-based mobile app to be patient-centered and easily accessible, of low cost and with good design. Physiotherapist-patient interaction may be improved by the use of mobile app. From the interviews emerged how physiotherapists’ clinical expertise should be used in mobile app’s design and development. Further research should be conducted to confirm those findings using a triangulation approach, interviewing a larger and more heterogeneous group of physiotherapists. Cost was the major limit identified, while use of mobile app may be a time-saving for clinicians,and mobile app could be used to monitor patients’ symptoms and compliance with exercises.

**References**

1. Braun V, Clarke V. Using thematic analysis in psychology. Qual Res Psychol. 2006; 3(2):77-101

2. Bort-Roig J, Gilson ND, Puig-Ribera A, Contreras RS, Trost SG. Measuring and influencing physical activity with smartphone technology: a systematic review. Sports Med. 2014 May;44(5):671-86.

3. Dute DJ, Bemelmans WJ, Breda J. Using mobile Apps to promote a healthy lifestyle amongst adolescents and students: a review of the theoretical basis and lessons learned. JMIR Mhealth Uhealth. 2016;4(2):e39.

4. Gefen R, Dunsky A, Hutzler Y. Balance training using an iPhone application in people with familial dysautonomia: three case reports. Phys Ther. 2015 Mar;95(3):380-8.

5. Kim K, Pham D, Schwarzkopf R. Mobile Application Use in Monitoring Patient Adherence to Perioperative Total Knee Arthroplasty Protocols. Surgical Technology International. 2019;4 (XXVIII):1-8

## P3 Cochrane Rehabilitation Field: evidence to rehabilitation and rehabilitation expertise to Cochrane

### Chiara Arienti^1^, Francesca Gimigliano^2^, Joel Pollet^3^, Carlotte Kiekens^4^, Stefano Negrini^3^

#### ^1^IRCCS Don Carlo Gnocchi Foundation, Milan, Italy; ^2^Department of Mental and Physical Health and Preventive Medicine, University of Campania “Luigi Vanvitelli”, Napoli, Italy; ^3^Clinical and Experimental Sciences Department, University of Brescia, Italy; ^4^Physical & Rehabilitation Medicine, University Hospitals Leuven, Belgium

##### **Correspondence:** Joel Pollet (joel.pollet.ft@gmail.com)

**Background and Objective:** Based on an initiative of the European Society of Physical and Rehabilitation Medicine (ESPRM), the idea of a Cochrane Rehabilitation Field was supported by a number of organisations, including the International Society of Physical and Rehabilitation Medicine (ISPRM). After approval by Cochrane Steering Group, Cochrane Rehabilitation has been launched on December 2016. The aim of Cochrane Rehabilitation is to bridge between Cochrane and Rehabilitation stakeholders, systematically identifying and spreading evidence, but also improving its quality and quantity production per clinical needs.

**Material and Methods:** Cochrane Rehabilitation is a network of individuals, coming from all continents. Therefore, a clear and well-structured organisation is required to make Cochrane Rehabilitation function effectively.

**Results:** Up to now 230 people from 49 countries expressed their willingness to collaborate. The Field Director will be directly responsible for the Knowledge Translation strategy and will be assisted by the Executive Committee. The Field Coordinator will ensure the implementation of a networking strategy, daily planning, organisation and coordination of activities between the Committees (Communication, Education, Methodology, Publication and Rehabilitation Reviews), Units and individual members. The Advisory Board will include key persons from different international stakeholders as well as recognised opinion leaders in rehabilitation.

**Conclusion:** Cochrane Rehabilitation will drive, on one side, evidence and methods developed by Cochrane to the world of Rehabilitation and, on the other, convey priorities, needs and specificities of Rehabilitation to Cochrane.

## P4 Cochrane and World Health Organization “Rehabilitation 2030: a call for action”

### Chiara Arienti^1^, Francesca Gimigliano^2^, Joel Pollet^3^, Carlotte Kiekens^4^, Stefano Negrini^1,3^

#### ^1^IRCCS Don Carlo Gnocchi Foundation, Milan, Italy; ^2^Department of Mental and Physical Health and Preventive Medicine, University of Campania “Luigi Vanvitelli”, Napoli, Italy; ^3^Clinical and Experimental Sciences Department, University of Brescia, Italy; ^4^Physical & Rehabilitation Medicine, University Hospitals Leuven, Belgium

##### **Correspondence:** Joel Pollet (joel.pollet.ft@gmail.com)

**Background and Objective:** The World Health Organization (WHO) has launched in February 2017 “Rehabilitation 2030 - a call for action”. This is likely to have a deep impact in the Health Systems in the next few years. Cochrane has approved the new Rehabilitation Field, that has been invited by WHO as a relevant stakeholder in this effort. WHO recognises the dramatic changes in health and demographic profiles of populations that are characterizing the 21^st^ century. People are living longer, with disabling chronic conditions and disabilities that impact their functioning and well-being. Main goals of WHO are to ensure healthy lives and promote well-being for all at all ages, and to articulate the importance of promoting healthy life expectancy. Health systems are confronted with these emerging challenges; hence, health policies are placing increased emphasis on services targeted at improving functioning, and not only at decreasing morbidity and mortality.

**Material and Methods:** According to WHO, rehabilitation could be an answer to this need. Cochrane’s strategy becomes significant in this context, as it is based on the production of high-quality evidence through systematic reviews to inform health decision making.

**Results:** Cochrane Rehabilitation is the appropriate instrument in this endeavour: its main goal is to convey to all rehabilitation professionals the best available evidence as gathered by high quality Cochrane systematic reviews, but also to improve the Cochrane methods for evidence synthesis. This will help rehabilitation professionals to make decisions according to the best and most appropriate evidence.

**Conclusion:** An important challenge of Cochrane Rehabilitation in the next future is to respond to the WHO “Rehabilitation 2030” call for action.

## P5 Comparison of physiotherapy and mindfulness in Patients with Parkinson’s Disease

### Roberta Balestriero^1^, Manuela Maieron^1^, Simona Schiavoni^1^, Vincenzo Patruno^1^, Davide Anchisi^2^, Fabio Forniz^3^

#### ^1^Istituto di Medicina Fisica e Riabilitazione ASUIUD (UD) Italy, Istituto di Medicina Fisica e Riabilitazione SOC Pneumologia Riabilitativa ASUIUD (UD) Italy; ^2^Università di Udine Facoltà di Medicina e Chirurgia (UD); ^3^Studente Corso di Laurea Fisioterapia (UD) Italy

##### **Correspondence:** Manuela Maieron (manuela.maieron@asuiud.sanita.fvg.it)

**Background and Objective:** Aim of this study was to clarify if a mindfulness plus rehabilitation program has better clinical and functional outcomes than rehabilitation program alone in patients with Parkinson disease (PD) .

**Materials and Methods:** Twenty-one patients with PD (HY ≤2, MMSE ≥ 24, 18 men, mean age 66yrs) were randomized in 3 different groups: group P (7 patients: only Physiotherapy), group PM (9 patients: physiotherapy plus 8-weeks of mindfulness), and group M (5 patients: only 8 -weeks of mindfulness). All patients underwent to UPDRS III, TUG, 10MWT, ABC, PDQ-39, NRS at pre and post treatment. In all patients we evaluated sympathovagal balance by spectral analysis of Heart Rate Variability (HF/LF) at pre and post treatment. T-test and Bayesan interference test were applyed in the within and between group statistical analisys.

**Results:** Significant within-group results were found in groups P (UPDRS III p= 0.033; TUG p=0.037; NRS p=0.042), PM (UPDRS III p=0.024; TUG p=0.025; 10MWT p=0.027; NRS p=0.047), and M (ABC p=0.010). Between-groups analysis showed better results in groups P and PM compared to group (NRS score). However, group M showed better results in ABC than groups P and PM. No statistical difference was found in sympathovagal balance (HF/LF p>0.5).

**Conclusions:** The present data supported a key-role of physioterapy program and suggested a supportive role of mindfulness when associate to a rehabilitation program in the management of patient with PD. A clear effect of rehabilitation program or mindfulness on sympathovagal balance was not supported. The main limitation of this study was a small sample size.

**References**

1. Pickut B, Vanneste S, Hirsch MA, Van Hecke W, Kerckhofs E, Mariën P, Parizel PM, Crosiers D, Cras P. Mindfulness Training among Individuals with Parkinson's Disease: Neurobehavioral Effects. Parkinsons Dis. 2015;2015:816404.

2. Krygier JR, Heathers JA, Shahrestani S, Abbott M, Gross JJ, Kemp AH. Mindfulness meditation, well-being, and heart rate variability: a preliminary investigation into the impact of intensive Vipassana meditation. Int J Psychophysiol. 2013 Sep;89(3):305-13.

## P6 Comparison of mechanical vibration and manual therapy for the treatment of cervical pain associated with postural dysfunction

### Giovanni Barassi^1^, Tim Ainslei^2^, Rosa Grazia Bellomo^3^, Giuseppe Giannuzzo^4^, Claudia Barbato^4^, Ilaria Pecoraro^4^, Raoul Saggini^5^

#### ^1^Coordinator of Degree Course in Physiotherapy. "Gabriele d'Annunzio" University – Chieti-Pescara, Italy; ^2^Senior Lecturer Degree Course in Physiotherapy. Faculty of Health and Life Sciences, Department of Sport and Health Sciences. Oxford Brookes University, United Kingdom; ^3^Associate Professor in Physical and Rehabilitation Medicine. "Gabriele d'Annunzio" University – Chieti-Pescara, Italy; ^4^Lecturer and Clinical Educator.Degree Course in Physiotherapy. "Gabriele d'Annunzio" University – Chieti-Pescara, Italy; ^5^Full Professor in Physical and Rehabilitation Medicine. President of Degree Course in Physiotherapy."Gabriele d'Annunzio" University – Chieti-Pescara, Italy

##### **Correspondence:** Giovanni Barassi (dottgiovannibarassi@gmail.com)

**Background and Objectives:** Neck pain (NP) is a common symptom in the general population. It is estimated that up to 67% of adults will experience neck pain at some stage in their lives. There is some evidence that Mulligan Mobilizations (MM) may improve joint function and mobility, and that Focal Vibration Sound System (FVSS) therapy may contribute to improve muscular parameters like elasticity, tone, and stiffness. The aim of this study was to evaluate and compare the effectiveness of MM and FVSS as independent approaches for postural NP.

**Materials and Methods:** A total of 30 participants were recruited after a clinical diagnosis of NP. Suitable patients were randomly allocated to two groups. Patients in the group A performed 6 sessions of Mulligan’s SNAGs intervention while patients in the group B performed 6 sessions of FVSS. Both treatments were combined with a home-based exercise program. Outcome measures were: Neck Disability Index, VAS, pressure algometer, neck’s range of motion via the Kinovea® software, muscular parameters via the Myoton, and posture by using the RAROG software.

**Results:** Excellent results were obtained in both groups for range of neck flexion and rotation. There were significant reductions in pain scores for both groups. For the Myoton parameters significant improvements were observed in both A (elasticity) and B group (elasticity and stiffness). Postural changes of head position and shoulders - both in frontal and side plane - were detected in B group.

**Conclusion:** Both interventions were found to be effective in approaching postural NP by improving range of motion and muscular elasticity. A greater effectiveness regarding muscle stiffness and postural correction was obtained with FVSS. Further studies with a control group are recommended, and an investigation on the application of MM and FVSS as a combined treatment compared to single application is suggested.

**References**

1. Brink Y, Louw QA. A systematic review of the relationship between sitting and upper quadrant musculoskeletal pain in children and adolescents. Man Ther. 2013 Aug;18(4):281-8

2. Mulligan BR. Manual Therapy: “Nags” “Snags” “MWMs”. 2004. Orthopaedic Physical Therapy and Rehabilitation

3. Pietrangelo T, Mancinelli R, Toniolo L, Cancellara L, Paoli A, Puglielli C, Iodice P, Doria C, Bosco G, D'Amelio L, di Tano G, Fulle S, Saggini R, Fanò G, Reggiani C. Effects of local vibrations on skeletal muscle trophism in elderly people: mechanical, cellular, and molecular events. Int J Mol Med. 2009 Oct;24(4):503-12.

4. Silva AG, Punt TD, Sharples P, Vilas-Boas JP, Johnson MI. Head posture and neck pain of chronic nontraumatic origin: a comparison between patients and pain-free persons. Arch Phys Med Rehabil. 2009 Apr;90(4):669-74.

## P7 Tinnitus and somatic dysfunction: the role of neuromuscular manual therapy

### Giovanni Barassi^1^, Rosa Grazia Bellomo^2^, Giuseppe Irace^3^, Ilaria Pecoraro^3^, Federico Pavone^4^, Raoul Saggini^5^

#### ^1^Coordinator of Degree Course in Physiotherapy. “Gabriele d'Annunzio” University – Chieti-Pescara, Italy; ^2^Associate Professor in Physical and Rehabilitation Medicine. “Gabriele d’Annunzio” University – Chieti-Pescara, Italy; ^3^Lecturer and Clinical Educator.Degree Course in Physiotherapy. “Gabriele d’Annunzio” University – Chieti-Pescar, Italy; ^4^Degree Course in Physiotherapy. “Gabriele d’Annunzio” University – Chieti-Pescara, Italy; ^5^Full Professor in Physical and Rehabilitation Medicine. President of Degree Course in Physiotherapy. “Gabriele d’Annunzio” University – Chieti-Pescara, Italy

##### **Correspondence:** Giovanni Barassi (dottgiovannibarassi@gmail.com)

**Background and Objectives:** Tinnitus, or the perception of a ghost sound not perceptible by other people, is a growing problem in the general population. It can be defined as objective, that is a sound generated by the body that is perceived by the auger, and subjective, caused by an aberrant electric activity generated in the auditory centers that emulates the activity evoked by the sounds. Recent studies have shed light on the role that somatic-sensory informations from the periphery has in the genesis of such aberrant activity of auditory centers. Physiotherapy, therefore, through neuromuscular manual therapy can play a central role in tinnitus management. In this study, we aimed to evaluate the effectiveness of neuromuscular manual therapy as a tinnitus management tool, comparing its effects with the classic cognitive dysfunction approach.

**Materials and methods:** An experimental group A (n=10) and a control group B (n=15) were subjected to two different therapies: group A received 8 seizures of neuromuscular therapy, group B was subjected to standardized TRT cognitive-behavioral therapy. THI questionnaire was used as outcome measure. Group A was also measured by 3 numerical scales (1-100) on some aspects of tinnitus.

**Results:** After treatment, both groups showed significant improvements compared to baseline. The average change on the THI total score was of 7 points in group A (p=0.0088) and of 31.7 points in group B (p=0.000721). Numerical scales scores in group A were all significantly improved: mean volume of tinnitus (mean change 16 points, p=0.00557), time percentage with tinnitus (mean change 24.5 points, p=0.0124), and time percentage with negative feelings and emotions (mean change 13.5 points, p=0.030516).

**Conclusions:** These results showed that neuromuscular manual therapy can play a significant role in the management of subjective tinnitus. Hence, further studies are expected, with the extension of the sample size and combining neuromuscular manual therapy with standard cognitive therapy.

**References**

1. Hoffman HJ; Reed GW. Epidemiology of tinnitus. Tinnitus: Theory and management. 2004. 16-41.

2. Shore S; Zhou J, Koehler S. Neural mechanisms underlying somatic tinnitus. Prog Brain Res. 2007;166:107-23.

3. McPartland JM, Simons DG. Myofascial trigger points: translating molecular theory into manual therapy. J Man Manip Ther. 2006;14(4):232-9.

4. Levine RA, Nam EC, Oron Y, Melcher JR. Evidence for a tinnitus subgroup responsive to somatosensory based treatment modalities. Prog Brain Res. 2007;166:195-207.

## P8 A home-based exercise program can improve ankle range of motion in patients with venous ulcer

### Rosa Grazia Bellomo^1^, Antonio Antico^2^, Lorella Capriotti^2^, Antonella Di Iuilio^3^, Giovanni Barassi^4^, Piera Attilia Di Felice^3^, Loris Prosperi^3^, Raoul Saggini^5^

#### ^1^Associate Professor in Physical and Rehabilitation Medicine. “Gabriele d’Annunzio” University – Chieti-Pescar, Italy; ^2^Vascular Surgery Division. S Spirito Hospital –Pescara, Italy; ^3^Lecturer and Clinical Educator.Degree Course in Physiotherapy. “Gabriele d’Annunzio” University – Chieti-Pescara, Italy; ^4^Coordinator of Degree Course in Physiotherapy. “Gabriele d’Annunzio” University – Chieti-Pescara, Italy; ^5^Full Professor in Physical and Rehabilitation Medicine. President of Degree Course in Physiotherapy. “Gabriele d’Annunzio” University – Chieti-Pescara, Italy

##### **Correspondence:** Giovanni Barassi (dottgiovannibarassi@gmail.com)

**Background and Objectives:** Leg ulcerations are a common problem, with an estimated prevalence of 1% to 2% in the adult population and they are primarily treated in outpatient settings. Moreover, decreased ankle mobility is associated with delayed healing of venous ulcers. In this study we highlighted the relationships between the range of ankle motion (ROAM) for adults with venous leg ulcers. Indeed, the aim of this study was to assess the efficacy of 8-week home-based exercise program in increasing ROAM. The effect of exercise will also be considered in relation to the healing rates for adults experiencing venous leg ulceration.

**Materials and Methods:** The study comprised 35 patients with long-standing venous ulcers. Participants were encouraged to undertake a home-based daily ankle exercise program in an 8-week single-arm pilot study. Patients were excluded if they had secondary pathologies such as pyoderma gangrenous, rheumatoid arthritis, uncontrolled diabetes mellitus, squamous cell carcinoma, suspected wound infection, osteomyelitis, lymphedema or vasculitis. The ROAM was assessed at baseline and early after treatment. The ROAM was assessed by goniometry in the supine, non weight-bearing position. Venous disease was classified according to the CEAP classification (International Consensus Committee reporting standards on venous disease). The exercise consisted in 10x3 sets 3 times per day everyday of: plantar flexion of the ankle, seated heel-rises (both legs) and standing heel-rises (both legs).

**Results:** After 8 weeks of treatment significant improvements were observed in ROAM (p=0.02), without any delayed healing of venous ulcers.

**Conclusion:** These results showed that a simple, home-based exercise program may contribute to improve ROAM and may help to promote the healing of venous ulcers. Good patients adherence to the program indicated also its feasibility. A larger randomized controlled study is needed to show whether there is a positive effect on ulcer healing.

**References**

1. Davies JA, Bull RH, Farrelly IJ, Wakelin MJ. A home-based exercise programme improves ankle range of motion in long-term venous ulcer patients. Phlebology. 2007;22(2):86-9.

2. Yim E, Richmond NA, Baquerizo K, Van Driessche F, Slade HB, Pieper B, Kirsner RS. The effect of ankle range of motion on venous ulcer healing rates. Wound Repair Regen. 2014 Jul-Aug;22(4):492-6.

3. O'Brien J, Finlayson K, Kerr G, Edwards H. Evaluating the effectiveness of a self-management exercise intervention on wound healing, functional ability and health-related quality of life outcomes in adults with venous leg ulcers: a randomised controlled trial. Int Wound J. 2017 Feb;14(1):130-137.

## P9 Transcranial electrical stimulation and cortical plasticity: the role in short term memory and rehabilitation

### Rosa Grazia Bellomo^1^, Giovanni Barassi^2^, Giusebbe Giannuzzo^3^, Piera Attilia Di Felice^3^, Loris Prosperi^3^, Antonella Di Iulio^3^, Raoul Saggini^4^

#### ^1^Associate Professor in Physical and Rehabilitation Medicine. “Gabriele d’Annunzio” University – Chieti-Pescara, Italy; ^2^Coordinator of Degree Course in Physiotherapy. “Gabriele d’Annunzio” University – Chieti-Pescara, Italy; ^3^Lecturer and Clinical Educator.Degree Course in Physiotherapy. “Gabriele d’Annunzio” University – Chieti-Pescara, Italy; ^4^Full Professor in Physical and Rehabilitation Medicine. President of Degree Course in Physiotherapy. “Gabriele d’Annunzio” University – Chieti-Pescara, Italy

##### **Correspondence:** Giovanni Barassi (dottgiovannibarassi@gmail.com)

**Background and Objectives:** DC transcranial stimulation (tDCS) is a neuro-modulation technique that allows to stimulate different cerebral parts without significant side effects. It consists of a weak electrical current which is applied to the scalp via a pair of protected electrodes. There is therefore an excitatory anode and an inhibitory cathode whose electrical activities are determined by a modification of the neuronal membrane potential. In rehabilitation the first electrical currents designed to stimulate the brain were introduced around 1870. To date, although its mechanisms are still not fully understood, tDCS represents a potentially useful tool for rehabilitation. The present study aimed at examining the relationship between tDCS stimulation and working memory.

**Materials and Methods:** In this randomized, single-blinded trial, 40 healthy subjects were included and divided into 2 equitable groups: an experimental group (group A) that received tDCS, and a control group (group B) subjected to placebo stimulation. Subjects have never experienced tDCS before. During the experiment, specific tests for working memory were performed, namely N-Back tests in two levels of difficulty. The sessions were in total 3 over a week on alternate days. In the various sessions, the performed tests were replicated by patients without stimulation, 40 minutes after the first stimulation, in order to evaluate the maintenance of cognitive performance following treatment (both real and placebo).

**Results:** The results showed that tDCS has long-term effects. In particular, the performance improvements of group A were significantly more stable and linear than those of the control group.These improvements can be considered as a treatment effect plus a training effect in the required tasks.

**Conclusions:** tDCS is a developing technique in reahabilitation, and further studies on its therapeutic effects are required. Despite this, our findings highlight how tDCS may introduce a new and potentially effective treatment in a variety of fields, from the neurological to the motor one. More detailed and structured studies on the potential and the specific applications of this innovative therapeutic approach are needed.

**References**

1. Rossini PM, Burke D, Chen R, Cohen LG, Daskalakis Z, Di Iorio R, Di Lazzaro V, Ferreri F, Fitzgerald PB, George MS, Hallett M, Lefaucheur JP, Langguth B, Matsumoto H, Miniussi C, Nitsche MA, Pascual-Leone A, Paulus W, Rossi S, Rothwell JC, Siebner HR, Ugawa Y, Walsh V, Ziemann U. Non-invasive electrical and magnetic stimulation of the brain, spinal cord, roots and peripheral nerves: Basic principles and procedures for routine clinical and research application. An updated report from an I.F.C.N. Committee. Clin Neurophysiol. 2015 Jun;126(6):1071-107.

2. Ardolino G, Bossi B, Barbieri S, Priori A. Non-synaptic mechanisms underlie the after-effects of cathodal transcutaneous direct current stimulation of the human brain. J Physiol. 2005 Oct 15;568(Pt 2):653-63.

3. Hoy KE, Emonson MR, Arnold SL, Thomson RH, Daskalakis ZJ, Fitzgerald PB. Testing the limits: Investigating the effect of tDCS dose on working memory enhancement in healthy controls. Neuropsychologia. 2013 Aug;51(9):1777-84.

4. Saggini R, Barassi G, Carmignano SM, Ancona E, Di Felice P, Giannuzzo G, Banchetti A, Bellomo RG. Bilateral Transcranial Direct-current Stimulation (tDCS) of Dorsolateral Prefrontal Cortex during Specific Working Memory Tasks. Int J Phys Med Rehabil. 2016;4:364.

## P10 Clinical pattern and psychosocial domains like risk factors of persistent pregnancy-related pelvic girdle pain (PPGP): a review

### Elisa Burani^1^, Daniele Ceron^2^, Gloria Giglioni^3^

#### ^1^University of Rome, Rome, Italy; ^2^ University of Rome and Padova, Padova, Italy. ^3^ University of Rome, Rome, Italy

##### **Correspondence:** Elisa Burani (elisaburani64@gmail.com)

**Background and Objective:** About 20% of pregnant women develop Pregnancy-related Pelvic Girdle Pain (PPGP). Among them, 7-10% presents self-limiting symptoms lasting 2 to 11 years after the birth, and leading to severe disability. Recently, the risk factors which contributes to the outbreak and to the maintenance of this process were investigated. A critical analisys of literature was used to identify psychosocial risk factors in women at greater risk of developing persistent PPGP, and to guide clinicians toearly identify this subgroup of patients.

**Materials and Methods:** Electronic search was carried out using 3 different databases: Pedro, Medline and Cochrane Library. The limit year for research publication was 2000, and the search was limited to English language articles. Eligibility criteria have been set for the selection of the articles. Population: women with diagnosis of PPGP or PGP+LBP, with no age limit, assessed through self-report questionnaires and/or clinical examination. Studies focused on women with traumatic, gynaecological or urological PGP, pregnancy-related LBP as well as those which take in consideration only biological factors, were excluded. Outcome assessment: to study the correlation with PPGP, patients have been followed in an observational-longitudinal prospective way, collecting outcome measurements in two or more follow-up; outcomes are clinical (through physical examination) and psychosocial (through questionnaires). Study selection has been made after examination of title, abstract and full text, discarding duplicate and not relevant articles.

**Results:** Fourteen articles were included in the review (12 prospective cohort studies, 1 prospective questionnaire, 1 cross-sectional study), whose results were qualitatively analyzed.

**Conclusions:** Intensity of pain, high number of positive tests and emotional distress are predictive factors of persistent PPGP. There are weak and contradictory evidences of relation between persistent PPGP and sick leave, sleep quality/quantity, catastrophising, working conditions; kineshiophobia, body perception, self-efficacy, improvement expectations, relathionship satisfaction and fear avoidance believe researches are lacking. Future studies should analyze in a systematic way pregnancy-related clincal patterns and psychosocial risk factors, related to after-birth persistent PGP and they should elaborate effective management strategies to be used with risk group during pregnancy.

**References**

1. Robinson HS, Veierød MB, Mengshoel AM, Vøllestad NK. Pelvic girdle pain--associations between risk factors in early pregnancy and disability or pain intensity in late pregnancy: a prospective cohort study. BMC Musculoskelet Disord. 2010 May 13;11:91.

2. Bakker EC, van Nimwegen-Matzinger CW, Ekkel-van der Voorden W, Nijkamp MD, Völlink T. Psychological determinants of pregnancy-related lumbopelvic pain: a prospective cohort study. Acta Obstet Gynecol Scand. 2013 Jul;92(7):797-803.

3. Elden H, Gutke A, Kjellby-Wendt G, Fagevik-Olsen M, Ostgaard HC. Predictors and consequences of long-term pregnancy-related pelvic girdle pain: a longitudinal follow-up study. BMC Musculoskelet Disord. 2016 Jul 12;17:276.

4. Bjelland EK, Stuge B, Engdahl B, Eberhard-Gran M. The effect of emotional distress on persistent pelvic girdle pain after delivery: a longitudinal population study. BJOG. 2013 Jan;120(1):32-40.

5. Bergström C, Persson M, Mogren I. Pregnancy-related low back pain and pelvic girdle pain approximately 14 months after pregnancy - pain status, self-rated health and family situation. BMC Pregnancy Childbirth. 2014 Jan 25;14:48.

## P11 Effects of high-load strength training after total knee arthroplasty: an observational study

### Simone Carantoni^1^, Simone Marivo^2^, Leonardo Piano^3^, Valentina Morra^3^, Simone Barbero^4^, Francesco Sartorio^5^, Stefano Vercelli^5^

#### ^1^Physiotherapy student, University of Insubria, Varese (VA), Italy; ^2^DOC Service SRL, Novara, Italy; ^3^Rehabilitation and Functional Recovery, Casa di Cura La Residenza, Rodello (CN), Italy; ^4^Physiotherapy student, University of Eastern Piedmont, Fossano (CN), Italy; ^5^Laboratory of Ergonomics and Musculoskeletal Disorders Assessment, Division of Physical Medicine and Rehabilitation, Istituti Clinici Scientifici Maugeri SpA-SB, IRCCS, Veruno (NO), Italy

##### **Correspondence:** Simone Carantoni (simocara94@gmail.com)

**Background and Objectives:** Quadriceps weakness is associated to poor functional outcomes after total knee arthroplasty (TKA) [1]. There is a lack of established standards for prescribing strength training early after TKA, but some studies [1-3] suggested that high intensity strength training may improve patient function without compromising safety. Aim of this study was to assess the effects of an early, progressive, high-intensity strength training in subjects with TKA.

**Material and Methods:** This is a controlled observational study. All subjects with primary unilateral TKA hospitalized in two rehabilitation institutes between July 2016 and July 2017 were considered eligible. Patients who fulfilled the inclusion/exclusion criteria were enrolled, and the estimated sample size was 18 subjects per group. In the experimental group patients received conventional therapy aimed to improve strength, ROM, gait, and function, with two exercises (leg extension and squat) performed three times/week with a progressive (8-15 RM) high-intensity (HI), according to the American College of Sports Medicine recommendations [4]. A control group of subjects hospitalized between June 2015 and June 2016 received the same treatment, but exercises were performed at lower intensities (LI). Outcome measures were quadriceps strength (1RM on leg extension; number of squat repetitions, Nsqrep) and function (10mwt; WOMAC score) [Figure 1]. Subjects were evaluated at baseline and discharge with an intention-to-treat analysis. Independent t-test and paired t-test were used respectively for between-groups and within-groups comparisons. Level of significance was set at 5%.

**Results:** Seventy-eight subjects were included to HI (n=36) or LI (n=42) group. No significant differences were observed at baseline between groups (p<0.001). Subject of both groups improved in all outcomes at discharge (all p<0.05). The between-groups analysis [Table 1] showed higher strength levels (1RM, p<0,05; Nsqrep, p<0.01) in HI group, but no differences were observed for 10mwt (p>0.05) and WOMAC (p>0.05).

**Conclusion:** Both the HI and LI interventions were effective in improving strength and function after TKA in the acute setting. The HI intervention was safe (no adverse events were reported) and showed higher amount of strength gain than LI. Follow-up are needed to explore functional performance in the long term.

**References**

1. Bade MJ, Stevens-Lapsley JE. Early high-intensity rehabilitation following total knee arthroplasty improves outcomes. J Orthop Sports Phys Ther. 2011;41:932-41

2. Husby VS, Helgerud J, Bjorgen S, Husby OS, Benum P, Hoff J. Early maximal strength training is an efficient treatment for patients operated with total hip arthroplasty. Arch Phys Med Rehabil. 2009;90:1658-67.

3. Jakobsen TL, Husted H, Kehlet H, Bandholm T. Progressive strength training (10RM) commenced imemdiately after fast-track total knee arthroplasty: is it feasible? Disabil Rehabil 2012;34:1034-40.

4. American College of Sports Medicine. American College of Sports Medicine position stand. Progression models in resistance training for healthy adults. Med Sci Sports Exerc. 2009;41(3):687-708.

**Table 1 (abstract P11).** Between-groups comparison of changes for all outcomes measures. Data are expressed as mean ± standard deviation

Outcome Measures	Mean changes pre-post treatment	
	High Intensity	Low Intensity
1 RM (kg)	7.06 ± 4.67	4.27 ± 3.77
Squat^†^ (N Rep.)	18.54 ± 18.13	7.92 ± 8.83
10mwt (sec.)	-3.70 ± 16.24	-1.80 ± 13.44
WOMAC pain	-4.68 ± 2.89	-4.32 ± 3.47
WOMAC function	-12.45 ± 6.64	-12.86 ± 7.00

Legend: WOMAC, Western Ontario and McMaster Universities Osteoarthritis Index; *, Statistically significant difference; ^†^, Squat analysis was performed on 24 subjects of HI, and 26 subjects of LI groups

**Fig. 1 (abstract P11).**
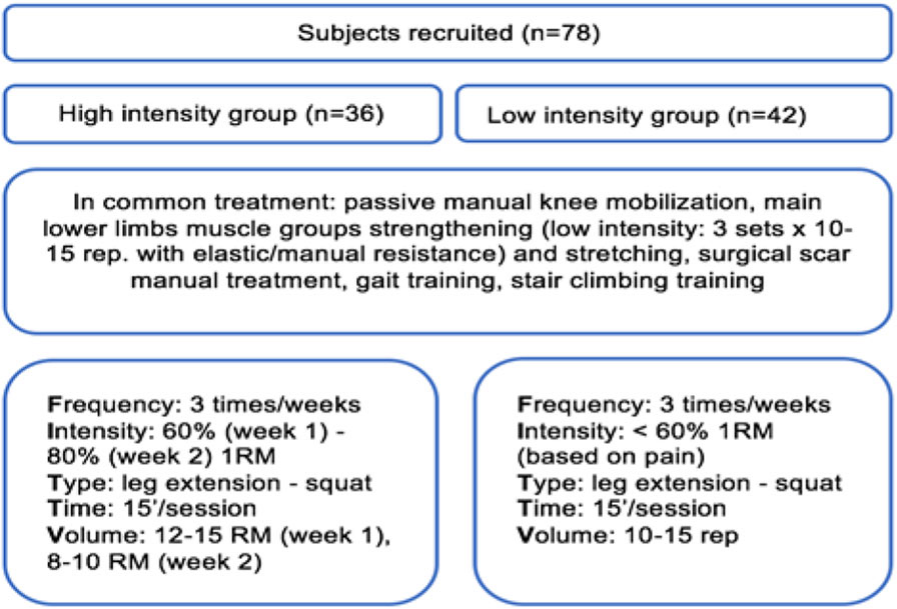
Treatment regimens. Both group underwent the same conventional rehabilitation program except for the intensity of strength training parameters regarding leg extension and squat exercises

## P12 Longitudinal prevention study of low back pain in sport children: a preliminary case-control study

### Valentina Cattaruzza, Francesca Policastro, Roberto Marcovich, Elisa Priano, Manuela Deodato

#### University of Trieste, Trieste, Italy

##### **Correspondence:** Valentina Cattaruzza (vale.cattaruzza@hotmail.it)

**Background and Objective:** Epidemiologic evidences about Low Back Pain (LBP) demonstrate a significant increasing incidence on adolescents [1]. Furthermore juvenile LBP is a risk factor for adult LBP [2]. In the Italian context there are no more researches about this topic.

The aim of this study is to investigate the influence of sport practice and other risk factors on adolescents’ LBP. Through a prevention program, we would verify if the incidence of this disease would change in the future. The project considers a sample of young basketball players aged between 8 and 11 years old.

**Materials and Methods:** The study was approved by the Ethic Committee of the University of Trieste (Italy) and parents’ informed consent was collected.

The sample consists of 57 children (43 M and 14 F) between 11 and 12 years old (mean-age 11.3; SD=0.45). The children followed two different programs of training. The study group was composed by 35 children (14F; 21M), mean-age 11.5 years (SD=0.5), and the control group of 22 children (22 M), mean-age 11 years.

The first assessment consisted in an anamnestic questionnaire (which also investigated on health, physic condition and sport participation of the children), Body Mass Index, observational postural assessment, photogrammetric assessment (through validated software PASS/SAPO), motor assessment (Hexagon’s test, side direction change test). After the first assessment, a specific Prevention Protocol was proposed to the the study group.

All the partecipants of both groups did 3 basketball trainings every week. In addition, children of the study group did the Prevention Protocol (30 minutes for week of: global active stretching, selective stretching, core stability/balance/proprioception exercises);

Partecipants have been assessed after a period of three months.

**Results:** At the first assessment the groups were statistically comparable for all the items we have considered (P value > 0.05 ).

At the 3 months assessment, the difference between the Lumbar-Pelvic Angle (LPA) of the groups is statistically significant (P value < 0.01). In the longitudinal analysis, after this short period of prevention, LPA of the study group has not been modified (P value=0.5056), while LPA of control group has changed with a significant negative difference (P value=0.0008).

**Conclusion:** The outcomes demonstrate a significant difference between groups, even if the Prevention Program has been proposed for a short period of three months. Especially we found a difference in the LPA: this is very important because evidences demonstrate the correlation (as risk factor) between LPA and LBP. The project is going on by increasing the sample with younger participants, and by focusing on the longitudinal analysis of the cases.

**References**

1. Balagué F, Burton AK, Cardon G, Eriksen HR, Hänninen O, Harvey EL, Henrotin Y, Lahad A, Leclerc A, Müller G, van der Beek AJ. European guidelines for prevention in low back pain. Eur Spine J. 2006; 15 (Suppl. 2): S136–S168

2. Harreby MS, Neergaard K, Hesselsøe G, Kjer J. [Are low back pain and radiological changes during puberty risk factors for low back pain in adult age? A 25-year prospective cohort study of 640 school children]. Ugeskr Laeger. 1997 Jan 6;159(2):171-4.

## P13 Definition of a cluster of evaluation scales for the identification in the elderly population of subjects at risk of fall-related fractures (scientific validation)

### Francesco Ciaghi, Davide Concato, Mauro Mazzurana

#### **Correspondence:** Francesco Ciaghi (franz.ciaghi@gmail.com)

**Background and Objective:** According to WHO data, between 28 and 35% of elderly people (65 years old or more) fall every year. Falls are limiting, risky and costly both for the person and the community. Furthermore, fractures that occur as a result entail significant yearly costs for the National Healthcare System.

**Materials and Methods:** The sample of the study was composed of patients of 6 Nursing Homes (RSA) of the Trentino region in Italy. Everyone could walk autonomously (with or without a walker), was 75 years or older and without cognitive damages or with a mild impairment (evaluated with the Mini Mental State Examination Test or Short Portable Mental Questionnaire). None of them had a psychiatric or atassic disease of cerebellar origin. Four Evaluation scales were used to evaluate the risk factors: Berg Balance, Tinetti, Morse Scale ed Hendrick Fall II Risk Model. We considered the total value and the single item value of every evaluation scale. After 6 months we conducted a follow-up to verify the number of fall events and the fall-related fractures.

**Discussion:** We identified 8 predictive items for the risk of falling and 2 predictive items for the risk of sustaining a fracture as a result of falling (p ≤ 0.05). These items are supported by the scientific literature and statistical data.

**Conclusion:** In order to reduce the costs of falls and of fall related fractures it is appropriate to work as a multi-disciplinary team and evaluate the patient with a cluster of evaluation scales.

**References**

1. World Health Organization. Ageing; Life Course Unit. WHO global report on falls prevention in older age. World Health Organization, 2008.

2. Deandrea S, Lucenteforte E, Bravi F, Foschi R, La Vecchia C, Negri E. Risk factors for falls in community-dwelling older people: a systematic review and meta-analysis. Epidemiology. 2010 Sep;21(5):658-68.

3. Shimada H, Suzukawa M, Ishizaki T, Kobayashi K, Kim H, Suzuki T. Relationship between subjective fall risk assessment and falls and fall-related fractures in frail elderly people. BMC Geriatr. 2011 Aug 12;11:4

## P14 Effects of combined cerebellar cortical stimulation and neurorehabilitation in chronic stroke patiens: a randomized double blind controlled repetitive TMS trial

### Alex Martino Cinnera^1^, Sonia Bonnì^1^, Elias P. Casula^1^, Viviana Ponzo^1^, Maria Concetta Pellicciari^1,2^, Michele Maiella^1^, Carlo Caltagirone^3^, Marco Iosa^4^, Stefano Paolucci^4^, Giacomo Koch^1,5^

#### ^1^Non Invasive Brain Stimulation Unit, Department of Behavioural and Clinical Neurology, Santa Lucia Foundation IRCCS, Rome, Italy; ^2^ Cognitive Neuroscience Section, Center San Giovanni di Dio Fatebenefratelli IRCCS, Brescia, Italy; ^3^ Department of System Medicine, Tor Vergata University, Rome, Italy; ^4^ Clinical Laboratory of Experimental Neurorehabilitation, Santa Lucia Foundation IRCCS, Rome, Italy; ^5^ Stroke Unit, Tor Vergata Policlinic, Rome, Italy

##### **Correspondence:** Alex Martino Cinnera (a.martino@hsantalucia.it)

**Background and Objectives:** The cerebellum is implicated in the functional reorganization of motor networks in stroke patients. It plays a critical role in promoting learning of new motor tasks, which is an essential function for motor recovery [1]. Motor learning can be potentiated by repetitive transcranial magnetic stimulation (rTMS) [2]. rTMS can be used to enhance adaptive processes and prevent those potentially maladaptive in stroke recovery[3]. In this randomized, sham-controlled study we aim to investigate the efficacy of cerebellar intermittent theta-burst stimulation (iTBS) coupled with physical therapy (PT) in promoting recovery of motor recruitment and balance functions in patients with hemispheric stroke.

**Materials and Methods:** 21 patients in the chronic stage of recovery (i.e. at least 6 months after stroke), with first ever-ischemic stroke in the territory of middle cerebral artery (8 females; 61±9.98 years) were recruited. Patients were randomly assigned to real-iTBS (n=11), or sham-iTBS (n=9). The iTBS stimulation was applied over the cerebellar hemisphere ipsilateral to the motor affected side, for fifteen days, immediately before starting the PT session. TMS-EEG and clinical evaluation ( Fugl-Meyer Assessment scale –FMA-; Berg Balance scale –BBS-; Barthel Index -BI-) were performed before (T0), immediately after (T1) and 15 days after T1 (T2).

**Results:** Real cerebellar iTBS produced a remarkable improvement in balance functions as compared to the sham condition (p = 0.02). In addition, we found that this improvement (T0 vs. T1; p=0.001) lasts until the follow-up (T0 vs. T2; p=0.001). Moreover combined iTBS-PT treatment increased postero-parietal-cortex (PPC) reactivity in T1 condition, compared to T0 (p=0.048). Finally enhancement of PPC reactivity significantly correlated with the improvement observed in the BBS score. Specifically, we observed higher PPC reactivity in presence of higher BBS score (r=.504; p=0.039).

**Conclusions:** Cerebellar iTBS coupled with PT drives a profound reorganization of cerebello-cortical networks by potentiating clinical recovery of balance accompanied by an enhancement of PPC reactivity and theta-range oscillations. These results suggest that cerebellar iTBS coupled PT may be an effective strategy in enhancing balance recovery in chronic stroke.

**References**

1. Dayan E, Cohen LG. Neuroplasticity subserving motor skill learning. Neuron. 2011 Nov 3;72(3):443-54.

2. Galea JM, Vazquez A, Pasricha N, de Xivry JJ, Celnik P. Dissociating the roles of the cerebellum and motor cortex during adaptive learning: the motor cortex retains what the cerebellum learns. Cereb Cortex. 2011 Aug;21(8):1761-70.

3. Koch G. Repetitive transcranial magnetic stimulation: a tool for human cerebellar plasticity. Funct Neurol. 2010 Jul-Sep;25(3):159-63.

## P15 Airway clearance in laryngectomy patient: Effective assessment of respiratory treatment with PEP Acapella system

### Marina Ciriello, Vincenzo Errico, Daniela Mondiello

#### Ospedali dei Colli -Monaldi-, Napoli, Italy

##### **Correspondence:** Marina Ciriello (marina.ciriello@ospedalideicolli.it)

**Background and Objectives**: The role of physiotherapy in cancer rehabilitation isn't well understood , particularly in head and neck cancer patients.

Head and neck cancer results in various residual deformities and dysfunctions. In fact there are many functional head and neck disorders after total laryngectomy and radiotherapy. This restricts chest and shoulder movements and also decreases lung ventilation, with an increasing of secretions in most patients who are ex-smokers. Secretion removal is a key issue in patients' rehabilitation after total laryngectomy, in addition to pain prevention, mobility improvement and lymphedema reduction. In total-laryngectomy patients is shown a progressive increase of bronchial obstruction and tracheal bacterial infection in the first year after the operation.

One of the most important prognostic factor regarding laryngectomy patients' survival is the progressive deterioration of pulmonary function and lung disease is the second leading causeof death of these patients.

The expiratory flow resistence, due to tracheostomy and its consequent early alveolar collapse, the loss of air filter and conditioning function with bronchial hypersecretion response and the cough mechanism alteration require a specific treatment for airway clearence.

The Acapella devices are used for secretion removal in daily clinical practice, but it has not been possible until now using them in laringectomy patients' treatment.

Therefore we have adapted the Acapella to the laringectomy patients' anatomical needs.

**Materials and Methods:** We administrated the clearence bronchial treatment for 12 weeks (7 in the Center and 5 of self administration) to 4 total laryngectomy ex-smokers patients, at the Regional Center of Reference for Rehabilitation of Head and Neck Oncological Pathology (Monaldi, Napoli), evaluated before and after spirometry (FEV1 and MEF 50%), VAS obstruction and VAS difficulty in expetoration.

**Graph 1 (abstract P15).**
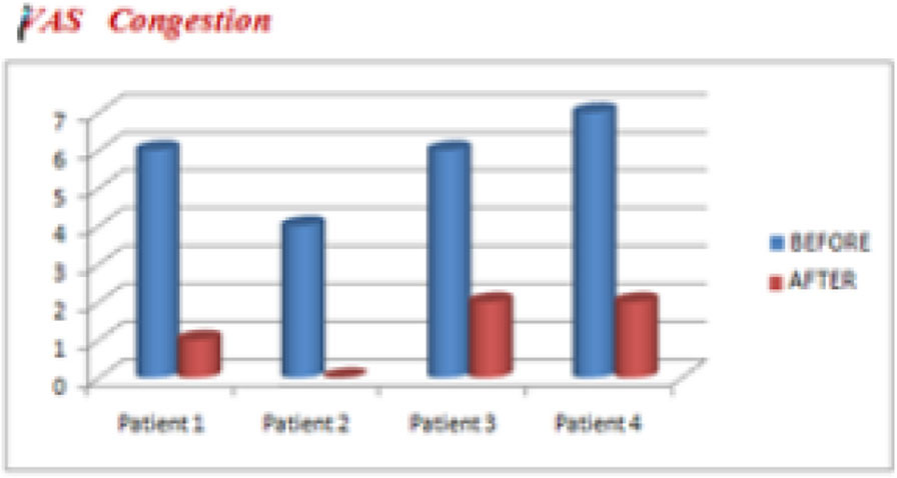
See text for description

**Graph 2 (abstract P15).**
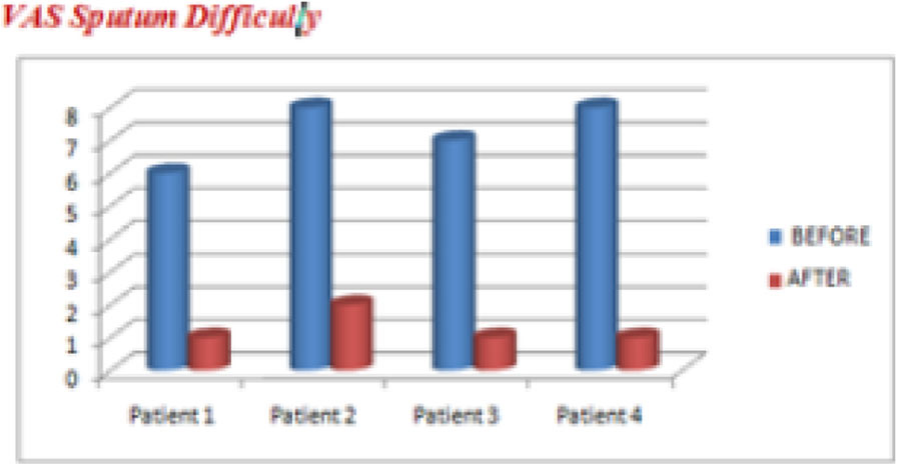
See text for description

**Results:** Results are showed in Table 1, Graph 1 and Graph 2.

**Conclusions:** In agreement with the literature [1-4], our study shows that patients' spirometric results after respiratory treatment don't

significantly change with the obstruction intervention, but there was a remarkable improvement in obstruction perception and difficulty in expectoration. These initial results are promising for future more methodologically appropriate investigation with wider statistics, maybe more useful if only performed on COPD patients with hyperesecretion (at greater risk of experiencing respiratory complications) and evaluating the possible reduction of exacerbation events in the long term.

**References**

1. Todisco T, Maurizi M, Paludetti G, Dottorini M, Merante F. Laryngeal cancer: long-term follow-up of respiratory functions after laryngectomy. Respiration. 1984;45(3):303-15.

2. Vázquez de la Iglesia F, Fernández González S. [Method for the study of pulmonary function in laryngectomized patients]. Acta Otorrinolaringol Esp. 2006 Jun-Jul;57(6):275-8.

3. Togawa K, Konno A, Hoshino T. A physiologic study on respiratory handicap of the laryngectomized. Arch Otorhinolaryngol. 1980;229(1):69-79.

4. Gregor RT, Hassman E. Respiratory function in post-laryngectomy patients related to stomal size. Acta Otolaryngol. 1984 Jan-Feb;97(1-2):177-83.

5. Castro MA, Dedivitis RA, Macedo AG. Evaluation of a method for assessing pulmonary function in laryngectomees. Acta Otorhinolaryngol Ital. 2011 Aug;31(4):243-7.

**Table 1 (abstract P15).** See text for description

	MEF 50%		FEV1%
	Before	After	Before
Patient 1	64	66	73
Patient 2	82	88	65
Patient 3	49	32	61
Patient 4	77	77	84

## P16 Effects of dance therapy in the patient with Parkinson’s disease

### Marina Ciriello^1^, Rosaria Cangiano^2^

#### ^1^AORN dei Colli, Napoli Italy; ^2^Clinic Center Napoli Italy

##### **Correspondence:** Marina Ciriello (marina.ciriello@ospedalideicolli.it)

**Background and Objective:** Dance could be a tool for physiotherapy in patients with Parkinson's disease it could be used to improve posture and body awareness, static and dynamic balance, fluidity in movement and coordination in space management, control of respiration, and it reduces stiffness and strengthens the cardiovascular, pulmonary and musculoskeletal system, also acting on social aspects, improving self-esteem and communication.

The KNGF guidelines recommend for these patients cognitive motor strategies, group treatments, and visual, acoustic, tactile, kinesthetic cues. Literature analysis suggests that the use of tango can improve the aspects of movement, measured with the UPDRS scale 3, the balance, measured with the Mini BESTest or Balance Scale Balance, and the gait, measured through the Timed Up and Go test. In addition, some studies [1] showed positive effects on fatigue, participation in activities and quality of life.

**Materials and Methods:** In this project 4 patients were recruited, they were treated for about six months with breathing, coordination, balance, stretching exercises, programmed gaits, functional activities and patient specific activities; after two months the dance was added, the first dance was the Sirtaki and then the Tango.

**Results:** The results are showed in Table 1.

**Conclusions:** The dynamic movement of dance, especially of Argentine tango, allows to find and conquer the right equilibrium together with the use of auditive and musical cues, allows to face the typical problems of bradycinesia

In particular, with the hug of tango, the dancer receives the stimulus for walking and movement from his partner, who in this case acts as an external peace-maker, replacing the "internal stimulus" compromised by the disease.

Dancing involves complex tasks, problem solving through increased mental engagement, motor strategy development and mirror neurons attivation

But tango-therapy also facilitates communication between participants, establishing interpersonal relationships relationships, the emergence of positive feelings originating from the feeling of belonging.

**References**

1. Lötzke D, Ostermann T, Büssing A. Argentine tango in Parkinson disease—a systematic review and meta-analysis. BMC Neurol. 2015 Nov 5;15:226

2. Foster ER, Golden L, Duncan RP, Earhart GM. Community-based Argentine tango dance program is associated with increased activity participation among individuals with Parkinson's disease. Arch Phys Med Rehabil. 2013 Feb;94(2):240-9.

3. Rios Romenets S, Anang J, Fereshtehnejad SM, Pelletier A, Postuma R. Tango for treatment of motor and non-motor manifestations in Parkinson's disease: a randomized control study. Complement Ther Med. 2015 Apr;23(2):175-84.

4. Duncan RP, Earhart GM. Randomized controlled trial of community-based dancing to modify disease progression in Parkinson disease. Neurorehabil Neural Repair.2012 Feb;26(2):132-43.

**Table 1 (abstract P16).** See text for description

Patient	Age	Pharmacological therapy	Initial assessment				Final evaluation		
			UPDRS (III) score	H&Y	Mini-BEST test	PDQ-39	UPDRS (III) score	H&Y	Mini-BEST test
A	71	Stalevo, Sirio, Sinem et	Limbs’s rigidity (2) sn>dx; remarkable postural instability (3)	3	13/28	21%	Limbs’s rigidity (1) sn>dx; remarkable postural instability (1)	3	20/28
B	80	Azilect, Sinem et	Very important rigidity & tremor to the right (3) postural instability (2)	3	18/28	18%	Rigidity dx (2) postural instability (1)	3	21/28
C	63	Azilect, Sinem et	Limbs’s rigidity (1) hand gesture deficit (2)	1.5	27/28	5%	Limbs’s rigidity (1) hand gesture deficit (1)	1.5	28/28
D	67	Madopal, Azilet, Mirapexil	Deambulation (1) Evident postural instability (3)	2.5	22/28	10%	Deambulation (1) Evident postural instability (1)	2.5	25/28

## P17 The effectiveness of aquatic therapy on the postural balance of elderly patients. A systematic revision

### Virginia Colibazzi¹, Davide Savini¹, Adriano Coladonato², Stefano Filippo Castiglia³, Roberta Mollica^4^, Emilio Romanini ^5^

#### ¹Equipe Terapeutica, Rome, Italy; ²ANIK Associazione Nazionale Italiana Idrokinesiterapisti, Italy; ³ NCL Neurological Center of Latium, Neuromed, Rome, Italy; ^4^ La Sapienza, University of Rome, Italy; ^5^ Artrogruppo, Rome,Italy

##### **Correspondence:** Virginia Colibazzi (virginia.colibazzi@gmail.com)

**Background and Objective:** Evidences emphasize the role of physical activity as a prevention factor for falls in elderly people, through the increase of muscular strength, aerobic abilities and balance. Thanks to water and to the low risk of the environment, aquatic therapy -therapeutic exercises that in water- can facilitate physical activities and balance exercises, improving also the elderly patients' compliance to the treatment.

The goal of this study is to evaluate the effectiveness of aquatic therapy on the postural balance of elderly patients as an alternative therapeutic proposal to conventional land based treatments.

**Materials and Methods:** The research was conducted between September 2015 and January 2016 on the main search engines. The following keywords were used: hydrotherapy, aquatic therapy, aquatic exercise, water rehabilitation, postural balance, falls; we selected RCT, Quasi-RCT, or RCCOT with a sample of subjects over 60 years old, and with at least an outcome measure concearning postural balance or the risk of falls.

Two independent reviewers evaluated the risk of bias using the Cochrane Collaboration's tool, and the methodological quality using the Pedro scale.

**Results and Discussion:** 9 studies met the eligibility criteria and underwent data mining and evaluation (7 RCT, 1 RCCOT and 1 Quasi-RCT), for a sample of 638 patients.

The methodological quality was good for both of the evaluation scales we had chosen; however, in the included studies there was a remarkable heterogeneity, in terms of the analyzed outcomes and in terms of the rehabilitative interventions and the evaluation tools. Such heterogeneity made impossible a quantitative synthesis and/or a meta-analysis.

The results seem to confirm the hypothesis according to which aquatic therapy is as effective as land based therapy in the improvement of physical and postural parameters, and even more effective for dynamic balance. The studies, moreover, attest an improvement of HRQoL, in the areas of vitality and social functions.

**Conclusion:** Aquatic therapy seems to be a safe and effective therapeutic proposal to improve the postural balance of elderly patients, in terms of functional and physical performances.

**References**

1. Crocker T, Forster A, Young J, Brown L, Ozer S, Smith J, Green J, Hardy J, Burns E, Glidewell E, Greenwood DC. Physical rehabilitation for older people in long-term care. Cochrane Database Syst Rev. 2013 Feb 28;(2):CD004294.

2. Panel on Prevention of Falls in Older Persons, American Geriatrics Society and British Geriatrics Society. Summary of the Updated American Geriatrics Society/British Geriatrics Society clinical practice guideline for prevention of falls in older persons. J Am Geriatr Soc. 2011 Jan;59(1):148-57.

3. Barker AL, Talevski J, Morello RT, Brand CA, Rahmann AE, Urquhart DM. Effectiveness of aquatic exercise for musculoskeletal conditions: a meta-analysis. Arch Phys Med Rehabil. 2014 Sep;95(9):1776-86.

4. Pendergast DR, Moon RE, Krasney JJ, Held HE, Zamparo P. Human physiology in an aquatic environment. Comprehensive physiology, 2015;5:1705-50

## P18 Action observation training modifies the function and structure of the mirror neuron system in multiple sclerosis patients with right upper limb motor deficits

### Claudio Cordani^1^, Maria Assunta Rocca^1,2^, Silvia Fumagalli^1,3^, Paolo Preziosa^1,2^, Roberto Gatti^3^, Filippo Martinelli-Boneschi^2^, Mauro Comola^2^, Giancarlo Comi^2^, Massimo Filippi^1,2^

#### ^1^Neuroimaging Research Unit, Institute of Experimental Neurology, Division of Neuroscience, San Raffaele Scientific Institute and Vita-Salute San Raffaele University, Milan, Italy; ^2^Department of Neurology, San Raffaele Hospital, Milan, Italy; ^3^Laboratory of Movement Analysis, Vita-Salute San Raffaele University, Milan Italy

##### **Correspondence:** Claudio Cordani (claudio.cordani@hrs.it)

**Background and Objective:** Applying structural and functional MRI techniques, we assessed the modifications of brain gray matter (GM) volumes, white matter (WM) architecture and patterns of activation of the mirror neuron system (MNS) following action observation training (AOT) in healthy controls (HC) and multiple sclerosis (MS) patients, and their correlations with improvement of motor performance.

**Materials and Methods:** Forty-six right-handed HC and 41 right-handed MS patients with right-hand motor impairment were randomized into: 2 experimental groups (HC-AOT n=23; MS-AOT n=20) and 2 control groups (HC-C n=23; MS-C n=21). Training consisted of 10 sessions of 45 minutes in 2 weeks. AOT-groups watched 3 videos of daily-life actions alternated by their execution with the right-hand; C-groups performed the same tasks, but watched landscapes videos. At baseline and after 2 weeks (w2), functional scales, brain structural (3D T1-weight and diffusion tensor sequences) and fMRI scans during object manipulation with the right hand were obtained.

**Results:** At w2, all groups improved at functional scales. Compared with C-groups, AOT-groups had more improvements at right-hand strength measures. At w2, no WM modifications occurred. At w2, HC-AOT vs HC-C experienced increased volume of the superior frontal gyrus (SFG) and decreased volume of fronto-temporal areas; at w2, MS-AOT vs MS-C had increased volumes of SFG, temporo-occipital areas and decreased volume of the supplementary motor area. At w2, HC-AOT vs HC-C had higher activation of the pre-central gyrus and lower activation of the middle temporal gyrus, while MS-AOT vs MS-C had higher activation of the inferior frontal gyrus. In MS-AOT, measures of functional improvement correlated with MRI modifications.

**Conclusions:** A 10-day AOT modifies GM structure and activations of motor network and MNS, promoting functional competence in HC and MS patients.

**References**

1. Karni A, Meyer G, Rey-Hipolito C, Jezzard P, Adams MM, Turner R, Ungerleider LG. The acquisition of skilled motor performance: fast and slow experience-dirven changes in primary motor cortex. Proc Natl Acad Sci USA. 1998 Feb 3;95 (3): 861-8.

2. Rizzolatti G, Craighero L. The mirror-neuron system. Annu Rev Neurosci. 2004;27:169-92

3. Ertelt D, Small S, Solodkin A, Dettmers C, McNamara A, Binkofski F, Buccino G. Action observation has a positive impact on rehabilitation of motor deficits after stroke. Neuroimage. 2007;36 Suppl 2:T164-73.

## P19 Structural MRI correlates of hand performance in patients with multiple sclerosis

### Claudio Cordani^1^, Maria Assunta Rocca^1,2^, Claudio Piazza^1^, Marco Roselli^1^, Federica Esposito^2^, Marta Radaelli^2^, Bruno Colombo^2^, Giancarlo Comi^2^, Massimo Filippi^1,2^

#### ^1^Neuroimaging Research Unit, Institute of Experimental Neurology, Division of Neuroscience, San Raffaele Scientific Institute, Vita-Salute San Raffaele University, Milan, Italy; ^2^Department of Neurology, San Raffaele Hospital, Milan, Italy

##### **Correspondence:** Claudio Cordani (cordani.claudio@hsr.it)

**Background and Objective**: We applied structural MRI techniques in a large cohort of Multiple Sclerosis (MS) patients to evaluate the correlation between abnormalities of regional brain gray matter (GM) volumes and white matter (WM) architecture and measures of manual dexterity and Expanded Disability Status Scale (EDSS).

**Materials and Methods:** From 134 healthy control (HC) and 366 right-handed MS patients, brain 3D T1-weighted and diffusion tensor (DT) MRI scans were acquired and used to performed a Voxel-based Morphometry and a Tract-based Spatial Statistic. Correlations between altered MRI measures and EDSS as well as manual dexterity tests [9 Hole Peg Test (9HPT) and Finger Tapping (FT) test] were investigated.

**Results:** Compared with HC, MS patients show a widespread pattern of GM atrophy involving the frontal, parietal and occipital lobes. The analysis of WM architecture showed a distributed reduction of fractional anisotropy (FA) and an increased axial (AD), radial (RD) and mean diffusivity (MD) in MS patients compared to HC. In MS patients, better performance at 9HPT correlated with higher volume of the putamen, insula and cerebellum, whereas lower 9HPT performance correlated with R cerebellum atrophy. Better FT performance correlated with higher left superior temporal gyrus volume, whereas higher EDSS correlated with atrophy of the cerebellum, temporal lobe and putamen. Finally, a negative correlation between reduced FA and increased AD, RD and MD with worse manual dexterity performances was found.

**Conclusions:** Tissue loss and microscopic tissue abnormalities of the cerebellum and deep GM structures contributes to explain motor dysfunction in patients with MS.

**References**

1. Losseff NA, Wang L, Lai HM, Yoo DS, Gawne-Cain ML, McDonald WI, Miller DH, Thompson AJ. Progressive cerebral atrophy in multiple sclerosis. A serial MRI study. Brain. 1996 Dec;119 (Pt6):2009-19.

2. Onu M, Roceanu A, Soboto-Frankenstein U, Bendic R, Tarta E, Preoteasa F, Bajenaru O. Diffusion abnormality maps in demyelinating disease: correlations with clinical scores. Eur J Radiol. 2012 Mar;81(3):e386-91

3. Bodini B, Khaleeli Z, Cercignani M, Miller DH, Thompson AJ, Ciccarelli O. Exploring the relationship between white matter and gray matter damage in early primary progressive multiple sclerosis: An in vivo study with TBSS and VBM. Hum Brain Mapp .2009 Sep;30(9):2852-61

## P20 Aquatic therapy after rotator cuff surgery: when to start? A study about 18 patients in two different protocols

### Lucia Coppola^1^, Carlo Lollo^2^, Anna Chiara Frigo^3^, Marco Caia^4^, Giorgio Granzotto^4^, Francesca Gattinoni^5^

#### ^1^Physical therapist, AULSS 6 Euganea, Padova, Italy; ^2^ Physical therapist, Treviso, Italy; ^3^ MSC, Dipartimento di Scienze Cardiologiche Toraciche e Vascolari, University of Padova, Italy; ^4^ Physical therapist, adjunct professor of Physiotherapy degree Course, University of Padova, Italy; ^5^ MD; Direttore dell'U.O.C. Medicina fisica e Riabilitazione, AULSS 2 Marca Trevigiana, Distretto di Pieve di Soligo, Italy

##### **Correspondence:** Lucia Coppola (lucia.coppola@unipd.it)

**Background and Objectives**: Aquatic therapy is commonly prescribed after rotator cuff surgery. The aim of this paper is to define whether aquatic therapy should start 20 or 35 days after surgical intervention.

**Materials and Methods:** 18 patients (9 from Vittorio Veneto, 9 from Conegliano) which underwent repair of the rotator cuff by the same medical equipe were evaluated starting in February 2016 and ending in June 2016. All of the patients followed the same rehabilitation protocol though in different moments (20 days after surgical operation in Conegliano, 35 days after in Vittorio Veneto [Table 1]). None of them showed any sign of other related disfunctions. Patients were evaluated through Visual Analogic Scale and Constant scale at 20 days (t0), 35 days (t1) and 50 days (t2) days. Data processing was conducted via SAS 9.4 for Windows. Fisher's exact test was adopted to evaluate qualitative variables, while Wilcoxon test was adopted to analyze quantitative variables.

**Results:** 9 patients (2F/ 7M) were recruited at Conegliano hospital, average age was 56 (test group). 9 patients (3F/ 6M) were recruited at Vittorio Veneto hospital, average age was 65 (control group). At t 0 pain and disability were similar in both groups. The test group resulted in obtaining a better improvement in almost all sections of Constant Scale, even though the related p-values weren't statistically relevant. The improvement in disability is statistically relevant and is greater in the test group (p<0,0172).

**Conclusions:** Patients undergoing surgical repair of the rotator cuff may start aquatic therapy with benefit 20 days after surgical intervention.

**References**

1. Coppola L, Masiero S. Riabilitazione in ortopedia. Piccin, 2005.

2. Gallagher BP, Bishop ME, Tjoumakaris FP, Freedman KB. Early versus delayed rehabilitation following arthroscopic rotator cuff repair: A systematic review. Phys Sportsmed. 2015 May;43(2):178-87.

3. Thomson S, Jukes C, Lewis J. Rehabilitation following surgical repair of the rotator cuff: a systematic review. Physiotherapy. 2016 Mar;102(1):20-8.

4. Zanazzo M, De Ruvo F, Efficacia dell’idroterapia negli esiti di riparazione della cuffia dei rotatori. Gazzetta Medica Italiana Archivio per le Scienze Mediche. 2014;173(11):539-45.

**Table 1 (abstract P20).** See text for description

Rehabilitation protocol in different rehabilitative structures	
CONEGLIANO	
Medical Assessment	
Physician assessment	
1° rehabilitative intervention (10 sessions)	
2° rehabilitative intervention Individual physiotherapy + aquatic therapy 20 alternate sessions	
Physician assessment	

## P21 Effects of taping for the treatment of shoulder impairments after stroke: systematic review

### Lorenzo D'Agostino^1^, Matteo Paci^2^

#### ^1^Private practice, Firenze, Italy; ^2^Unit of Functional Rehabilitation, Azienda USL Toscana Centro, Prato, Italy

##### **Correspondence:** Matteo Paci (matteo.paci@sif-fisioterapia.it)

**Background and Objectives:** Tape for shoulder impairments in hemiplegic patient is commonly used in clinical practice [1], but published systematic reviews did not report clear results. The purpose of this review is to examine the effectiveness of taping for shoulder impairments in hemiplegic patients.

**Materials and methods:** A literature search was performed through three databases until February 2017. The results were compared using the weighted mean difference WMD). Reported quality was assessd by PEDro score [2].

**Results:** Seven studies (410 participants) were included in the quantitative analysis (PEDro score: median = 6; range = 5-8). Tape application for the treatment of shoulder pain after stroke seems to have effects in terms of motor function (WMD: 1.24; 95% CI: 0.41-2.07) and pain reduction (WMD: -1.98; 95% CI: -3.45- -0.51) when compared with no treatment. A weak effect on muscle tone was found, when compared with placebo treatment (WMD: 0.43; 95% CI: 0.01-0.87). No additional effect was found.

**Conclusion:** Despite the methodological quality of the studies, the limited number of controlled randomized trials and the heterogeneity of the application techniques suggest to interpret results with coution. It is necessary to investigate the potential mechanisms underlying the tape application in order to standardize application modes. Further studies are needed to confirm the results.

**References**

1. Appel C, Perry L, Jones F. Shoulder strapping for stroke-related upper limb dysfunction and shoulder impairments: systematic review. NeuroRehabilitation. 2014;35(2):191-204.

2. Moseley AM, Herbert RD, Sherrington C, Maher CG. Evidence for physiotherapy practice: a survey of the physiotherapy evidence database (PEDro). Aust J Physiother. 2002; 48: pp. 43-49.

## P22 Feasibility of different Nintendo Wii video games for balance training in GMFCS Level III-IV children

### Chiara Degl’Innocenti^1^, Silvia Paoli^2^, Silvia Camici^3^

#### ^1^Fisioterapista, specialista in area pediatrica, Libero Professionista; ^2^Fisioterapista, specialista in area pediatrica, Coord. didattico Master di I livello Fisioterapia Pediatrica, Università degli Studi di Firenze; ^3^Fisioterapista, specialista in area pediatrica, Servizio di riabilitazione dell’ASL centro sede di Prato

##### **Correspondence:** Chiara Degl’Innocenti (chiarin6@gmail.com)

**Background and Objective:** Studies including the use of Nintendo Wii video games as a rehabilitation tool for balance and selective motor control focused primarily on children with GMFCS level I and II. We therefore proposed to map video games for Nintendo Wii and test their feasibility in children with neuromotor disorders and level III-IV of GMFCS.

**Materials and Methods:** Children from 6 to 18 years old and III-IV level of GMFCS were recruited from Prato Rehabilitation Service. Exclusion criteria were: orthopedic surgery within the previous 6 months and presence of sensory or cognitive impairments incompatible with the study proposal. Each game was initially tested by a physiotherapist and categorized in terms of difficulty. Each child played every suitable game for a maximum of 3 times. A game was considered unmanageable if children failed all three attempts or if the subject did not meet the requirements for execution. Achievement of the game’s goal, location of game session, and facilitations made by the experimenter were collected along with the reports of overall pleasure experienced.

**Results:** 53 games were proposed to 8 subjects (5 GMFCS III, 3 GMFCS IV). 21 games were accessible and suitable to all children. The use of adaptations (verbal and manual guidance, physical contact, support, aids and orthoses) and varying the playing position (sitting, kneeling, standing) enhanced the feasibility of games.

**Conclusion:** Many Nintendo Wii games can be offered to children with high levels of functional disability (GMFCS III and IV). The study has made it possible to build a small guide containing information useful for personalizing this therapeutic proposal that could also be performed at home for children in charge in Territorial Services. Further studies would be helpful to evaluate and compare different consoles potentialities as rehabilitation tools within GMFCS level III and IV children.

## P23 Two cases-study of different Nintendo Wii video games for balance training in GMFCS Level III children

### Chiara Degl’Innocenti^1^, Silvia Paoli^2^, Silvia Camici^3^

#### ^1^Fisioterapista, specialista in area pediatrica, Libero Professionista; ^2^Fisioterapista, specialista in area pediatrica, Coord. didattico Master di I livello Fisioterapia Pediatrica, Università degli Studi di Firenze; ^3^Fisioterapista, specialista in area pediatrica, Servizio di riabilitazione dell’ASL centro sede di Prato

##### **Correspondence:** Chiara Degl’Innocenti (chiarin6@gmail.com)

**Background and Objective:** A small number of studies focused on Nintendo Wii console video games as a rehabilitaion tool in children with neurodisabilities, most of which considered only GMFCS I and II level subjects. Given the encouraging results of these studies and aware of the strong impact that using this technology have on motivation, we proposed to evaluate whether more severe children could also benefit from specific training mediated by this technology.

In particular, we try to evaluate the effect on postural control and the balance of a 2 month bi-weekly training with a selection of rated and suitable video games within GMFCS level III subjects

**Materials and Methods:** Children from 6 to 18 years old and III-IV level of GMFCS were recruited from Prato Rehabilitation Service. Exclusion criteria were as follows: orthopedic surgery within the previous 6 months, presence of sensory or cognitive impairments incompatible with the study proposal. Two subjects underwent an initial (V0) and final (V1) evaluation after 2 months of treatment. At the end of the initial evaluation (V0) a target activities was selected according to Goal Attainment Scale (GAS) which was agreed upon with the child and the family. The outcome measure selected for the first subject evaluation were: Gross Motor Function Measurement (GMFM), D and E sections, Pediatric Balance Scale (PBS) and Pediatric Reach Test (PRT). For the second subject were used: the Sitting Assessment for Children with Neuromotor Dysfunction (SACND) and the Pediatric Reach Test (PRT). In each session 5 games were proposed (Fish Hunt, Slalom, Headshot, Snowboard and Crazy Balls), each to be repeated 3 times interrupted by a 1-2 minute break. Some games have been offered in sitting or kneeling.

**Results:** Both children benefitted from the training which has produced positive changes in all outcome measure especially in the Pediatric Reach Test.

**Conclusions:** The use of Nintendo Wii video games can be a valuable tool for developing treatments that help improve posture control and balance in children with neuromotor disorders.

## P24 The E.S.A.C.C. Rehabilitation technique for treating prevalently cervicogenic equilibrium disorders

### Maria Domanico (maria.domanicosi@libero.it)

#### Physiotherapist at Niguarda Hospital until 31\12\2016

**Background and objective:** The E.S.A.C.C. technique (Elasticizzazione, Scollamento, Allenamento, Calore, Carrucola = elasticization, detachment, training, heat, pulley) consists in a combination of detachment actions for treating trigger points, repeated active movements, suspension exercises with pulley and exogenous heat. This summary illustrates the E.S.A.C.C. rehabilitation technique, worked out and devised in balance disorders of cervical origin mostly caused by wrong postures over time and by distorsive traumas. Symptomatology is characterized by pains, contractures, articular blockage of the cervical-dorsal section, migraine, dizziness, tinnituses, instability, empty head feeling, vertigoes, nausea and/or vomit. The E.S.A.C.C. technique aimed to restoring soft tissues elasticity, recovery of muscle and tendon functions and of interferences with associated structures, biomechanical rebalancing and reprogramming CNS central mechanisms of integration and elaboration.

**Materials and methods:** For the purpose of this research, in cooperation with the medical division of the Physical Therapy and Rehabilitation Unit of the Niguarda Hospital in Milan, a sample of 106 patients was involved between 2000 and 2001. All subjects showed negativity on the neurological examination, objective signs of cervical involvement and positivity on balance assessment during the retroflexed head test, with a significant index of cervical interference for a participation of the cervical proprioception component. Patients underwent segmental and regional examination of the cervical rachis, balance examination in the three standard conditions and were given a survey on subjective parameters, at the beginning and at the end of the E.S.A.C.C. therapy.

**Results:** After being treated with the E.S.A.C.C. therapy, 74.5% of patients presented a normalization of balance parameters together with a recovery of subjective symptoms; the other 25.5% showed an improvement of subjective symptoms but not a normalization of balance parameters.

**Conclusions:** The E.S.A.C.C. technique is an evolving therapeutic strategy. Its principles await further experimentation in new physiotherapy areas.

**References**

1. Cossu M, Rega V, Domanico M. Revisione clinica delle sindromi disfunzionali dello stretto toracico Parte II: proposta riabilitativa, casistica e risultati. Riabilitazione-Milan. 1997;30:3-10.

2. Domanico M. Sias N. Cossu M. Una nuova proposta riabilitativa per i disturbi dell’equilibrio di origine cervicale: la tecnica E.S.A.C. La Riabilitazione. 2001;34(1):35-40.

3. Cossu M, Crimaldi S, Rossi L, Domanico M. The Correlation between Stabilometry and the Dizziness Handicap Inventory in the Evaluation of the Effectiveness of ESAC Treatment. La Riabilitazione. 2002/1

## P25 Effectiveness of Pain Neurophysiology Education in chronic low-back pain: a review

### Matteo Fascia, Paolo Bizzarri

#### **Correspondence:** Matteo Fascia (matteo.fascia1991@libero.it)

**Background and Objectives:** Chronic Low-Back Pain (CLBP) represents a complex multifactorial phenomenon, significantly interfering with social and working life. Different educational and counseling interventions are widely employed in the CLBP management. Among these interventions, Pain Neurophysiology Education (PNE) represents a promising approach. It is based on the explanation of the neurophysiology processes underlying the painful experience of the patient, positively affecting symptoms, physical performance and therapy expectations. Aim of this study was to review the consistency and effectiveness of the PNE in the CLBP treatment, both as an isolated therapy and as a part of others.

**Materials and Methods:** MEDLINE, PEDro, Google Scholar, and the Cochrane Reviews databases were searched. Used keywords were: pain, chronic pain, education, neurobiology, low back pain, pain neurophysiology education, neuroscience. Studies published after 2002 and written in English or Italian languages were included. Studies were clinical trials, systematic reviews, or meta-analyses that involved subjects with CLBP treated by using PNE as an isolated therapy or in addition to other therapies. No limitations on the outcome measurements.

**Results:** A total of 12 studies were selected: 2 systematic reviews, 2 systematic reviews with meta-analyses, 7 RCTs and 1 non-randomized clinical trial. These studies reported the effectiveness of the PNE respect to the traditional education. Given the evidence available, it is believed that the PNE has to be included in a multimodal approach to pain management.

**Conclusions:** PNE as an isolated therapy seems effective in reducing fear of movement, catastrophizing, and false beliefs about pain. However, this practice is not as much efficient in relieving pain intensity and perceived disability, and the lack of long-term follow-up in the reviewed studies hampers the possibility to evaluate the patients’ ability in preserving the learnt advices and the presence of true and long-lasting variations in pain perception and behavior. Although the growing interest towards this discipline, further researches are needed to examine in depth the effectivenss of the PNE and to devise a set of clinical practice recommendations.

## P26 Temporomandibular disorders: from diagnostic criteria to neuroscience. A narrative review

### Ferrara Daniele^1^, Alessandro Agostini^2^

#### ^1^Università degli Studi di Roma Tor Vergata, Facoltà di Medicina e Chirurgia, Master in Terapia Manuale applicata alla Fisioterapia; ^2^Università degli Studi di Roma Tor Vergata. Facoltà di Medicina e Chirurgia, Master in Terapia Manuale applicata alla Fisioterapia, Medicina del dolore

##### **Correspondence:** Ferrara Daniele (daniele.ferrara.fkt@email.it)

**Background and Objectives:** Temporomandibular Disorders (TMD) represent a heterogeneous set of stomatognatic system pathologies that embrace a number of disorders involving chewing musculature, temporomandibular joint and associated structures [1]. The complexity and multifactoriality of this bio-psycho-social disorder require a specific clinical approach [2]. Aim of this study was to carry out a narrative review in order to understand i) the risk factors for developing a TMD, ii) the latest and updated knowledge of neuroscience associated with TMD, and iii) the modern clinical implications of treatment.

**Material and Methods:** A review was carried out searching on PubMed. Inclusion criteria and search limits were: publication of the last 10 years, studies conducted on humans aged 19 to 44 years, English-language articles, and abstracts availability. Exclusion criteria were: studies involving psycho-social disorders associated with musculoskeletal dysfunctions of other districts, disorders associated with cancer, psycho-social disorders, disorders associated with prosthetic implantology, somatization processes and risk factors in Psychiatric patients. The selection of the studies was carried out on the basis of the title, the abstract and then the complete reading of the article.

**Results:** Of the 470 records identified by the search strategy, 18 articles were included and reviewed.

**Conclusions:** TMDs represent a complex set of etiology disorders resulting from the interaction of multiple genetic and environmental factors. The risk factors for developing a TMD were 3: the state of health, the psychological, and the orofacial factors (Figure 1). Minor contribution comes from socio-demographic dominance, sensitivity to pain, and autonomic functions [3]. Patients could be also clustered in 3 specific categories with evidence of response to treatment: adaptive, pain sensitivity, and global symptoms [4]. Finally, neuroscience seems to include in the etiology of TMD the formation of precise neuro-plastic highways at the level of the limbic and trigeminal system, with cortical changes occurring in the thalam, in the anterior and median cortical cortex and in the premotory cortex. From a functional point of view, this translates into an altered processing of cognitive, attentive and emotional information with neural pathways modified by peripheral and central disregulation [5].

**References**

1. Randhawa K, Bohay R, Côté P, et al. The effectiveness of noninvasive interventions for temporomandibular disorders: A systematic review by the Ontario Protocol for Traffic Injury Management (OPTIMa) Collaboration. Clin J Pain. 2016;32(3):260-78.

2. Dıraçoǧlu D, Yıldırım NK, Saral İ, et al. Temporomandibular dysfunction and risk factors for anxiety and depression. J Back Musculoskelet Rehabil. 2016;29(3):487-91.

3. Slade GD, Fillingim RB, Sanders AE, et al. Summary of findings from the OPPERA prospective cohort study of incidence of first-onset temporomandibular disorder: implications and future directions. J Pain. 2013;14(12 Suppl):T116-24.

4. Slade GD, Ohrbach R, Greenspan JD, et al. Painful temporomandibular disorder: decade of discovery from OPPERA Studies. J Dent Res. 2016;95(10):1084-92.

5. Ichesco E, Quintero A, Clauw DJ, et al. Altered functional connectivity between the insula and the cingulate cortex in patients with temporomandibular disorder: a pilot study. Headache. 2012;52(3):441-54.

**Fig. 1 (abstract P26).**
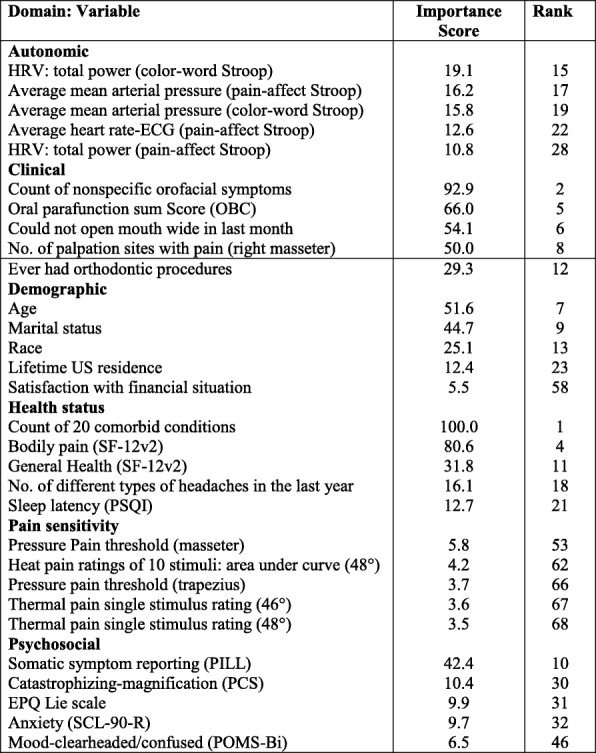
Risk factors for developing TMDs

## P27 Psycho-social process underlying motivations to participate in a research study: a grounded theory study in patients with non-small cell lung cancer

### Filippo Ferrari^1^, Luca Ghirotto^1^, Chiara Montermini^2^, Roberta Bardelli^3^, Carlotta Mainini^4^, Stefania Fugazzaro^4^, Stefania Costi^3^

#### ^1^Student of Physiotherapy; Department of Biomedical, Metabolic and Neural Sciences, University of Moden and Reggio Emilia, Modena Italy; ^2^ Scientific Direction of Istituto di Ricerca e Cura a Carattere Scientifico - Arcispedale Santa Maria Nuova, Reggio Emilia, Italy; ^3^Professor of the Department of Biomedical Sciences, metabolic and neural, University of Modena and Reggio Emilia, Modena Italy; ^4^Unit of Physical and Rehabilitation Medicine, Istituto di Ricerca e Cura a Carattere Scientifico - Azienda Unità Sanitaria Locale, Reggio Emilia, Italy

##### **Correspondence:** Filippo Ferrari (filippoferrari23@gmail.com)

**Background and Objective:** Non-small cell lung cancer (NSCLC) represents 85% of lung cancers, and no standardized and well-studied rehabilitation approaches are available [1]. The willingness to participate in an experimental study and treatment compliance are critical issues that emerged in the conduct of clinical research, also in the rehabilitation field [2]. Aim of this study was to analyze the psycho-social process that occurs when it is proposed to patients with NSCLC to participate in a rehabilitation research project, and what brings them to join that.

**Materials and methods:** This was a Grounded Theory qualitative study, part of a larger project (PuReAIR) aimed to analyze the effectiveness of a rehabilitative intervention in patients with NSCLC that is currently in place in the AUSL-IRCCS of Reggio Emilia. Subjects were recruited among those participating in the PuReAIR project, and subsequent snowball sampling was adopted. A semi-structured interview was used to investigate patients experience. Data were encoded by constructing of conceptual categories to build a theory.

**Results:** A total of 9 subjects were included in this study. The analysis of the data revealed that the investigated process is based on two main categories: i) trust in science and ii) in the subject that proposes the study, reinforced by a strong perception of the established therapeutic relationship with the operators -in the foreground the Physiotherapists- and fed by the positive feedback.

**Conclusions:** The proposal to participate in an experimental rehabilitative treatment, advanced immediately after the diagnosis of cancer, was welcomed by the patients. Being able to take advantage of a new therapy opportunity, that does not involve risks and that is perceived as help for oneself and others, are important elements for the patient, who can help in the decision to adhere to the experimentation.

**References**

1. Nici L. The role of pulmonary rehabilitation in the lung cancer patient. Semin Respir Crit Care Med. 2009;30(6):670-4.

2. Wright JR, Whelan TJ, Schiff S, et al. Why cancer patients enter randomized clinical trials: exploring the factors that influence their decision. J Clin Oncol. 2004;22(21):4312-8.

## P28 Factors associated with citation rate of systematic reviews in physiotherapy

### Virginia Fidi^1^, Matteo Paci^2^

#### ^1^Private practice, Firenze, Italy; ^2^Unit of Functional Rehabilitation, Azienda USL Toscana Centro, Prato, Italy

##### **Correspondence:** Matteo Paci (matteo.paci@sif-fisioterapia.it)

**Background and Objective:** The use of citation rate as a measure of quality of a stydy is a very criticized method, but it is the most used to assess the performance of researchers, articles and journals [1]. It is also believed that, in order to measure the impact of an article, the number of quotes it receives should be associated with its methodological qualities and the relevance of the subject being discussed [2]. The purpose of this study is to detect which factors are associated with the citation rate of systematic reviews published in physiotherapy.

**Materials and Methods:** Articles indexed on the PEDro and Scopus databases in 2010 were selected. The following independent variables were recorded: language of publication, indexing in PubMed database, type of access to articles (open access, delayed open access or restricted access), sub-discipline, 5 years Impact factor of journals where the articles were published, number of authors, country where the study was conducted and to be a Cochrane review. The citation rate until December 2015 was considered as dependent variable. Data were analysed using a stepwise multiple regression model.

**Results:** A total of 436 articles were extracted, 68 were excluded, and 368 articles were analyzed on the PEDro database as well as on Scopus. From the data analysis it was noted that the factor most associated with the number of citations was the IF on 5 years (β = 0.314) explained 5.6% of variance (adj R2 = 0.056), followed by a Cochrane review (β = - 0.246) explaining additional 5.1% of variance (adj R2 = 0.107) and finally with the association with the number of authors (β = 0.181) explaining additional 0.3% of variance (adj R2 = 0.137).

**Conclusions:** The study showed that the Impact Factor over 5 years, the fact of not being a Cochrane review and the number of authors are the predictors associated with the number of quotes of systematic reviews. All of these results explain a small part and should be analyzed over a longer period of time.

**References**

1. Radicchi F, Fortunato S, Castellano C. Universality of citation distributions: toward an objective measure of scientific impact. Proc Natl Acad Sci U S A. 2008;105(45):17268-72.

2. Paci M, Landi N, Briganti G, Lombardi B. Factors associated with citation rate of randomised controlled trials in physiotherapy. Arch Physiother. 2015;5:9.

## P29 Use of normocapnic hyperpnoea in treating of thoracic musculoskeletal disorders. A single subject design

### Guglielmo Formichella^1^, Leonardo Ciampoli^2^

#### ^1^Studio di Fisioterapia di Guglielmo Formichella, Sant’Agnello (Na), Italy; ^2^University of Rome “Tor Vergata”, Rome, Italy

##### **Correspondence:** Guglielmo Formichella (gugfor@gmail.com)

**Background and Objective:** Patients with thoracic musculoskeletal pain may beneficiate of Respiratory Muscles Endurance Training (RMET) and Functional Respiratory Stretching (SRF). Both these therapeutic approaches have important implications on physiological mechanisms, increased load capacity in daily life activities and/or sports [1-3]. This article will describe the treatment with respiratory training with normocapnic hyperpnoea in a patient with muscular thoracic pain related to motor dysfunction of the respiratory act.

**Materials and Methods:** Study design: single subject design. First, a review on the PubMed database was performed (May, 2017) to identify studies that used RMET and SRF approaches in patients with thoracic musculoskeletal pain. According to literature, a treatment with respiratory training in normocapnic hyperpnoea was then planned alternating 2 cycles of 2 weeks of treatment and 2 weeks without treatment. Outcome measures were clinical (Numeric Pain Rating Scale -NPRS- for pain intensity and Patient-Specific Functional Scale –PSFS- for load capacity) and instrumental (Spinal Mouse to measure trunk flexion, and Spirometer to measure the respiratory performance).

**Results:** Patient expiration mobility improved after treatment, accompanied by FEV1 increase in spirometric examination. Clinical measures showed pain resolution (NPRS=0) and increased load capacity (PSFS).

**Discussion**: The results of this study supported the evidence available on previous studies [4-5], showing the close relationship between thoracic biomechanics and respiratory patterns and load capacity, between motor control and functional overload pain.

**Conclusion:** Musculoskeletal thoracic pain is a challenge for the close relationship with the body's functions that can be affected by the dysfunction. The results of this study encourages further research, such as assessment of multimodal treatment.

**References**

1. Briggs AM, Smith AJ, Straker LM, Bragge P. Thoracic spine pain in the general population: prevalence, incidence and associated factors in children, adolescents and adults. A systematic review. BMC Musculoskelet Disord. 2009;10:77.

2. Lee DG. Biomechanics of the thorax - research evidence and clinical expertise. J Man Manip Ther. 2015;23(3):128-38.

3. Bradley H, Esformes J. Breathing pattern disorders and functional movement. Int J Sports Phys Ther. 2014;9(1):28-39.

4. Bernardi E, Pomidori L, Bassal F, Contoli M, Cogo A. Respiratory muscle training with normocapnic hyperpnea improves ventilatory pattern and thoracoabdominal coordination, and reduces oxygen desaturation during endurance exercise testing in COPD patients. Int J Chron Obstruct Pulmon Dis. 2015;10:1899-906.

5. González-Álvarez FJ, Valenza MC, Torres-Sánchez I, Cabrera-Martos I, Rodríguez-Torres J, Castellote-Caballero Y. Effects of diaphragm stretching on posterior chain muscle kinematics and rib cage and abdominal excursion: a randomized controlled trial. Braz J Phys Ther. 2016;20(5):405-411.

**Fig. 1 (abstract P29).**
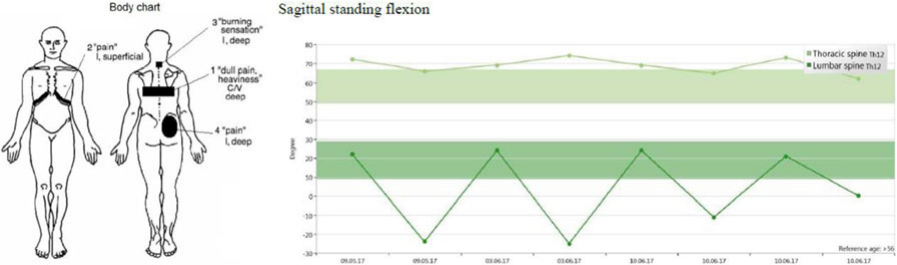
Localisation of symptoms (left) and thoracic mobility values during the monitored period (rigth)

## P30 The effect of physiotherapy on fatigue and physical functioning in chronic fatigue syndrome patients: A systematic review

### Giovanni Galeoto^1^, Roberta Mollica^2^,Valter Santilli^2^, Annamaria Servadio^3^

#### ^1^Department of Public Health, Sapienza University of Rome; ^2^Department of Anatomical, Histological, Forensic and Orthopedic Sciences, “Sapienza” University of Rome, Italy; ^3^Department of Health Professions, Policlinico “Tor Vergata” of Rome, Italy

##### **Correspondence:** Giovanni Galeoto (giovanni.galeoto@uniroma1.it)

**Background and Objective:** The objectives of this work were to fill this gap in the scientific literature and to evaluate the results of physical therapy treatments in individuals affected by chronic fatigue syndrome, looking only at studies that employed a randomized controlled trial

**Materials and Methods:** A systematic review was carried out according to PRISMA guidelines. Three bibliographic databases were searched: MEDLINE, Cochrane Library, and PEDro. The minimal prerequisites for papers to be included in the systematic review were that they had to (a) employ a randomized controlled trial; (b) be published in English; and (c) be published during the last ten years (2007–2017). The studies were evaluated according to their Jadad score [1].

**Results:** Five studies were included. This systematic review suggests that a treatment that is more efficient than all the others cannot be defined. This conclusion is related to the low number of investigated studies; therefore, the collected results cannot be generalized [2-5].

**Conclusion:** Chronic fatigue syndrome is not yet a well-understood pathology, and the physical mechanisms that influence the outcomes still need more study. Rehabilitation programs that promote physiotherapy techniques such as exercise, mobilization, and body awareness (e.g., MRT and GET) are the most effective in reducing medium and long-term fatigue severity in CFS patients.

**References**

1. Clark HD, Wells GA, Huët C, McAlister FA, Salmi LR, Fergusson D. Assessing the quality of randomized trials: reliability of the Jadad scale. Control Clin Trials. 1999; 20(5), 448-52.

2. Gordon BA, Knapman LM, Lubitz L.Graduated exercise training and progressive resistance training in adolescents with chronic fatigue syndrome: a randomized controlled pilot study. Clin Rehabil. 2010;24(12):1072-9.

3. Núñez M, Fernández-Solà J, Nuñez E, Fernández-Huerta JM, Godás-Sieso T, Gomez-Gil E. Health-related quality of life in patients with chronic fatigue syndrome: group cognitive behavioural therapy and graded exercise versus usual treatment. A randomised controlled trial with 1 year of follow-up. Clin Rheumatol. 2011;30(3), 381-89.

4. White PD, Goldsmith KA, Johnson AL, Potts L, Walwyn R, DeCesare JC. Comparison of adaptive pacing therapy, cognitive behaviour therapy, graded exercise therapy, and specialist medical care for chronic fatigue syndrome (PACE): a randomised trial. The Lancet. 2011;377(9768):823-36.

5. Fukuda K, Straus SE, Hickie I, Sharpe MC, Dobbins JG, Komaroff A. The chronic fatigue syndrome: a comprehensive approach to its definition and study. Ann Intern Med. 1994:121(12):953-9.

## P31 Rotating treadmill rehabilitation for ban+lance and gait in Parkinson’s disease

### Marica Giardini^1^, Marco Godi^1^, Anna Maria Turcato^1^, Ilaria Arcolin^1^, Fabrizio Pisano^1^, Marco Schieppati^2^, Antonio Nardone^3,4^

#### ^1^Istituti Clinici Scientifici Maugeri Spa SB (IRCCS), Scientific Institute of Veruno, Italy; ^2^ LUNEX International University of Health, Exercise and Sports, Differdange, Lussemburgo; ^3^ Istituti Clinici Scientifici Maugeri Spa SB (IRCCS), Scientific Institute of Pavia, Italy; ^4^ University of Pavia, Pavia, Italy

##### **Correspondence:** Marica Giardini (marica.giardini@gmail.com)

**Background and Objective:** Postural unsteadiness is a major problem of Parkinson’s disease patients (PD). This is frequently associated to falls, since poor dynamic balance aggravates gait problems, in particular in directional changes and curved trajectories [1]. It is well known that stepping in place on a continuously rotating treadmill with open eyes causes to the subject a podokinetic stimulation (PKS). At the end of PKS, if we turn off treadmill and we ask to the blended subject to stepping in place, subject spontaneously rotate towards the opposite direction of platform rotation. This effect is so-called podokinetic after rotation (PKAR) [2]. It was tested that adaptation to the rotating platform might improve balance and curved walking in PD [3]. Here, we compared traditional balance exercises (BE) directed by a physiotherapist to stepping-in-place on a rotating treadmill (RT) as means of improving steadiness in PD.

**Materials and Methods:** Treatments were administered to two PD groups of 15 subjects each, matched for age and severity (H&Y 2.4). Both groups completed 10 treatment sessions (3 weeks), each lasting one hour. In all patients we noted motor section of Unified Parkinson’s Disease Rating Scale, dynamic balance by using Mini-BESTest and gait spatio-temporal variables (while walking along linear and curved trajectories), before and after the training protocol.

**Results:** There were no significant differences between both groups at baseline evaluation in all variables. At the final evaluation, the score of Mini-BESTest increased in both groups (p < 0.005), signifying enhanced dynamic balance control. Linear walking variables did not change in RT group, whilst gait speed improved (p < 0.05) in BE group as consequence of increase in cadence (p < 0.05). In curved walking, RT group increased gait speed due to longer stride length (p < 0.05), whilst BE group increased gait speed due to increased cadence (p < 0.05).

**Conclusion:** These preliminary data suggest that PD patients can improve their dynamic balance control when trained on a RT, likely because it automatically implicates a fine medio-lateral control of the trunk. Not surprisingly, RT also improves gait along curved trajectories. Conversely, BE training is moderately helpful for specific balance performance, without improving walking under challenging conditions.

**References**

1. Guglielmetti S, Nardone A, De Nunzio AM, Godi M, Schieppati M. Walking along circular trajectories in Parkinson's disease. Mov Disord. 2009 Mar 15;24(4):598-604.

2. Earhart GM, Hong M. Kinematics of podokinetic after-rotation: similarities to voluntary turning and potential clinical implications. Brain Res Bull. 2006 Jun 15;70(1):15-21.

3. Godi M, Giardini M, Nardone A, Turcato AM, Caligari M, Pisano F, Schieppati M. Curved Walking Rehabilitation with a Rotating Treadmill in Patients with Parkinson's Disease: A Proof of Concept. Front Neurol. 2017 Feb 28;8:53.

## P32 New frontiers of research in physiotherapy: the importance of education for development of innovative strategies in physiotherapy

### Antonella Giffone^1^, Roberto Gusinu^1^, Giada Morini^2^, Maria Visceglia^2^, Patrozoa Galantini^1^

#### ^1^Corso di Laurea Magistrale in Scienze Riabilitative delle Professioni Sanitarie, University of Florence, Firenze, Italy; ^2^UO Formazione, Azienda Ospedaliero Universitaria Careggi, Florence, Italy

##### **Correspondence:** Antonella Giffone (antonella.giffone@stud.unifi.it)

**Background and Objctive:** the Continuing Medical Education (ECM), set of learning activities, theoretical and practical, has an important role in the constant maintenance of an updated training for professional health care and is a guarantee of a health service of high quality [1]. In spite of that, ECM is not always perceived as priorities for the professionals. To assess the interest and commitment of health professionals to enterprise training post-graduate in physiotherapy, it is taken into account the training activity recorded in a company University Hospital of the Tuscany Region.

**Materials and Methods**: through the management software available at the Department of Education of University Hospital Careggi in Florence (AOUC), for the last three years the Annual Training Plans (PAF) of AOUC have been taken into account from 2014 to 2016 for research of relevance physiotherapy courses; excluding those not purely clinical (eg. enterprise value) it is sought is the prevalence of the types of training activity between teaching in the classroom (TC), distance learning (DL), training in the field (TF), workshops (W), simulation (S).RESULTS: in the period studied they are programmed a total of 1164 training events, of which only 9% (99) was of interest physiotherapy, to different teaching type. They were divided as follows: 28% in 2014, 28% in 2015 and 43% in 2016. For each year, a large percentage (64% - 75%) was not carried out, and the type of the remaining courses has been almost a total TC. In particular, in 2014 and in 2015 the entire totality of the courses were carried TC; in 2016 4% has been dedicated to DL, and the rest to TC. Despite some of TF and W courses were setting, none of these has been completed.

**Conclusion:** This picture shows the general trend to a purely frontal unidirectional character education, even if it physiotherapy, which needs to develop technical-pratical capacity, could not be separated from learning experiential and field training. Thus the data collected show the need for development of FSC courses, reducing DA, also allow for more meaningful process of diffusion of skills and knowledge, useful to integrate the training of young graduates [2-3]

**References**

1. Beard J, Marriott J, Purdie H, Crossley J. Assessing the surgical skills of trainees in the operating theatre: a prospective observational study of the methodology. Clinical Governance: An International Journal. 2011;16.3.

2. Bortone, G. Formazione e cambiamento-Teoria e prassi. Aracne, 2008.

3. Boyatzis RE. The competent manager: a model for effective performance. 1982. John Wiley&Sons, New York, 1982.

## P33 Construct validity of the brief-BESTest in individuals with balance disorders

### Marco Godi^1^, Marica Giardini^1^, Ilaria Arcolin^1^, Simone Guglielmetti^1^, Stefano Corna^1^, Antonio Nardone^2,3^

#### ^1^Istituti Clinici Scientifici Maugeri Spa SB (IRCCS), Scientific Institute of Veruno, Italy; ^2^ Istituti Clinici Scientifici Maugeri Spa SB (IRCCS), Scientific Institute of Pavia, Italy; ^3^ University of Pavia, Pavia, Italy

##### **Correspondence:** Marco Godi (marco.godi@icsmaugeri.it)

**Background and Objective:** The Brief-Balance Evaluation System Test (Brief-BESTest) has been recently proposed as a useful clinical examination for measuring balance disorders [1], but some authors raised doubts about internal structure [2,3]. The objective of this study is to address the existing knowledge gap by examining the construct validity of Brief-BESTest.

**Materials and Methods:** For this reason, we evaluated: a) structural validity, comparing the different models presented in the literature of Brief-BESTest; b) concurrent validity, assessing relationship between Brief-BESTest and Activities-specific Balance Confidence Scale – 5 levels (ABC-5L); c) discriminant validity, estimating the ability of Brief-BESTest to identify fallers. We used a confirmatory factor analysis to investigate construct validity of the Brief-BESTest on a sample of 246 patients with balance disorders. To assess structural validity, we constructed three models of Brief-BESTest. Model 1 shows a RMSEA of 0.12 (C.I. 95% = 0.099–0.136), that suggested a low fit with data; not all fit indices of Model 2 reached an acceptable value (only SRMR was below its preselected cut-off of 0.05 for a well-fitted model); for Model 3 analysis revealed that the model fit (χ2 = 25.8, CFI = 0.97, TLI = 0.95, RMSEA = 0.03) was significantly better than the Model 1 and 2. Concurrent validity was assessed by calculating the correlation between Brief-BESTest and ABC scale total scores. No differences were found between values of Spearman correlation between Model 1 and ABC-5L, and between Model 3 and ABC-5L (rho=0.61 and 0.62 respectively, p=0.82). ROC curves were plotted to estimate discriminant validity, but no tests reached good level of accuracy. The AUC was 0.71 (C.I. 95% = 0.63–0.78) for Model 1 and 0.71 (C.I. 95% = 0.63–0.79) for Model 3.

**Results:** Our results confirmed the good level of construct validity of Brief-BESTest, in neurological patients with balance disorders, after applying some changes such as: removal of item 1 and the change of modality for calculation of total score, as proposed by Model 3. The scale was found to be unidimensional, and to have a good convergent validity with measure of balance confidence. Moreover, the Brief-BESTest confirmed to be able to identify fallers from non-fallers better than ABC.

**References**

1. Padgett PK, Jacobs JV, Kasser SL. Is the BESTest at its best? A suggested brief version based on interrater reliability, validity, internal consistency, and theoretical construct. Phys Ther. 2012 Sep;92(9):1197-207.

2. Franchignoni F, Giordano A. On "Is the BESTest at its best?...." Padgett PK, Jacobs JV, Kasser SL. Phys Ther. 2012;92:1197-1207. Phys Ther. 2012 Sep;92(9):1236-7.

3. Bravini E, Nardone A, Godi M, Guglielmetti S, Franchignoni F, Giordano A. Does the Brief-BESTest Meet Classical Test Theory and Rasch Analysis Requirements for Balance Assessment in People With Neurological Disorders? Phys Ther. 2016 Oct;96(10):1610-1619.

## P34 A “token economy intervention to improve adherence to aerosol therapy in children with cystic fibrosis: outcome research

### Luigi Graziano^1^, Gianluca Paris^1^, Chiara Fantacci^2^, Tamara Perelli^1^, Beniamino Giacomodonato^1^, Matteo De Marchis^1^, Enea Bonci^1^

#### ^1^Policlinico Umberto I, Rome (Italy); ^2^Freelance psychologist

##### **Correspondence:** Luigi Graziano (luigi.graziano@uniroma1.it)

**Background and Objective:** Self management skills are needed for children with cystic fibrosis (CF) to reach an acceptable adherence to recommended aerosol medicines. Many studies showed how this adherence is generally poor, but nobody until today concentrated on how improve it [1].

Our aim is to evaluate the effects of an occupational therapy intervention on cardiovascular function (Six Minutes Walking Test), lung function (FEV1, FVC, FEF25/75) as consequence of a better adherence monitored using Test of Adherence to Inhalers questionnaire (TAI) [2] lasting one month, in a group of pediatric CF patients.

**Materials and Methods:** 15 patients with CF were enrolled. Inclusion criteria: CF diagnosis; prescription of aerosol therapy; age between 6 and 14. Exclusion criteria: diagnosis of atipical kind of CF; transplanted; awaiting transplant; difficulties in under standing Italian language. Assessments were conducted immediately before and after the intervention period. The token economy technique was managed by the child and caregiver together at home.

**Results:** Mean TAI scores improve statistically significative (3,286; p<0,001), so did lung function (mean FEV1 improved by 0,09) and cardiovascular function (mean SMWT (70,27 m; p<0,001).

**Conclusions:** An occupational therapy intervention, based on token economy [3], should be the way to improve adherence to aerosol therapy for CF pediatric patients.

**References**

1. Bishay LC, Sawicki GS. Strategies to optimize treatment adherence in adolescent patients with cystic fibrosis. Adolesc Health Med Ther. 2016 Oct 21;7:117-24

2. Plaza V, Fernández-Rodríguez C, Melero C, Cosío BG, Entrenas LM, de Llano LP, Gutiérrez-Pereyra F, Tarragona E, Palomino R, López-Viña A; TAI Study Group. Validation of the 'Test of the Adherence to Inhalers' (TAI) for Asthma and COPD Patients. J Aerosol Med Pulm Drug Deliv. 2016 Apr;29(2):142-52. doi: 10.1089/jamp.2015.1212. Epub 2015 Jul 31. PubMed PMID: 26230150; PubMed Central PMCID: PMC4841905.

3. Bernard RS, Cohen LL, Moffett K. A token economy for exercise adherence in pediatric cystic fibrosis: a single-subject analysis. J Pediatr Psychol. 2009 May;34(4):354-65.

## P35 Cystic fibrosis and game: a lucky meeting? A randomized controlled trial

### Luigi Graziano, MatteoDe Marchis, Francesca Alatri, Tamara Perelli, Beniamino Giacomodonato, Gianluca Paris, Enea Bonci

#### Policlinico Umberto I, Rome (Italy)

##### **Correspondence:** Luigi Graziano (luigi.graziano@uniroma1.it)

**Background and Objective:** Patients with cystic fibrosis (CF) generally practice physical activity, but adherence is often poor, especially for paediatric population, and the hospitalization for a pulmonary exacerbation can determine general inactivity[1-2]. In fact, patients receive only chest physiotherapy and antibiotic therapy. The aim of the trial was to evaluate the physical intervention’s benefits, based on ludic activities, about exercise’s capacity (Six Minutes Walking Test), lung function (FEV1) and Quality of Life (CFQ-R), obtained during hospitalization for pulmonary exacerbation lasting two weeks, in a group of paediatric CF patients [3]. We decided, also, to analyze patient’s preference between ludic activities and usually prescribed physical activity thanks to Likert Scale [4].

**Materials and Methods:** 30 Pediatric CF subjects had been admitted to a hospital for a 15-d programmed intravenous antibiotic cycle, were recruited after obtaining informed consent. They were simply randomized, assigned to a physical activity based on ludic activities (study group) or to a physical exercise programme (control group). Inclusion criteria were: CF diagnosis confirmed using sweat test, hospitalization for pulmonary exacerbation, age between 6 and 18 y inclusive. Exclusion criteria included fever at admission. All measurements were performed at admission and at discharge. The experimental intervention was characterized by “animal games” (e.g. jumping like a rabbit, then like a kangaroo…), circuit routes with different materials, and also traditional games (cherry picking, cops and robbers…).

**Results:** We found statistically significant increases in patients’ preference for intervention based on ludic activities versus usual physical activity (Likert + 23,20/100; p=0,001). We didn’t find for the other outcomes a statistical significance.

**Conclusions:** We believe that children are an appropriate audience for the physical intervention based on ludic activities especially to improve adherence to general physical activity.

**References**

1. Stevens D, Oades PJ, Armstrong N, Williams CA. A survey of exercise testing and training in UK cystic fibrosis clinics. J Cyst Fibros. 2010 Sep;9(5):302-6.

2. Wheatley CM, Wilkins BW, Snyder EM.. Exercise is medicine in cystic fibrosis. Exercise and sport sciences reviews, 2011, 39.3: 155-160.

3. Riekert KA, Eakin MN, Bilderback A, Ridge AK, Marshall BC. Opportunities for cystic fibrosis care teams to support treatment adherence. J Cyst Fibros. 2015 Jan;14(1):142-8.

4. Knapp TR. Assessing Validity and Reliability of Likert and Visual Analogue Scales. Available at http://www.statlit.org/pdf/2013-knapp-likert-and-visual-analog-scales.pdf

## P36 Programme of perioperative pulmonary rehabilitation in surgically treated lung cancer patients: preliminary data

### Carlotta Mainini^1^, Roberta Bardelli^1^, Besa Kopliku^1^, Patrícia Filipa Sobral Rebelo^1^, Laura Cantarelli^1^, Sara Tenconi^2^, Cristian Rapicetta^2^, Roberto Piro^3^, Stefania Costi^1,4^, Carla Galeone^2^, Patrizia Ruggiero^3^, Claudio Tedeschi^1^, Stefania Fugazzaro^1^

#### ^1^Unit of Physical and Rehabilitation Medicine, Istituto di Ricerca e Cura a Carattere Scientifico - Arcispedale Santa Maria Nuova, Reggio Emilia, Italy; ^2^Unit of Thoracic Surgery, Istituto di Ricerca e Cura a Carattere Scientifico - Arcispedale Santa Maria Nuova, Reggio Emilia, Italy; ^3^Unit of Pneumology, Istituto di Ricerca e Cura a Carattere Scientifico - Arcispedale Santa Maria Nuova, Reggio Emilia, Italy; ^4^Department of Biomedical, Metabolic and Neural Sciences, University of Modena and Reggio Emilia, Modena Italy

##### **Correspondence:** Carlotta Mainini (carlotta.mainini@ausl.re.it)

**Background and Objective:** Non-small Cell Lung Cancer (NSCLC) comprises 85% of all lung cancers. Lung resection is the election treatment but surgery might have a significant impact on Quality of Life (QoL) and physical condition. Pulmonary rehabilitation (PR), both before and after surgery, including aerobic and strength exercises, could reduce symptoms and morbidity and improve exercise capacity, pulmonary function and QoL.

Aim: investigate the efficacy of intensive PR on exercise capacity for NSCLC patients surgically treated.

**Materials and Methods:** Open-label randomized controlled trial. Participants: suspected or diagnosed NSCLC (staging I-II), waiting for surgery, not candidates for neo-adjuvant or adjuvant therapy.

Control group (CG): one therapeutic educational session the day before surgery and early standard inpatient PR after surgery.

Intervention group (IG): early standard inpatient PR after surgery plus 14 preoperative PR sessions (6 outpatient and 8 home-based) and 39 postoperative PR sessions (15 outpatient e 24 home-based). This experimental treatment is based on aerobic, resistance and respiratory training both pre and post-operative. Detailed experimental programme is reported in figure 1.

Patients are assessed at enrollment (T0), the day before surgery (T1), one month after surgery (T2) and six month after surgery (T3) for exercise capacity, respiratory functions, pain, mood disturbances and quality of life (Table 1).

Primary outcome: Six Minutes Walk Test (6MWT)

**Results:** We present data regarding the first 86 patients enrolled (42 IG; 44 CG). Preliminary analysis of the primary outcome (6MWT) in IG shows an average improvement of 56m 6 months after surgery and the difference from T0 to T3 is statistical significant (p=0,002). This difference in CG is not significant (p=0,809).

The compliance is high: 71% in the preoperative phase and 86% in the postoperative phase.

No adverse effects were registered.

**Conclusion:** Preliminary data seems to highlight the efficacy of perioperative PR improving exercise capacity. The experimental intensive PR programme implemented registered high level of adherence and no side effects treatment related.

**Table 1 (abstract P36).**
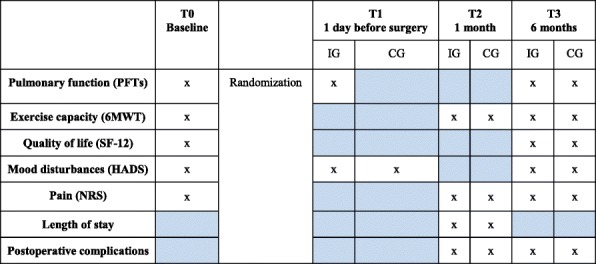
Compliance at experimental programme, both pre -and postoperative

**Fig. 1 (abstract P36).**
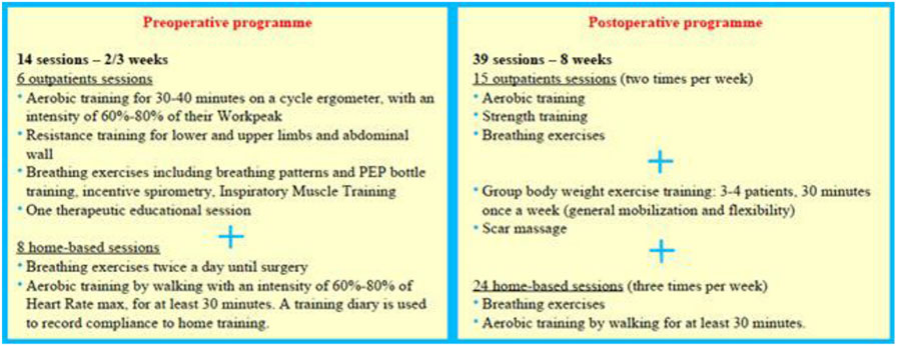
Preoperative and postoperative programme

## P37 Respiratory management of people with sci (spinal cord injury) from hospital to discharge: flow-chart proposal fron an Italian spinal unit

### Giulia Marescotti, Francesca Plazzi, Marianna Tessitore, Alessandra Areni, Jacopo Bonavita

#### Unità Spinale di Montecatone (BO)

##### **Correspondence:** Giulia Marescotti (giulia.marescotti@montecatone.com)

**Background and Objective:** Respiratory function is compromised by Spinal Cord Injury (SCI) and the more severe the deficiency is the higher the AIS level is [1]. Respiratory deficiency is caused by muscle paralysis and by reduced capacity in clearing respiratory secretion. Even now infection pneumonia is one the main cause of mortality in SCI [1]. In people with SCI, rehabilitative treatment has to set two main goals: keep the lungs and throat clear of mucus and improves respiratory muscle strength and endurance. Cough assistance (CA) is one of the most important treatment in respiratory rehabilitation [2-4].

There are other respiratory management techniques which are associated to CA.

Aim: in order to even out the management of rehabilitation process in our Spinal Unit, we made a flow-chart about respiratory treatment in people with SCI.

**Materials and Methods:** This flow chart describes our experience about respiratory management from acute phase to discharge, in respect with the scientific evidence. The aim is for people affected by SCI , in spontaneous breathing, to reach as much autonomy as possible. This is a procedure that should be followed “step by step”.

**Results:** The use of this flow-chart in the SU Montecatone has the aim not only to define a uniform rehabilitation process, but also to be a model for the training process of the respiratory therapist. It will be necessary more studies to validate this kind of process. We believe that for the application of rehabilitative program is fundamental a perfect integration between the team’s professional and the correct use of medical exams.

**References**

1. Zimmer MB, Nantwi K, Goshgarian HG. Effect of spinal cord injury on the respiratory system: basic research and current clinical treatment options. J pinal Cord Med. 2007;30(4):319-30.

2. Amirjani N, Kiernan MC, McKenzie DK, Butler JE, Gandevia SC. Is there a case for diaphragm pacing for amyotrophic lateral sclerosis patients? Amyotroph Lateral Scler. 2012 Oct;13(6):521-7.

3. Bach JR, Saporito LR, Shah HR, Sinquee D. Decanulation of patients with severe respiratory muscle insufficiency: efficacy of mechanical insufflation-exsufflation. J Rehabil Med. 2014 Nov;46(10):1037-41.

4. Gómez-Merino E, Sancho J, Marín J, Servera E, Blasco ML, Belda FJ, Castro C, Bach JR. Mechanical insufflation-exsufflation: pressure, volume, and flow relationships and the adequacy of the manufacturer's guidelines. Am J Phys Med Rehabil. 2002 Aug;81(8):579-83.

## P38 Effects of a functional exercise program on manual wheelchair propulsion ability and life satisfaction in paraplegic subjects: two case reports

### Luca Marin^1,2^, Claudio Lisi^3^, Giuseppe Di Natali^3^, Fabrizio Abbiati^4^, Matteo Vandoni^1^, Sara Ottobrini^1,5^

#### ^1^University of Pavia, Pavia, Italy; ^2^University of Roma Tor Vergata, Rome, Italy; ^3^IRCCS Policlinico S. Matteo Foundation, Pavia, Italy; ^4^A.S.P. Rehabilitation and Care Institute Santa Margherita, Pavia, Italy; ^5^University of Genoa, Genoa, Italy

##### **Correspondence:** Luca Marin (luca.marin@unipv.it)

**Background and Objectives:** The functional exercise improves many abilities in disabled people [1-2]. This study aimed to evaluate the effects of a functional exercise (FE) program on manual wheelchair propulsion ability (MWPA) and life satisfaction in two paraplegic subjects.

**Materials and Methods:** Two women with long term complete and incomplete paraplegia (subject A: 7 years post injury, AIS A, complete T7 lesion, aged 21; subject B: 7 years post injury, AIS C, incomplete T8 lesion, aged 20) completed a twice a week 75minute FE program, based on aerobic and anaerobic exercises, for 7 months.

Subjects were evaluated before (T0) and after (T1) the training period.

Level and covered distance in a multistage field test (MFT) and a Vanlandewijck’s 30second sprint test were the outcomes used to assess MWPA.

Satisfaction profile questionnaire (SAT-P) was used to evaluate subjects’ satisfaction about different life areas. SAT-P is composed by 5 factors: work (W), sleep-nutrition-free time (S-N-FT) and psychological (PSY), physical (PHY) and social (SOC) functions.

**Results:** Subject A improved from level 5 (480 meters) to level 8 (900 meters) and subject B from level 6 (575 meters) to level 7 (840 meters) in MFT.

In 30second sprint test, women increased from 64 to 71 (A) and from 66 to 69 meters (B).

Subject A satisfaction increased in every factor; while subject B satisfaction improved in physical function, work and sleep-nutrition-free time factors.

**Conclusion:** The results encourage the hypothesis that a functional exercise program is able to improve both MWPA and life satisfaction in paraplegic subjects with different AIS score. It would be interesting to verify the correlation between MWPA and satisfaction in different life areas [3]. Further results from a larger sample are necessary to clarify this topic.

**References**

1. Bochkezanian V, Raymond J, de Oliveira CQ, Davis GM. Can combined aerobic and muscle strength training improve aerobic fitness, muscle strength, function and quality of life in people with spinal cord injury? A systematic review. Spinal Cord. 2015 Jun;53(6):418-31.

2. Nash MS. Exercise as a health-promoting activity following spinal cord injury. J Neurol Phys Ther. 2005 Jun;29(2):87-103, 106.

3. Stevens SL, Caputo JL, Fuller DK, Morgan DW. Physical activity and quality of life in adults with spinal cord injury. J Spinal Cord Med. 2008;31(4):373-8.

**Fig. 1 (abstract P38).**
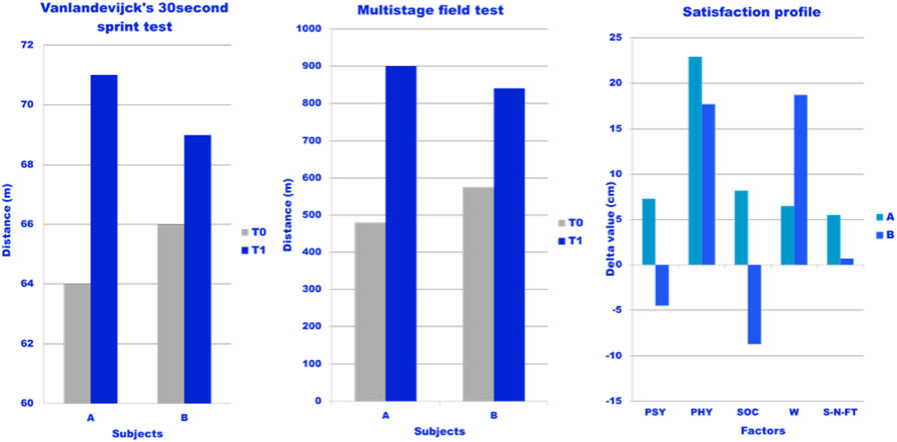
Changes in Vanlandevjck’s test, Multistage test and Satisfaction profile

## P39 Short-term effects of two feedback systems on the self-correction movement in patients with idiopathic scoliosis. A study design

### Luca Marin^1,2,3^, Luisella Pedrotti^1,3^, Massimiliano Febbi^2^, Manuela Anelli^4^, Sara Ottobrini^1,5^

#### ^1^University of Pavia, Pavia, Italy; ^2^University of Roma Tor Vergata, Rome, Italy; ^3^Città di Pavia Hospital, Pavia, Italy; ^4^IULM University, Milan, Italy; ^5^University of Genoa, Genoa, Italy

##### **Correspondence:** Luca Marin (luca.marin@unipv.it)

**Background and Objectives:** Physiotherapy exercises of schools that demonstrated their efficacy through scientific studies are recommended in the idiopathic scoliosis treatment [1]: they have to be performed in front of a mirror, which acts as a useful visual feedback system [2] for learning and performing the self-correction movement.

Feedback systems are also used in orthopaedic rehabilitation as a support during the exercise performance [3]. The one based on surface electromyography (sEMG) is effective in muscle rehabilitation [4]. Several studies used sEMG to examine the paraspinal muscles activity in adolescents with idiopathic scoliosis. Nevertheless, participants were not reported back on any information [5]. Namely, short-term effects of biofeedback sEMG system use on the self-correction movement performance during posture exercise sessions have never been investigated.

Aim: To evaluate and to compare the effects of two exercise sessions, the former with the aid of a mirror, the latter using a sEMG biofeedback on the self-correction movement performed by individuals with idiopathic scoliosis.

**Materials and Methods:** Fifty subjects aged 8-14 years with juvenile and adolescent idiopathic scoliosis diagnosis, with ≤ 20^o^ Cobb angle either single or double curve will be recruited from the paediatric orthopaedic outpatient department of the hospital “Città di Pavia”. Participants will have to be new to posture exercise. Exclusion criteria will be: brace therapy or its indication, concomitant orthopaedic diseases. Participants’ parents will consent in a written form.

Participants will take part in three sessions: training, mirror aid, sEMG biofeedback. Sessions will take place within a week of each other, the last two being in a random order. During the first session participants will learn the self-correction movement. In the next ones, by being supported by feedback (see figures 1 and 2), they will perform four self-correction-based exercises suggested by scientific literature. Through randomization each participant will be assigned a Physiotherapist assisting him during all sessions and an Evaluator. Both will be blinded.

At the beginning and at the end of the feedback-supported sessions scoliotic curves, both in the resting position and during self-correction, will be measured through rasterstereography. Three measurement will be recorded in each position. The best one will be used to assess differences between beginning and end of each session.

**References**

1. Romano M, Minozzi S, Bettany-Saltikov J, Zaina F, Chockalingam N, Kotwicki T, Maier-Hennes A, Negrini S. Exercises for adolescent idiopathic scoliosis. Cochrane Database Syst Rev. 2012;15;(8):CD007837.

2. Brun C, Guerraz M. Anchoring the "floating arm": Use of proprioceptive and mirror visual feedback from one arm to control involuntary displacement of the other arm. Neuroscience. 2015;310:268-78.

3. Teran-Yengle P, Cole KJ, Yack HJ. Short and long-term effects of gait retraining using real-time biofeedback to reduce knee hyperextension pattern in young women. Gait Posture. 2016;50:185-189.

4. Lyons GM, Sharma P, Baker M, O’Malley S, Shanahan A. A computer game-based EMG biofeedback system for muscle rehabilitation. In: Engineering in Medicine and Biology Society, 2003. Proceedings of the 25th Annual International Conference of the IEEE. IEEE, 2003;1625-1628.

5. Chwała W, Koziana A, Kasperczyk T, Walaszek R, Płaszewski M. Electromyographic assessment of functional symmetry of paraspinal muscles during static exercises in adolescents with idiopathic scoliosis. Biomed Res Int. 2014;2014:573276.

**Fig. 1 (abstract P39).**
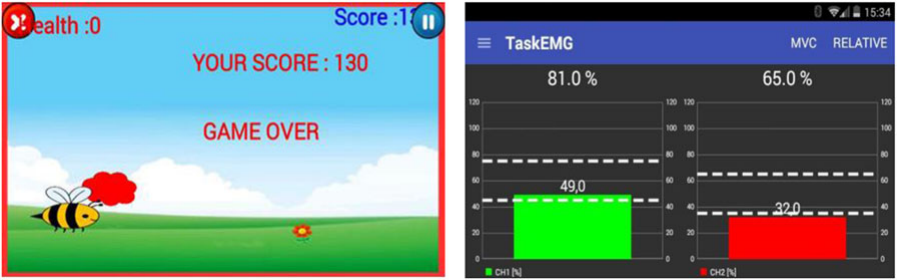
Examples of feedback by self-correction-based exercises

## P40 The effectiveness of the rehabilitative treatment in the child with cerebellar ataxia

### Elena Masala^1^, Silvia Paoli^2^, Rosanna Deriu^3^

#### ^1^Physiotherapist, specialist in pediatric area, Sassari, Italy; ^2^Physiotherapist, specialist in pediatric area, Azienda Ospedaliero Universitaria Meyer, Firenze, Italy; ^3^Physiotherapist, Sassari, Italy

##### **Correspondence:** Elena Masala (elenamasala88@gmail.com)

**Background and Objective:** There are numerous different physiotherapeutic tools used for the treatment of a child affected by ataxia. Among the most studied methods you can find: treadmill training with or without Body Weight Support, Biofeedback, balance and manual coordination training within the methods of Physical Therapy and Occupational Therapy, virtual reality with "Exergames" video games, Axial Weighting and orthotics Lycra garments. According to medical literature they are grouped into two main categories: compensatory tools and rejuvenating tools. In clinical practice, these tools are combined in a variety of ways, making it impossible to elaborate specific guidelines for the treatment of ataxic pathologies.

The objective of the study is to analyze which tools are available for the treatment of ataxic syndromes in the developing age and to examine which efficacy tests are available in the published literature for each of them, in order to support clinical practice based on evidence as far as possible.

**Materials and Methods:** Researches on literature have been carried out by consulting the following electronic databases: PubMED, PEDro, Google Scholar, Web of Science and The Cochrane Library as well as websites of American and Australian physiotherapy associations and sites dedicated to this pathology. The study, first conducted on pediatric age groups, was thereafter extended to adults.

**Results:** Out of the 33 articles reviewed, there were 3 systematic reviews, 5 randomized controlled trials, 9 quasi-experimental studies, 3 case-control studies, 5 case series, 3 case reports and 5 single-case studies. Among these, only 6 studies concerned the pediatric field. In the case of progressive ataxia, in all its forms, there is the absolute necessity of intensive interventions and home-based activities. Analyzed pediatric tools included, among others, treadmill training and body weight support, Exergames, and the use of orthotics Lycra garments.

**Conclusions:** The most effective treatments are those that envisage coordination and balance exercises, if combined with functional activities with problem-solving cognitive approach. It clearly emerged that there is need for guidelines for the treatment of ataxia, especially for ataxic disorders in the developing age.

**References**

1. Cassidy E, Kilbride C, Holland A, Ataxia UK. Management of the Ataxias: towards best Clinical Practice. 2009. Available at: http://citeseerx.ist.psu.edu/viewdoc/download?doi=10.1.1.469.6391&rep=rep1&type=pdf

2. Ferrari A, Biagioni E, Paolicelli PB. Le atassie progressive e non progressive del bambino: principali quadri clinici. Le atassie non progressive del bambino: quadri clinici ed orientamenti riabilitativi. Tirrenia, Del Cerro, 1998

3. Fonteyn EM, Keus SH, Verstappen CC, Schöls L, de Groot IJ, van de Warrenburg BP. The effectiveness of allied health care in patients with ataxia: a systematic review. J Neurol. 2014;261:251-8.

4. Marquer A, Barbieri G, Pérennou D. The assessment and treatment of postural disorders in cerebellar ataxia: a systematic review. Ann Phys Rehabil Med. 2014;57:67-78.

5. Miyai I, Ito M, Hattori N, Mihara M, Hatakenaka M, Yagura H, Sobue G, Nishizawa M; Cerebellar Ataxia Rehabilitation Trialists Collaboration. Cerebellar ataxia rehabilitation trial in degenerative cerebellar diseases. Neurorehabil Neural Repair. 2012;26:515-22.

## P41 Action observation plus sonification. A novel therapeutic protocol for Parkinson’s patient with freezing of gait

### Susanna Mezzarobba^1,4^, Lorella Pellegrini^4^, Michele Grassi^1^, Mauro Catalan^2^, Bjorn Kruger^3^, Paolo Manganotti^2,4^, Paolo Bernardis^1^

#### ^1^Department of Life Sciences, University of Trieste, Italy; ^2^ Azienda Sanitaria Universitaria Integrata di Trieste, Italy; ^3^ Gokhale Method Institute, CA, USA; ^4^ Department of Medical, Surgical and Health Sciences, University of Trieste, Italy

##### **Correspondence:** Lorella Pellegrini (lpellegrini@units.it)

This abstracts have been omitted from this publication as it has previously been published elsewhere. A summary is included below.

In this randomized controlled the authors studied the effects of a multisensory approach that combined visual and auditory stimula in a small sample of patients with Parkoinson’s disease and freezing of gait. Participants in the experimental group performed gait-related actions while observing videos showing the same gestures, where the auditory component was obtained by sonification, i.e. by transforming the kinematic data into sounds. The control group performed the same actions without observing any videos. Significant improvements were observed in the experimental group at the end of treatment, which were maintained at a 3 months follow-up. This study has been published as a full text article after the AIFI Congress [1].

**Reference**

1. Mezzarobba S, Grassi M, Pellegrini L, Catalan M, Kruger B, Furlanis G, Manganotti P, Bernardis P. Action Observation Plus Sonification. A Novel Therapeutic Protocol for Parkinson's Patient with Freezing of Gait. Front Neurol. 2018 Jan 4;8:723. doi: 10.3389/fneur.2017.00723. eCollection 2017.

## P42 Efficacy of centrally applied Mulligan sustained natural apophyseal glide mobilization on patients with chronic mechanical neck dysfunction

### Sara Mohamed Samir^1^, Lilian Albert Zaki^2^, Mohamed Omar Soliman^3^, Enas Metwaly Abd Elmenam^4^

#### ^1^Faculty of Physical Therapy, Cairo University, Egypt; ^2^Faculty of Physical Therapy, Cairo University, Egypt; ^3^Faculty of Medicine, Cairo university, Egypt; ^4^Faculty of Physical Therapy, Cairo University, Egypt

##### **Correspondence:** Sara Mohamed Samir (enhortho80@yahoo.com)

**Background and Objective:** Mechanical neck dysfunction (MND) is a common disorder prevailing among individuals of different population [1]. This study conducted to investigate the efficacy of cervical central sustained natural apophyseal glides (SNAGs) [2-4] on neck pain severity level and functional disability in patients with chronic mechanical neck dysfunction.

**Materials and Methods:** Thirty male and female patients who met the inclusion criteria were randomly assigned into two groups. Group A (n=15) received central SNAGs in addition to conventional exercise therapy program for the neck in form of (isometric exercises, stretching exercises, and postural exercises), Group B (n =15) were treated by same exercise therapy program only, treatment received three sessions per week for successive 4 weeks. Visual analogue scale (VAS) and neck disability index (NDI) were measured at two intervals pre-treatment and post-treatment.

**Results:** MANOVA and post hoc tests revealed that there was statistical significant reduction in pain severity level and functional disability within both groups (p< 0.001) and there was no statistical significant results between groups (P=0.134). But there was clinical difference and high percent of improvement “clinically” favor to group A concerning pain level and functional disability, the percentage change in scores of VAS and NDI were higher in group A (50.59%, 4.47% respectively, P=0.001) than in group B (41.86% and 3.09% respectively, P=0.001).

**Conclusion:** Both conventional exercise therapy and SNAGs mobilization are effective modalities in alleviating pain and improving neck dysfunction in patients with chronic mechanical neck dysfunction. Centrally Mulligan SNAGs mobilization has an acceptable clinical applicability.

**References**

1. Gross AR, Hoving JL, Haines TA, Goldsmith CH, Kay T, Aker P, Bronfort G; Cervical Overview Group. A Cochrane review of manipulation and mobilization for mechanical neck disorders. Spine (Phila Pa 1976). 2004;29:1541-8.

2. Exelby L. The Mulligan concept: its application in the management of spinal conditions. Man Ther. 2002;7:64-70.

3. Hall T, Chan HT, Christensen L, Odenthal B, Wells C, Robinson K. Efficacy of a C1-C2 self-sustained natural apophyseal glide (SNAG) in the management of cervicogenic headache. J Orthop Sports Phys Ther. 2007;37:100-7.

4. Mulligan BR. Manual Therapy: “Nags” “Snags” “MWMs”. 5th ed. Wellington, New Zealand: Plane View Service; 2005.

**Fig. 1 (abstract P42).**
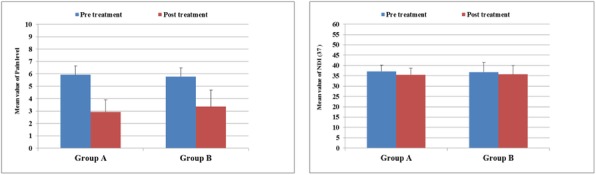
See text for description

**Table 1 (abstract P42).** See text for description

	NDI					Pain level			
	Pre test Mean± SD	Post test Mean± SD	MD	% of change	p- value	Pre test Mean± SD	Post test Mean± SD	MD	% of change
Group A	37.13±2.94	35.46±3.15	1.66	4.47	0.003*	5.93±0.70	2.93 ±0.96	3	50.59
Group B	36.85 ±4.68	35.71 ±4.28	1.14	3.09	0.043*	5.78 ±0.69	3.35±1.33	2.42	41.86
MD	0.27	-0.24				0.14	-0.42		
p- value	0.85	0.86				0.576	0.333		

## P43 Change in the muscle tension of the shoulder girdle muscles in patients with pain, using the tone control® technique

### Umberto Motta^1,2^, Ester da Pos^1^

#### ^1^ASP Pio Albergo Trivulzio, Milano, Italy; ^2^School of Physiotherapy, University of Milan, Milan, Italy

##### **Correspondence:** Umberto Motta (motta.umberto@gmail.com)

**Background and Objective:** The aim of the study, which is randomized blinded controlled, was to verify the efficacy of the Tone-Control® method in inducing a reduction in the tension of the muscles of the shoulder girdle, and therefore a normalisation of posture in the segment [1-3].

**Materials and Methods:** The authors analysed the change in posture, which was related to the muscle tension of the pectoralis major muscle and the trapezius muscle, resulting from the administration of a programme of encoded exercises.

The study was conducted on 70 patients with postural back pain, aged between 25 and 81 years of age and with a mean age of 61.9 years, 11 male patients and 59 female patients, divided into a study group of 40 patients and a control group of 30 patients. Rehabilitation sessions were held in groups of four or five persons, for a minimum of 10 and a maximum of 15 sessions lasting one hour each. Acute phase patients, patients on anti-inflammatory pharmacological treatment and patients with hernias or bulging causing thecal sac impingement were excluded from the study.

The study group has performed some sequences of active exercises of the Tone Control® method, whereas the control group performed active mobilization and proprioceptive stimulation of the shoulder girdle.

Both groups integrated this process using the same sequence of active exercises of mobilization for spine and lower limbs, to improve the segmental reinforcement of the abdominal muscles, quadriceps muscles and stabilisers of the pelvis, active stretching of the posterior chain of lower limbs and proprioceptive stimulation when loading with both static and dynamic balance exercises.

Measurements of the angles of the joints in the scapulohumeral segment, evaluated in degrees using the goniometer, were specifically detected on shoulder anteposition, elevation of the shoulder girdle and shoulder flexion during the first and last sessions. The NRS pain scale was administered at the start and end of the cycle of sessions.

**Results and Conclusion:** Patients in the study group experienced improvements in the angle measurements that were proportionally greater than those of the control group, together with a considerable reduction in perceived pain, with an overall improvement in posture and girdle function.

**References**

1. Korr IM. Proprioceptors and somatic dysfunction. J Am Osteopath Assoc. 1975 Mar;74(7):638-50.

2. Radovanovic D, Peikert K, Lindström M, Domellöf FP. Sympathetic innervation of human muscle spindles. J Anat. 2015 Jun;226(6):542-8.

3. Hník P. Controversial aspects of skeletal muscle tone. Biomed Biochim Acta. 1986;45(1-2):S139-43.

**Fig. 1 (abstract P43).**
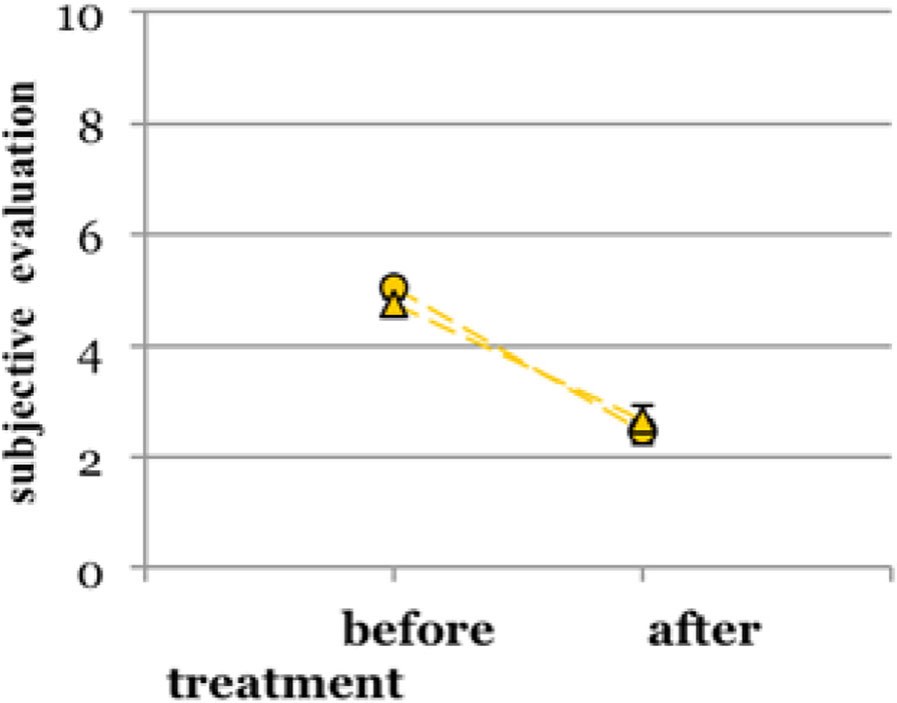
Subjective evaluation of pain before and after treatment, in the Tone Control group (circles) and in the control group (triangles). Conclusion: both treatments therefore achieve the purpose of reducing pain, with an almost identical improvement, although the gain of the Tone Control group is 1.25 times that of the control group

**Fig. 2 (abstract P43).**
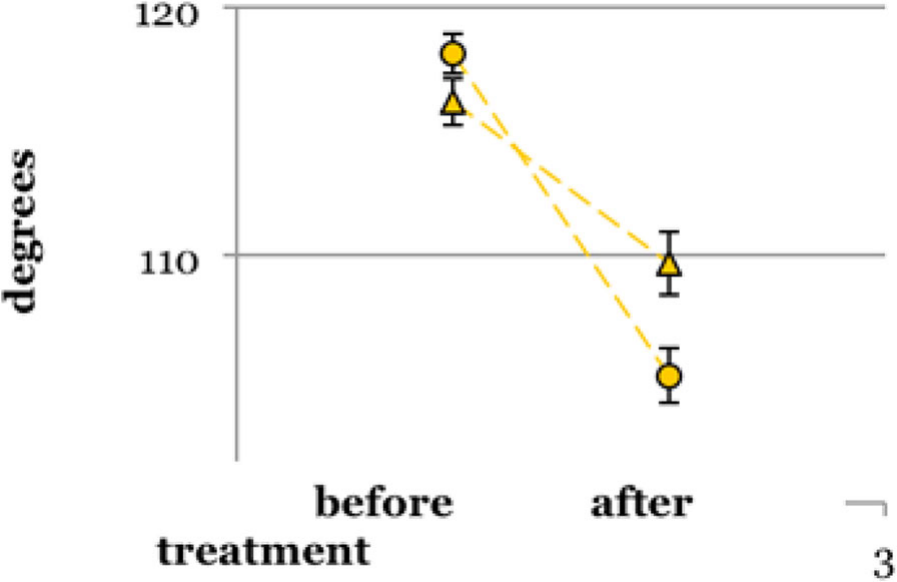
Curvature angle of clavicle before and after treatment, in the Tone Control groups (circles) and in the control (triangles) group. Conclusion: The gain of the Tone Control group is double that of the control group

**Fig. 3 (abstract P43).**
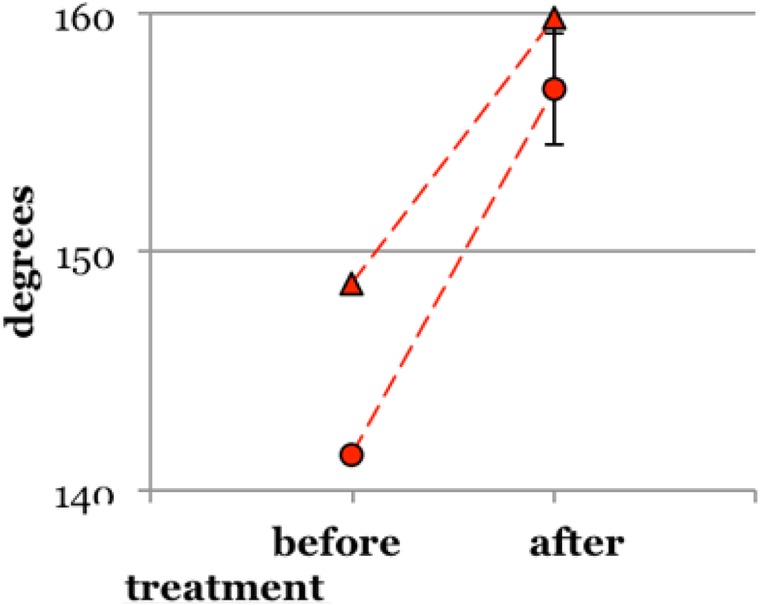
Shoulder flexion before and after treatment, in the Tone Control (circles) and in the control (triangles) group. Conclusion: The gain of the Tone-control group is 1.38 times that of the control group

**Fig. 4 (abstract P43).**
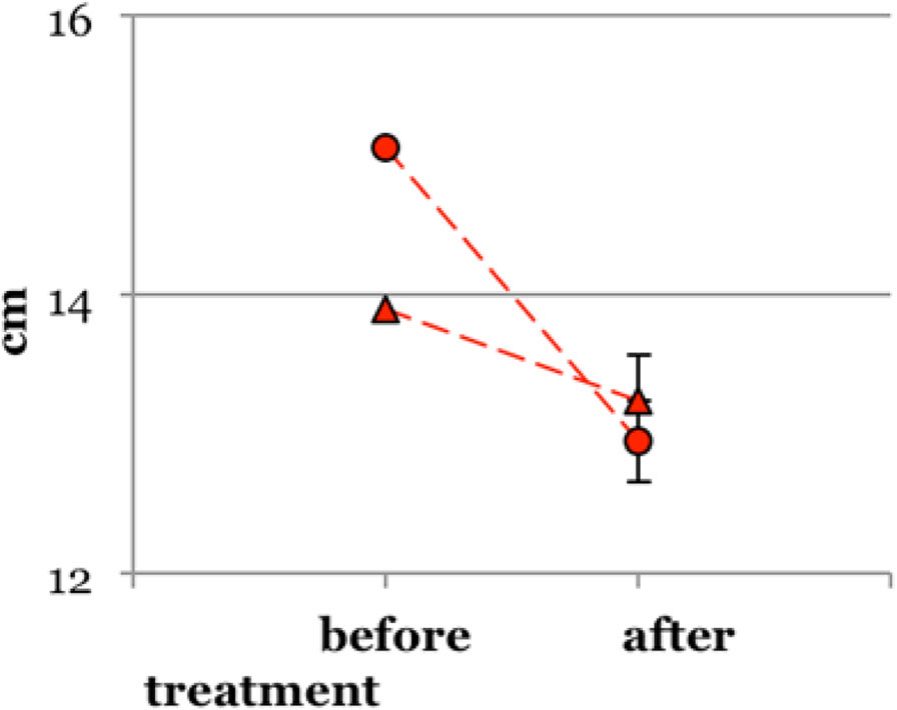
Shoulder anteposition before and after treatment, in the Tone Control (circles) and in the control (triangles) group. Conclusion: the gain of the Tone Control group is 3.15 that of the control group

## P44 Italian cross-cultural adaptation of the Short Sensory Profile

### Alessandra Nale, Rita Pirovano, Giulio Valagussa, Enzo Grossi

#### Autism Research Unit, Villa S. Maria Institute, Tavernerio (CO), Italy

##### **Correspondence:** Giulio Valagussa (giulio.valagussa@gmail.com)

**Background and Objective:** Autism is a neurodevelopmental disorder characterized by abnormalities of reciprocal social interactions and communication, restricted interests and repetitive behavior. Sensory processing problems are reported in children with ASD [1] and are included in the diagnosis in the latest Diagnostic and Statistical Manual of Mental Disorders (DSM V). One of the most useful tools to assess sensory characteristics in ASD subjects is the Short Sensory Profile (SSP) [2], but no Italian version of this instrument is currently available. The aim of this study is to validate an Italian cross-cultural adaptation of the Short Sensory Profile.

**Materials and Methods:** Following the guidelines for the process of cross-cultural adaptation of self-report measures [3] we did a forward translation, followed by a back translation and by a final review. We also did a pilot study to apply the SSP in a sample of 46 Italian ASD children (7 females; 39 males; mean age 163.5 months – SD 34.3 months). The ASD diagnosis was done using the DSM V criteria, and it was confirmed using the ADOS 2.

**Results:** The SSP mean total score was 147.65, pointing out the presence of sensory function impairment. In the sample, 32% (N=15) of the participants obtained a typical performance (TP) total score (range 155-190), 30.4% (N=14) obtained a probable difference (PD) score (range 142-154), and 37% (N=17) obtained a definite difference (DD) score (range 38-141). The sensory function impairment resulted particularly severe in two of the Scale sections (table 1): “Underresponsive/Seeks Sensation” (8.7% TP score, 26.1% PD score, 65.2% DD score) and “Auditory Filtering” (17.4% TP score, 39.1% PD score, 43.5% DD score). The section “Low energy/Weak” has a total mean score in the range of probable difference (58.7% TP score, 2.2% PD score, 39.1% DD score). The others sections have a mean score in the range of typical performance (Table 1).

**Conclusion:** The Short Sensory Profile scale is now validated in Italian. The performance of the scales are in line with findings observed in the literature [4,5]. We confirm the existence of sensory impairments in ASD, particularly expressed as under-responsiveness or seeking stimuli and an increased or decreased response to auditory stimuli.

**References**

1. Ermer J, Dunn W. The sensory profile: a discriminant analysis of children with and without disabilities. Am J Occup Ther. 1998;52:283-90.

2. McIntosh DN, Miller LJ, Shyu, V, Dunn W. Development and validation of the short sensory profile. Sensory profile manual. 1999. 59-73.

3. Beaton DE, Bombardier C, Guillemin F, Ferraz MB. Guidelines for the process of cross-cultural adaptation of self-report measures. Spine (Phila Pa 1976). 2000;25:3186-91.

4. Tomchek SD, Dunn W. Sensory processing in children with and without autism: a comparative study using the short sensory profile. Am J Occup Ther. 2007;61:190-200.

5. Kern JK, Trivedi MH, Garver CR, Grannemann BD, Andrews AA, Savla JS, Johnson DG, Mehta JA, Schroeder JL. The pattern of sensory processing abnormalities in autism. Autism. 2006;10:480-94.

**Table 1 (abstract P44).** Summarize of the Short Sensory Profile data in our sample (N = 46)

	Minimum	Maximum	Mean	SD
Total Short Sensory Profile Score	119	176	147.68	15.047
Tactile Sensitivity	11.00	35.00	29.0435	4.82105
Taste/Smess Sensitivity	4.00	20.00	17.5652	4.23558
Movement Sensitivity	7.00	15.00	13.1739	2.56735
Underresponsive/Seeks Sensations	9.00	34.00	21.3913	6.75106
Auditory Filtering	11.00	27.00	19.6957	3.97699
Low Energy/Weak	12.00	30.00	25.0652	5.42178
Visual/Auditory Sensitivity	16.00	25.00	21.6522	2.89227

## P45 Return to work of cancer survivors in Europe: systematic review of the literature

### Sara Paltrinieri^1^, Stefania Fugazzaro^1^, Maria Chiara Bassi^2^, Martina Pellegrini^1^, Massimo Vicentini^3^, Claudio Tedeschi^1^, Elisa Mazzini^4^, Stefania Costi^1,5,6^

#### ^1^Physical Medicine and Rehabilitation Unit - Arcispedale Santa Maria Nuova-IRCCS, Reggio Emilia – Italy; ^2^ Medical library, Arcispedale Santa Maria Nuova-IRCCS, Reggio Emilia - Italy; ^3^ Interinstitutional Epidemiology Unit, AUSL Reggio Emilia, Reggio Emilia – Italy; ^4^ Medical Directorate, Arcispedale Santa Maria Nuova-IRCCS, Reggio Emilia - Italy; ^5^ Department of Surgery, Medicine, Dentistry and Morphological Sciences, University of Modena and Reggio Emilia, Modena – Italy; ^6^ Department of Neuroscience, Rehabilitation, Ophthalmology, Genetics and Maternal Child Health, University of Genoa, Genova – Italy

##### **Correspondence:** Stefania Costi (stefania.costi@unimore.it)

**Background and Objective:** Cancer incidence and survival are growing. Over 1/3 of cancer survivors (CSs) are in their working-age [1]. CSs experience pain, fatigue, cognitive dysfunction, mood disorders that may adversely affect social functioning [2].

Systematic reviews show 64% employment rate for CSs, with high variability in different contexts (range 24% -94%) [3]. We reviewed the recent literature on the employment rate of CS in Europe, investigating the factors influencing the return to work (RTW).

**Materials and Methods:** Bibliographic research was conducted in MEDLINE, CINAHL, EMBASE, PsycINFO, COCHRANE library from January 2010 to April 2017. Three independent researchers analyzed and critically evaluated each citation through the CASP [4]. We included european cancer population studies with remote follow-up. Table 1 shows the data extracted from each study. This study was supported by Chamber of Commerce, GRADE Onlus and Hospital IRCCS-ASMN of Reggio Emilia (Italy).

**Results:** Through the selection process we included 10 studies on 914 citations.

Investigated cohorts were diagnosed from 1995 to 2009, follow-up had an average duration of 2 years (range 0.2-23.4 years). The included samples range from 382 to 5074 working-age individuals. The most represented cancer locations were: breast (6038), genital and prostate (4021), gastrointestinal (1546), hematologic (1182), upper aero-digestive tract/lung (n.944), urogenital non-prostate (n. 933) (n. 311), head and neck (n. 23) and unspecified sites (n. 1250).

The rate of RTW fluctuate from 55.9% to 77%. Among the employed at the time of diagnosis RTW fluctuate from 60 to 84%. Factors associated with RTW are shown in Figure 1.

The results reflect the situation in Northern Europe. Southern Europe is completely not represented and Central Europe is scarcely represented.

**Conclusion:** There is urgent need of precise and up-to-date data collected in South and Central Europe, to allow for understanding if RTW is problematic in CSs and whether it requires socio-rehabilitative interventions to contain its potential impact on individuals and society.

**References**

1. Ferlay J, Soerjomataram I, Dikshit R, Eser S, Mathers C, Rebelo M, Parkin DM, Forman D, Bray F. Cancer incidence and mortality worldwide: sources, methods and major patterns in GLOBOCAN 2012. Int J Cancer. 2015;136:E359-86.

2. Spelten ER, Sprangers MA, Verbeek JH. Factors reported to influence the return o work of cancer survivors: a literature review. Psychooncology. 2002;11:124-31.

3. Mehnert A. Employment and work-related issues in cancer survivors. Crit Rev Oncol Hematol. 2011;77:109-30.

4. Casp UK. Critical Appraisal Skills Programme (CASP). Qualitative research checklist, 2017, 31: 13.

**Table 1 (abstract P45).** Data extracted for each study included in the review

Country	
Study objective	
Study design	
Main outcome measure	
Data collection strategy	
Data collection period	
Response rate	
Time since diagnosis	
Follow-up duration	
Inclusion criteria for target population	
Sample size	
Return to work rate	
Factors associated to RTW	
Sick leave	

**Fig. 1 (abstract P45).**
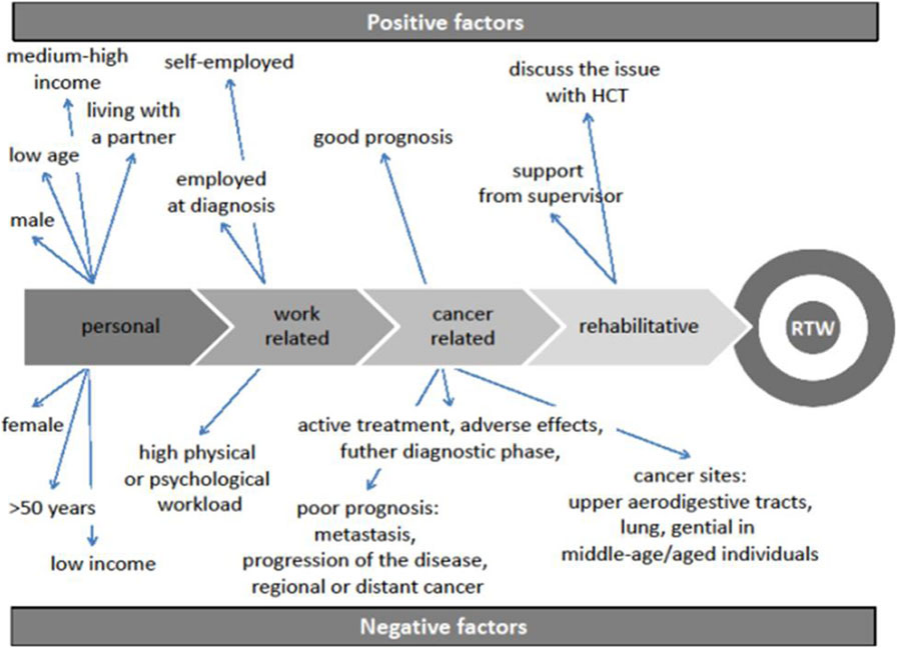
See text for description

## P46 Neural mobilization to improve motion and reduce pain hypersensitivity in hand osteoarthritis: A preliminary study

### Paolo Pedersini^1^, Alberto Borboni^2^, Stefano Negrini^1,2^, Jorge Hugo Villafañe^1^

#### ^1^IRCCS Fondazione Don Carlo Gnocchi, Milan, Italy; ^2^Università degli studi di Brescia

##### **Correspondence:** Paolo Pedersini (pedersini93@gmail.com)

**Background and Objective:** Pain in osteoarthritis (OA) is considered a complex integration of sensory and cognitive processes involving several abnormal cellular mechanisms at peripheral and central levels of the nervous system [1]. The peripherally directed therapies may modulate pain perception bilaterally. We hypothesized that these patients would show hypoalgesia of neural mobilization as compared to robotic assisted mobilization. Therefore, the purpose of this randomized controlled trial financed by “Ministero della Salute” from italy, is to examine the effects of nerves mobilization (NM) vs. robotic assisted passive mobilization of the hand on pain in sensitivity, hand function, analyze the quantitative and qualitative movement of hand in subjects with hand OA. The aim of the present preliminary study is to detail the protocol for a randomised controlled trial (RCT) of neural manual on pain in sensitivity as well as analyse the quantitative and qualitative movement of hand in subjects with hand osteoarthritis. We show some preliminary data about the group handled with NM.

**Materials and Methods:** Fourteen patients, aged 50 to 90 years old, with a diagnosis of hand OA, have been recruited. They received bilaterally an experimental intervention: NM of radial, ulnar and median nerves, plus exercise. Treatment took place for 12 sessions over 4 weeks. Evaluation consist of administration of: VAS, Quick-DASH, evaluation of grip/pinch strenght and pressure pain threshold (PPT) by mechanical pressure algometry of 6 points: Assessment points was been at baseline and end of therapy. The outcomes of this intervention was been pain and determine the central pain processing mechanisms.

**Results:** The analyses showed that patients with hand OA present bilaterally increased PPTs over the first CMC joint and median nerve as compared to pre-treatment (all, P<0.05). Similarly, tip pinch of the bilaterally increased did increase after treatment (P<0,05). Patients with hand OA also exhibited a hand right reduction in VAS than pre-treatment (P<0.05). A significant correlation was found between PPT over the ulnar nerve and QuickDASH (r=0.567, P=0.037).

**Conclusion:** Treatment shows a signifier increase of PPTs over the first CMC joint and median nerve. NM decreases pain in hand with OA and increases bilaterally pinch strength after treatment.

**References**

1. Dieppe PA, Lohmander LS. Pathogenesis and management of pain in osteoarthritis. Lancet. 2005;365(9463):965-73.

2. Dray A, Read SJ. Arthritis and pain. Future targets to control osteoarthritis pain. Arthritis Res Ther. 2007;9:212.

3. Villafañe JH, Bishop MD, Fernández-de-Las-Peñas C, Langford D. Radial nerve mobilisation had bilateral sensory effects in people with thumb carpometacarpal osteoarthritis: a randomised trial. J Physiother. 2013;59:25-30.

4. Villafañe JH, Fernandez de-Las-Peñas C, Silva GB, Negrini S. Contralateral sensory and motor effects of unilateral kaltenborn mobilization in patients with thumb carpometacarpal osteoarthritis: a secondary analysis. J Phys Ther Sci. 2014;26:807-12.

5. Villafañe JH, Cleland JA, Fernandez-de-Las-Peñas C. Bilateral sensory effects of unilateral passive accessory mobilization in patients with thumb carpometacarpal osteoarthritis. J Manipulative Physiol Ther. 2013;36:232-7.

**Table 1 (abstract P46).** Characteristic of the NM group. Values are expressed as mean

CHARACTERISTIC	MEAN	SD
Age	65,64	±5.68
Gender	10/14	(71,43% male)

**Fig.1 (abstract P46).**
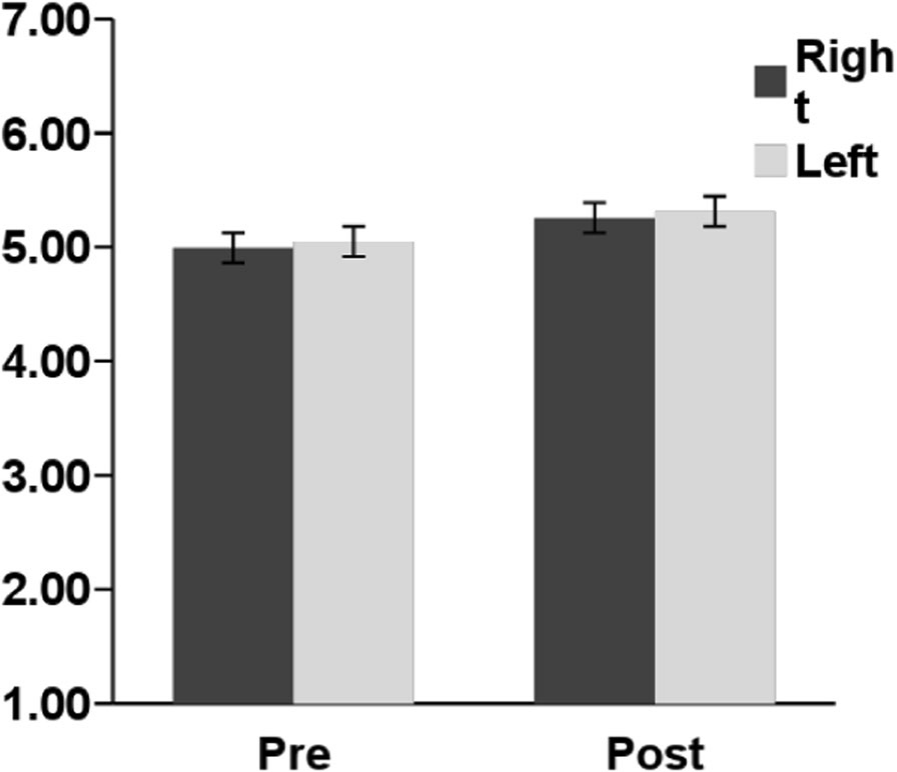
PPT on CMC joint

**Fig.2 (abstract P46).**
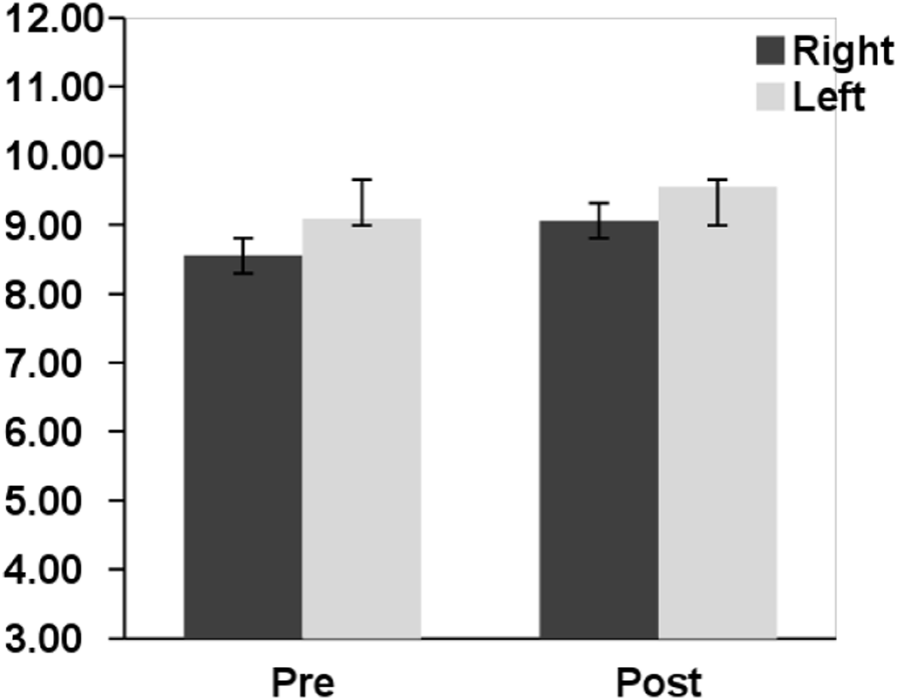
Pinch strength

## P47 Shoulder pain in patients with stroke is associated with Myofascial Trigger Points: a cross sectional study

### Paolo Pedersini^1^, Jorge Hugo Villafañe^1^, Maria Pilar López Royo^2^, Stefano Negrini^1,3^

#### ^1^IRCCS Fondazione Don Carlo Gnocchi, Milan, Italy; ^2^Universidad San Jorge, Zaragoza, Spain; ^3^Università degli studi di Brescia, Brescia, Italy

##### **Correspondence:** Paolo Pedersini (pedersini93@gmail.com)

**Background and Objective:** The aim of this study was to determine the prevalence of Myofascial Trigger Points (MTrPs) and the correlation between MTrPs and pain and function in a sample of patients presenting with shoulder pain following a stroke [1-3].

**Materials and Methods:** 50 consecutive stroke patients with shoulder pain (age range, 30-85 yrs) participated in the cross sectional study. The clinical assessments included [4]: palpation of the infraspinatus, supraspinatus, teres minor, and upper trapezius for clinical characteristics of a total of 4 MTrPs.

**Results:** The association of latent MTrPs and shoulder pain was estimated to have a point prevalence rate of 68%, 92%, 40% and 62% for supraspinatus, infraspinatus, teres minor, and trapezius upper muscle, respectively. The association between active MTrPs and shoulder pain were estimated to have a point prevalence rate of 34%, 50%, 12% and 20% for supraspinatus, infraspinatus, teres minor, and upper trapezius muscle respectively. Pain was measured with the VAS scale and was moderately correlated with the total prevalence of MTrPs (r=0.349; p=0.014) and active MTrPs (r=0.311; p=0.030) in the supraspinatus muscle. Disability was measured with the DASH and was moderately correlated with latent MTrPs in infraspinatus (r=0.308; p=0.030) and active MTrPs of supraspinatus (s=0.319; p=0.024).

**Conclusions:** This study shows that MTrPs may be a major source of pain and dysfunction in patents following a stroke. The criteria of “referred pain familiar to the patient” should be reconsidered when determining if MTrPs are active in this population [5].

**References**

1. van Bladel A, Lambrecht G, Oostra KM, Vanderstraeten G, Cambier D. A randomized controlled trial on the immediate and long-term effects of arm slings on shoulder subluxation in stroke patients. Eur J Phys Rehabil Med. 2017;53:400-409.

2. Chang MC. The effects of ultrasound-guided corticosteroid injection for the treatment of hemiplegic shoulder pain on depression and anxiety in patients with chronic stroke. Int J Neurosci. 2017;127:958-964.

3. Wofford JL, Mansfield RJ, Watkins RS. Patient characteristics and clinical management of patients with shoulder pain in U.S. primary care settings: secondary data analysis of the National Ambulatory Medical Care Survey. BMC Musculoskelet Disord. 2005;6:4.

4. Villafañe JH, Valdes K, Anselmi F, Pirali C, Negrini S. The diagnostic accuracy of five tests for diagnosing partial-thickness tears of the supraspinatus tendon: A cohort study. J Hand Ther. 2015;28:247-51

5. Campbell M. Problems With Large Joints: Shoulder Conditions. FP Essent. 2016;446:25-30.

**Fig. 1 (abstract P47).**
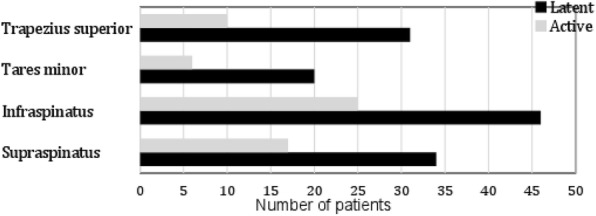
The number of latent (black bar) and active (grey bar) of MTrPs for patient

**Table 1 (abstract P47).** Performance of the combinations of 3 tests for latent and 4 tests for active MTrPs

	Sensitivity (%)	+ LR (95% CI)
Latent MTrPs, (Tb+NE+HN)		
Supraspinatus	68	1.96 (1.26 -3.07)
Infraspinatus	92	5.92 (2.33-15.08)
Teres minor	40	2.51 (1.86 -3.38
Upper trapezius	62	2.55 (1.70 -3.83)
Active MTrPs, (Tb+NE+HN+RP)		
Supraspinatus	34	2.35 (1.76-3.13)
Infraspinatus	50	2.33 (1.64 -3.32)
Teres minor	12	2.14 (1.72 -2.65)
Upper trapezius	20	1.31 (0.85-2.04)

## P48 Which patient-reported outcome measure has the best psychometric properties for Italian subjects with non-specific neck pain? A systematic review

### Leonardo Pellicciari^1^, Francesca Bonetti^2^, Damiano Di Foggia^3^, Mauro Monesi^4^, Stefano Vercelli^5^

#### ^1^Unit of Functional Rehabilitation, Azienda USL Toscana Centro, Empoli (FI), Italy; ^2^ Department of Clinical Sciences and Translational Medicine, Tor Vergata University, Rome, Italy; ^3^ Private practitioner, Rome, Italy; ^4^ Department of Neuroscience, Rehabilitation, Ophthalmology, Genetics, Maternal and Child Health, University of Genova - Campus of Savona, Savona, Italy; ^5^ Laboratory of Ergonomics and Musculoskeletal Disorders Assessment, Division of Physical Medicine and Rehabilitation, Istituti Clinici Scientifici Maugeri SpA-SB, IRCCS Veruno (NO), Italy

##### **Correspondence:** Leonardo Pellicciari (leonardo.pellicciari@gmail.com)

**Background and Objective:** To systematically review the validated Italian-language patient-reported outcome measures (PROMs) for subjects with non-specific neck pain (NP), providing insightful regarding their clinical utility.

**Methods:** Two reviewers independently searched MEDLINE, EMBASE, and CINAHL in June 2017 using the following keywords: psychometric, validity, reliability, responsiveness, neck pain, cervical pain. All articles published in English or Italian, studying subjects with acute, subacute and chronic NP and regarding the validation of PROMs available in the Italian language were included. Data about reliability, validity and responsiveness were extracted.

**Results:** The search carried out 4027 articles; 72 articles were included in this study (Figure 1). Four instruments measuring function and disability [Neck Disability Index (NDI), Neck Pain and Disability Scale (NPDS), Neck Bournemouth Questionnaire (NBQ), and Core Outcome Measures Index (COMI)], and one measuring activity-related fear of movement (NeckPix©), were identified. Data regarding their psychometric properties from Italian subjects are presented in Table 1.

The NDI showed important shortcomings regarding dimensionality (unidimensionality was achieved removing from 1 to 5 items among different studies), and responsiveness (related also to the large variability of measurement error).

There is no evidence about the unidimensionality of NPDS; the factor analysis on different versions showed 2 to 4 factors, and the items composing each factor were not consistent across the studies.

The NBQ was studied through classical theory tests and item response theory. The explorative and confirmatory factor analysis revealed 2 subscales; after removing item#7, the first factor fitted the Rasch model, while the second factor fitted the model without modifications.

The COMI had low responsiveness and inconsistence in the calculation of the total score.

The NeckPix® showed 1 factor, and good reliability and validity, but no data about its responsiveness were available.

**Conclusion:** Five PROMs are available to assess Italian subjects with NP. However, 4 of them showed psychometric weaknesses. NDI, COMI and NeckPix® reported problems with responsiveness, and NPDS with dimensionality. On the other hand, the NBQ demonstrated acceptable psychometric properties, and could be considered a valid instrument to measure disability in Italian subjects with NP.

**References**

1. Monticone M, Baiardi P, Nido N, Righini C, Tomba A, Giovanazzi E. Development of the Italian version of the Neck Pain and Disability Scale, NPDS-I: cross-cultural adaptation, reliability, and validity. Spine (Phila Pa 1976). 2008;33:E429-34.

2. Monticone M, Ferrante S, Vernon H, Rocca B, Dal Farra F, Foti C. Development of the Italian version of the Neck Disability Index: cross-cultural adaptation, factor analysis, reliability, validity, and sensitivity to change. Spine (Phila Pa 1976). 2012;37:E1038-44.

3. Geri T, Signori A, Gianola S, et al. Cross-cultural adaptation and validation of the Neck Bournemouth Questionnaire in the Italian population. Qual Life Res. 2015;24:735–45.

4. Monticone M, Ferrante S, Maggioni S, Grenat G, Checchia GA, et al. Reliability, validity and responsiveness of the cross-culturally adapted Italian version of the Core Outcome Measures Index (COMI) for the neck. Eur Spine J. 2014;23:863-72.

5. Monticone M, Vernon H, Brunati R, Rocca B, Ferrante S. The NeckPix(©): development of an evaluation tool for assessing kinesiophobia in subjects with chronic neck pain. Eur Spine J. 2015;24:72-9.

**Table 1 (abstract P48).** Psychometric properties of the patient reported outcome measures validated in Italian subjects with non-specific neck pain

Outcome measure	Dimensionality	Internal consistency	Reliability	Validity	Responsiveness	
					Distribution-based methods	Anchor-based methods
Neck Disability Index	2 factors: activity of daily living (F#1), pain and concentration (F#2)	Total: α=.84 F#1: α=.82 F#2: α=.72	ICC=.85 (95%CI.78-.89) F#1: ICC=.81 (95%CI .73-.87) F#2: ICC=.83 (95%CI .76-.88)	r_s_=.69 NPDS r_s_=.55 HADS-D r_s_=.44 NRS r_s_=.42 HADS-A	MDC= 3 points F#1: MDC=1 F#2: MDC=1 ES=.66 SRM=1.09 GRI=.70	MCID=3.5 points AUC=.96 (spec .81; sens .98)
Neck Pain and Disability Scale	3 factors: neck dysfunction related to general activities (F#1), neck pain and cognitive-behavioral aspects (F#2), neck dysfunction related to activities of the cervical spine (F#3)	Total: α=.94 F#1: α=.92 F#2: α =.86 F#3: α =.89	Total NPDS: r_s_=.91 F#1: r_s_=.89 F#2: r_s_=.93 F#3: r_s_=.92	r_P_=-.47 SF-36 r_P_=-.45 to -.17 SF-36 subscales	ES= .73 SRM=1.26 GRI=.73	MCID=10 points AUC= .91 (Sens .93; Spec .83)
Neck Bournemouth Questionnaire	2 factors: pain & functioning (F#1); anxiety & depression (F#2)	Total: α=.89 (95%CI.84-92) F#1: α=.88 (95%CI.83-92) F#2:α=.90 (95%CI.86-94)	Not studied	r=.67-.70 NPDS r=.63-.73 NRS	Not studied	MCID=5.5 points AUC=.72 (Sens. 75%; Spec. 60%)
Core Outcome Measure Index for neck pain	Not studied	Not studied	ICC=.87 (95%CI .81-.91)	Pain: r_p_=.45 NRS Pain: r_p_=.48 NPDS Function: r_p_=.49-.55 NPDS QoL: r_p_=-.44 EQ-5D Disability: r_p_=.45-.48 NPDS	MDC= 1.8/10 points SEM=.65/10 points SRM= 1.23	AUC: .73 (.62-.85) (Sens=.55; Spec=.88)
NeckPix®	1 factor	α=.95	ICC=.98 (95% CI .97-.98)	r_p_=.76 TSK r_p_=.58 PCS r_p_=.52 NDI r_p_=.45 NRS	Not studied	Not studied

Legend: ICC: Intraclass Correlation Coefficient; CI: Confident Interval; r_s_: Spearman’s Correlation Coefficient; NPDS: Neck Pain Disability Scale; HADS-D: Hospital Anxiety and Depression Scale of Depression; NRS: Numerical Rating Scale; HADS-A: Hospital Anxiety and Depression Scale of Anxiety; MDC: Minimal Detectable Change; ES: Effect Size; SRM: Standardized Response Mean; GRI: Guyatt’s Responsiveness Index; MCID: Minimal Clinical Important Difference; AUC: Area Under the Curve; Sens: Sensibility; Spec: Specificity; SF-36: Short Form-36; r: Correlation Coefficient; EQ-5D: Euroqol 5-Dimensions; SEM: Standard Error of Measurement; TSK: Tampa Scale of Kinesiofobia; NDI: Neck Disability Index

**Fig. 1 (abstract P48).**
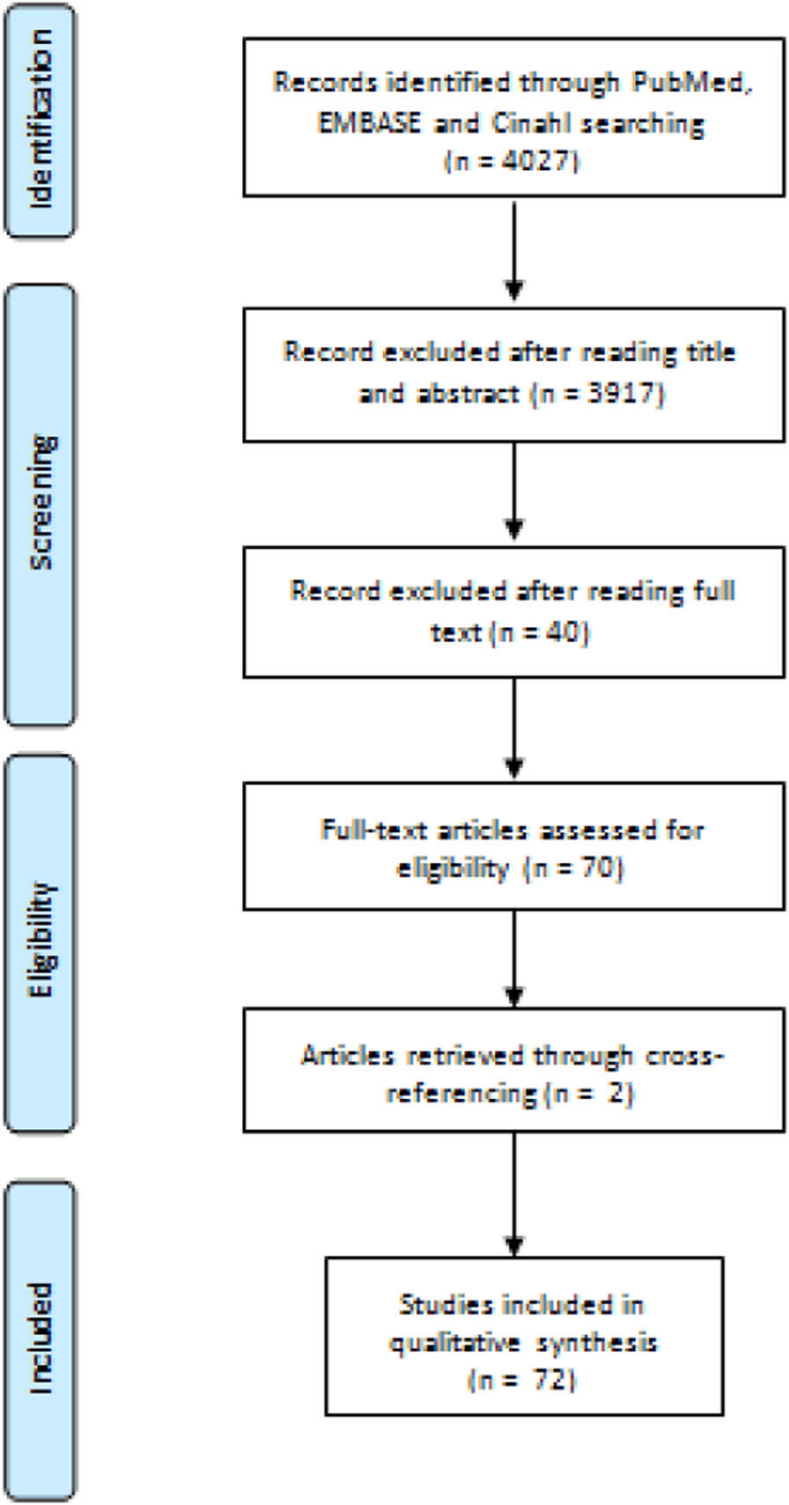
Flow-chart of the studies selection

## P49 Effect of rehabilitation after selective dorsal rhizotomy: case series of Gaslini Children’s Hospital

### Alice Perata, Carla Ferrari

#### Istituto Giannina Gaslini, Genova, Italy

##### **Correspondence:** Alice Perata (alice.perata@gmail.com)

**Background and Objective:** Selective dorsal rhizotomy (SDR) is an irreversible neurosurgical technique that aims to reduce the muscular tone of selected muscle groups [1-3].

The mini-invasive approach, which was developed in Saint Louis Children's Hospital and performed for the first time in Italy in 2016, allows to reduce recovery times and complication’s number [4,5]. The aim of the study is to highlight the importance of rehabilitation and to better define the role of physiotherapist in the individual rehabilitation program for children with Cerebral Palsy, who were operated on SDR with mini-invasive technique, starting from the experience of the Gaslini Children’s Hospital.

**Materials and Methods:** This study presents a case series on the 3 minors (2 females and 1 male) operated at the Gaslini’s Hospital, who also performed a post-surgical intensive rehabilitation period under the Day Hospital (DH) [4,5]. This observational study includes a summary of the clinical history of patients, rehabilitation treatments performed and evaluation results, collected in graphs and tables.

**Results:** The results of the study showed, for all three cases, an initial worsening of motor functions, followed by a gradual improvement during both intensive and maintenance rehab. However, the improvement was only found in a few items in the selected tests. The treatment is focused on a more standardized initial phase and a phase in DH, customized on the goals of individual children.

**Conclusion:** Since the intervention of selective rhizotomy by mini-invasive technique has been performed for the first time in Italy in 2016, it seems that this study will be useful to better understand the role of physiotherapist and to understand the importance of Rehabilitation in teams, into the Italian hospitals. Rehabilitation and physiotherapy play a crucial role in the recovery of motor skills and autonomy in Activities of Daily Living even in little patients who was operated on SDR. Physiotherapists must also ensure a constant comparison with the various professionals, promote multidisciplinary care and seek a family-centered approach [4].

**References**

1. O'Brien DF, Park TS. A review of orthopedic surgeries after selective dorsal rhizotomy. Neurosurg Focus. 2006 Aug 15;21(2):e2.

2. “Clinical comminssioning policy statement: Selective Dorsal Rhizotomy (SDR)” – NHS Commissioning Board, April 2013

3. National Collaborating Centre for Women's and Children's Health (UK). Spasticity in Children and Young People with Non-Progressive Brain Disorders: Management of Spasticity and Co-Existing Motor Disorders and Their Early Musculoskeletal Complications. London: RCOG Press; 2012 Jul.

4. “Spasticity in under 19s: management” – NICE Clinical Guideline n.145, 25 July 2012

5. “Selective dorsal rhizotomy for spasticity in cerebral palsy” – NICE Interventional Procedure Guidance n.373, 15 December 2010

## P50 Responsiveness of the Instrumented Timed Up & Go test in elderly neurological patients

### Michela Picardi, Antonio Caronni, Irma Sterpi, Luciana Sciumè, Paola Antoniotti, Evdoxia Aristidou, Fortunati Nicolaci, Giuseppe Pintavalle, Valentina Redaelli, Gianluca Achille, Massimo Corbo

#### Department of Neurorehabilitation Sciences Casa di Cura del Policlinico, Milano, Italy

##### **Correspondence:** Michela Picardi (michelapicardi.ft@gmail.com)

**Background and Objective:** The Timed Up and Go (TUG) test is a common outcome measure in rehabilitation and shortening of the total TUG duration (TTD) marks the improvement of the patient's performance [1]. However, when a patient shortens his/her TTD the clinician wonders whether this modification reflects the homogeneous improvement of all the TUG phases or the improvement of only some of these. The instrumental TUG test (ITUG; i.e. the TUG measured by inertial sensors, IS) makes it possible to explore this issue [2,3]. In the current work we explored the ITUG test modification after rehabilitation. These results are discussed in the responsiveness framework.

**Materials and Methods:** Seventy-six (mean age: 76.6 years, SD: 6.1, 35 females) older adults with a neurological disease were recruited (acute group, AG; n=33; chronic group, CG, n=43). All patients participated to an inpatient physiotherapy program. Participants completed the ITUG on admission (T0) and discharge (T1) with an IS secured to their back. IS signals were used to split the TUG into subsequent phases (sit-to-stand, walk1, turn1, walk2, turn2, turn-and-sit). ITUG phases duration and TTD were measured. The Wilcoxon signed rank and rank sum tests were used for within- and between-groups comparison, respectively. The Cohen’s d was calculated as a responsiveness index [4].

**Results:** TTD, walk1 and walk2 duration only showed the expected pattern of patient’s improvement. TTD, walk1 and walk2 were significantly shorter at T1 than T0 in both the AG and CG (within-groups difference). At T1, TTD, walk1 and walk2 were significantly shorter in AG than CG (between-groups difference), while no between-groups difference was present at T0. Sit-to-stand and turn-and-sit showed within-groups differences in both groups, but no between-groups difference. Turn1 and turn2 showed within-groups difference in AG only. AG TTD, AG walk1 and walk2 showed the largest effect sizes (Table1).

**Conclusion:** The TTD, walk1 and walk2 duration were sensitive in detecting changes in elderly neurological patients. In chronic neurological patients, shortening of the TTD after rehabilitation probably reflects the improvement of walking and transfers. Clinicians interested in demonstrating the modification of turning should use the ITUG measures rather than inferring it from the improvement of the TTD.

**References**

1. Podsiadlo D, Richardson S. The timed "Up & Go": a test of basic functional mobility for frail elderly persons. J Am Geriatr Soc. 1991;39:142-8.

2. Weiss A, Herman T, Plotnik M, Brozgol M, Giladi N, Hausdorff JM. An instrumented timed up and go: the added value of an accelerometer for identifying fall risk in idiopathic fallers. Physiol Meas. 2011;32:2003-18.

3. Weiss A, Mirelman A, Buchman AS, Bennett DA, Hausdorff JM. Using a body-fixed sensor to identify subclinical gait difficulties in older adults with IADL disability: maximizing the output of the timed up and go. PLoS One. 2013;8:e68885.

4. Sawilowsky SS. New effect size rules of thumb. J Mod Appl Stat Methods. 2009;8:597-9.

**Table 1 (abstract P50).** See text for description

ITUG	acute group (AG)		chronic group (CG)
	Cohen's d	Effect size	Cohen's d
sit to stand	0.78	large	0.33
walk 1	1.07	large - very large	0.36
turn 1	0.84	Large	0.21
walk 2	0.97	Large	0.39
turn 2	0.83	large	0.25
turn and sit	0.93	large	0.57
total TUG (TTD)	1.13	large - very large	0.45

## P51 Effects of soft tissue mobilization (STM) manual techniques on postsurgical scar adherences: an observational study

### Diego Poddighe^1^, Matteo Moroso^2^, Elisabetta Bravini^3^, Francesco Sartorio^4^, Stefano Vercelli^4^

#### ^1^Physiotherapy Student at University of Insubria, Varese, Italy; ^2^Physiotherapy Student at University of Piemonte Orientale, Novara, Italy; ^3^Italian Society of Physiotherapy, Firenze, Italy; ^4^Laboratory of Ergonomics and Musculoskeletal Disorders Assessment, Division of Physical Medicine and Rehabilitation, Istituti Clinici Scientifici Maugeri SpA-SB, IRCCS Veruno, Italy

##### **Correspondence:** Diego Poddighe (diego.poddighe@gmail.com)

**Background and Objectives:** Scar adherence is the failure of tissues to successfully establish independent layering, reducing skin and joint mobility. This frequently occurs after surgery, and may have a severe impact on body function and quality of life. Manual therapy is one of the most widespread treatment option for pathological scars in rehabilitation, and two recent case studies [1,2] demonstrated preliminary clinical improvements in scar mobility with the use of soft tissue manual techniques (STM). Aim of this observational study was to analyze the effects of STM on postsurgical scars adherences.

**Material and Methods:** All patients referred to the Istituti Clinici Scientifici Maugeri SpA-SB for orthopaedic postsurgical rehabilitation from May 2015 to August 2017 were considered eligible in this cohort observational study. Severity of scar adherence was measured by the Adheremeter, a validated outcome measure that allow to calculate the Adherence Severity index (AS, score range: 0 to 1) [3]. Inclusion criteria was an AS <0.49 at the worst scar point. Measurements were repeated after treatment. Patients enrolled were treated with STM (Figure 1) for 5 to 15 sessions of about 20 minutes each, twice a week. Treatment effect was analyzed with Student t-test for paired data (significance level set at p<0.05) and Effect Size. The proportion of patients who had changes greater than the minimal detectable change (MDC) of AS (that is 0.20), was also used to determine clinical treatment effects. Statistical power was assessed post-hoc.

**Results:** A total of 19 patients were considered eligible and were included in the study. The pre-post treatment effect of the AS index was statistically significant (p<0.001), with large Effect Size (Table 1). A moderate number of subjects reached or passed the MDC in this sample (Table 1). The post-hoc analysis revealed a 100% statistical power.

**Discussion:** STM is aimed at restoring scar pliability and reduce the adherence severity by improving soft tissue layering under the scar. The results of this study suggest that STM treatment was statistically and clinically effective to improve scar mobility in a population of subjects with a moderate-to-severe scar adherence severity. A post-hoc analysis of scores distribution revealed that patients with more severe adhesive scars showed lower improvements than those who had a less severe condition at baseline. This means that the AS may represent also a valid prognostic index. The main limitation of this study was the lack of a control group, warranting further investigations.

**Conclusions:** The STM manual techniques produced a moderate effect on mobility of adherent scars, independently of their adhesion severity at baseline. More studies are necessary to better define the most effective duration and frequency of treatments. Other manual or instrumental techniques can be also compared in the future, in order to determine which intervention is the most beneficial in treating adherent scars.

**References**

1. Vercelli S, Ferriero G, Sartorio F, Foti C. Assessment and manual treatment of adhesive scars: a case report. It J Physiother. 2011;1:55-9.

2. Wasserman JB, Steele-Thornborrow JL, Yuen JS, Halkiotis M, Riggins EM. Chronic caesarian section scar pain treated with fascial scar release techniques: A case series. J Bodyw Mov Ther. 2016;20:906-913.

3. Ferriero G, Vercelli S, Salgovic L, Stissi V, Sartorio F. Validation of a New Device to Measure Postsurgical Scar Adherence. Phys Ther. 2010;90:776-783.

**Fig. 1 (abstract P51).**
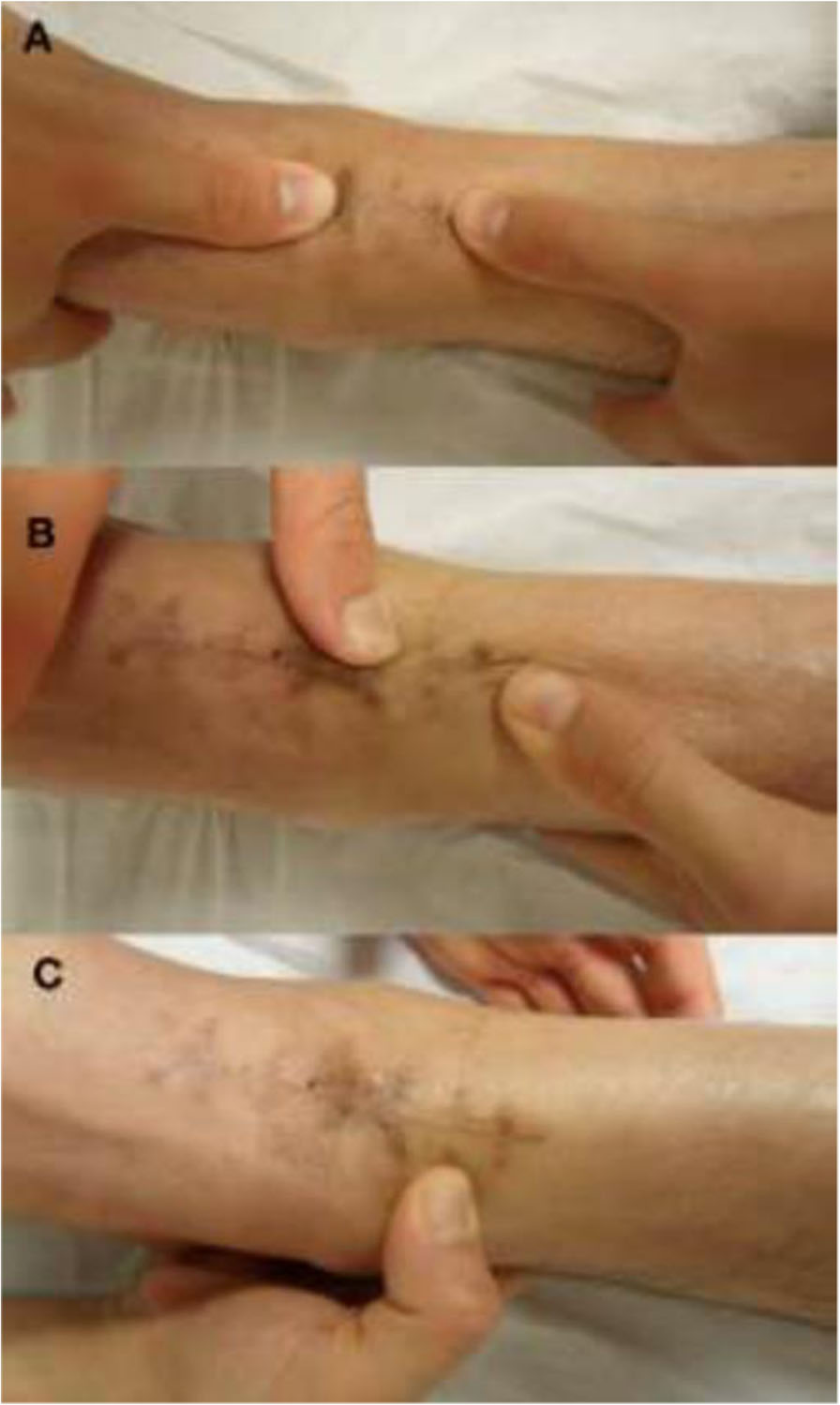
Soft Tissue Mobilization is a system of manual techniques employing specific, graded and progressive forces applied in multidirectional vectors to improve mobility between overlying and adjacent connective tissue layers. Three techniques were used: push-pull (a), indian burn (b), and J stroke (c)

**Table 1 (abstract P51).** See text for description

	Baseline mean (SD)	After treatment mean (SD)	Mean change (SD)	t-test (p)	Effect size	Proportion of subjects changed > MDC
Adherence Severity Index (AS)	0.15 (±0.12)	0.44 (±0.26)	0.29 (0.19)	<0.001	2.38	58% (N=11/19)

## P52 Efficacy of therapeutic exercise in the management of headache: a systematic review

### Elisa Ravizzotti (elisa.ravizzotti@gmail.com)

#### Private practitioner, Varese, Italy

**Background and objectives:** Headache is a widespread disabling disorder. Main classification distinguishes primary and secondary headache but an appropriate diagnosis is often unclear, consequently this problem is underestimated and under-treated. Pain is a common feature in all kind of headache characterized by intensity, frequency and duration. Conservative treatments targeting headache include medications, patient education, lifestyle modification and a physical therapy. The aim of this review is to evaluate the efficacy of therapeutic exercises in headache disorders.

**Material and Methods:** A systematic literature review of randomized clinical trial studies was conducted searching in Pubmed, PEDro, and Cochrane Library databases. The search was performed by combining the Mesh terms ("Headache Disorders" OR Headache) AND ("therapeutic exercise*" OR exercise OR "Exercise Movement Techniques" OR "Exercise Therapy"). Only studies with PEDro Scale score ≥5, written in English, were considered. Any limitation about publication period was set.

**Results:** The trials flaw is shown in Figure 1. Ten studies were identified and analysed (Table 1). Outcomes considered were expressed in at least one of the following headache parameters: intensity (numeric pain rating scale 0-10, visual analogue scale, Borg Category Ratio-10), frequency (days/weekly, days/monthly) and duration (hours/day). Intensity decreased significantly with stretching exercises addressed to the neck (1) and shoulder, (2) correcting posture (2) and through relaxation exercises (2,4). Frequency decreased significantly with relaxation and stretching exercises for neck/shoulder (2). Therapeutic exercises reduced significantly headache duration specially if combined with manipulative therapy (5) Low-load endurance exercises, with elastic resistance, improved all headache parameters also after 12 months (5). General training did not improve any parameters of headache more than common relaxation or medication treatments. (3)

**Discussion:** Nine studies considered primary headache, only one study (5) analysed therapeutic exercise effects on cervicogenic secondary headache and its results were boded well and the trial showed good quality methodology (7/10 PEDro scale). This review considered effects on headache pain, but therapeutic exercise could influence also neck/shoulder pain, strength of upper extremities, aerobic capacity, quality of life and general health. Further studies would help to understand the best exercise in different type of headache.

**Conclusion:** Therapeutic exercise seems to be effective for primary and secondary headache however their application should be examine in depth.

**References**

1. Lin LY, Wang RH. Effectiveness of a neck stretching intervention on nurses' primary headaches. Workplace Health Saf. 2015 Mar;63(3):100-6.

2. Mongini F, Evangelista A, Milani C, Ferrero L, Ciccone G, Ugolini A, Piedimonte A, Sigaudo M, Carlino E, Banzatti E, Galassi C. An educational and physical program to reduce headache, neck/shoulder pain in a working community: a cluster-randomized controlled trial. PLoS One. 2012;7(1):e29637.

3. Varkey E, Cider A, Carlsson J, Linde M. Exercise as migraine prophylaxis: a randomized study using relaxation and topiramate as controls. Cephalalgia. 2011 Oct;31(14):1428-38.

4. Kumar S, Raje A. Effect of progressive muscular relaxation exercises versus transcutaneous electrical nerve stimulation on tension headache: A comparative study. Hong Kong Physiotherapy Journal. 2014; 32(2):86-91.

5. Jull G, Trott P, Potter H, Zito G, Niere K, Shirley D, Emberson J, Marschner I, Richardson C. A randomized controlled trial of exercise and manipulative therapy for cervicogenic headache. Spine (Phila Pa 1976). 2002 Sep 1;27(17):1835-43

**Fig. 1 (abstract P52).**
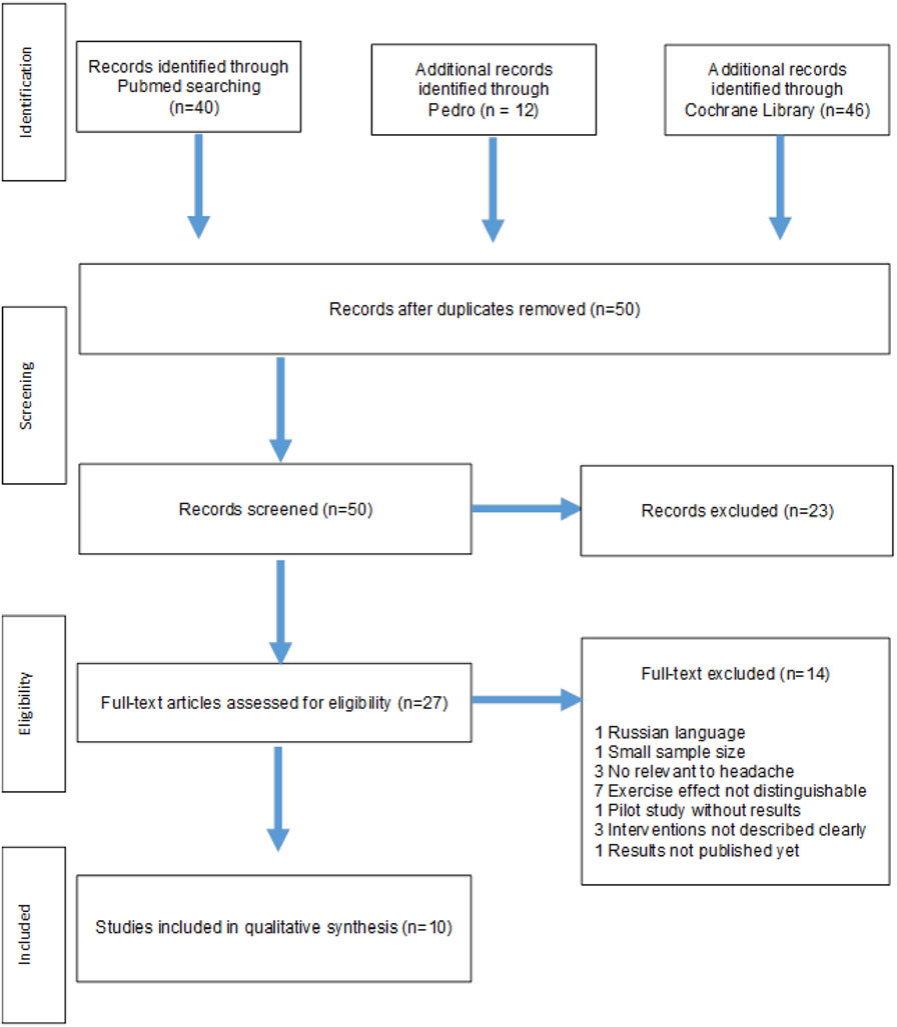
Flowchart of review based on PRISMA guidelines

**Table 1 (abstract P52).** Basic studies characteristics

Author	Headache type	Therapeutic exercise
Li-Ying Lin, et al. 2015	Primary headache	Neck stretching exercises included (a) sit or stand in a comfortable and relaxed position; (b) slowly turn the head and neck from side to side; (c) slowly stretch the neck in any direction, especially in the direction that is painful for 10 seconds repeating for 20'
Mognoni F. et al. 2012	TTH, migraine, myogenus neck/shoulder pain	Relaxion exercise daily Posture and stretching exercises for neck and shoulder Daily every 2-3 hours
Varkey E. et al. 2011	Migraine with/without urea	Training 40 minutes, three times a week. (indoor cycling)
Andersen L. et al. 2011	TTH, migraine, unknown	2 or 12 minutes of progressive neck/shoulder resistance training with elastic resistance tubing performed 5 times a week at the workplace
Söderberg E. et al. 2011	TTH chronic	Physical training group with five exercises focused on neck and shoulder muscles and similar home-training programme
van Ettekoven H et al. 2006	TTH (episodic, chronic)	Craniocervical training programme (CTP) using low-load endurance exercises to cervicoscapular and craniocervical regions using a latex band CPT also at home twice a day for 10 min per session and then at least twice a week
Sjögren T. et al. 2005	General headache	Progressive light resistance training with six dynamic symmetrical movements: upper extremity extension, upper extremity flexion, trunk rotation to the right, trunk rotation to the left, knee extension and knee flexion. 20 times with a 30 s pause between the training movements, three group sessions of 20’
Jull G, et al. 2002	CCH	6 weeks, included 8/12 treatments no longer than 30' :Therapeutic low-load endurance exercises twice daily: craniocervical flexion with/without feedback; exercises of scapular adduction and retraction; isometric exercises using a low level of rotatory resistance to flex-ext neck Postural correction exercises in the sitting position (muscle lengthening exercises if necessary) 6 weeks with 8-12 treatments max 30 minutes
Kumar S, Raje A 2014	TTH (chronic, frequent, infrequent)	Unilateral progressive muscular relaxation exercises on 4 muscle groups: tense the muscle group for 5/7 seconds and then relax for 30-40 seconds. 15-minute session of relaxation per day, for 7 days.
Tornoe Andersen LL et al. 2016	TTH (frequent episodic or chronic)	10 weeks of supervised progressive specific strength training with a focus on the trapezius muscles with resistive tubing elastics three times a week at home with the aid of parental support for 10 weeks

TTH= Tension-type headache, CCH= cervicogenic headache

## P53 Innovation, training, rehabilitation, and swim drills by the tool with methods REVFIN

### Giuseppe Righini^1^, I. Boriani^2^, G.Torriani, S. Longoni

#### ^1^L.U.de.S. Lugano (CH) Cdl. In Fisioterapia; UCSC Milano CDL S. Motorie; ^2^Centro studi ReVFIN

##### **Correspondence:** Giuseppe Righini (gi.righigi@alice.it)

All those water strength exercises and endurance building up drills which are needful in athletic conditioning disciplines get significant improvements by REVFIN tool. The innovation comes from its special design that adds Drag to the movement in the water and allows modulating it as desired. Because of this peculiarity, REVFIN is particularly helpful in hydro-kinesitherapy and water rehabilitation as well. The principle is very simple: the Drag force is applied to the feet by the water that flows through the resistant surface offered by a paddle. REVFIN is a complete and versatile tool made of a comfortable ecological rubber shoe underneath which is firmly bound the paddle that can be oriented under the sole, in the so called Classic Mode, or in front of the foot. The latter case is called Extended Mode. The workouts performed by means of REVFIN in Classic Mode take advantage from the water fluid-dynamics that imposes the resultant of the resistant forces to be directed perpendicularly to the sole with the application point at the ankle. This opens up to a series of benefits in workouts related to both athletic training and spine pathologies rehabilitation. In fact, when the body weight is discharged by the water flotation the stretching of the rachis is at its higher degree, thanks to the Drag force. In addition to that, the drills performed in horizontal position, like swimming, enhance the arms and legs muscles work due to higher effort to be applied in order to move. On the other side, the drills executed in vertical position, and particularly those that involve the hip extensors, do not overload unduly the ankles and the quadriceps because of the resistant force which in axis with the movement.

**References**

1. Righini G. Mi alleno nuotando con stile. Ed. Carabà. 2016

2. Barbosa TM, Ramos R, Silva AJ, Marinho DA. Assessment of passive drag in swimming by numerical simulation and analytical procedure. J Sports Sci. 2018 Mar;36(5):492-498.

3. Hazrati P, Sinclair PJ, Spratford W, Ferdinands RE, Mason BR. Contribution of uncertainty in estimation of active drag using assisted towing method in front crawl swimming. J Sports Sci. 2018 Jan;36(1):7-13.

4. Zhan JM, Li TZ, Chen XB, Li YS. Hydrodynamic analysis of human swimming based on VOF method. Computer methods in biomechanics and biomedical engineering, 2017, 20.6: 645-652.

## P54 High intensity training®: a new approach to rehabilitation in recovered subjects having cystic fibrosis

### Marco Rivolta^1^, Luigi Graziano^2^, Tamara Perelli^2^, Beniamino Giacomodonato^2^, Matteo De Marchis^2^, Emanuele Mechelli^2^, Alessandro Di Vito^1^

#### ^1^San Giovanni Addolorata Hospital, Rome, Italy; ^2^Policlinico Umberto I, Rome, Italy

##### **Correspondence:** Marco Rivolta (marco.rivolta94@gmail.com)

**Background and Objective:** Physical activity in people affected by cystic fibrosis (CF) determines beneficial effects on aerobic exercise capacity, lung function, and improved health-related quality of life. Anyway, we know little about physical exercise modality in adults affected by cystic fibrosis.

The purpose of this study is to evaluate the effects of an in-hospital training program that combines muscular strength training and aerobic capacity training during the same treatment session, to determine a distinct training program.

**Materials and Methods:** This study involved two groups of participants. All participants had to be older than16 years, have a FEV1>40% and they had not to have fever at their ward entrance. The comparison group, performed a standard rehabilitation treatment (respiratory physical therapy and aerobic capacity training), while the experimental group subjected to a new training program for hospitalized patients. Each session, lasted one hour and being supervised by a physical therapist. It was composed of different exercises for strength training, the rest period was replaced by aerobic exercise. The training intensity is established according to patients’ physical and health conditions at their hospital admission.

**Results:** We found a non-statistically significant difference (p=0.12) regarding participants preferences favouring the experimental group. This encourages the pursuance of the study on a larger sample size, to investigate the High Intensity Training® role to promote physical activity adherence people with CF hospitalized for bronchopulmonary exacerbation.

**References**

1. Savi D, Di Paolo M, Simmonds N, Onorati P, Internullo M, Quattrucci S, Winston B, Laveneziana P, Palange P. Relationship between daily physical activity and aerobic fitness in adults with cystic fibrosis. BMC Pulm Med. 2015 May 9;15:59.

2. Hebestreit H, Schmid K, Kieser S, Junge S, Ballmann M, Roth K, Hebestreit A, Schenk T, Schindler C, Posselt HG, Kriemler S. Quality of life is associated with physical activity and fitness in cystic fibrosis. BMC Pulm Med. 2014 Feb 27;14:26. doi: 10.1186/1471-2466-14-26.

3. Radtke T, Nolan SJ, Hebestreit H, Kriemler S. Physical exercise training for cystic fibrosis. Cochrane Database Syst Rev. 2015 Jun 28;(6):CD002768.

4. Hebestreit H, Kriemler S, Radtke T. Exercise for all cystic fibrosis patients: is the evidence strengthening? Curr Opin Pulm Med. 2015 Nov;21(6):591-5.

5. Williams CA. Physical activity and health of adults with cystic fibrosis. Respirology. 2016;21(3):404-5.

## P55 “*Approccio sequenziale propedeutico”* (ASP) for trunk control in persons with spinal cord injury with high levels at level T7

### Manuel Rocco^1^, Tatiana Bianconi^2^, Dalia Eleonora Croce^3^, Marco A. Mangiarotti^4^

#### 1University of Pisa, Pisa, Italy; ^2^USU – ASST Grande Ospedale Metropolitano Niguarda Milano, Milano, Italy; ^3^University of Milano, Milano, Italy; ^4^Responsabile Scientifico ANIK (Associazione Nazionale Idrokinesiterapisti)

##### **Correspondence:** Manuel Rocco (manuel.rocco88@gmail.com)

**Background and Objective:** Spinal Cord Injury (SCI) is an event that occurs when the spinal cord interrupts, partially or totally and causes changes in its function, either temporary or permanent. These changes translate into loss of muscle function, sensation, or autonomic function in parts of the body served by the spinal cord below the level of the lesion. In Italy the incidence of the SCI is about 20/25 new cases per million inhabitants per year. The person with SCI starting to develop new postural control models. These subjects accuse a delay in postural reactions so sitting posture is one of the main goals in rehabilitation. Aquatic therapy is an intervention used to improve trunk balance, recruitment and postural control. It incorporates slow movements of progressive difficulty.

The study is to evaluate the effectiveness of Aquatic Therapy using sequences of the *Approccio Sequenziale Propedeutico* (ASP) of the Associazione Nazionale Idrokinesiterapisti (ANIK) on trunk control in subjects with SCI without voluntary control of abdominal muscles.

**Materials and Methods:** The study was conducted in Unità Spinale Unipolare (USU) of the ASST Grande Ospedale Metropolitano Niguarda, between January and May 2017. Six male participants, between 16 and 65 years old have been enrolled, 4 presenting cervical SCI and 2 a thoracic SCI (T4). The patients participated in an individualized aquatic therapy (ASP) program one time a week for 8 weeks. Trunk balance is measured with Sitting Balance Assessment for spinal cord injury (SBA-sci) and Spinal Cord Independence Measure (SCIM) were collected pre(T0), mid-term (T1) and post therapy (T2).

**Results:** Five patients completed individualized aquatic therapy (ASP) program (n = 5; 1 drop-out). Wilcoxson Signed-Rank Test was used. After the treatment (T1-T2) subjects showed a significant improvement of trunk balance measured SBA-sci (-2.02 ± 1.96; p<0.05). No statistically significant improvement of SCIM (-1.60 ± 1.96; p>0.05).

**Conclusion:** Our results show that ASP is effective for improving trunk control in patients with SCI without voluntary control of abdominal muscles. There was no association between increase of SBA-sci scale and the SCIM scale.

**References**

1. Marinho-Buzelli AR, Rouhani H, Masani K, Verrier MC, Popovic MR. The influence of the aquatic environment on the control of postural sway. Gait Posture. 2017 Jan;51:70-76.

2. Cavuoto F, Mangiarotti MA. La riabilitazione in acqua secondo il metodo A.S.P. Arti Grafiche Rugantino Roma 2010.

3. Preuss R, Fung J. Musculature and biomechanics of the trunk in the maintenance of upright posture. J Electromyogr Kinesiol. 2008 Oct;18(5):815-28.

4. Cole AJ, Becker BE. Comprehensive aquatic therapy. Butterworth-Heinemann, 2004.

5. Bolin I, Bodin P, Kreuter M. Sitting position - posture and performance in C5 - C6 tetraplegia. Spinal Cord. 2000 Jul;38(7):425-34.

**Table 1 (abstract P55).** See text for description

	ASIA	L.O.M.	T0			T1				T2			
			Ashworth	SBA-sci	SCIM	Ashworth	SBA-sci	SCIM	Water	Ashworth	SBA-sci	SCIM	Water
pz1	C4C	✔	4	25.5	27	INV.	30	28	42	INV.	35	28	46
pz2	C5 A	✔	0	7.5	22	INV.	7.5	27	62	INV.	-	-	-
pz3	C5 A	✔	2	12.5	32	INV.	13	39	78	INV.	24	39	115
pz4	C5-C6 A		0	15	42	INV.	15	43	61	INV.	23	45	91
pz5	D4 A	✔	3	18	40	INV.	23	50	76	INV.	30	61	100
pz6	D4 A	✔	0	12	30	INV.	17	40	55	INV.	19.5	66	76

## P56 The use of ICF in Parkinson’s disease: potentiality and limits

### Helena Romano^1^, Franca Tirinelli^2^

#### ^1^Como; ^2^Ospedale San Giovanni Battista ACISMOM Roma

##### **Correspondence:** Franca Tirinelli (franca1965@alice.it)

**Background and Objective:** The present research aims to find the points of contact between Parkinson’s disease (PD) and the International Classification of Functioning, Disability and Health (ICF), through a methodological approach.

**Materials and Methods:** The study is divided into two parts.

In the first part, 11 experts in PD, (9 professionals and 2 patients), have identified the most relevant ICF categories of the disease. The agreement among responses and their congruence across different domains have been investigated with the intraclass correlation coefficient (ICC) and the Cronbach’s alpha. In the second part, a group of 10 patients has been evaluated with the Unified Parkinson's Disease Rating Scale (UPDRS)-part III, the Parkinson's Disease Quality of Life Questionnaire (PDQ-39), the Hoehn and Yahr rating scale (H&Y) and the ICF categories emerged in the initial phase of the research. The Pearson correlation coefficient (p) and the Mann Whitney's U test have been used to verify the presence of a correlation between ICF categories and rating scales.

**Results:** For the first part of the study, the 25 ICF categories with the highest frequency have been selected. The values of Cronbach's alpha (0.783) and ICC (0.783) provide evidence that the choice of the ICF categories is consistent. In the second part, the data analysis shows a correlation between the total ICF and the sections of the PDQ-39 concerning communication (p=0.083) and social support (p=0.07). Individual b320 categories (which are functions of voice articulation) and the s110 ones (brain structure) turn out to be correlated to the H&Y staging. No correlations have been observed between ICF and UPDRS.

**Conclusion:** During the selection of the ICF categories relative to the PD, some issues of interpretation emerged in the domain of *Body Structures*.

The lack of correlation between ICF and UPDRS-part III and, by contrast, the presence of correlation between ICF and PDQ-39 emphasize the imbalanced development of motor and non-motor symptoms of PD, and they display the important role played by the latter in life quality.

**References**

1. World Health Organization. International Classification of Functioning, Disability and Health: ICF. World Health Organization, 2001.

2. Raggi A, Leonardi M, Ajovalasit D, Carella F, Soliveri P, Albanese A, Romito L. Disability and profiles of functioning of patients with Parkinson's disease described with ICF classification. Int J Rehabil Res. 2011 Jun;34(2):141-50.

3. Selb M, Escorpizo R, Kostanjsek N, Stucki G, Üstün B, Cieza A. A guide on how to develop an International Classification of Functioning, Disability and Health Core Set. Eur J Phys Rehabil Med. 2015 Feb;51(1):105-17.

4. Peto V, Jenkinson C, Fitzpatrick R. PDQ-39: a review of the development, validation and application of a Parkinson's disease quality of life questionnaire and its associated measures. J Neurol. 1998 May;245 Suppl 1:S10-4.

5. World Health Organization. The Global Burden of Disease: World Health Organization. 2008.

## P57 Action observation training effects on brain structural and functional changes in Parkinson’s disease

### Elisabetta Sarasso^1,3^, Federica Agosta^1^, Mattia Di Meo^1,4^, Mattia Giacobbe^1,4^, Maria Antonietta Volontè^2^, Giancarlo Comi^2^, Andrea Tettamanti^3,4^, Roberto Gatti^5^, Massimo Filippi^1,2^

#### ^1^Neuroimaging Research Unit; ^2^Department of Neurology, Institute of Experimental Neurology, Division of Neuroscience, San Raffaele Scientific Institute, Vita-Salute San Raffaele University; ^3^Laboratory of Movement Analysis, Division of Neuroscience, San Raffaele Scientific Institute, Milan, Italy; ^4^School of Physiotherapy, Vita-Salute San Raffaele University, Milan, Italy; ^5^Hunimed University Physiotherapy Degree Course Rozzano, Milan, Italy

##### **Correspondence:** Elisabetta Sarasso (sarasso.elisabetta@hsr.it)

**Background and Objective:** To assess brain functional and structural changes following action observation training (AOT) associated with exercises of balance, gait, transfers and manual dexterity relative to pure exercises in Parkinson’s disease (PD) patients. (1)

**Materials and Methods:** Twelve PD patients were randomized into two groups: AOT-group and LANDSCAPE-group. In AOT-group, training consisted of AO combined with practicing the observed actions; LANDSCAPE-group performed the same exercises combined with landscape-videos observation. (2) Both groups performed a 4-week training, three times a week, one hour each session. At baseline (T0) and week 4 (W4), patients underwent neurological, neuropsychological, and physiotherapy assessments. 3D T1-weighted, diffusion tensor (DT) magnetic resonance image (MRI) and functional MRI (fMRI) were acquired. fMRI tasks consisted of hand anti-phase movements and motor-imagery of circumstances representing activities of daily living. Clinical evaluations were repeated at 3-month follow-up.

**Results:** At W4, both groups showed changes of the step frequency at spontaneous velocity. The AOT group had an improvement of quality of life at W4 and velocity during manual activities at 3-months. During the hand anti-phase task, AOT-group showed an increased activity of frontal areas and a decreased recruitment of cerebello-thalamo-cortical network, while the LANDSCAPE-group had an increased activity of the thalamus and a decreased recruitment of parietal areas. During the motor-imagery task AOT-group showed a reduced recruitment of the cerebello-thalamo-cortical network and occipital areas, while the LANDSCAPE-group showed an increased activity of motor areas. Only in the AOT-group, functional plasticity was correlated with clinical improvements. Moreover, AOT-group showed an increased white matter integrity of cerebellar peduncles which was correlated to cerebellar functional plasticity.

**Conclusions:** After 4 weeks of training both PD groups showed a brain activity reorganization (3,4) during the fMRI tasks. Only in the AOT-group, functional plasticity was correlated with clinical changes such as improvements in quality of life and velocity during manual activities. Moreover, only the AOT-group showed a correlation between brain functional plasticity and structural changes in white matter tracts belonging to cerebellar areas. The combination between physical and cognitive exercises has the potential to stimulate motor learning and to provide a more long-lasting effect compared to a pure motor training in PD patients.

**References**

1. Buccino G, Gatti R, Giusti MC, Negrotti A, Rossi A, Calzetti S, Cappa SF. Action observation treatment improves autonomy in daily activities in Parkinson's disease patients: results from a pilot study. Mov Disord. 2011 Aug 15;26(10):1963-4.

2. Agosta F, Gatti R, Sarasso E, Volonté MA, Canu E, Meani A, Sarro L, Copetti M, Cattrysse E, Kerckhofs E, Comi G, Falini A, Filippi M. Brain plasticity in Parkinson's disease with freezing of gait induced by action observation training. J Neurol. 2017 Jan;264(1):88-101.

3. Yang J. The influence of motor expertise on the brain activity of motor task performance: A meta-analysis of functional magnetic resonance imaging studies. Cogn Affect Behav Neurosci. 2015 Jun;15(2):381-94.

4. Balser N, Lorey B, Pilgramm S, Stark R, Bischoff M, Zentgraf K, Williams AM, Munzert J. Prediction of human actions: expertise and task-related effects on neural activation of the action observation network. Hum Brain Mapp. 2014 Aug;35(8):4016-34.

## P58 Narrative medicine. “My DBS and Sword of Damocles”: an autobiographical narrative writing experience

### Franca Tirinelli^1^, Fabio Viselli^1^, Maria.Elena Tondinelli^1^, Paola Caruso^2^

#### ^1^Ospedale San Giovanni Battista, Roma; ^2^S.I.Fi.R

##### **Correspondence:** Franca Tirinelli (franca1965@alice.it)

Narrative medicine is a medicine practiced with narrative competence, understood as the ability to recognize the significance of the sick people’s stories listening or reading, to understand and interpret their meaning and to act on these narratives in the conduct of clinical practice” (1). The narration of experience and, in particular, autobiographical writing can be used as intervention treatment, as an instrument, or even as a research technique to collect qualitative data on treatments.

Ivana has been suffering from Parkinson's disease since more than 10 years and, to the worsening of her motor and emotional state no longer controlled by drugs, she has decided to undergo Deep Brain Stimulation (DBS). During hospitalization, she started an autobiographical writing path, that made her more aware of her state of illness and facilitated the choice of intervention.

The DBS is a neurosurgical procedure that involves the implantation of a neurostimulator that sends electrical impulses via electrodes implanted in the basal ganglia for the treatment of movement disorders.

Ivana writes: “There is a time for the DBS. It is the time when it does not make you afraid, until a moment before you think you will never do it, and then something called dignity takes off, which makes you realize that it is the time."

Writing the experience is revealed as a useful tool to understand the complexity of Ivana’s past, her recovery and the evolution of her mind before, during and after her intervention, and has made it possible to understand more deeply the meaning of the experience and to realize the cure process.

Attention to the past and its way of perceiving the disease was a tool of improving the cure and making the therapies more effective.

**Conclusions:** Written narrative has been recognized as an excellent tool to discuss the process of care and approach to the person, to understand patients’ experienced and understand the complexity of related therapy. Autobiographical writing can therefore be a valid tool for learning and collecting data in a narrative-based perspective. (2, 3)

**References**

1. Charon R. Narrative and medicine. N Engl J Med. 2004 Feb 26;350(9):862-4.

2. Brustenghi P, Garrino L, Giarelli G, Lala R, Lombardi Ricci M, Marsico G, Taruscio D, Corea F, Delpiano AM, De Santis M, Dimonte V, Fenocchio G, Gregorino S, Lesmo I, Montanari P, Picco E, Rustighi P, Scapinelli F, Taranto M. Linee di indirizzo per l'utilizzo della medicina narrativa in ambito clinico assistenziale, per le malattie rare e cronico degenerativo. 2015.

3. Gentile AE, Luzi I, Razeto S, Taruscio D (Ed.). Convegno. Medicina narrativa e malattie rare. Istituto Superiore di Sanità. Roma, 26 giugno 2009. Atti. Roma: Istituto Superiore di Sanità; 2009. (Rapporti ISTISAN 09/50).

## P59 Postural control assessment in Autism Spectrum Disorder (ASD) subjects using the Pediatric Balance Scale and the Fall Screen Assessment System: results from a pilot Study

### Giulio Valagussa^1,2^, Luca Trentin^1^, Erica Terragni^2^, Cesare Cerri^2^, Valentina Gariboldi^2^, Cecilia Perin^2^, Davide Mauri^1^, Enzo Grossi^1^

#### ^1^Autism Research Unit, Villa Santa Maria Institute, Tavernerio (CO), Italy; ^2^School of Medicine and Surgery, University of Milano Bicocca, Milano, Italy

##### **Correspondence:** Giulio Valagussa (giulio.valagussa@gmail.com)

**Background and Objectives :** The maintenance of balance depends on the interaction of multiple sensory, motor and integrative systems (i.e. vestibular function, vision, peripheral sensation, muscle force and reaction time). A deficit in any one of these factors may increase the risk of falling. A key sensorimotor control process affected by ASD is the management of upright standing. Few studies on this topic are available in the literature; most of them used instrumental approaches, neglecting the assessment of different balance components. The aims of this pilot study are: 1) to assess balance in a group of ASD subjects using the Pediatric Balance Scale (PBS); 2) to assess balance in the same sample, using the Fall Screen Assessment System (FSAS), comparing the results with a control group of normally developing children.

**Material and Methods:** The ASD sample included nine individuals (mean 12.2 years, 4.29 standard deviation (SD)) diagnosed according to the DSM V criteria and confirmed through ADOS 2; control group included sixteen healthy age subjects (mean 12.8 years, 3.8 SD). We employed: a) FSAS, a multi-item scale internationally validated on adult subjects; b) PBS, a multi-item functional assessment tool exploring functional balance.

**Results:** We found that five ASD subjects (56%) showed a balance deficit as detected by the PBS and were also positive for the FSAS. Two more subjects were found at risk of falling only by FSAS. FSAS showed a statistically significant difference between the two groups in the following tests: visual contrast sensitivity, touch sensitivity, ankle dorsiflexion force, knee extension and flexion force, hand reaction time, and all postural sway tests (Table 1), thus evidencing an overall postural control impairment in ASD.

**Conclusions:** This study confirms that ASD individuals are at major risk of falling. This is attributable to an altered integration and elaboration of sensory and motor information. FSAS integrates the information derived from standard clinical assessment and can be suggested as a complementary tool in the management of ASD. Moreover, by directly assessing an individual’s physiological abilities, intervention strategies can be implemented to target areas of deficit. Further studies are necessary to confirm the results of this pilot study.

**References**

1. Memari AH, Ghanouni P, Shayestehfar M, Ghaheri B. Postural control impairments in individuals with autism spectrum disorder: a critical review of current literature. Asian J Sports Med. 2014 Sep;5(3):e22963.

2. Lord SR, Menz HB, Tiedemann A. A physiological profile approach to falls risk assessment and prevention. Phys Ther. 2003 Mar;83(3):237-52.

**Table 1 (abstract P59).** Fall screen test parameters in the two study groups

Fall Screen Assessment Scale Items	Control group Mean (95%C.I.)	ASD group (CI) Mean (95%C.I.)	T-test independent means
Visual acuity high contrast (MAR) (C.I.)	0.94 (0.15)	1.31 (0.26)	t-value 0.17423 p-value 0.862
Visual acuity low contrast (MAR) (C.I.)	1.79 (0.45)	2.21 (0.36)	t-value 0.70349 p-value 0.485
Contrast sensitivity (dB) (C.I.)	21.63 (0.58)	20.44 (1.02)	t-value 2.40312 p-value 0.025
Depth perception (cm) (C.I.)	1.07 (0.41)	1.69 (0.66)	t-value 0.42532 p-value 0.673
Proprioception (degrees) (C.I.)	2.05 (0.51)	1.80 (0.83)	t-value 0.67554 p-value 0.503
Touch sensitivity (Log 0.1 mg) (C.I.)	3.93 (0.27)	4.42 (0.95)	t-value -2.15436 p-value 0.036
Ankle DF (kg) (C.I.)	13.13 (1.85)	9.89 (3.01)	t-value 2.13406 p-value 0.044
Knee ext (kg) (C.I.)	39.25 (9.22)	22.22 (8.36)	t-value 2.65761 p-value 0.014
Knee flex (kg) (C.I.)	19.38 (3.89)	11.22 (3.3)	t-value 3.04885 p-value 0.006
Reaction time hand (ms) (C.I.)	235.91 (26.34)	385.17 (105.21)	t-value -2.16577 p-value: 0.036
Reaction time foot (ms) (C.I.)	318.34 (36.27)	456.49 (89.76)	t-value -1.78796 p-value 0.081
Sway floor EO (mm) (C.I.)	68.19 (18.73)	195.93 (69.9)	t-value -3.10778 p-value 0.003
Sway floor EC (mm) (C.I.)	98.29 (14.76)	211.01 (81.99)	t-value -2.0911 p-value 0.042
Sway foam EO (mm) (CI)	137.08 (36.17)	598.39 (338.01)	t-value -2.8892 p-value 0.006
Sway foam EC (mm) (C.I.)	278.12 (101.43)	851.77 (434.75)	t-value -2.70773 p-value 0.009
Co-Ordinated stability track (n) (C.I.)	3.45 (3.93)	15.67 (9.98)	t-value -1.81446 p-value 0.077
Maximal balance range (mm) (C.I.)	215.00 (17.86)	172.19 (48.01)	t-value 1.18023 p-value 0.244
TOTAL SCORE (C.I.)	0.28 (0.54)	3.30 (1.17)	t-value -0.884 p-value 0.381

## P60 Standing, walking and running acquisition milestones in Autism Spectrum Disorder (ASD) subjects with Tip-Toe Behavior: a cohort study

### Giulio Valagussa, Valeria Balatti, Luca Trentin, Enzo Grossi

#### Autism Research Unit, Villa Santa Maria Institute, Tavernerio (Como), Italy

##### **Correspondence:** Giulio Valagussa (giulio.valagussa@gmail.com)

**Background and Objective:** Twenty-thirty percent of individuals with autism walk on their tiptoes. (1) In a previous study, we found that this behaviour transpires not only during walking but also while standing and running. (2) Systematic observations about the natural history of Tip-toe Behavior (TTB) in ASD subjects are scarce. The aims of this retrospective study are: 1) to describe when TTB ASD subjects started to stand, walk and run compared to both normal population and non-TTB ASD subjects; 2) to observe if TTB was exhibited simultaneously or subsequently to the acquisition of standing, walking and running milestones.

**Material and Methods:** Our study included 36 ASD subjects (34 males; mean age: 14.3 years, 3.22 standard deviation (SD)) diagnosed with Autism according to DSM V criteria, confirmed through ADOS 2 under observation at our Institute. We collected information about standing, walking and running milestones, if and when TTB was observed and when it eventually stopped using a structured interview to parents. Another therapist confirmed the presence of TTB using a standardized method we described previously. (2)

**Results:** We found that 18 subjects (50%) never showed TTB, 13 TTB subjects (36%) presented TTB at least in one of three previous described situations, while 5 subjects (14%) had TTB in the past but it later stopped. The age of standing acquisition of the ASD sample resulted in line with the normative values3, without significant differences between TTB and non-TTB subjects (Table 1). The age of walking and running acquisition of the ASD sample resulted significantly higher compared to the normative values3,4 (16.4 months, 5.55 SD Vs. 12.1 months, 1.8 SD, and 26.55 months, 14.5 SD Vs. 15 months, 11.8 SD respectively) without significant differences between TTB and non-TTB subjects. We observed that Tip-toe behaviour in TTB subjects started significantly later than the acquisition of standing and walking milestone. Conversely, there was no significant difference between running acquisition and the start of TTB while running.

**Conclusions:** The ASD sample showed a delay in walking and running acquisition compared to the normative values. TTB subjects exhibit this behaviour significantly later to the acquisition of standing and walking milestones

**References**

1. Barrow WJ, Jaworski M, Accardo PJ. Persistent toe walking in autism. J Child Neurol. 2011 May;26(5):619-21.

2. Valagussa G, Trentin L, Balatti V, Grossi E. Assessment of presentation patterns, clinical severity, and sensorial mechanism of tip-toe behavior in severe ASD subjects with intellectual disability: A cohort observational study. Autism Res. 2017 Sep;10(9):1547-57

3. WHO Multicentre Growth Reference Study Group. WHO Motor Development Study: windows of achievement for six gross motor development milestones. Acta Paediatr Suppl. 2006 Apr;450:86-95.

4. Mangani C, Cheung YB, Maleta K, Phuka J, Thakwalakwa C, Dewey K, Manary M, Puumalainen T, Ashorn P. Providing lipid-based nutrient supplements does not affect developmental milestones among Malawian children. Acta Paediatr. 2014 an;103(1):e17-26.

**Table 1 (abstract P60).** Milestone acquisition ages in the sample and in the two subgroups

	All sample	No-TTB subjects	TTB subjects	Normative Values^3.4^
N° of subjects	36	18	18	
Mean Age of the sample (years) (SD)	14.21 (3.22)	14.77 (3.78)	13.64 (2.52)	
Age Range of the sample (years)	6.4 - 21.3	6.4 - 21.3	6.4 - 16.8	
Standing Acquisition mean (months) (SD)	10.92 (3.56)	10.67 (3.12)	11.17 (4.03)	11 (1.9)
Standing acquisition range (months)	6-24	8-18	6-24	
Walking acquisition mean (months) (SD)	16.4 (5.55)	16.25 (6.55)	16.56 (4.53)	12.1 (1.8)
Walking acquisition range (months)	9-30	9-30	9-24	
Running acquisition mean (months) (SD)	12-72	12-36	15-72	

## P61 Pain in the periscaphoid area. Diagnosis criteria of a scaphoid fracture and application of clinical reasoning in manual therapy

### Mirko Zitti, Sara Di Serio

**Background and Objective:** In the wrist-hand area, in a limited anatomical space in comparison with other body areas, where a lot of tendon, bone and muscle structures go with, it is therefore quite difficult to make an accurate differential diagnosis. In particular, other diseases can cause pain in the periscaphoid area and provoke a bunch of misunderstood scaphoid fractures. The purpose of this study is, through a literature review, to identify the diagnostic criteria, the more valid according to the principles of Evidence-based medicine, in order to identify scaphoid fractures.

**Materials and Methods:** Resources data: from December 2015 to March 2016, a review of the literature has been conducted consulting electronic databases of PubMed and Scopus using a combination of the following search terms: “scaphoid bone”, “clinical diagnostic evaluation”, “clinical evaluation”, “physical examination”, “examination tests”, “scaphoid fracture”, “acute scaphoid fractures”, “wrist injuries”, “anatomic snuff-box tenderness”, “longitudinal thumb compression”, “scaphoid tubercle tenderness”.

Review methods: all articles written in English or Italian were selected, with no limits concerning the study design.

**Results:** 20 articles regarding the physical examination of the scaphoid suspected fractures have been included.

**Conclusion:** The more sensitive and specific clinical tests to identify a fracture of the scaphoid are: pressure pain in the anatomical snuffbox (Snuffbox Tenderness Test), pain on axial compression of the thumb (Thumb axial compression Test), deficits in grip strength (Grip hand force Test) and pain in the pronation of the forearm (Forearm pronation Test).

The presence of swelling and hematoma in the region of the wrist associated with the positivity of some clinical tests represent a valid support for physical therapist, examining a patient with direct access and history-taking of trauma on the wrist, in the absence of comorbidity or major clinical signs of severe pathology.

